# Financial hardship associated with catastrophic out-of-pocket spending tied to primary care services in low- and lower-middle-income countries: findings from a modeling study

**DOI:** 10.1186/s12916-023-02957-w

**Published:** 2023-09-14

**Authors:** Sarah Bolongaita, Yeeun Lee, Kjell Arne Johansson, Øystein A. Haaland, Mieraf Taddesse Tolla, Jongwook Lee, Stéphane Verguet

**Affiliations:** 1 Department of Global Health and Population, Harvard T.H. Chan School of Public Health, 677 Huntington Avenue, Boston, MA 02115 USA; 2 Department of Global Public Health and Primary Care, University of Bergen, Pb. 7804, NO-5020, Bergen, Norway; 3 Addis Center for Ethics and Priority Setting, Addis Ababa University, Addis Ababa, Ethiopia; 4 Africa Centers for Disease Control and Prevention, Addis Ababa, Ethiopia; 5 Department of Agricultural Economics and Rural Development, Seoul National University, Seoul, South Korea

**Keywords:** Universal health coverage, Financial risk protection, Out-of-pocket medical costs, Catastrophic health expenditures, Priority setting

## Abstract

**Background:**

Financial risk protection (FRP) is a key component of universal health coverage (UHC): all individuals must be able to obtain the health services they need without experiencing financial hardship. In many low-income and lower-middle-income countries, however, the health system fails to provide sufficient protection against high out-of-pocket (OOP) spending on health services. In 2018, OOP health spending comprised approximately 40% of current health expenditures in low-income and lower-middle-income countries.

**Methods:**

We model the household risk of catastrophic health expenditures (CHE), conditional on having a given disease or condition—defined as OOP health spending that exceeds a threshold percentage (10, 25, or 40%) of annual income—for 29 health services across 13 disease categories (e.g., diarrheal diseases, cardiovascular diseases) in 34 low-income and lower-middle-income countries. Health services were included in the analysis if delivered at the primary care level and part of the *Disease Control Priorities*, 3rd edition “highest priority package.” Data were compiled from several publicly available sources, including national health accounts, household surveys, and the published literature. A risk of CHE, conditional on having disease, was modeled as depending on usage, captured through utilization indicators; affordability, captured via the level of public financing and OOP health service unit costs; and income.

**Results:**

Across all countries, diseases, and health services, the risk of CHE (conditional on having a disease) would be concentrated among poorer quintiles (6.8% risk in quintile 1 vs. 1.3% in quintile 5 using a 10% CHE threshold). The risk of CHE would be higher for a few disease areas, including cardiovascular disease and mental/behavioral disorders (7.8% and 9.8% using a 10% CHE threshold), while lower risks of CHE were observed for lower cost services.

**Conclusions:**

Insufficient FRP stands as a major barrier to achieving UHC, and risk of CHE is a major problem for health systems in low-income and lower-middle-income countries. Beyond its threat to the financial stability of households, CHE may also lead to worse health outcomes, especially among the poorest for whom both ill health and financial risk are most severe. Modeling the risk of CHE associated with specific disease areas and services can help policymakers set progressive health sector priorities. Decision-makers could explicitly include FRP as a criterion for consideration when assessing the health interventions for inclusion in national essential benefit packages.

**Supplementary Information:**

The online version contains supplementary material available at 10.1186/s12916-023-02957-w.

## Background

Universal health coverage (UHC) is the key programmatic engine for Sustainable Development Goal (SDG) 3, which aims to ensure healthy lives and promote well-being for all individuals of all ages [1]. Achieving UHC (SDG target 3.8) requires that national health systems provide citizens with both high-quality essential health services and adequate financial risk protection (FRP). Individuals must be able to access the health services they need without experiencing financial hardship. In many low- and middle-income countries (LMICs), however, FRP is severely lacking. In 2018, out-of-pocket (OOP) health spending comprised nearly 40% of current health expenditures in LMICs on average [2]. This has important implications for household wellbeing, with millions of poor families forced to weigh the tradeoffs between health and financial stability [3]. For example, in Ethiopia, the cost of health services has been found to factor heavily into families’ decisions to seek treatment for newborns: while illness was recognized as dangerous for the newborn, families had reasonable concern that seeking healthcare could threaten the economic survival of the entire family [4].

At the household level, financial risk is primarily due to high OOP costs for health services. In turn, at the health system level, high OOP costs are often driven by low government expenditures on health (i.e., government expenditures comprise a relatively small percentage of total health expenditures) [2, 5, 6]. The lack of FRP is commonly measured using two threshold-based indicators: catastrophic health expenditures (CHE), which occur when OOP health spending exceeds a given threshold (usually 10 or 25%) of total household income or consumption, and impoverishing health expenditures (IHE), which occur when households are pushed below a given poverty line (e.g., the international poverty line of $1.90 per day at purchasing power parity (PPP)) as a result of OOP health spending [5–7].

The global prevalence of CHE (using a 10% threshold) increased from 10% in 2000 to 12% in 2015, with the largest increases occurring in Africa and Asia (3% and 2% increase, respectively) [5]. In 2015, the year the SDGs were adopted, an estimated 927 million people experienced CHE (10% threshold) [2]. In Ethiopia, 36% of the country’s total health expenditures are paid for out of pocket [2]. At the household level, this can be reflected by OOP costs ranging from $6 to $65 (USD 2016) for diarrhea treatment and $8 to $52 for pneumonia treatment, for example [8]. As a result, approximately 2.0% of the Ethiopian population (roughly 2 million people) can experience CHE (at a 10% threshold), although regional rates can be substantially higher (e.g., 5.8% in Afar, Ethiopia) [9]. Despite global efforts to improve FRP through UHC, it is predicted that global incidence of CHE will continue to rise until 2030 [7].

The extent of OOP health spending can vary drastically across types of health services and disease areas [10–12]. This is especially true in countries with developing health systems, where there is often limited provision of even basic health services [13]. Understanding OOP health spending by disease area can provide important policy implications for reducing household financial burden and achieving overarching UHC objectives. For example, countries could invest in financing specific health services or mandate increases in insurance coverage for certain health services based on their relative contribution to the population’s experience of CHE. However, few studies have pursued a systematic assessment of disease-specific OOP health spending associated with CHE in LMICs [10, 14, 15].

Through this research, we intend to model which disease areas and conditions could pose the greatest financial risks to individuals when seeking treatment at the primary care level in LMICs, with the hypothesis that the diseases that are more expensive to treat or have limited public financing would lead to higher risks of CHE. We model the risk of CHE due to disease-specific OOP health spending, conditional on having a given disease, in 34 low-income and lower-middle-income countries. Our intent is to accordingly provide modeled estimates on what might be the extent of FRP benefits (materialized here by reductions in those risks of CHE, conditional on having a given disease) provided by publicly financing key primary care services. In doing so, this may be helpful in conceptualizing the design of publicly financed essential health services packages that fully deliver on FRP [16].

## Methods

Our mathematical model utilized inputs on simulated income, health service utilization, and OOP spending to estimate the risk of CHE, conditional on having a given disease, for primary care services in 34 low- and lower-middle-income countries. We disaggregated our computation by wealth quintile, disease area, and health service to provide insight into the conditions that would pose the greatest risk for CHE, and to identify who, in terms of socioeconomic group, would be most at risk for experiencing CHE.

### Disease-specific OOP spending inputs

We sourced and reviewed national health account (NHA) reports to derive inputs on disease-specific OOP spending. The most comprehensive and consistent data on health financing are generated from NHAs, which collect health expenditure information within an internationally recognized framework; however not all countries maintain or update NHAs, nor do all countries report OOP health expenditures by disease category [17, 18]. Limiting our search of NHA reports to years 2010 or later, we obtained disease-specific OOP health spending data for 34 countries (17 low-income countries and 17 lower-middle-income countries according to the World Bank’s 2022 income group classifications; see Table 1, Additional file 1: Table A.1) and 13 disease areas (“childhood health,” “diarrheal diseases,” “HIV/AIDS & other sexually transmitted infections” (STIs), “malaria,” “other infectious & parasitic diseases,” “tuberculosis” (TB), “cardiovascular diseases” (CVD), “endocrine & metabolic disorders,” “mental/behavioral disorders & neurological conditions,” “other noncommunicable diseases” (NCDs), “contraceptive management (family planning),”^1^ “maternal conditions,” and “perinatal conditions”). Disease areas, originally defined by the World Health Organization’s (WHO) Global Health Expenditure Database (GHED), were classified into four broad categories (childhood health, infectious and parasitic diseases, NCDs, and reproductive health; see Additional file 1: Table A.2) [19]. We directly calculated the percent of disease-specific OOP expenditures paid by households whenever data was available from a country’s NHA report. If unavailable, we used a regional average instead (Additional file 1: Table A.3).

**Table 1:** Study countries by income group (according to World Bank 2022 income group classifications), presented with descriptive and financial risk protection indicators for the year of the country’s NHA, unless otherwise indicated in parentheses. The presented health expenditure indicators report aggregate (i.e., not disease- or wealth quintile-specific) estimates at the national level. Data are available from the World Bank’s World Development Indicators (WDI) database

Country	NHA year	GNI	Gini coefficient	Domestic health expenditures		OOP health expenditures	
		Per capita (2016 USD)		% total health expenditures	Per capita (2016 USD)	% total health expenditures	Per capita (2016 USD)
Low-income							
Afghanistan	2014	$558	-	5.0%	$3	73.1%	$38
Burkina Faso	2016	$602	47.3 (2018)^a^	40.1%	$15	31.4%	$11
Burundi	2013	$230	38.6	18.0%	$4	21.1%	$4
Cambodia	2014	$903	-	18.5%	$12	58.6%	$38
Congo, Democratic Republic of the	2018	$443	42.1 (2012)^a^	15.1%	$2	41.6%	$7
Ethiopia	2011	$345	35.0 (2015)^a^	8.5%	$1	46.5%	$6
Gambia, Republic of the	2015	$549	35.9	31.4%	$6	23.4%	$4
Guinea	2014	$655	29.6 (2018)^a^	12.8%	$4	58.4%	$19
Malawi	2015	$310	38.5 (2019)^a^	28.6%	$9	11.0%	$3
Mali	2014	$717	36.1 (2018)^a^	19.9%	$7	33.6%	$11
Mozambique	2015	$567	54.0 (2014)^a^	27.0%	$9	11.3%	$4
Nepal	2016	$779	32.8 (2010)^a^	18.6%	$8	55.4%	$24
Niger	2015	$496	37.3 (2018)^a^	20.8%	$5	51.7%	$12
Sierra Leone	2013	$585	35.7 (2018)^a^	7.0%	$5	62.9%	$46
Tanzania	2015	$868	40.5 (2018)^a^	34.4%	$10	25.8%	$8
Uganda	2016	$709	42.8	15.7%	$5	38.6%	$14
Zimbabwe	2010	$620	50.3 (2019)^a^	26.3%	$23	34.4%	$30
Lower-middle-income							
Armenia	2016	$3330	32.5	16.5%	$52	80.6%	$255
Benin	2013	$1081	37.8 (2018)^a^	26.7%	$8	42.3%	$13
Cabo Verde	2016	$2772	42.4 (2015)^a^	63.6%	$85	28.7%	$39
Cameroon	2011	$1258	46.6 (2014)^a^	14.9%	$7	69.7%	$35
Congo, Republic of the	2015	$2746	48.9 (2011)^a^	45.5%	$24	32.5%	$17
Côte D’Ivoire	2014	$1789	37.2 (2018)^a^	20.8%	$15	51.1%	$36
Ghana	2015	$1718	43.5 (2016)^a^	35.1%	$25	35.8%	$26
Haiti	2014	$1267	41.1 (2012)^a^	10.4%	$6	31.0%	$18
Kenya	2016	$1293	40.8 (2015)^a^	42.8%	$27	25.0%	$16
Lao People’s Democratic Republic	2012	$1213	36.0	21.0%	$6	48.6%	$14
Myanmar	2018	$1081	30.7 (2017)^a^	14.8%	$8	76.4%	$40
Nigeria	2016	$2152	35.1 (2018)^a^	13.0%	$9	75.2%	$53
Samoa	2015	$3508	38.7 (2013)^a^	79.1%	$156	11.5%	$23
São Tomé & Príncipe	2013	$1267	40.7 (2017)^a^	33.5%	$37	14.3%	$16
Senegal	2013	$1187	38.1 (2018)^a^	26.7%	$14	55.2%	$29
Tajikistan	2013	$1178	34.0 (2015)^a^	29.1%	$18	61.9%	$38
Viet Nam	2015	$2179	35.7 (2018)^a^	41.8%	$44	43.5%	$45

*GNI* gross national income, *NHA* national health account, *OOP* out-of-pocket

^a^Data from NHA year are not available; data from most recent year available presented

- Data unavailable

### Health service costs inputs

Health service unit cost inputs were adapted from the *Disease Control Priorities*, 3rd edition (DCP3) project [20–22]. Unit costs were available for 242 health services in low-income countries and 246 health services in lower-middle-income countries. Here, we included the 29 services to be delivered at the health center level as included in DCP3’s “highest priority package.” According to DCP3, this package includes services that have good value for money, address the health needs of the worse off (i.e., those with the least lifetime health), or are likely to offer substantial FRP (Additional file 1: Table A.4) [20, 22]. Health services were classified according to disease area (group) using expert opinion.

### Health services utilization inputs

Indicators of health services utilization included in the model captured both demand- and supply-side dimensions of healthcare access. However, health service-specific utilization data are rarely routinely and systematically collected nor available, so we had to rely on proxy indicators of health services utilization from various sources: the Demographic and Health Surveys (DHS) [23], the WHO STEPwise Approach to Surveillance (STEPS) reports [24], the World Bank’s World Development Indicators (WDI) and Health Equity and Financial Protection Indicators (HEFPI) databases [2], as well as the published literature (Additional file 1: Table A.5). If data for a country were not available for a given indicator, a regional average was used instead (Additional file 1: Table A.6). Most inputs for health services utilization were available disaggregated by wealth quintile. However, if no empirical gradient between quintiles was available for a utilization input by country or region (for example, for inputs derived from the literature), a country’s average wealth quintile gradient was then used.

### Simulated income distributions

We simulated income distributions for each country: we created gamma distributions using gross national income (GNI) per capita as a proxy for average income and the Gini index as a measure of inequality (Table 1) [25]. Estimates on these indicators (GNI, Gini) were available from the World Bank’s WDI database for most country-years. If estimates were not available for the year of a country’s NHA, the most recent country-year estimate was used. If no estimates were available after 2010, a regional average was used (Additional file 1: Table A.7) [2, 26]. To align simulated income with utilization inputs, which were disaggregated by wealth quintile, we assigned quintiles to the simulated income distributions (a process which involved ordering incomes from lowest to highest and then splitting the sorted incomes into fifths, such that each quintile had the same proportion, 20%, of the number of simulated incomes)^2^.

### Modeling approach

Because the various input data sources employed different terminologies and captured information on different aspects of the health system, we needed to link the datasets together manually. As described above, DCP3 provided a list of costed health services and NHA reports provided a list of disease groups from their line-item accounting. Health services were linked to disease groups based on the primary disease that a given service addressed. Health services were then evaluated to identify utilization proxy inputs commonly available in public datasets (e.g., DHS, STEPs). After assessing input data availability, the most suitable utilization input was selected for each health service, with preference given to utilization inputs disaggregated by wealth quintile. See Additional file 1: Table A.8 for details on data linkage.

For each country, a population was simulated with incomes drawn from the country’s simulated income distribution (as described above). Contingent upon needing a health intervention/service (i.e., having the disease), quintile-specific utilization rates were applied to the simulated population. Only those individuals who were modeled as utilizing health services, then incurred OOP payment amounts for receiving those services were assigned. These OOP payment amounts were calculated by multiplying the health service’s unit cost by the relevant disease-specific percent of total health expenditures paid for out of pocket. The OOP payments were then compared with the simulated incomes to determine if CHE occurred using three different thresholds (10%, 25%, and 40%). At the 10% threshold, for example, CHE would occur if the OOP payment amount exceeded 10% of the simulated annual income. The risk of CHE was then modeled by calculating the proportion of 1000 individuals with the disease that experienced CHE, averaged over *n* = 1000 simulated samples.

All monetary values were converted to 2016 USD^3^ using the US Bureau of Labor Statistics consumer price index (https://data.bls.gov/timeseries/CUUR0000SA0) and World Bank exchange rates (https://data.worldbank.org/indicator/PA.NUS.FCRF). All computations and simulations were conducted using R software (www.r-project.org).

## Results

**Fig. 1:**
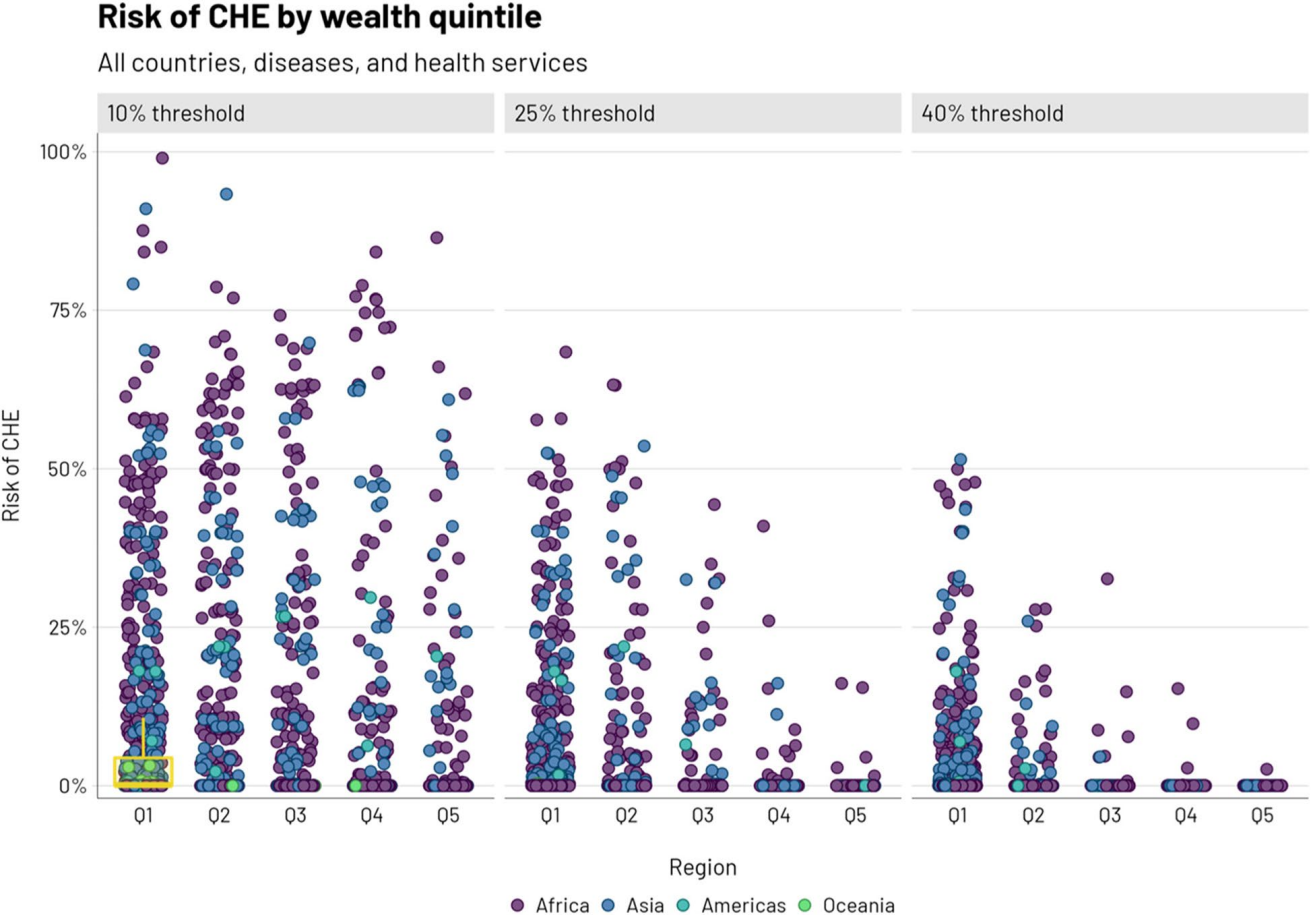
Risk of catastrophic health expenditures (CHE, conditional on having disease or condition) at three thresholds of income (10, 25, and 40%) by wealth quintile. Points represent all countries, diseases, and health services and are colored by region. A boxplot is depicted if the 75th percentile of a given CHE risk distribution surpasses 1%

We report on the estimated risk of CHE due to health service- and disease-specific OOP spending in 34 low-income and lower-middle-income countries. Across all countries and health services, OOP amounts ranged from nothing ($0.00) to $309.60 (see Additional file 1: Tables B1-B34). On average across all countries, quintiles, and health services, the lowest OOP amounts were found for childhood health conditions ($0.09) and the highest for CVD ($79.93). The risk of CHE (conditional on having disease or condition) was systematically concentrated among poorer quintiles (Fig. 1). On average, across all countries and diseases, the poorest quintile would have a CHE risk (conditional on having disease or condition) of 6.8% (at a 10% threshold), compared to the richest quintile who would have a CHE risk (conditional on having disease or condition) of 1.3% (Table 2).

**Table 2:** Risk of catastrophic health expenditures (CHE, conditional on having disease or condition) in all study countries at two thresholds (10 and 25%) by disease and wealth quintile. Q1 indicates the first quintile (the poorest); Q5 indicates the fifth quintile (the richest)

	Risk of CHE 10% threshold (25% threshold in parentheses)					
**Disease**	**Q1**	**Q2**	**Q3**	**Q4**	**Q5**	**Total**
Childhood health	0.0 (0.0)	0.0 (0.0)	0.0 (0.0)	0.0 (0.0)	0.0 (0.0)	0.0 (0.0)
Infectious and parasitic diseases	6.4 (2.5)	3.5 (0.4)	2.1 (0.0)	1.2 (0.0)	0.3 (0.0)	2.7 (0.6)
Diarrheal diseases	0.4 (0.0)	0.0 (0.0)	0.0 (0.0)	0.0 (0.0)	0.0 (0.0)	0.1 (0.0)
HIV/AIDS and other sexually transmitted diseases	6.2 (2.2)	3.1 (0.3)	1.4 (0.0)	1.0 (0.0)	0.3 (0.0)	2.4 (0.5)
Malaria	0.0 (0.0)	0.0 (0.0)	0.0 (0.0)	0.0 (0.0)	0.0 (0.0)	0.0 (0.0)
Tuberculosis	26.0 (11.2)	16.0 (2.0)	11.0 (0.0)	5.2 (0.0)	1.2 (0.0)	11.9 (2.6)
Other infectious and parasitic diseases	0.0 (0.0)	0.0 (0.0)	0.0 (0.0)	0.0 (0.0)	0.0 (0.0)	0.0 (0.0)
Noncommunicable diseases (NCDs)	10.9 (7.0)	9.5 (3.8)	7.6 (1.5)	6.2 (0.4)	3.7 (0.1)	7.6 (2.6)
Cardiovascular diseases	9.5 (7.3)	9.6 (4.4)	8.1 (2.8)	7.1 (1.3)	4.9 (0.4)	7.8 (3.2)
Endocrine and metabolic disorders	5.1 (1.9)	3.1 (0.1)	1.3 (0.0)	0.6 (0.0)	0.0 (0.0)	2.0 (0.4)
Mental/behavioral disorders and neurological conditions	14.7 (9.1)	12.2 (5.0)	9.9 (1.3)	8.0 (0.1)	4.4 (0.0)	9.8 (3.1)
Other NCDs	2.2 (1.1)	1.6 (0.0)	1.1 (0.0)	0.1 (0.0)	0.0 (0.0)	1.0 (0.2)
Reproductive health	3.8 (1.3)	2.7 (0.0)	0.9 (0.0)	0.2 (0.0)	0.0 (0.0)	1.5 (0.3)
Family planning	0.0 (0.0)	0.0 (0.0)	0.0 (0.0)	0.0 (0.0)	0.0 (0.0)	0.0 (0.0)
Maternal conditions	6.1 (2.1)	4.5 (0.0)	1.4 (0.0)	0.4 (0.0)	0.0 (0.0)	2.5 (0.4)
Perinatal conditions	0.2 (0.0)	0.0 (0.0)	0.0 (0.0)	0.0 (0.0)	0.0 (0.0)	0.0 (0.0)
Average	6.8 (3.6)	5.2 (1.4)	3.5 (0.5)	2.5 (0.1)	1.3 (0.0)	3.9 (1.1)

**Fig. 2:**
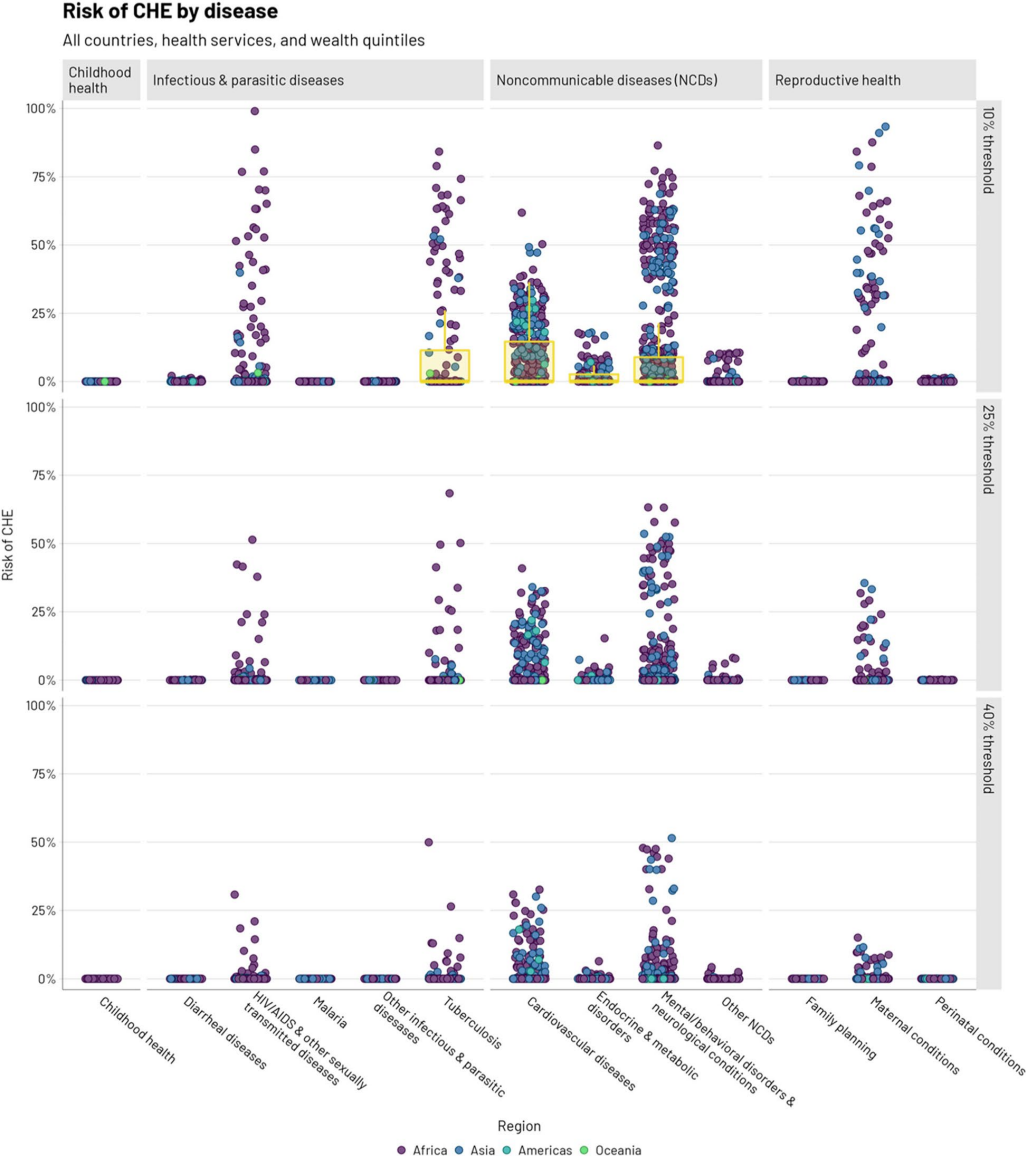
Risk of catastrophic health expenditures (CHE, conditional on having disease or condition) at three thresholds of income (10, 25, and 40%) by broad disease area. Points represent all countries, health services, and wealth quintiles and are colored by region. A boxplot is depicted if the 75th percentile of a given CHE risk distribution surpasses 1%

Higher CHE risk would be faced when seeking NCD services, notably for mental/behavioral disorders and neurological conditions (9.8% at a 10% threshold, 3.1% at a 25% threshold) and CVD (7.8% at a 10% threshold, 3.2% at a 25% threshold) (Fig. 2, Table 2). Disease categories for which lower shares of OOP spending within total health expenditures were observed in the NHAs (e.g., childhood health, infectious and parasitic diseases, maternal health) would have lower CHE risks. However, within the infectious and parasitic disease group, HIV/AIDS (2.4% at a 10% threshold, 0.5% at a 25% threshold) and TB (11.9% at a 10% threshold, 2.6% at a 25% threshold) services would pose the greatest CHE risk. Within reproductive health, maternal conditions (2.5% at a 10% threshold, 0.4% at a 25% threshold) would pose the greatest CHE risk.

**Fig. 3:**
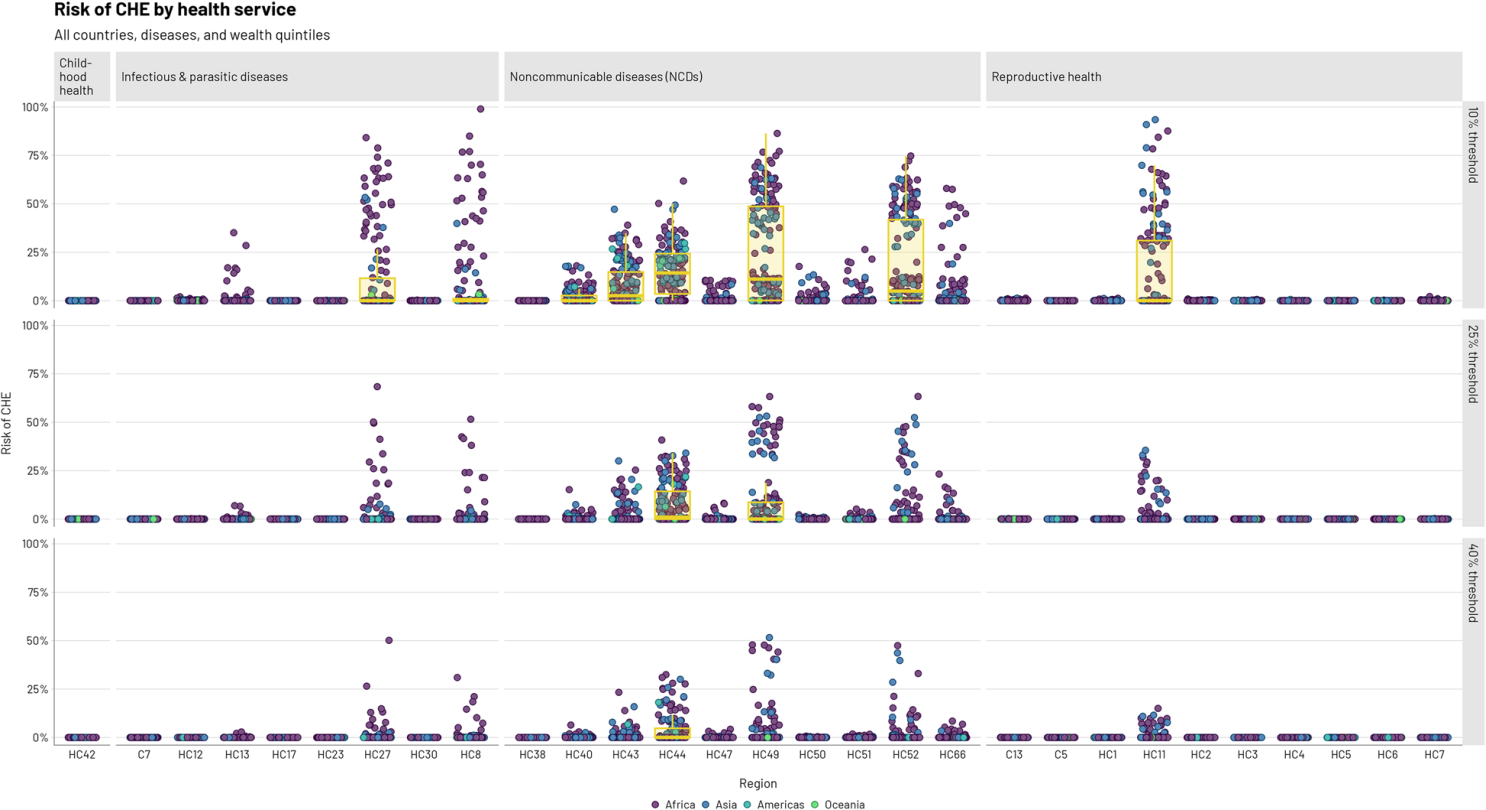
Risk of catastrophic health expenditures (CHE, conditional on having disease or condition) at three thresholds of income (10, 25, and 40%) by health service type. Points represent all countries, diseases, and wealth quintiles and are colored by region. A boxplot is depicted if the 75th percentile of a given CHE risk distribution surpasses 1%. For descriptions of health services listed on the x-axis see Table 3

Although most of the health services examined in our analysis would not contribute substantially to CHE risk, seven services would be associated with a CHE risk of more than 5% (when using a 10% threshold; Fig. 3, Table 3). Only one of these was for reproductive health (basic emergency newborn and obstetric care: CHE risk of 14.9% at a 10% threshold), whereas four were concerning NCDs. Management of heart failure, bipolar disorder, and schizophrenia all would have CHE risks greater than 10% (at a 10% threshold). Among infectious and parasitic disease services, diagnosis and treatment of TB would pose the highest CHE risk (11.9%) followed by prevention of maternal to child transmission of HIV and syphilis (8.7%). It is important to note that CHE would still occur quite frequently using a higher 40% threshold.

**Table 3:** Risk of catastrophic health expenditures (CHE, conditional on having disease or condition) in all countries at two thresholds of income (10 and 25%) by health service type and wealth quintile. The unit cost of health services is noted underneath health service descriptions; estimates on health service unit costs were available by country income group: low-income (LI) and lower-middle-income (LMI). See Additional file 1: Table A.4 for detailed health service descriptions

Health service type		Risk of CHE 10% threshold (25% threshold in parentheses)					
Code	Description	Q1	Q2	Q3	Q4	Q5	Total
Childhood health							
HC42	Acute pharyngitis treatment	0.0 (0.0)	0.0 (0.0)	0.0 (0.0)	0.0 (0.0)	0.0 (0.0)	0.0 (0.0)
	LI $0.17, LMI $0.24						
Infectious & parasitic diseases							
C7	Intermittent preventive treatment (pregnancy)	0.0 (0.0)	0.0 (0.0)	0.0 (0.0)	0.0 (0.0)	0.0 (0.0)	0.0 (0.0)
	LI $0.45, LMI $1.02						
HC8	HIV & syphilis PMTCT	20.6 (8.1)	11.9 (1.3)	5.8 (0.0)	4.1 (0.0)	1.1 (0.0)	8.7 (1.9)
	LI $176.35, LMI $313.51						
HC12	Diagnosis & treatment of infections (IMCI)	0.4 (0.0)	0.0 (0.0)	0.0 (0.0)	0.0 (0.0)	0.0 (0.0)	0.1 (0.0)
	LI $4.79, LMI $10.29						
HC13	ART and viral load monitoring	4.3 (0.7)	0.5 (0.0)	0.0 (0.0)	0.0 (0.0)	0.0 (0.0)	1.0 (0.1)
	LI $71.45, LMI $121.57						
HC17	Syndromic management of STIs	0.0 (0.0)	0.0 (0.0)	0.0 (0.0)	0.0 (0.0)	0.0 (0.0)	0.0 (0.0)
	LI $5.67, LMI $10.69						
HC23	HIV, STIs, hepatitis testing, and counseling	0.0 (0.0)	0.0 (0.0)	0.0 (0.0)	0.0 (0.0)	0.0 (0.0)	0.0 (0.0)
	LI $4.31, LMI $6.08						
HC27	Diagnosis and treatment of TB	26.0 (11.2)	16.0 (2.0)	11.0 (0.0)	5.2 (0.0)	1.2 (0.0)	11.9 (2.6)
	LI $135.09, LMI $175.65						
HC30	Management and referrals for fever (IMAI)	0.0 (0.0)	0.0 (0.0)	0.0 (0.0)	0.0 (0.0)	0.0 (0.0)	0.0 (0.0)
	LI $3.11, LMI $6.83						
Noncommunicable diseases (NCDs)							
HC38	Aspirin for acute myocardial infarction	0.0 (0.0)	0.0 (0.0)	0.0 (0.0)	0.0 (0.0)	0.0 (0.0)	0.0 (0.0)
	LI $0.03, LMI $0.05						
HC40	Screening and management of diabetes	5.1 (1.9)	3.1 (0.1)	1.3 (0.0)	0.6 (0.0)	0.0 (0.0)	2.0 (0.4)
	LI $64.16, LMI $92.52						
HC43	Management of ischemic heart disease	13.8 (8.5)	13.3 (1.6)	8.8 (0.1)	4.6 (0.0)	0.9 (0.0)	8.3 (2.1)
	LI $83.97, LMI $190.17						
HC44	Management of heart failure	14.6 (13.2)	15.4 (11.7)	15.7 (8.4)	16.7 (3.9)	13.8 (1.1)	15.2 (7.7)
	LI $249.96, LMI $342.48						
HC47	Palliative care	2.2 (1.1)	1.6 (0.0)	1.1 (0.0)	0.1 (0.0)	0.0 (0.0)	1.0 (0.2)
	LI $64.63, LMI $21.32						
HC49	Management of bipolar disorder	26.2 (22.8)	27.2 (17.1)	27.4 (5.5)	26.9 (0.4)	16.3 (0.0)	24.8 (9.2)
	LI $184.57, LMI $365.39						
HC50	Management of depression	3.6 (0.4)	0.0 (0.0)	0.0 (0.0)	0.0 (0.0)	0.0 (0.0)	0.7 (0.1)
	LI $16.11, LMI $48.02						
HC51	Management of epilepsy	5.5 (0.8)	0.3 (0.0)	0.0 (0.0)	0.0 (0.0)	0.0 (0.0)	1.2 (0.2)
	LI $27.53, LMI $53.73						
HC52	Management of schizophrenia	25.8 (17.9)	26.9 (7.6)	20.3 (1.0)	13.1 (0.0)	5.8 (0.0)	18.4 (5.3)
	LI $99.43, LMI $329.34						
HC66	Psychosocial support and counseling	12.2 (3.8)	6.7 (0.0)	1.6 (0.0)	0.2 (0.0)	0.0 (0.0)	4.1 (0.8)
	LI $64.63, LMI $21.32						
Reproductive health							
C5	Antenatal tetanus immunization	0.0 (0.0)	0.0 (0.0)	0.0 (0.0)	0.0 (0.0)	0.0 (0.0)	0.0 (0.0)
	LI $0.39, LMI $0.44						
C13	Cotrimoxazole for HIV-exposed children	0.3 (0.0)	0.0 (0.0)	0.0 (0.0)	0.0 (0.0)	0.0 (0.0)	0.1 (0.0)
	LI $6.55, LMI $11.95						
HC1	Antibiotics for neonatal pneumonia	0.2 (0.0)	0.0 (0.0)	0.0 (0.0)	0.0 (0.0)	0.0 (0.0)	0.0 (0.0)
	LI $6.22, LMI $6.61						
HC2	Post-abortion care	0.2 (0.0)	0.0 (0.0)	0.0 (0.0)	0.0 (0.0)	0.0 (0.0)	0.0 (0.0)
	LI $4.54, LMI $8.23						
HC3	Treatment of premature membrane rupture	0.1 (0.0)	0.0 (0.0)	0.0 (0.0)	0.0 (0.0)	0.0 (0.0)	0.0 (0.0)
	LI $4.04, LMI $4.66						
HC4	Contraceptives	0.0 (0.0)	0.0 (0.0)	0.0 (0.0)	0.0 (0.0)	0.0 (0.0)	0.0 (0.0)
	LI $4.97, LMI $10.32						
HC5	Kangaroo mother care counseling	0.0 (0.0)	0.0 (0.0)	0.0 (0.0)	0.0 (0.0)	0.0 (0.0)	0.0 (0.0)
	LI $2.33, LMI $4.61						
HC6	Neonatal sepsis, pneumonia, meningitis	0.0 (0.0)	0.0 (0.0)	0.0 (0.0)	0.0 (0.0)	0.0 (0.0)	0.0 (0.0)
	LI $2.66, LMI $3.51						
HC7	Medical abortion	0.2 (0.0)	0.0 (0.0)	0.0 (0.0)	0.0 (0.0)	0.0 (0.0)	0.0 (0.0)
	LI $4.81, LMI $5.52						
HC11	Basic emergency newborn and obstetric care	36.3 (12.5)	27.3 (0.1)	8.5 (0.0)	2.2 (0.0)	0.0 (0.0)	14.9 (2.5)
	LI $69.14, LMI $145.10						

*ART* antiretroviral therapy, *IMAI* integrated management of adolescent and adult illness, *IMCI* integrated management of childhood illness, *PMTCT* prevention of mother-to-child-transmission, *STI* sexually transmitted infection, *TB* tuberculosis

## Discussion

In this research, we modeled and estimated risks of CHE (conditional on having a given disease or condition) for 29 health services across 13 disease categories corresponding to an essential benefits package delivered at the primary care level (as defined by DCP3 [20, 22]) in 34 low-income and lower-middle-income countries. This modeling exercise focused on low-income and lower-middle-income countries for three reasons, firstly, because these countries capture a rather homogeneous set of countries, regarding levels of development and who often face similar health system and financing issues; secondly, because they often have weaker social protection and health insurance programs (especially vis-à-vis richer countries like those in the Organisation for Economic Co-operation and Development and upper-middle-income countries), which makes their populations more vulnerable to OOP payments and catastrophic and impoverishing health expenditures; thirdly, because this group category of low-income and lower-middle-income countries often presents with consistent ready-to-use datasets (e.g., DHS and STEPs surveys, DCP3 inputs, NHA reports).

Our computations show that high CHE risks, especially for the poorest, could be faced when seeking basic primary care services. Differences in CHE risk between quintiles were expected, since OOP payment amounts inherently comprised a larger share of income for poorer individuals with fewer resources. Across all countries and health services examined, the poorest quintile had, on average, 5 times greater CHE risks (compared to the richest quintile; using a 10% threshold) (Table 1). Such fairness concerns would be reduced for diseases with either low-cost or highly subsidized services (e.g., childhood illness treatment, ART), with several services causing virtually (in our modeling exercise) no instances of CHE across all quintiles (e.g., antenatal tetanus immunization). High-cost services, on the other hand, would show greater disparities. When seeking TB diagnosis and treatment, for example, the poorest seemed to be at disproportionately greater CHE risk (compared to the richest).

The methodology implemented in this analysis could be replicated by analysts when designing publicly financed essential benefit packages in LMICs which aim to provide a defined set of services to the entire population at little to no cost [16, 27]. Cost-effectiveness is often the prime consideration when designing such benefit packages, although FRP is increasingly used given UHC goals [2, 26]. Here, we provide a first preliminary and simple attempt at modeling the FRP dimension and potential benefits that could ensue the rollout of specific essential services when included in benefit packages on the path to UHC in LMICs.

In our model, a risk of CHE (conditional on having disease) depends on three health system factors: usage (captured through health services utilization); affordability (captured via health service costs); and level of public financing (captured by the percent of costs paid for out of pocket). Methodically parsing out the relative impact of these three factors on CHE risk is beyond the scope of our modeling exercise. However, there are several components of this research that lend insights into this discussion, particularly as it relates to the usage factor.

In alignment with other studies on FRP and estimations of catastrophic and impoverishing health expenditures in LMICs [28–31], CHE risks (conditional on having disease) are, evidently, highly dependent on utilization for health services. Utilization is itself impacted by financial considerations; individuals who fear high OOP costs for receiving health services are less likely to seek those services in the first place, thereby limiting their exposure to OOP payments and eventual CHE risk. It is thus important to consider what might be the impact of including utilization—if all individuals in need of health services were to actually seek those services without consideration of financial consequences, CHE risks would likely be much higher.

Additionally, utilization of health services also depends on the supply of those health services. For example, many LMICs have limited provision of services for NCDs, as well as for some reproductive health services, like medical abortions [32–35]. The health services utilization input parameters used in our model intended to capture these supply-side limitations to care access; however, those parameters used were proxies and would not reflect the nuance and specific availability of individual services in select geographical locations (e.g., urban vs. rural setting). Therefore, these proxy utilization input parameters might over- or under-estimate the true utilization of some health services. For services with low utilization, considering the financial risks associated with providing those services may be premature since the supply-side constraints may be paramount. In alignment with the mission of UHC, individuals must be able to first obtain the quality health services they need before they can do so without financial hardship.

In summary, the extents of OOP payment amounts and potential ensuing medical impoverishment would depend on both the supply (e.g., are the services for specific diseases available?) and the demand (e.g., are the services for specific diseases accessible physically and financially by the populations impacted?) for health services [30].

Importantly, this study has several limitations. First, the model utilized data inputs and estimates from several heterogeneous sources (e.g., DHS and STEPs surveys, NHA reports, DCP3 unit cost inputs), with some input datasets eventually linked manually. Second, there were many instances of missing input data, particularly for health services utilization proxies, which were partially addressed by substituting missing values with regional averages, which limits the specificity of results for some countries (see Additional file 1: Table A.6). This limitation is particularly relevant for missing input data on the percent of disease expenditures paid for out of pocket, given that health system financing is particularly nuanced and country-specific: for example, in certain countries a larger number of services and disease areas are publicly financed (see Additional file 1: Table A.3 for detailed information). Third, there are inherent limitations due to the use of threshold-based metrics of (lack of) FRP—like the headcount of CHE [36, 37]—notably the fact that those metrics do not include the individuals who do not seek care due to access or financial barriers, as well as the arbitrariness of the retained thresholds of income (10, 25, and 40%, for example) [37–39]. Yet, these are commonly used metrics and we have conducted sensitivity analyses while using a variety of alternative thresholds. Fourth, our modeling approach was rudimentary by design, because of the great lack of data, and was based on the simple combination of health services utilization, OOP spending, and income. We made no attempt to include more sophisticated approaches, like the superimposition of utility functions (e.g., to estimate risk premiums) [40, 41]. In particular, following the routinely reported CHE metric, we only included direct medical costs and did not add direct non-medical costs (e.g., transportation to and from health facilities) and indirect costs (e.g., lost wages), which could be substantial (e.g., for chronic and long-lasting conditions). Likewise, the time period for the modeling analysis was 1 year only, while some diseases, like chronic NCDs for example, can occur over multiple years. We also did not model the crowding of OOP spending within households (i.e., households facing multiple disease-related OOP expenditures simultaneously, which thereby would increase CHE risks). Additionally, due to a lack of data, we did not fully prescribe household characteristics, such as composition (e.g., number of children, adults, and elderly), average age of members, education levels, and geographic locations. Likewise, we were not able to stratify health services utilization for specific conditions by provider type (e.g., public vs. private) and setting (e.g., rural vs. urban). We also used simulated incomes (with per capita GNI and Gini as proxies) for the computation of CHE (in place of consumption expenditures) because consumption expenditures input data were not available for all country-years examined here. Yet, this choice would likely not affect our modeling strategy nor impact on our relative general findings across the different disease areas. Fifth, beyond input data constraints, the model also made several assumptions. Input data on costs were available at the health service level, while input data on OOP expenditures were available at the disease level (as obtainable from NHA reports). Our model applied national-level disease-specific OOP spending percentages to individual-level payments for services targeting that disease, which might not be a reasonable assumption for all disease areas or health services considered. Relatedly, the model did not account for health insurance coverage, nor for differential provider types (e.g., public vs. private). Disease-specific OOP spending percentages reflected a population-level aggregate which was applied to all individuals equally (i.e., all quintiles were assumed to pay the same OOP percentage). However, some countries might have insurance schemes that could reduce the cost of services for the poor. In those instances, the quintile gradient of CHE risk would therefore likely shift to middle or richer quintiles, even though the poor typically spend less in absolute terms because of their lower capacity to pay.

## Conclusions

In conclusion, we present a preliminary model for estimating the risk of CHE associated with utilizing primary care services, by service and disease, following the DCP3 highest priority package typology [21]. Our results indicate that financial threats could be severe, especially for the poorest. The CHE risks would likely increase if more health services were available and if there were no financial barriers to access. Toward achieving UHC, much work remains to be done. Our preliminary methods could be replicated by analysts aiming to design essential benefit packages: we provide here a stepping stone toward explicit quantitative inclusion of FRP considerations that could ensue inclusion of services in benefit packages in LMICs.

### Supplementary Information

**Additional file 1:** Webappendix.pdf [42–46].

## Data Availability

Upon request, the input data and analytical codes described in the manuscript will be made available to any scientist wishing to use them for non-commercial purposes.

## Abbreviations

ART: Antiretroviral therapy
CHE: Catastrophic health expenditures
CVD: Cardiovascular diseases
DCP3: Disease Control Priorities, 3rd edition
DHS: Demographic and Health Surveys
FRP: Financial risk protection
GHED: Global Health Expenditure Database
GNI: Gross national income
HEFPI: Health Equity and Financial Protection Indicators
IHE: Impoverishing health expenditures
IMAI: Integrated management of adult infections
IMCI: Integrated management of childhood infections
LI: Low-income
LMI: Lower-middle-income
LMICs: Low-middle-income countries
NCDs: Noncommunicable diseases
NHA: National health account
OOP: Out-of-pocket
PMTCT: Prevention of mother-to-child transmission
PPP: Purchasing power parity
SDG: Sustainable Development Goal
STEPS: STEPwise Approach to Surveillance
STIs: Sexually transmitted infections
TB: Tuberculosis
UHC: Universal health coverage
WDI: World Development Indicators
WHO: World Health Organization

## Footnotes

1 We refer to the “contraceptive management (family planning)” disease area simply as “family planning” in this analysis.

2 Note that this is a crude approximation and additional characteristics, such as household composition (e.g., number of children, adults, and elderly), average age of household members, education levels, and geographical locations, were not taken into account.

3 We chose 2016 USD as our analysis currency primarily for simplicity; DCP3 health service unit costs were reported in 2016 USD. 2016 USD also suited the NHA data, given that roughly a quarter of the NHA reports were from 2016 (another quarter of reports were from 2015; see Table 1).
