# Supplementary material for: Financial hardship associated with catastrophic out-of-pocket spending tied to primary care services in low- and lower-middle-income countries: findings from a modeling study
Source: BMC Med. 2023 Sep 14;21:356. doi: 10.1186/s12916-023-02957-w (PMC10503078; doi:10.1186/s12916-023-02957-w)
Supplement: Supplementary file 1 — Additional file 1: Webappendix.pdf [42–46]. [file 12916_2023_2957_MOESM1_ESM.pdf]

Additional file

Financial hardship associated with catastrophic out-of-pocket spending tied to primary care services in low- and lower middle-income countries: findings from a modeling study

Sarah Bolongaita[1,2], Yeeun Lee[1], Kjell Arne Johansson[2], Øystein A. Haaland[2], Mieraf Taddesse Tolla[3,4], Jongwook Lee[1,5], Stéphane Verguet[1]

[1] Department of Global Health and Population, Harvard T.H. Chan School of Public Health, Boston, MA, USA

[2] Department of Global Public Health and Primary Care, University of Bergen, Bergen, Norway

[3] Addis Center for Ethics and Priority Setting, Addis Ababa University, Addis Ababa, Ethiopia

[4] Africa Centers for Disease Control and Prevention Addis Ababa, Ethiopia

[5] Department of Agricultural Economics and Rural Development, Seoul National University, Seoul, South Korea

Table of Contents

A. Detailed methodology 2

1. Input data organization and classification 2
   - Classification of countries by region and income group 2
   - Classification of diseases by broad disease area 3
2. Data sources and estimates 4
   - Disease-specific OOP spending 4
   - Health service unit costs 13
   - Health services utilization 14
   - Simulated income distributions (proxied with GNI per capita, Gini coefficient) 32
   - Linkage between diseases, health services, and utilization indicators 33

B. Full model results 36

A. Detailed methodology

330 National Health Accounts (NHA) reports from 99 countries were obtained from the World Health Organization's (WHO) Global Health Expenditure Database (https://apps.who.int/nha/database/Home/Index/en) and reviewed for disease-specific out-of-pocket (OOP) information in October 2020.[20] Disease-specific OOP information was available in NHA reports from 45 countries (24 low-income countries, 14 lower middle-income countries, four upper middle-income countries, and three high-income countries). By country, the most recent NHA report containing disease-specific OOP information was used to extract disease-specific OOP expenditure (expressed as a percentage of total disease-specific expenditure). Countries were included in this analysis if they were classified as low- or middle-income during the year in which their most recent NHA report containing disease-specific OOP information was published.

1. Input data organization and classification

<u>Classification of countries by region and income group</u>

Table A.1. Analysis countries with regional and income group classifications. Regions are based on the UN Statistics Division classifications: regions reflect geographic continents and are subdivided into sub-regions, which are then subdivided into intermediary regions (https://unstats.un.org/unsd/methodology/m49/#geo-regions).[46] Income groups are classified according to the World Bank's 2022 classification scheme (https://datahelpdesk.worldbank.org/knowledgebase/articles/906519-world-bank-country-and-lending-groups).[47] Classification was based on countries' gross national income (GNI) per capita for the year of the country's national health account (NHA) data.

Country	Intermediate region	Sub-region	Region	Income group
Afghanistan	-	Southern Asia	Asia	Low income
Armenia	-	Western Asia	Asia	Lower middle income
Benin	Western Africa	Sub-Saharan Africa	Africa	Lower middle income
Burkina Faso	Western Africa	Sub-Saharan Africa	Africa	Low income
Burundi	Eastern Africa	Sub-Saharan Africa	Africa	Low income
Cabo Verde	Western Africa	Sub-Saharan Africa	Africa	Lower middle income
Cambodia	-	South-eastern Asia	Asia	Low income
Cameroon	Middle Africa	Sub-Saharan Africa	Africa	Lower middle income
Congo, Democratic Republic of the	Middle Africa	Sub-Saharan Africa	Africa	Low income
Congo, Republic of the	Middle Africa	Sub-Saharan Africa	Africa	Lower middle income
Côte d'Ivoire	Western Africa	Sub-Saharan Africa	Africa	Lower middle income
Ethiopia	Eastern Africa	Sub-Saharan Africa	Africa	Low income
Gambia, Republic of the	Western Africa	Sub-Saharan Africa	Africa	Low income
Ghana	Western Africa	Sub-Saharan Africa	Africa	Lower middle income
Guinea	Western Africa	Sub-Saharan Africa	Africa	Low income
Haiti	Caribbean	Latin America & the Caribbean	Americas	Lower middle income
Kenya	Eastern Africa	Sub-Saharan Africa	Africa	Lower middle income
Lao PDR	-	South-eastern Asia	Asia	Lower middle income
Malawi	Eastern Africa	Sub-Saharan Africa	Africa	Low income
Mali	Western Africa	Sub-Saharan Africa	Africa	Low income
Mozambique	Eastern Africa	Sub-Saharan Africa	Africa	Low income
Myanmar	-	South-eastern Asia	Asia	Lower middle income
Nepal	-	Southern Asia	Asia	Low income
Niger	Western Africa	Sub-Saharan Africa	Africa	Low income
Nigeria	Western Africa	Sub-Saharan Africa	Africa	Lower middle income
Samoa	-	Polynesia	Oceania	Lower middle income
São Tomé & Príncipe	Middle Africa	Sub-Saharan Africa	Africa	Lower middle income
Senegal	Western Africa	Sub-Saharan Africa	Africa	Lower middle income
Sierra Leone	Western Africa	Sub-Saharan Africa	Africa	Low income
Tajikistan	-	Central Asia	Asia	Lower middle income

Tanzania	Eastern Africa	Sub-Saharan Africa	Africa	Low income
Uganda	Eastern Africa	Sub-Saharan Africa	Africa	Low income
Viet Nam	-	South-eastern Asia	Asia	Lower middle income
Zimbabwe	Eastern Africa	Sub-Saharan Africa	Africa	Low income

Classification of diseases by broad disease area

The disease categories included in this analysis are based on the categories for which countries reported OOP information in their NHAs. Countries that provided disease-specific OOP information generally did so according to WHO disease categories (see the WHO's "Methodology for the Update of the Global Heath Expenditure Database, 2000-2018: Technical Note" for detailed information), although not all countries reported OOP spending for all disease categories.[48] For the purposes of this analysis, we aggregated disease categories into broad disease areas, listed in Table A.2.

Table A.2. Selected National Health Account (NHA) disease categories for which disaggregated disease-specific out-of-pocket (OOP) health spending was available. Broad disease areas are indicated in yellow-banded rows; diseases are indicated below the respective disease category.

**Disease**
Childhood health
Childhood health
Infectious & parasitic diseases
Diarrheal diseases
HIV/AIDS & other sexually transmitted diseases
Malaria
Tuberculosis
Other infectious & parasitic diseases
Noncommunicable diseases (NCDs)
Cardiovascular diseases
Endocrine & metabolic disorders
Mental/behavioral disorders & neurological conditions
Other NCDs
Reproductive health
Contraceptive management (family planning)*
Maternal conditions
Perinatal conditions

* We refer to the "contraceptive management (family planning)" disease area simply as "family planning" in this analysis.

2. Data sources and estimates

Disease-specific OOP spending

Table A.3. Source of country estimate for percent of disease expenditures paid for out-of-pocket (OOP). For each disease, if the percent of disease expenditures paid for OOP was available from the country's National Health Account (NHA) data, it was used as the estimate. If unavailable, a regional average was used with the following decreasing priority according to data availability: intermediate region, sub-region, region, income group. Regions were defined and classified according to the Statistics Division of the United Nations and income groups were classified according to the World Bank's 2022 classifications (see Table A.1). NHA data was sourced from the WHO Global Health Expenditure database.

	OOP	
**Disease**	Estimate	Source
Afghanistan		
Childhood health	39.3%	IGA: Low income
Diarrheal diseases	85.8%	CE (2014)
HIV/AIDS & other sexually transmitted diseases	13.9%	RA: Asia
Malaria	33.2%	CE (2014)
Tuberculosis	12.6%	CE (2014)
Other infectious & parasitic diseases	70.0%	CE (2014)
Cardiovascular diseases	78.4%	RA: Asia
Endocrine & metabolic disorders	71.9%	RA: Asia
Mental/behavioral disorders & neurological conditions	66.0%	RA: Asia
Other NCDs	83.4%	RA: Asia
Family planning	83.2%	CE (2014)
Maternal conditions	75.2%	CE (2014)
Perinatal conditions	84.1%	CE (2014)
Armenia		
Childhood health	45.3%	IGA: Lower middle income
Diarrheal diseases	45.6%	CE (2016)
HIV/AIDS & other sexually transmitted diseases	32.6%	CE (2016)
Malaria	15.0%	RA: Asia
Tuberculosis	12.0%	CE (2016)
Other infectious & parasitic diseases	31.0%	CE (2016)
Cardiovascular diseases	72.4%	CE (2016)
Endocrine & metabolic disorders	52.4%	CE (2016)
Mental/behavioral disorders & neurological conditions	42.5%	CE (2016)
Other NCDs	87.8%	CE (2016)
Family planning	67.6%	RA: Asia
Maternal conditions	28.0%	CE (2016)
Perinatal conditions	0.0%	CE (2016)
Benin		
Childhood health	42.3%	SRA: Sub-Saharan Africa
Diarrheal diseases	65.9%	IRA: Western Africa
HIV/AIDS & other sexually transmitted diseases	4.8%	CE (2013)
Malaria	42.6%	CE (2013)
Tuberculosis	0.3%	CE (2013)
Other infectious & parasitic diseases	48.4%	IRA: Western Africa
Cardiovascular diseases	59.8%	IRA: Western Africa
Endocrine & metabolic disorders	63.7%	IRA: Western Africa
Mental/behavioral disorders & neurological conditions	52.2%	IRA: Western Africa
Other NCDs	37.2%	IRA: Western Africa
Family planning	22.9%	IRA: Western Africa
Maternal conditions	52.6%	IRA: Western Africa
Perinatal conditions	53.1%	IRA: Western Africa

Disease	OOP	
	Estimate	Source
Burkina Faso		
Childhood health	42.3%	SRA: Sub-Saharan Africa
Diarrheal diseases	65.9%	IRA: Western Africa
HIV/AIDS & other sexually transmitted diseases	2.7%	CE (2016)
Malaria	26.7%	CE (2016)
Tuberculosis	40.3%	IRA: Western Africa
Other infectious & parasitic diseases	48.4%	IRA: Western Africa
Cardiovascular diseases	59.8%	IRA: Western Africa
Endocrine & metabolic disorders	63.7%	IRA: Western Africa
Mental/behavioral disorders & neurological conditions	52.2%	IRA: Western Africa
Other NCDs	37.2%	IRA: Western Africa
Family planning	3.3%	CE (2016)
Maternal conditions	52.6%	IRA: Western Africa
Perinatal conditions	53.1%	IRA: Western Africa
Burundi		
Childhood health	39.3%	IRA: Eastern Africa
Diarrheal diseases	48.3%	CE (2013)
HIV/AIDS & other sexually transmitted diseases	0.2%	CE (2013)
Malaria	35.9%	CE (2013)
Tuberculosis	7.2%	CE (2013)
Other infectious & parasitic diseases	22.6%	CE (2013)
Cardiovascular diseases	56.5%	IRA: Eastern Africa
Endocrine & metabolic disorders	53.9%	IRA: Eastern Africa
Mental/behavioral disorders & neurological conditions	50.5%	IRA: Eastern Africa
Other NCDs	38.0%	CE (2013)
Family planning	0.3%	IRA: Eastern Africa
Maternal conditions	10.2%	CE (2013)
Perinatal conditions	38.6%	IRA: Eastern Africa
Cabo Verde		
Childhood health	42.3%	SRA: Sub-Saharan Africa
Diarrheal diseases	53.4%	CE (2016)
HIV/AIDS & other sexually transmitted diseases	22.9%	IRA: Western Africa
Malaria	53.2%	IRA: Western Africa
Tuberculosis	40.3%	IRA: Western Africa
Other infectious & parasitic diseases	49.8%	CE (2016)
Cardiovascular diseases	11.9%	CE (2016)
Endocrine & metabolic disorders	16.8%	CE (2016)
Mental/behavioral disorders & neurological conditions	5.5%	CE (2016)
Other NCDs	15.0%	CE (2016)
Family planning	22.9%	IRA: Western Africa
Maternal conditions	5.8%	CE (2016)
Perinatal conditions	1.2%	CE (2016)
Cambodia		
Childhood health	39.3%	IGA: Low income
Diarrheal diseases	73.8%	CE (2014)
HIV/AIDS & other sexually transmitted diseases	4.5%	SRA: South-eastern Asia
Malaria	5.1%	CE (2014)
Tuberculosis	30.1%	SRA: South-eastern Asia
Other infectious & parasitic diseases	44.7%	CE (2014)
Cardiovascular diseases	82.2%	CE (2014)
Endocrine & metabolic disorders	79.2%	CE (2014)
Mental/behavioral disorders & neurological conditions	83.7%	CE (2014)
Other NCDs	81.2%	SRA: South-eastern Asia
Family planning	71.8%	CE (2014)

Disease	OOP	
	Estimate	Source
Maternal conditions	83.0%	CE (2014)
Perinatal conditions	55.2%	SRA: South-eastern Asia
Cameroon		
Childhood health	44.5%	CE (2011)
Diarrheal diseases	37.1%	IRA: Middle Africa
HIV/AIDS & other sexually transmitted diseases	29.0%	CE (2011)
Malaria	48.1%	CE (2011)
Tuberculosis	41.5%	CE (2011)
Other infectious & parasitic diseases	26.9%	IRA: Middle Africa
Cardiovascular diseases	8.7%	IRA: Middle Africa
Endocrine & metabolic disorders	8.9%	IRA: Middle Africa
Mental/behavioral disorders & neurological conditions	2.3%	IRA: Middle Africa
Other NCDs	44.4%	IRA: Middle Africa
Family planning	1.2%	IRA: Middle Africa
Maternal conditions	76.4%	IRA: Middle Africa
Perinatal conditions	49.4%	SRA: Sub-Saharan Africa
Congo, Democratic Republic of the		
Childhood health	45.3%	IRA: Middle Africa
Diarrheal diseases	74.1%	CE (2018)
HIV/AIDS & other sexually transmitted diseases	1.7%	CE (2018)
Malaria	53.3%	CE (2018)
Tuberculosis	8.3%	CE (2018)
Other infectious & parasitic diseases	42.0%	CE (2018)
Cardiovascular diseases	8.7%	IRA: Middle Africa
Endocrine & metabolic disorders	8.9%	IRA: Middle Africa
Mental/behavioral disorders & neurological conditions	2.3%	IRA: Middle Africa
Other NCDs	44.4%	IRA: Middle Africa
Family planning	1.2%	CE (2018)
Maternal conditions	76.4%	CE (2018)
Perinatal conditions	49.4%	SRA: Sub-Saharan Africa
Congo, Republic of the		
Childhood health	46.1%	CE (2015)
Diarrheal diseases	28.4%	CE (2015)
HIV/AIDS & other sexually transmitted diseases	1.7%	CE (2015)
Malaria	31.5%	CE (2015)
Tuberculosis	3.4%	CE (2015)
Other infectious & parasitic diseases	26.9%	IRA: Middle Africa
Cardiovascular diseases	8.7%	IRA: Middle Africa
Endocrine & metabolic disorders	8.9%	IRA: Middle Africa
Mental/behavioral disorders & neurological conditions	2.3%	IRA: Middle Africa
Other NCDs	44.4%	IRA: Middle Africa
Family planning	1.2%	IRA: Middle Africa
Maternal conditions	76.4%	IRA: Middle Africa
Perinatal conditions	49.4%	SRA: Sub-Saharan Africa
Côte d’Ivoire		
Childhood health	42.3%	SRA: Sub-Saharan Africa
Diarrheal diseases	91.7%	CE (2014)
HIV/AIDS & other sexually transmitted diseases	3.5%	CE (2014)
Malaria	75.8%	CE (2014)
Tuberculosis	6.6%	CE (2014)
Other infectious & parasitic diseases	59.8%	CE (2014)
Cardiovascular diseases	71.3%	CE (2014)
Endocrine & metabolic disorders	98.5%	CE (2014)
Mental/behavioral disorders & neurological conditions	52.2%	IRA: Western Africa

Disease	OOP	
	Estimate	Source
Other NCDs	37.2%	IRA: Western Africa
Family planning	0.1%	CE (2014)
Maternal conditions	85.8%	CE (2014)
Perinatal conditions	67.0%	CE (2014)
Ethiopia		
Childhood health	51.5%	CE (2011)
Diarrheal diseases	42.4%	IRA: Eastern Africa
HIV/AIDS & other sexually transmitted diseases	2.2%	CE (2011)
Malaria	14.6%	CE (2011)
Tuberculosis	37.5%	CE (2011)
Other infectious & parasitic diseases	19.8%	IRA: Eastern Africa
Cardiovascular diseases	56.5%	IRA: Eastern Africa
Endocrine & metabolic disorders	53.9%	IRA: Eastern Africa
Mental/behavioral disorders & neurological conditions	50.5%	IRA: Eastern Africa
Other NCDs	57.7%	IRA: Eastern Africa
Family planning	0.3%	IRA: Eastern Africa
Maternal conditions	29.9%	IRA: Eastern Africa
Perinatal conditions	38.6%	IRA: Eastern Africa
Gambia, Republic of the		
Childhood health	42.3%	SRA: Sub-Saharan Africa
Diarrheal diseases	65.9%	IRA: Western Africa
HIV/AIDS & other sexually transmitted diseases	22.9%	IRA: Western Africa
Malaria	53.2%	IRA: Western Africa
Tuberculosis	40.3%	IRA: Western Africa
Other infectious & parasitic diseases	11.8%	CE (2015)
Cardiovascular diseases	59.8%	IRA: Western Africa
Endocrine & metabolic disorders	63.7%	IRA: Western Africa
Mental/behavioral disorders & neurological conditions	52.2%	IRA: Western Africa
Other NCDs	37.2%	IRA: Western Africa
Family planning	22.9%	IRA: Western Africa
Maternal conditions	52.6%	IRA: Western Africa
Perinatal conditions	53.1%	IRA: Western Africa
Ghana		
Childhood health	42.3%	SRA: Sub-Saharan Africa
Diarrheal diseases	50.3%	CE (2015)
HIV/AIDS & other sexually transmitted diseases	0.9%	CE (2015)
Malaria	30.4%	CE (2015)
Tuberculosis	0.6%	CE (2015)
Other infectious & parasitic diseases	25.6%	CE (2015)
Cardiovascular diseases	70.1%	CE (2015)
Endocrine & metabolic disorders	46.3%	CE (2015)
Mental/behavioral disorders & neurological conditions	39.0%	CE (2015)
Other NCDs	70.3%	CE (2015)
Family planning	22.9%	IRA: Western Africa
Maternal conditions	68.9%	CE (2015)
Perinatal conditions	77.3%	CE (2015)
Guinea		
Childhood health	42.3%	SRA: Sub-Saharan Africa
Diarrheal diseases	74.8%	CE (2014)
HIV/AIDS & other sexually transmitted diseases	58.7%	CE (2014)
Malaria	51.3%	CE (2014)
Tuberculosis	49.8%	CE (2014)
Other infectious & parasitic diseases	52.5%	CE (2014)
Cardiovascular diseases	59.8%	IRA: Western Africa

Disease	OOP	
	Estimate	Source
Endocrine & metabolic disorders	63.7%	IRA: Western Africa
Mental/behavioral disorders & neurological conditions	52.2%	IRA: Western Africa
Other NCDs	37.2%	IRA: Western Africa
Family planning	22.9%	IRA: Western Africa
Maternal conditions	52.6%	IRA: Western Africa
Perinatal conditions	53.1%	IRA: Western Africa
Haiti		
Childhood health	45.3%	IGA: Lower middle income
Diarrheal diseases	73.2%	CE (2014)
HIV/AIDS & other sexually transmitted diseases	1.9%	IRA: Caribbean
Malaria	21.2%	CE (2014)
Tuberculosis	1.2%	SRA: Latin America & the Caribbean
Other infectious & parasitic diseases	17.2%	CE (2014)
Cardiovascular diseases	73.2%	CE (2014)
Endocrine & metabolic disorders	72.9%	CE (2014)
Mental/behavioral disorders & neurological conditions	0.4%	IRA: Caribbean
Other NCDs	51.1%	CE (2014)
Family planning	92.0%	IRA: Caribbean
Maternal conditions	1.6%	IRA: Caribbean
Perinatal conditions	45.9%	IGA: Lower middle income
Kenya		
Childhood health	39.3%	IRA: Eastern Africa
Diarrheal diseases	30.6%	CE (2016)
HIV/AIDS & other sexually transmitted diseases	9.0%	CE (2016)
Malaria	21.5%	CE (2016)
Tuberculosis	3.9%	CE (2016)
Other infectious & parasitic diseases	16.7%	CE (2016)
Cardiovascular diseases	56.5%	IRA: Eastern Africa
Endocrine & metabolic disorders	53.9%	IRA: Eastern Africa
Mental/behavioral disorders & neurological conditions	50.5%	IRA: Eastern Africa
Other NCDs	57.7%	IRA: Eastern Africa
Family planning	0.3%	IRA: Eastern Africa
Maternal conditions	29.9%	IRA: Eastern Africa
Perinatal conditions	38.6%	IRA: Eastern Africa
Lao People’s Democratic Republic		
Childhood health	45.3%	IGA: Lower middle income
Diarrheal diseases	59.2%	CE (2012)
HIV/AIDS & other sexually transmitted diseases	4.5%	SRA: South-eastern Asia
Malaria	5.9%	SRA: South-eastern Asia
Tuberculosis	30.1%	SRA: South-eastern Asia
Other infectious & parasitic diseases	32.7%	CE (2012)
Cardiovascular diseases	68.8%	CE (2012)
Endocrine & metabolic disorders	64.3%	CE (2012)
Mental/behavioral disorders & neurological conditions	66.8%	CE (2012)
Other NCDs	97.6%	CE (2012)
Family planning	48.0%	CE (2012)
Maternal conditions	52.3%	CE (2012)
Perinatal conditions	39.6%	CE (2012)
Malawi		
Childhood health	39.3%	IRA: Eastern Africa
Diarrheal diseases	6.9%	CE (2015)
HIV/AIDS & other sexually transmitted diseases	9.7%	CE (2015)
Malaria	12.9%	CE (2015)
Tuberculosis	14.7%	CE (2015)

Disease	OOP	
	Estimate	Source
Other infectious & parasitic diseases	10.3%	CE (2015)
Cardiovascular diseases	56.5%	IRA: Eastern Africa
Endocrine & metabolic disorders	53.9%	IRA: Eastern Africa
Mental/behavioral disorders & neurological conditions	50.5%	IRA: Eastern Africa
Other NCDs	14.8%	CE (2015)
Family planning	0.3%	IRA: Eastern Africa
Maternal conditions	29.9%	IRA: Eastern Africa
Perinatal conditions	38.6%	IRA: Eastern Africa
Mali		
Childhood health	42.3%	SRA: Sub-Saharan Africa
Diarrheal diseases	49.2%	CE (2014)
HIV/AIDS & other sexually transmitted diseases	22.9%	IRA: Western Africa
Malaria	59.5%	CE (2014)
Tuberculosis	40.3%	IRA: Western Africa
Other infectious & parasitic diseases	51.5%	CE (2014)
Cardiovascular diseases	76.3%	CE (2014)
Endocrine & metabolic disorders	63.7%	IRA: Western Africa
Mental/behavioral disorders & neurological conditions	79.1%	CE (2014)
Other NCDs	16.5%	CE (2014)
Family planning	22.9%	IRA: Western Africa
Maternal conditions	37.5%	CE (2014)
Perinatal conditions	53.1%	IRA: Western Africa
Mozambique		
Childhood health	39.3%	IRA: Eastern Africa
Diarrheal diseases	42.4%	IRA: Eastern Africa
HIV/AIDS & other sexually transmitted diseases	8.5%	IRA: Eastern Africa
Malaria	3.5%	CE (2015)
Tuberculosis	12.7%	IRA: Eastern Africa
Other infectious & parasitic diseases	1.3%	CE (2015)
Cardiovascular diseases	100.0%	CE (2015)
Endocrine & metabolic disorders	100.0%	CE (2015)
Mental/behavioral disorders & neurological conditions	79.6%	CE (2015)
Other NCDs	89.6%	CE (2015)
Family planning	0.6%	CE (2015)
Maternal conditions	0.1%	CE (2015)
Perinatal conditions	38.6%	IRA: Eastern Africa
Myanmar		
Childhood health	45.3%	IGA: Lower middle income
Diarrheal diseases	85.0%	CE (2018)
HIV/AIDS & other sexually transmitted diseases	4.2%	CE (2018)
Malaria	6.7%	CE (2018)
Tuberculosis	30.1%	CE (2018)
Other infectious & parasitic diseases	61.9%	CE (2018)
Cardiovascular diseases	90.4%	CE (2018)
Endocrine & metabolic disorders	91.7%	CE (2018)
Mental/behavioral disorders & neurological conditions	71.2%	CE (2018)
Other NCDs	64.8%	CE (2018)
Family planning	59.9%	SRA: South-eastern Asia
Maternal conditions	90.7%	CE (2018)
Perinatal conditions	70.8%	CE (2018)
Nepal		
Childhood health	39.3%	IGA: Low income
Diarrheal diseases	85.8%	SRA: Southern Asia
HIV/AIDS & other sexually transmitted diseases	13.9%	RA: Asia

	OOP	
**Disease**	Estimate	Source
Malaria	33.2%	SRA: Southern Asia
Tuberculosis	12.6%	SRA: Southern Asia
Other infectious & parasitic diseases	54.8%	CE (2016)
Cardiovascular diseases	78.4%	RA: Asia
Endocrine & metabolic disorders	71.9%	RA: Asia
Mental/behavioral disorders & neurological conditions	66.0%	RA: Asia
Other NCDs	83.4%	RA: Asia
Family planning	83.2%	SRA: Southern Asia
Maternal conditions	75.2%	SRA: Southern Asia
Perinatal conditions	84.1%	SRA: Southern Asia
Niger		
Childhood health	42.3%	SRA: Sub-Saharan Africa
Diarrheal diseases	76.2%	CE (2015)
HIV/AIDS & other sexually transmitted diseases	15.7%	CE (2015)
Malaria	59.2%	CE (2015)
Tuberculosis	40.3%	IRA: Western Africa
Other infectious & parasitic diseases	48.2%	CE (2015)
Cardiovascular diseases	64.7%	CE (2015)
Endocrine & metabolic disorders	96.3%	CE (2015)
Mental/behavioral disorders & neurological conditions	66.1%	CE (2015)
Other NCDs	37.2%	IRA: Western Africa
Family planning	22.9%	IRA: Western Africa
Maternal conditions	23.0%	CE (2015)
Perinatal conditions	30.9%	CE (2015)
Nigeria		
Childhood health	42.3%	SRA: Sub-Saharan Africa
Diarrheal diseases	79.4%	CE (2016)
HIV/AIDS & other sexually transmitted diseases	30.7%	CE (2016)
Malaria	84.2%	CE (2016)
Tuberculosis	95.4%	CE (2016)
Other infectious & parasitic diseases	74.9%	CE (2016)
Cardiovascular diseases	59.8%	IRA: Western Africa
Endocrine & metabolic disorders	63.7%	IRA: Western Africa
Mental/behavioral disorders & neurological conditions	52.2%	IRA: Western Africa
Other NCDs	37.2%	IRA: Western Africa
Family planning	30.3%	CE (2016)
Maternal conditions	69.4%	CE (2016)
Perinatal conditions	67.9%	CE (2016)
Samoa		
Childhood health	45.3%	IGA: Lower middle income
Diarrheal diseases	54.8%	IGA: Lower middle income
HIV/AIDS & other sexually transmitted diseases	13.4%	IGA: Lower middle income
Malaria	36.9%	IGA: Lower middle income
Tuberculosis	23.3%	CE (2015)
Other infectious & parasitic diseases	18.8%	CE (2015)
Cardiovascular diseases	12.7%	CE (2015)
Endocrine & metabolic disorders	5.9%	CE (2015)
Mental/behavioral disorders & neurological conditions	8.5%	CE (2015)
Other NCDs	11.4%	CE (2015)
Family planning	2.5%	CE (2015)
Maternal conditions	2.2%	CE (2015)
Perinatal conditions	14.7%	CE (2015)
São Tomé & Príncipe		
Childhood health	45.3%	IRA: Middle Africa

Disease	OOP	
	Estimate	Source
Diarrheal diseases	8.8%	CE (2013)
HIV/AIDS & other sexually transmitted diseases	1.6%	CE (2013)
Malaria	8.1%	CE (2013)
Tuberculosis	0.1%	CE (2013)
Other infectious & parasitic diseases	11.9%	CE (2013)
Cardiovascular diseases	8.7%	CE (2013)
Endocrine & metabolic disorders	8.9%	CE (2013)
Mental/behavioral disorders & neurological conditions	2.3%	CE (2013)
Other NCDs	44.4%	CE (2013)
Family planning	1.2%	IRA: Middle Africa
Maternal conditions	76.4%	IRA: Middle Africa
Perinatal conditions	49.4%	SRA: Sub-Saharan Africa
Senegal		
Childhood health	42.3%	SRA: Sub-Saharan Africa
Diarrheal diseases	52.4%	CE (2013)
HIV/AIDS & other sexually transmitted diseases	38.1%	CE (2013)
Malaria	36.1%	CE (2013)
Tuberculosis	56.2%	CE (2013)
Other infectious & parasitic diseases	46.3%	CE (2013)
Cardiovascular diseases	64.7%	CE (2013)
Endocrine & metabolic disorders	60.5%	CE (2013)
Mental/behavioral disorders & neurological conditions	71.3%	CE (2013)
Other NCDs	47.1%	CE (2013)
Family planning	9.7%	CE (2013)
Maternal conditions	67.5%	CE (2013)
Perinatal conditions	74.2%	CE (2013)
Sierra Leone		
Childhood health	42.3%	SRA: Sub-Saharan Africa
Diarrheal diseases	65.9%	IRA: Western Africa
HIV/AIDS & other sexually transmitted diseases	50.7%	CE (2013)
Malaria	66.0%	CE (2013)
Tuberculosis	73.1%	CE (2013)
Other infectious & parasitic diseases	63.6%	CE (2013)
Cardiovascular diseases	59.8%	IRA: Western Africa
Endocrine & metabolic disorders	63.7%	IRA: Western Africa
Mental/behavioral disorders & neurological conditions	52.2%	IRA: Western Africa
Other NCDs	37.2%	IRA: Western Africa
Family planning	70.9%	CE (2013)
Maternal conditions	62.6%	CE (2013)
Perinatal conditions	53.1%	IRA: Western Africa
Tajikistan		
Childhood health	45.3%	IGA: Lower middle income
Diarrheal diseases	69.9%	RA: Asia
HIV/AIDS & other sexually transmitted diseases	13.9%	RA: Asia
Malaria	15.0%	RA: Asia
Tuberculosis	18.2%	RA: Asia
Other infectious & parasitic diseases	28.1%	CE (2013)
Cardiovascular diseases	78.4%	RA: Asia
Endocrine & metabolic disorders	71.9%	RA: Asia
Mental/behavioral disorders & neurological conditions	66.0%	RA: Asia
Other NCDs	83.4%	RA: Asia
Family planning	67.6%	RA: Asia
Maternal conditions	65.8%	RA: Asia
Perinatal conditions	48.6%	RA: Asia

Disease	OOP	
	Estimate	Source
Tanzania		
Childhood health	27.1%	CE (2015)
Diarrheal diseases	42.4%	IRA: Eastern Africa
HIV/AIDS & other sexually transmitted diseases	12.4%	CE (2015)
Malaria	20.3%	CE (2015)
Tuberculosis	12.7%	IRA: Eastern Africa
Other infectious & parasitic diseases	19.8%	IRA: Eastern Africa
Cardiovascular diseases	56.5%	IRA: Eastern Africa
Endocrine & metabolic disorders	53.9%	IRA: Eastern Africa
Mental/behavioral disorders & neurological conditions	50.5%	IRA: Eastern Africa
Other NCDs	57.7%	IRA: Eastern Africa
Family planning	0.3%	IRA: Eastern Africa
Maternal conditions	29.9%	IRA: Eastern Africa
Perinatal conditions	38.6%	IRA: Eastern Africa
Uganda		
Childhood health	39.3%	IRA: Eastern Africa
Diarrheal diseases	54.3%	CE (2016)
HIV/AIDS & other sexually transmitted diseases	8.1%	CE (2016)
Malaria	70.6%	CE (2016)
Tuberculosis	0.0%	CE (2016)
Other infectious & parasitic diseases	33.5%	CE (2016)
Cardiovascular diseases	0.1%	CE (2016)
Endocrine & metabolic disorders	0.1%	CE (2016)
Mental/behavioral disorders & neurological conditions	45.1%	CE (2016)
Other NCDs	93.6%	CE (2016)
Family planning	0.0%	CE (2016)
Maternal conditions	60.0%	CE (2016)
Perinatal conditions	67.6%	CE (2016)
Viet Nam		
Childhood health	45.3%	IGA: Lower middle income
Diarrheal diseases	72.7%	SRA: South-eastern Asia
HIV/AIDS & other sexually transmitted diseases	4.7%	CE (2015)
Malaria	5.9%	SRA: South-eastern Asia
Tuberculosis	30.1%	SRA: South-eastern Asia
Other infectious & parasitic diseases	46.4%	SRA: South-eastern Asia
Cardiovascular diseases	80.5%	SRA: South-eastern Asia
Endocrine & metabolic disorders	78.4%	SRA: South-eastern Asia
Mental/behavioral disorders & neurological conditions	73.9%	SRA: South-eastern Asia
Other NCDs	81.2%	SRA: South-eastern Asia
Family planning	59.9%	SRA: South-eastern Asia
Maternal conditions	75.3%	SRA: South-eastern Asia
Perinatal conditions	55.2%	SRA: South-eastern Asia
Zimbabwe		
Childhood health	39.3%	IRA: Eastern Africa
Diarrheal diseases	42.4%	IRA: Eastern Africa
HIV/AIDS & other sexually transmitted diseases	23.0%	CE (2010)
Malaria	25.6%	IRA: Eastern Africa
Tuberculosis	12.7%	IRA: Eastern Africa
Other infectious & parasitic diseases	19.8%	IRA: Eastern Africa
Cardiovascular diseases	56.5%	IRA: Eastern Africa
Endocrine & metabolic disorders	53.9%	IRA: Eastern Africa
Mental/behavioral disorders & neurological conditions	50.5%	IRA: Eastern Africa
Other NCDs	57.7%	IRA: Eastern Africa
Family planning	0.3%	IRA: Eastern Africa

Disease	OOP	
	Estimate	Source
Maternal conditions	29.9%	IRA: Eastern Africa
Perinatal conditions	38.6%	IRA: Eastern Africa

CE country estimate, IGA income group average, IRA intermediate region average, RA regional average, SRA sub-region average

Health service unit costs

Table A.4. Health service unit costs by country income-group: low-income country (LI), lower middle-income country (LMI). Health service descriptions include short descriptions (on top and in bold), used for convenience in the main text, tables, and figures, as well as Disease Control Priorities, 3rd edition (DCP3) full descriptions (below short descriptions). Data are available from DCP3 (https://dcp-uw.shinyapps.io/dcp-cm/).

Health service		Unit cost	
Code	Name and description	LI	LMI
Childhood health			
HC42	**Acute pharyngitis treatment**<br>Treatment of acute pharyngitis in children to prevent rheumatic fever	$0.17	$0.24
Infectious & parasitic diseases			
C7	**Intermittent preventive treatment (pregnancy)**<br>In high malaria transmission settings, intermittent preventive treatment in pregnancy	$0.45	$1.02
HC8	**HIV & syphilis prevention of PMTCT**<br>Prevention of mother-to-child transmission (PMTCT) of HIV (Option B+) and syphilis	$176.35	$313.51
HC12	**Diagnosis & treatment of infections (IMCI)**<br>Detection and treatment of childhood infections with danger signs using integrated management of childhood illness (IMCI)	$4.79	$10.29
HC13	**ART & viral load monitoring**<br>Among all individuals who are known to be HIV positive, immediate antiretroviral therapy (ART) initiation with regular monitoring of viral load for adherence and development of resistance	$71.45	$121.57
HC17	**Syndromic management of STIs**<br>Syndromic management of common sexually transmitted infections (STIs) and reproductive tract infections (for example urethral discharge, genital ulcer, and others) according to WHO guidelines	$5.67	$10.69
HC23	**HIV, STIs, hepatitis testing & counseling**<br>Provider-initiated testing and counseling for HIV, sexually transmitted infections (STIs), and hepatitis, for all in contact with health system in high-prevalence settings, including prenatal care with appropriate referral or linkage to care including immediate antiretroviral therapy (ART) initiation for those testing positive for HIV	$4.31	$6.08
HC27	**Diagnosis & treatment of TB**<br>Diagnosis of tuberculosis (TB), including assessment of rifampicin resistance using rapid molecular diagnostics (UltraXpert), and initiation of first-line treatment per current WHO guidelines for drug-susceptible TB; referral for confirmation, further assessment of drug resistance, and treatment of drug-resistant TB	$135.09	$175.65
HC30	**Management & referrals for fever (IMAI)**<br>Evaluation and management of fever in clinically stable individuals using WHO integrated management of adolescent and adult illness (IMAI) guidelines, with referral of unstable individuals to first-level hospital care	$3.11	$6.83
Noncommunicable diseases (NCDs)			
HC38	**Aspirin for acute myocardial infarction**<br>Provision of aspirin for all cases of suspected acute myocardial infarction	$0.03	$0.05
HC40	**Screening & management of diabetes**<br>Screening and management of diabetes among at-risk adults, including glycemic control, management of blood pressure and lipids, and consistent foot care	$64.16	$92.52
HC43	**Management of ischemic heart disease**<br>Long term management of ischemic heart disease, stroke, and peripheral vascular disease with aspirin, beta blockers, ACE inhibitors, and statins (as indicated) to reduce risk of further events	$83.97	$190.17
HC44	**Management of heart failure**<br>Medical management of heart failure with diuretics, beta-blockers, ACE inhibitors, and mineralocorticoid antagonists	$249.96	$342.48

Health service		Unit cost	
Code	Name and description	LI	LMI
HC47	**Palliative care**<br>Essential palliative care and pain control measures, including oral immediate release morphine and medicines for associated symptoms	$64.63	$21.32
HC49	**Management of bipolar disorder**<br>Management of bipolar disorder using generic mood-stabilizing medications and psychosocial treatment	$184.57	$365.39
HC50	**Management of depression**<br>Management of depression and anxiety disorders with psychological and generic antidepressant therapy	$16.11	$48.02
HC51	**Management of epilepsy**<br>Management of epilepsy, including acute stabilization and long-term management with generic anti-epileptics	$27.53	$53.73
HC52	**Management of schizophrenia**<br>Management of schizophrenia using generic anti-psychotic medications and psychosocial treatment	$99.43	$329.34
HC66	**Psychosocial support & counseling**<br>Psychosocial support and counseling services for individuals with serious, complex, or life-limiting health problems and their caregivers	$64.63	$21.32
Reproductive health			
C5	**Antenatal tetanus immunization**<br>Tetanus toxoid immunization among women attending antenatal care	$0.39	$0.44
C13	**Cotrimoxazole for HIV-exposed children**<br>Provision of cotrimoxazole to children born to HIV-positive mothers	$6.55	$11.95
HC1	**Antibiotics for neonatal pneumonia**<br>Early detection and treatment of neonatal pneumonia with oral antibiotics	$6.22	$6.61
HC2	**Post-abortion care**<br>Management of miscarriage or incomplete abortion and post abortion care	$4.54	$8.23
HC3	**Treatment of premature membrane rupture**<br>Management of preterm premature rupture of membranes, including administration of antibiotics	$4.04	$4.66
HC4	**Contraceptives**<br>Provision of condoms and hormonal contraceptives, including emergency contraceptives	$4.97	$10.32
HC5	**Kangaroo mother care counseling**<br>Counseling of mothers on providing kangaroo care for newborns	$2.33	$4.61
HC6	**Neonatal sepsis, pneumonia, meningitis**<br>Management of neonatal sepsis, pneumonia, and meningitis using injectable and oral antibiotics	$2.66	$3.51
HC7	**Medical abortion**<br>Pharmacological termination of pregnancy	$4.81	$5.52
HC11	**Basic emergency newborn & obstetric care**<br>Management of labor and delivery in low-risk women (i.e., base emergency newborn and obstetric care [BEmNOC]), including initial treatment of obstetric or delivery complications prior to transfer	$69.14	$145.10

Health services utilization

Data for most indicators were available disaggregated by wealth quintile. However, if no empirical gradient between quintiles was available for an indicator by country or region (for example, for indicators derived from the literature), a country's average quintile gradient was used. The average wealth quintile gradient was calculated by first determining each quintile's utilization, relative to the total population's utilization, for each utilization indicator (i.e., the quintile-specific utilization rates were divided by the total population's utilization rate, to determine each quintile's utilization relative to the total population for each indicator). The quintile's relative utilization rates were then averaged across all indicators to produce the quintile's average utilization relative to the total population. Given a total population's average relative utilization of 1, quintile 1 (Q1) could, for example, have an average relative utilization of 0.2 and quintile 5 (Q5) could have an average relative utilization of 1.6. This would mean that households in Q1 utilize health services at a rate that is 20% of the total population's utilization rate, on average; likewise,

households in Q5 utilize health services at a rate that is, on average, 60% greater than the total population's utilization rate.

Table A.5. Healthcare utilization proxy indicators.

Code	Description	Source
CH_ARIS_C_ABI	Percent of children with symptoms of acute respiratory infection (ARI) who received antibiotics	DHS
CH_DIAT_C_ADV	Percent of children with diarrhea who were to a health facility	DHS
HA_CPHT_MW_EVT	Percent of population ever receiving an HIV test	DHS
HA_STIS_W_STI	Percent of women reporting a sexually transmitted infection (STI)	DHS
ML_FEVT_C_ADV	Percent of children with fever for whom advice or treatment was sought	DHS
RH_ANCP_W_SKP	Percent of women with a prior live birth who received antenatal care from a skilled provider	DHS
RH_DELA_C_SKP	Percent of women with a prior live birth who received assistance during delivery from a skilled provider	DHS
RH_PCMT_W_DY2	Percent of women giving birth who had their first postnatal checkup 1-2 days after birth	DHS
HF.DYN.CONM.ZS	Percent of women ages 15-49 years using modern contraceptive methods	HEFPI
HF.STA.ANV4.ZS	Percent of women with a prior live birth who received at least 4 antenatal care visits	HEFPI
KNAUL	Percent of individuals with health conditions most associated with serious health-related suffering whose healthcare needs are met	Knaul et al., 2018
LORA	Percent of individuals with schizophrenic disorders who receive treatment	Lora et al., 2012
MEYER	Percent of individuals with epilepsy who receive treatment	Meyer et al., 2010
STEPS_BL_GLUCOSE	Percent of population with raised blood glucose or currently on medication for raised blood glucose	STEPS
STEPS_BP_CHOLESTEROL	Percent of population with raised total cholesterol, currently on medication for raised cholesterol or raised blood pressure	STEPS
SH.HIV.PMTC.ZS	Percent of pregnant women with HIV who are on antiretroviral therapy (ART) for prevention of mother-to-child-transmission (PMTCT)	WDI
SH.TBS.DTEC.ZS	Tuberculosis (TB) case detection rate (all forms of TB)	WDI

DHS Demographic and Health Surveys, *HEFPI* Health Equity and Financial Protection Indicators (World Bank), *STEPS* STEPwise Approach to NCD Risk Factor Surveillance (WHO), *WDI* World Development Indicators (World Bank)

Table A.6. Model country estimate source for health service utilization proxy inputs. For each utilization input, if data for the country was available, it was used as the estimate. If unavailable, a regional average was used with the following decreasing priority according to data availability: intermediate region, sub-region, region, income group. Regions were defined and classified according to the Statistics Division of the United Nations and income groups were classified according to the World Bank. For quintile gradient estimates, if there was no empirical gradient available for the input by country or region (for example, for inputs from the literature), then the average country gradient was used.

Utilization input	Total population		Quintile gradient	
Code	Estimate (%)	Source	Estimates (Q1 to Q5, %)	Source
Afghanistan				
CH_ARIS_C_ABI	56.2	CE (2015)	54.7, 57.5, 54.2, 56.4, 59.2	CE (2015)
CH_DIAT_C_ADV	64.6	CE (2015)	63.3, 65.9, 60.4, 66.9, 66.3	CE (2015)
HA_CPHT_MW_EVT	2.8	CE (2015)	1.4, 1.6, 1.3, 2.5, 7.2	CE (2015)
HA_STIS_W_STI	2.2	CE (2015)	2.0, 2.1, 2.1, 2.7, 2.0	CE (2015)
HF.DYN.CONM.ZS	19.8	CE (2015)	15.1, 16.1, 15.7, 22.0, 30.5	CE (2015)
HF.STA.ANV4.ZS	19.6	CE (2015)	11.9, 12.0, 14.3, 20.5, 38.8	CE (2015)
KNAUL	0.2	CE	0.2, 0.2, 0.2, 0.2, 0.3	CAG
LORA	5.0	CE	3.9, 4.2, 4.2, 5.4, 7.3	CAG
MEYER	35.5	SRA: Southern Asia	28.0, 29.9, 30.0, 38.0, 51.6	CAG
ML_FEVT_C_ADV	63.5	CE (2015)	63.1, 63.7, 59.2, 62.9, 69.4	CE (2015)
RH_ANCP_W_SKP	59.1	CE (2015)	50.8, 51.0, 54.3, 64.1, 76.3	CE (2015)
RH_DELA_C_SKP	54.2	CE (2015)	26.9, 39.7, 47.1, 68.6, 88.0	CE (2015)
RH_PCMT_W_DY2	39.9	CE (2015)	31.4, 33.1, 35.9, 41.1, 57.7	CE (2015)
SH.HIV.PMTC.ZS	9.0	CE (2019)	7.1, 7.6, 7.6, 9.6, 13.1	CAG
SH.TBS.DTEC.ZS	73.0	CE (2019)	57.6, 61.5, 61.8, 78.2, 100.0	CAG
STEPS_BL_GLUCOSE	21.3	SRA: Southern Asia	16.8, 17.9, 18.0, 22.8, 31.0	CAG
STEPS_BP_CHOLESTEROL	11.1	SRA: Southern Asia	8.8, 9.4, 9.4, 11.9, 16.1	CAG
Armenia				
CH_ARIS_C_ABI	19.6	CE (2016)	15.4, 19.2, 21.7, 21.4, 23.4	SRA: Western Asia
CH_DIAT_C_ADV	39.9	CE (2016)	39.6, 37.5, 37.0, 46.2, 43.8	SRA: Western Asia
HA_CPHT_MW_EVT	8.2	CE (2016)	6.4, 7.6, 8.8, 8.2, 9.7	CE (2016)
HA_STIS_W_STI	0.8	CE (2016)	0.6, 0.9, 0.8, 0.8, 0.9	CE (2016)
HF.DYN.CONM.ZS	28.0	CE (2015)	21.2, 24.6, 25.1, 30.3, 37.2	CE (2015)
HF.STA.ANV4.ZS	97.9	CE (2015)	96.1, 95.1, 99.0, 99.6, 99.6	CE (2015)
KNAUL	7.3	RA: Asia	6.5, 7.1, 7.4, 7.5, 8.1	CAG
LORA	95.0	CE	85.2, 92.9, 95.9, 97.9, 100.0	CAG
MEYER	30.3	RA: Asia	27.2, 29.7, 30.6, 31.2, 33.8	CAG
ML_FEVT_C_ADV	68.4	SRA: Western Asia	65.0, 64.2, 73.6, 67.9, 80.5	SRA: Western Asia
RH_ANCP_W_SKP	99.7	CE (2016)	99.1, 100.0, 99.7, 99.6, 100.0	CE (2016)
RH_DELA_C_SKP	99.7	CE (2016)	99.5, 100.0, 100.0, 98.7, 100.0	CE (2016)

Utilization input	Total population		Quintile gradient	
Code	Estimate (%)	Source	Estimates (Q1 to Q5, %)	Source
RH_PCMT_W_DY2	97.4	CE (2016)	95.7, 98.7, 97.6, 94.5, 99.4	CE (2016)
SH.HIV.PMTC.ZS	57.5	SRA: Western Asia	51.6, 56.2, 58.0, 59.2, 64.1	CAG
SH.TBS.DTEC.ZS	80.0	CE (2019)	71.8, 78.2, 80.8, 82.4, 89.2	CAG
STEPS_BL_GLUCOSE	5.7	CE (2016)	5.1, 5.6, 5.8, 5.9, 6.4	CAG
STEPS_BP_CHOLESTEROL	23.5	CE (2016)	21.1, 23.0, 23.7, 24.2, 26.2	CAG
Benin				
CH_ARIS_C_ABI	20.4	CE (2017)	11.6, 18.5, 25.3, 22.3, 34.0	CE (2017)
CH_DIAT_C_ADV	43.1	CE (2017)	39.4, 44.4, 37.3, 43.5, 57.4	CE (2017)
HA_CPHT_MW_EVT	29.1	CE (2017)	15.6, 20.2, 26.0, 33.2, 46.6	CE (2017)
HA_STIS_W_STI	7.0	CE (2017)	7.5, 7.3, 5.4, 7.4, 7.3	CE (2017)
HF.DYN.CONM.ZS	14.4	CE (2014)	9.6, 10.8, 12.5, 16.6, 22.2	CE (2014)
HF.STA.ANV4.ZS	56.1	CE (2017)	34.7, 47.0, 54.6, 62.2, 81.8	CE (2017)
KNAUL	0.2	IRA: Western Africa	0.1, 0.2, 0.2, 0.2, 0.3	CAG
LORA	65.0	IRA: Western Africa	47.3, 59.1, 62.4, 72.3, 87.8	CAG
MEYER	27.6	IRA: Western Africa	20.1, 25.1, 26.5, 30.7, 37.3	CAG
ML_FEVT_C_ADV	53.1	CE (2017)	38.6, 49.2, 48.0, 64.2, 68.6	CE (2017)
RH_ANCP_W_SKP	83.2	CE (2017)	63.6, 80.4, 85.7, 92.5, 96.0	CE (2017)
RH_DELA_C_SKP	79.3	CE (2017)	57.4, 74.7, 81.1, 90.0, 96.2	CE (2017)
RH_PCMT_W_DY2	65.5	CE (2017)	44.9, 65.4, 67.8, 72.5, 79.5	CE (2017)
SH.HIV.PMTC.ZS	100.0	CE (2019)	72.7, 90.9, 96.0, 100.0, 100.0	CAG
SH.TBS.DTEC.ZS	65.0	CE (2019)	47.3, 59.1, 62.4, 72.3, 87.8	CAG
STEPS_BL_GLUCOSE	12.4	CE (2015)	9.0, 11.3, 11.9, 13.8, 16.8	CAG
STEPS_BP_CHOLESTEROL	4.4	CE (2015)	3.2, 4.0, 4.2, 4.9, 5.9	CAG
Burkina Faso				
CH_ARIS_C_ABI	32.6	IRA: Western Africa	24.4, 31.8, 28.1, 36.8, 47.9	IRA: Western Africa
CH_DIAT_C_ADV	59.6	IRA: Western Africa	55.6, 56.7, 59.8, 61.4, 68.9	IRA: Western Africa
HA_CPHT_MW_EVT	28.5	IRA: Western Africa	14.9, 19.0, 24.0, 31.6, 46.1	IRA: Western Africa
HA_STIS_W_STI	9.6	IRA: Western Africa	7.8, 8.7, 8.9, 11.1, 11.1	IRA: Western Africa
HF.DYN.CONM.ZS	15.0	CE (2010)	7.1, 8.6, 9.9, 16.3, 33.8	CE (2010)
HF.STA.ANV4.ZS	32.7	CE (2010)	22.4, 28.3, 32.7, 38.6, 45.0	CE (2010)
KNAUL	0.2	IRA: Western Africa	0.1, 0.2, 0.2, 0.2, 0.3	CAG
LORA	65.0	IRA: Western Africa	48.1, 56.1, 59.8, 71.7, 91.8	CAG
MEYER	27.6	IRA: Western Africa	20.4, 23.8, 25.4, 30.4, 39.0	CAG
ML_FEVT_C_ADV	73.5	CE (2017)	66.7, 76.9, 72.5, 71.1, 83.5	CE (2017)
RH_ANCP_W_SKP	80.7	CE (2017)	79.4, 81.0, 76.6, 77.9, 89.6	CE (2017)
RH_DELA_C_SKP	63.4	IRA: Western Africa	37.1, 48.9, 61.3, 80.5, 97.7	IRA: Western Africa
RH_PCMT_W_DY2	64.4	IRA: Western Africa	48.2, 57.2, 63.9, 73.7, 84.5	IRA: Western Africa
SH.HIV.PMTC.ZS	81.0	CE (2019)	59.9, 70.0, 74.5, 89.3, 100.0	CAG
SH.TBS.DTEC.ZS	51.0	CE (2019)	37.7, 44.1, 46.9, 56.2, 72.0	CAG

Utilization input	Total population		Quintile gradient	
Code	Estimate (%)	Source	Estimates (Q1 to Q5, %)	Source
STEPS_BL_GLUCOSE	10.7	IRA: Western Africa	7.9, 9.2, 9.8, 11.8, 15.1	CAG
STEPS_BP_CHOLESTEROL	24.5	IRA: Western Africa	18.1, 21.2, 22.5, 27.0, 34.6	CAG
Burundi				
CH_ARIS_C_ABI	26.2	CE (2016)	23.6, 25.4, 20.9, 29.6, 36.8	CE (2016)
CH_DIAT_C_ADV	60.3	CE (2016)	59.3, 55.1, 62.0, 62.5, 66.0	CE (2016)
HA_CPHT_MW_EVT	58.5	CE (2016)	57.1, 56.5, 58.1, 55.7, 63.5	CE (2016)
HA_STIS_W_STI	3.3	CE (2016)	2.2, 2.5, 3.1, 3.5, 5.3	CE (2016)
HF.DYN.CONM.ZS	17.7	CE (2010)	14.6, 13.9, 17.5, 17.4, 26.3	CE (2010)
HF.STA.ANV4.ZS	45.5	CE (2016)	42.6, 47.1, 43.8, 44.2, 50.6	CE (2016)
KNAUL	11.0	IRA: Eastern Africa	10.0, 10.3, 10.6, 11.1, 13.5	CAG
LORA	5.0	CE	4.5, 4.7, 4.8, 5.1, 6.1	CAG
MEYER	22.2	IRA: Eastern Africa	20.1, 20.8, 21.4, 22.4, 27.3	CAG
ML_FEVT_C_ADV	69.6	CE (2016)	68.8, 70.2, 68.3, 68.4, 74.0	CE (2016)
RH_ANCP_W_SKP	99.3	CE (2016)	98.4, 99.2, 99.7, 99.8, 99.4	CE (2016)
RH_DELA_C_SKP	86.4	CE (2016)	79.1, 85.2, 86.7, 87.3, 96.2	CE (2016)
RH_PCMT_W_DY2	51.1	CE (2016)	44.6, 48.4, 48.0, 50.4, 68.0	CE (2016)
SH.HIV.PMTC.ZS	69.0	CE (2019)	62.5, 64.6, 66.5, 69.8, 84.8	CAG
SH.TBS.DTEC.ZS	55.0	CE (2019)	49.8, 51.5, 53.0, 55.6, 67.6	CAG
STEPS_BL_GLUCOSE	5.2	IRA: Eastern Africa	4.7, 4.8, 5.0, 5.2, 6.3	CAG
STEPS_BP_CHOLESTEROL	15.3	IRA: Eastern Africa	13.9, 14.4, 14.8, 15.5, 18.8	CAG
Cabo Verde				
CH_ARIS_C_ABI	32.6	IRA: Western Africa	24.4, 31.8, 28.1, 36.8, 47.9	IRA: Western Africa
CH_DIAT_C_ADV	59.6	IRA: Western Africa	55.6, 56.7, 59.8, 61.4, 68.9	IRA: Western Africa
HA_CPHT_MW_EVT	28.5	IRA: Western Africa	14.9, 19.0, 24.0, 31.6, 46.1	IRA: Western Africa
HA_STIS_W_STI	9.6	IRA: Western Africa	7.8, 8.7, 8.9, 11.1, 11.1	IRA: Western Africa
HF.DYN.CONM.ZS	16.2	IRA: Western Africa	9.6, 11.9, 14.6, 20.0, 25.7	IRA: Western Africa
HF.STA.ANV4.ZS	59.3	IRA: Western Africa	43.4, 51.0, 58.1, 67.7, 81.4	IRA: Western Africa
KNAUL	0.2	IRA: Western Africa	0.1, 0.2, 0.2, 0.2, 0.3	CAG
LORA	65.0	IRA: Western Africa	48.1, 56.1, 61.5, 73.9, 88.1	CAG
MEYER	27.6	IRA: Western Africa	20.4, 23.8, 26.1, 31.4, 37.4	CAG
ML_FEVT_C_ADV	64.1	IRA: Western Africa	57.0, 61.5, 62.9, 68.2, 74.8	IRA: Western Africa
RH_ANCP_W_SKP	83.3	IRA: Western Africa	69.8, 77.1, 83.0, 92.0, 99.2	IRA: Western Africa
RH_DELA_C_SKP	63.4	IRA: Western Africa	37.1, 48.9, 61.3, 80.5, 97.7	IRA: Western Africa
RH_PCMT_W_DY2	64.4	IRA: Western Africa	48.2, 57.2, 63.9, 73.7, 84.5	IRA: Western Africa
SH.HIV.PMTC.ZS	65.0	IRA: Western Africa	48.1, 56.1, 61.5, 73.9, 88.1	CAG
SH.TBS.DTEC.ZS	80.0	CE (2019)	59.2, 69.0, 75.6, 91.0, 100.0	CAG
STEPS_BL_GLUCOSE	10.7	IRA: Western Africa	7.9, 9.2, 10.1, 12.2, 14.5	CAG
STEPS_BP_CHOLESTEROL	24.5	IRA: Western Africa	18.1, 21.1, 23.2, 27.9, 33.2	CAG

Utilization input	Total population		Quintile gradient	
Code	Estimate (%)	Source	Estimates (Q1 to Q5, %)	Source
Cambodia				
CH_ARIS_C_ABI	82.5	CE (2014)	87.9, 78.8, 87.8, 71.8, 85.1	CE (2014)
CH_DIAT_C_ADV	77.4	CE (2014)	80.9, 81.1, 77.0, 76.9, 69.4	CE (2014)
HA_CPHT_MW_EVT	39.2	CE (2014)	29.0, 32.0, 34.9, 41.7, 54.5	CE (2014)
HA_STIS_W_STI	5.6	CE (2014)	5.9, 6.2, 5.4, 5.8, 4.9	CE (2014)
HF.DYN.CONM.ZS	38.8	CE (2014)	39.5, 42.3, 38.4, 39.2, 34.7	CE (2014)
HF.STA.ANV4.ZS	76.2	CE (2014)	62.3, 68.1, 79.7, 86.9, 88.1	CE (2014)
KNAUL	9.0	SRA: South-eastern Asia	8.4, 8.8, 9.0, 9.3, 9.5	CAG
LORA	42.7	SRA: South-eastern Asia	39.9, 41.8, 42.6, 44.2, 45.2	CAG
MEYER	3.0	SRA: South-eastern Asia	2.8, 2.9, 3.0, 3.1, 3.2	CAG
ML_FEVT_C_ADV	59.6	SRA: South-eastern Asia	51.8, 55.6, 56.8, 67.9, 71.2	SRA: South-eastern Asia
RH_ANCP_W_SKP	96.0	CE (2014)	90.8, 95.9, 96.6, 98.6, 99.3	CE (2014)
RH_DELA_C_SKP	91.8	CE (2014)	79.0, 90.9, 95.3, 97.9, 98.7	CE (2014)
RH_PCMT_W_DY2	90.3	CE (2014)	84.3, 87.5, 93.6, 92.2, 95.6	CE (2014)
SH.HIV.PMTC.ZS	89.0	CE (2019)	83.2, 87.2, 88.8, 92.3, 94.4	CAG
SH.TBS.DTEC.ZS	63.0	CE (2019)	58.9, 61.7, 62.9, 65.3, 66.8	CAG
STEPS_BL_GLUCOSE	2.9	CE (2010)	2.7, 2.8, 2.9, 3.0, 3.1	CAG
STEPS_BP_CHOLESTEROL	20.7	CE (2010)	19.4, 20.3, 20.7, 21.5, 21.9	CAG
Cameroon				
CH_ARIS_C_ABI	33.2	CE (2018)	23.7, 29.8, 33.3, 40.5, 42.7	IRA: Middle Africa
CH_DIAT_C_ADV	52.3	CE (2018)	49.2, 50.1, 52.8, 59.6, 47.5	CE (2018)
HA_CPHT_MW_EVT	63.9	CE (2018)	34.8, 55.8, 63.8, 73.7, 80.0	CE (2018)
HA_STIS_W_STI	4.6	CE (2018)	2.5, 4.1, 4.4, 5.2, 5.9	CE (2018)
HF.DYN.CONM.ZS	24.3	CE (2014)	10.6, 18.8, 29.1, 30.5, 33.3	CE (2014)
HF.STA.ANV4.ZS	60.5	CE (2014)	37.6, 47.5, 63.1, 76.0, 91.5	CE (2014)
KNAUL	5.6	SRA: Sub-Saharan Africa	3.6, 5.0, 5.9, 6.7, 7.1	CAG
LORA	8.0	IRA: Middle Africa	5.1, 7.2, 8.4, 9.5, 10.2	CAG
MEYER	68.0	CE	43.2, 61.0, 71.6, 81.1, 86.5	CAG
ML_FEVT_C_ADV	61.0	CE (2018)	46.9, 61.1, 62.1, 69.4, 71.4	CE (2018)
RH_ANCP_W_SKP	86.5	CE (2018)	68.2, 83.2, 90.7, 96.9, 98.9	CE (2018)
RH_DELA_C_SKP	69.8	CE (2018)	30.7, 62.0, 80.0, 91.4, 98.1	CE (2018)
RH_PCMT_W_DY2	58.9	CE (2018)	32.3, 55.1, 64.9, 70.5, 81.9	CE (2018)
SH.HIV.PMTC.ZS	73.0	CE (2019)	46.3, 65.5, 76.8, 87.0, 92.9	CAG
SH.TBS.DTEC.ZS	53.0	CE (2019)	33.6, 47.5, 55.8, 63.2, 67.4	CAG
STEPS_BL_GLUCOSE	13.9	CE (2003)	8.8, 12.5, 14.6, 16.6, 17.7	CAG
STEPS_BP_CHOLESTEROL	17.3	CE (2003)	11.0, 15.5, 18.2, 20.6, 22.0	CAG
Congo, Democratic Republic of the				
CH_ARIS_C_ABI	42.5	IRA: Middle Africa	30.4, 38.2, 42.6, 51.8, 54.7	IRA: Middle Africa
CH_DIAT_C_ADV	51.3	IRA: Middle Africa	43.7, 47.2, 53.7, 56.5, 56.6	IRA: Middle Africa

Utilization input	Total population		Quintile gradient	
Code	Estimate (%)	Source	Estimates (Q1 to Q5, %)	Source
HA_CPHT_MW_EVT	45.2	IRA: Middle Africa	24.1, 34.8, 41.7, 47.2, 67.2	IRA: Middle Africa
HA_STIS_W_STI	5.1	IRA: Middle Africa	3.1, 4.0, 5.1, 6.2, 6.4	IRA: Middle Africa
HF.DYN.CONM.ZS	7.8	CE (2013)	3.3, 4.7, 4.5, 10.9, 17.5	CE (2013)
HF.STA.ANV4.ZS	47.0	CE (2013)	38.3, 42.2, 45.3, 49.5, 64.1	CE (2013)
KNAUL	5.6	SRA: Sub-Saharan Africa	3.8, 4.7, 5.4, 6.4, 8.1	CAG
LORA	8.0	IRA: Middle Africa	5.4, 6.7, 7.7, 9.2, 11.5	CAG
MEYER	68.0	IRA: Middle Africa	45.6, 57.3, 65.2, 78.3, 98.0	CAG
ML_FEVT_C_ADV	56.1	IRA: Middle Africa	42.9, 53.5, 57.1, 62.0, 70.5	IRA: Middle Africa
RH_ANCP_W_SKP	81.7	IRA: Middle Africa	68.0, 77.9, 83.9, 87.2, 95.9	IRA: Middle Africa
RH_DELA_C_SKP	58.6	IRA: Middle Africa	31.9, 46.9, 59.4, 67.9, 99.1	IRA: Middle Africa
RH_PCMT_W_DY2	39.2	IRA: Middle Africa	24.9, 33.0, 39.9, 45.1, 60.6	IRA: Middle Africa
SH.HIV.PMTC.ZS	45.0	CE (2019)	30.2, 37.9, 43.2, 51.8, 64.8	CAG
SH.TBS.DTEC.ZS	64.0	CE (2019)	42.9, 53.9, 61.4, 73.7, 92.2	CAG
STEPS_BL_GLUCOSE	13.7	IRA: Middle Africa	9.2, 11.6, 13.2, 15.8, 19.8	CAG
STEPS_BP_CHOLESTEROL	17.1	CE (2005)	11.5, 14.4, 16.4, 19.7, 24.6	CAG
Congo, Republic of the				
CH_ARIS_C_ABI	42.5	IRA: Middle Africa	30.4, 38.2, 42.6, 51.8, 54.7	IRA: Middle Africa
CH_DIAT_C_ADV	51.3	IRA: Middle Africa	43.7, 47.2, 53.7, 56.5, 56.6	IRA: Middle Africa
HA_CPHT_MW_EVT	45.2	IRA: Middle Africa	24.1, 34.8, 41.7, 47.2, 67.2	IRA: Middle Africa
HA_STIS_W_STI	5.1	IRA: Middle Africa	3.1, 4.0, 5.1, 6.2, 6.4	IRA: Middle Africa
HF.DYN.CONM.ZS	21.0	CE (2014)	15.3, 22.3, 21.6, 23.7, 21.6	CE (2014)
HF.STA.ANV4.ZS	79.2	CE (2014)	57.6, 76.3, 88.3, 90.5, 92.3	CE (2014)
KNAUL	5.6	SRA: Sub-Saharan Africa	3.9, 5.0, 5.7, 6.3, 7.3	CAG
LORA	8.0	CE	5.5, 7.2, 8.2, 9.1, 10.4	CAG
MEYER	68.0	IRA: Middle Africa	47.1, 60.9, 69.4, 77.1, 88.4	CAG
ML_FEVT_C_ADV	56.1	IRA: Middle Africa	42.9, 53.5, 57.1, 62.0, 70.5	IRA: Middle Africa
RH_ANCP_W_SKP	81.7	IRA: Middle Africa	68.0, 77.9, 83.9, 87.2, 95.9	IRA: Middle Africa
RH_DELA_C_SKP	58.6	IRA: Middle Africa	31.9, 46.9, 59.4, 67.9, 99.1	IRA: Middle Africa
RH_PCMT_W_DY2	39.2	IRA: Middle Africa	24.9, 33.0, 39.9, 45.1, 60.6	IRA: Middle Africa
SH.HIV.PMTC.ZS	10.0	CE (2019)	6.9, 8.9, 10.2, 11.3, 13.0	CAG
SH.TBS.DTEC.ZS	59.0	CE (2019)	40.9, 52.8, 60.2, 66.9, 76.7	CAG
STEPS_BL_GLUCOSE	20.8	CE (2004)	14.4, 18.6, 21.2, 23.6, 27.0	CAG
STEPS_BP_CHOLESTEROL	33.3	CE (2004)	23.1, 29.8, 34.0, 37.7, 43.3	CAG
Côte d'Ivoire				
CH_ARIS_C_ABI	32.6	IRA: Western Africa	24.4, 31.8, 28.1, 36.8, 47.9	IRA: Western Africa
CH_DIAT_C_ADV	59.6	IRA: Western Africa	55.6, 56.7, 59.8, 61.4, 68.9	IRA: Western Africa
HA_CPHT_MW_EVT	28.5	IRA: Western Africa	14.9, 19.0, 24.0, 31.6, 46.1	IRA: Western Africa
HA_STIS_W_STI	9.6	IRA: Western Africa	7.8, 8.7, 8.9, 11.1, 11.1	IRA: Western Africa
HF.DYN.CONM.ZS	16.4	CE (2016)	10.8, 12.3, 15.7, 21.4, 22.9	CE (2016)

Utilization input	Total population		Quintile gradient	
Code	Estimate (%)	Source	Estimates (Q1 to Q5, %)	Source
HF.STA.ANV4.ZS	51.7	CE (2016)	32.3, 40.7, 47.9, 70.2, 84.4	CE (2016)
KNAUL	0.2	IRA: Western Africa	0.1, 0.2, 0.2, 0.2, 0.3	CAG
LORA	65.0	IRA: Western Africa	47.8, 55.8, 61.5, 75.9, 88.6	CAG
MEYER	27.6	IRA: Western Africa	20.3, 23.7, 26.1, 32.2, 37.6	CAG
ML_FEVT_C_ADV	64.1	IRA: Western Africa	57.0, 61.5, 62.9, 68.2, 74.8	IRA: Western Africa
RH_ANCP_W_SKP	83.3	IRA: Western Africa	69.8, 77.1, 83.0, 92.0, 99.2	IRA: Western Africa
RH_DELA_C_SKP	63.4	IRA: Western Africa	37.1, 48.9, 61.3, 80.5, 97.7	IRA: Western Africa
RH_PCMT_W_DY2	64.4	IRA: Western Africa	48.2, 57.2, 63.9, 73.7, 84.5	IRA: Western Africa
SH.HIV.PMTC.ZS	80.0	CE (2019)	58.9, 68.6, 75.7, 93.4, 100.0	CAG
SH.TBS.DTEC.ZS	60.0	CE (2019)	44.1, 51.5, 56.8, 70.0, 81.8	CAG
STEPS_BL_GLUCOSE	10.7	IRA: Western Africa	7.9, 9.2, 10.1, 12.5, 14.6	CAG
STEPS_BP_CHOLESTEROL	25.9	CE (2005)	19.1, 22.2, 24.5, 30.2, 35.3	CAG
Ethiopia				
CH_ARIS_C_ABI	22.9	CE (2016)	18.0, 22.7, 24.0, 26.7, 21.1	CE (2016)
CH_DIAT_C_ADV	47.0	CE (2016)	42.9, 38.6, 47.6, 47.3, 63.6	CE (2016)
HA_CPHT_MW_EVT	44.9	CE (2016)	26.0, 34.9, 38.4, 47.8, 66.2	CE (2016)
HA_STIS_W_STI	0.3	CE (2016)	0.4, 0.1, 0.1, 0.4, 0.4	CE (2016)
HF.DYN.CONM.ZS	35.3	CE (2016)	19.8, 30.9, 37.1, 40.2, 47.8	CE (2016)
HF.STA.ANV4.ZS	33.7	CE (2016)	17.9, 26.5, 30.9, 40.9, 64.0	CE (2016)
KNAUL	11.0	IRA: Eastern Africa	7.7, 8.6, 9.8, 12.3, 17.7	CAG
LORA	11.0	CE	7.7, 8.6, 9.8, 12.3, 17.7	CAG
MEYER	47.0	CE	32.8, 36.7, 42.0, 52.6, 75.5	CAG
ML_FEVT_C_ADV	35.3	CE (2016)	23.8, 30.4, 33.0, 42.3, 50.5	CE (2016)
RH_ANCP_W_SKP	63.6	CE (2016)	47.8, 57.6, 65.1, 69.0, 87.7	CE (2016)
RH_DELA_C_SKP	33.9	CE (2016)	14.0, 27.2, 30.2, 35.8, 78.3	CE (2016)
RH_PCMT_W_DY2	16.5	CE (2016)	7.3, 10.8, 14.3, 15.3, 43.0	CE (2016)
SH.HIV.PMTC.ZS	74.0	CE (2019)	51.7, 57.8, 66.1, 82.8, 100.0	CAG
SH.TBS.DTEC.ZS	71.0	CE (2019)	49.6, 55.4, 63.4, 79.4, 100.0	CAG
STEPS_BL_GLUCOSE	5.9	CE (2015)	4.1, 4.6, 5.3, 6.6, 9.5	CAG
STEPS_BP_CHOLESTEROL	5.6	CE (2015)	3.9, 4.4, 5.0, 6.3, 9.0	CAG
Gambia, Republic of the				
CH_ARIS_C_ABI	32.6	IRA: Western Africa	24.4, 31.8, 28.1, 36.8, 47.9	IRA: Western Africa
CH_DIAT_C_ADV	59.6	IRA: Western Africa	55.6, 56.7, 59.8, 61.4, 68.9	IRA: Western Africa
HA_CPHT_MW_EVT	28.5	IRA: Western Africa	14.9, 19.0, 24.0, 31.6, 46.1	IRA: Western Africa
HA_STIS_W_STI	9.6	IRA: Western Africa	7.8, 8.7, 8.9, 11.1, 11.1	IRA: Western Africa
HF.DYN.CONM.ZS	8.1	CE (2013)	4.3, 4.8, 5.6, 10.7, 15.4	CE (2013)
HF.STA.ANV4.ZS	76.9	CE (2013)	73.5, 78.2, 74.4, 75.6, 84.7	CE (2013)
KNAUL	0.2	IRA: Western Africa	0.2, 0.2, 0.2, 0.2, 0.3	CAG
LORA	65.0	IRA: Western Africa	49.1, 56.2, 60.1, 73.5, 88.5	CAG

Utilization input	Total population		Quintile gradient	
Code	Estimate (%)	Source	Estimates (Q1 to Q5, %)	Source
MEYER	0.0	CE	0.0, 0.0, 0.0, 0.0, 0.0	CAG
ML_FEVT_C_ADV	64.1	IRA: Western Africa	57.0, 61.5, 62.9, 68.2, 74.8	IRA: Western Africa
RH_ANCP_W_SKP	83.3	IRA: Western Africa	69.8, 77.1, 83.0, 92.0, 99.2	IRA: Western Africa
RH_DELA_C_SKP	63.4	IRA: Western Africa	37.1, 48.9, 61.3, 80.5, 97.7	IRA: Western Africa
RH_PCMT_W_DY2	64.4	IRA: Western Africa	48.2, 57.2, 63.9, 73.7, 84.5	IRA: Western Africa
SH.HIV.PMTC.ZS	49.0	CE (2019)	37.0, 42.3, 45.3, 55.4, 66.7	CAG
SH.TBS.DTEC.ZS	71.0	CE (2019)	53.7, 61.4, 65.6, 80.3, 96.6	CAG
STEPS_BL_GLUCOSE	10.7	IRA: Western Africa	8.1, 9.2, 9.9, 12.1, 14.6	CAG
STEPS_BP_CHOLESTEROL	26.3	CE (2010)	19.9, 22.7, 24.3, 29.7, 35.8	CAG
Ghana				
CH_ARIS_C_ABI	43.1	CE (2014)	32.3, 42.0, 37.2, 48.7, 63.3	IRA: Western Africa
CH_DIAT_C_ADV	69.7	CE (2014)	70.2, 69.2, 66.4, 75.4, 67.9	CE (2014)
HA_CPHT_MW_EVT	35.5	CE (2014)	21.2, 27.0, 31.4, 39.4, 50.6	CE (2014)
HA_STIS_W_STI	5.1	CE (2014)	5.0, 5.3, 5.5, 5.8, 4.1	CE (2014)
HF.DYN.CONM.ZS	22.2	CE (2014)	21.4, 24.7, 24.2, 21.8, 19.6	CE (2014)
HF.STA.ANV4.ZS	85.9	CE (2014)	76.3, 80.3, 85.6, 93.1, 97.3	CE (2014)
KNAUL	0.2	IRA: Western Africa	0.2, 0.2, 0.2, 0.2, 0.2	CAG
LORA	65.0	IRA: Western Africa	55.9, 62.2, 64.4, 70.8, 73.6	CAG
MEYER	27.6	IRA: Western Africa	23.7, 26.4, 27.3, 30.1, 31.2	CAG
ML_FEVT_C_ADV	69.0	CE (2019)	68.5, 68.9, 67.0, 65.2, 78.7	CE (2019)
RH_ANCP_W_SKP	97.4	CE (2019)	93.2, 96.2, 99.1, 99.3, 100.0	CE (2019)
RH_DELA_C_SKP	75.8	CE (2014)	50.6, 63.5, 77.9, 95.0, 97.6	CE (2014)
RH_PCMT_W_DY2	81.1	CE (2014)	64.6, 74.7, 83.2, 93.0, 95.0	CE (2014)
SH.HIV.PMTC.ZS	75.0	CE (2019)	64.5, 71.7, 74.3, 81.7, 84.9	CAG
SH.TBS.DTEC.ZS	34.0	CE (2019)	29.3, 32.5, 33.7, 37.0, 38.5	CAG
STEPS_BL_GLUCOSE	4.5	CE (2006)	3.9, 4.3, 4.5, 4.9, 5.1	CAG
STEPS_BP_CHOLESTEROL	36.7	CE (2006)	31.6, 35.1, 36.4, 40.0, 41.5	CAG
Guinea				
CH_ARIS_C_ABI	37.3	CE (2018)	28.0, 36.4, 32.2, 42.1, 54.8	IRA: Western Africa
CH_DIAT_C_ADV	67.0	CE (2018)	49.8, 63.8, 70.6, 74.9, 78.4	CE (2018)
HA_CPHT_MW_EVT	14.2	CE (2018)	5.6, 7.3, 10.2, 16.1, 27.4	CE (2018)
HA_STIS_W_STI	12.8	CE (2018)	10.9, 12.0, 12.8, 15.4, 13.0	CE (2018)
HF.DYN.CONM.ZS	9.0	CE (2016)	5.4, 8.8, 10.3, 8.8, 12.1	CE (2016)
HF.STA.ANV4.ZS	51.8	CE (2016)	31.0, 41.5, 51.6, 64.7, 77.7	CE (2016)
KNAUL	0.2	IRA: Western Africa	0.1, 0.2, 0.2, 0.2, 0.3	CAG
LORA	65.0	IRA: Western Africa	42.3, 56.7, 62.9, 76.7, 90.6	CAG
MEYER	27.6	IRA: Western Africa	18.0, 24.1, 26.7, 32.6, 38.5	CAG
ML_FEVT_C_ADV	62.3	CE (2018)	38.5, 60.2, 65.7, 70.5, 78.9	CE (2018)
RH_ANCP_W_SKP	81.7	CE (2018)	68.1, 77.6, 80.6, 92.8, 95.0	CE (2018)

Utilization input	Total population		Quintile gradient	
Code	Estimate (%)	Source	Estimates (Q1 to Q5, %)	Source
RH_DELA_C_SKP	57.5	CE (2018)	28.6, 43.3, 53.2, 81.9, 95.9	CE (2018)
RH_PCMT_W_DY2	48.6	CE (2018)	30.4, 43.6, 45.5, 63.4, 69.5	CE (2018)
SH.HIV.PMTC.ZS	65.0	IRA: Western Africa	42.3, 56.7, 62.9, 76.7, 90.6	CAG
SH.TBS.DTEC.ZS	73.0	CE (2019)	47.5, 63.7, 70.6, 86.2, 100.0	CAG
STEPS_BL_GLUCOSE	10.7	IRA: Western Africa	7.0, 9.3, 10.3, 12.6, 14.9	CAG
STEPS_BP_CHOLESTEROL	9.8	CE (2009)	6.4, 8.5, 9.5, 11.6, 13.7	CAG
Haiti				
CH_ARIS_C_ABI	28.6	CE (2016)	21.0, 20.3, 31.7, 38.2, 37.6	CE (2016)
CH_DIAT_C_ADV	37.8	CE (2016)	30.2, 34.7, 40.0, 43.1, 46.3	CE (2016)
HA_CPHT_MW_EVT	52.0	CE (2016)	31.9, 41.0, 51.9, 58.4, 66.3	CE (2016)
HA_STIS_W_STI	11.6	CE (2016)	11.2, 10.4, 12.3, 11.7, 12.2	CE (2016)
HF.DYN.CONM.ZS	31.3	CE (2012)	29.9, 30.1, 34.4, 33.8, 28.0	CE (2012)
HF.STA.ANV4.ZS	70.5	CE (2016)	52.3, 60.5, 72.4, 76.0, 88.2	CE (2016)
KNAUL	0.8	CE	0.6, 0.7, 0.8, 0.9, 1.1	CAG
LORA	48.2	IRA: Caribbean	34.3, 41.5, 50.9, 56.5, 65.0	CAG
MEYER	38.0	IRA: Caribbean	27.0, 32.7, 40.1, 44.5, 51.2	CAG
ML_FEVT_C_ADV	46.8	CE (2016)	31.2, 46.5, 51.6, 49.3, 65.7	CE (2016)
RH_ANCP_W_SKP	90.4	CE (2016)	80.6, 90.8, 92.0, 94.7, 98.5	CE (2016)
RH_DELA_C_SKP	41.5	CE (2016)	15.2, 28.3, 44.1, 59.9, 81.8	CE (2016)
RH_PCMT_W_DY2	31.0	CE (2016)	11.7, 24.7, 31.6, 43.3, 62.1	CE (2016)
SH.HIV.PMTC.ZS	86.0	CE (2019)	61.2, 74.0, 90.8, 100.0, 100.0	CAG
SH.TBS.DTEC.ZS	68.0	CE (2019)	48.4, 58.5, 71.8, 79.6, 91.7	CAG
STEPS_BL_GLUCOSE	9.9	IGA: Lower middle income	7.1, 8.5, 10.5, 11.6, 13.4	CAG
STEPS_BP_CHOLESTEROL	25.4	IGA: Lower middle income	18.0, 21.8, 26.8, 29.7, 34.2	CAG
Kenya				
CH_ARIS_C_ABI	54.8	CE (2014)	52.3, 48.0, 55.0, 65.1, 60.8	CE (2014)
CH_DIAT_C_ADV	66.0	CE (2014)	68.1, 63.8, 68.9, 65.1, 62.2	CE (2014)
HA_CPHT_MW_EVT	78.2	CE (2014)	67.4, 75.0, 77.4, 81.0, 84.8	CE (2014)
HA_STIS_W_STI	2.0	CE (2014)	2.0, 2.7, 1.9, 2.3, 1.3	CE (2014)
HF.DYN.CONM.ZS	53.2	CE (2014)	29.7, 54.3, 60.0, 60.8, 57.5	CE (2014)
HF.STA.ANV4.ZS	54.9	CE (2014)	43.9, 47.2, 53.6, 59.8, 73.7	CE (2014)
KNAUL	11.0	IRA: Eastern Africa	8.9, 10.8, 11.1, 12.4, 12.3	CAG
LORA	11.2	IRA: Eastern Africa	9.1, 11.0, 11.4, 12.6, 12.6	CAG
MEYER	15.0	CE	12.2, 14.7, 15.2, 16.9, 16.7	CAG
ML_FEVT_C_ADV	72.4	CE (2015)	63.0, 72.9, 74.3, 78.3, 77.4	CE (2015)
RH_ANCP_W_SKP	94.0	CE (2015)	88.7, 94.8, 93.6, 96.6, 97.8	CE (2015)
RH_DELA_C_SKP	64.5	CE (2014)	34.0, 54.0, 66.6, 84.1, 92.9	CE (2014)
RH_PCMT_W_DY2	52.9	CE (2014)	31.0, 48.2, 52.3, 65.2, 74.4	CE (2014)
SH.HIV.PMTC.ZS	94.0	CE (2019)	76.5, 92.1, 95.3, 100.0, 100.0	CAG

Utilization input	Total population		Quintile gradient	
Code	Estimate (%)	Source	Estimates (Q1 to Q5, %)	Source
SH.TBS.DTEC.ZS	60.0	CE (2019)	48.8, 58.8, 60.8, 67.4, 67.0	CAG
STEPS_BL_GLUCOSE	5.2	IRA: Eastern Africa	4.2, 5.1, 5.2, 5.8, 5.8	CAG
STEPS_BP_CHOLESTEROL	10.1	CE (2015)	8.2, 9.9, 10.2, 11.3, 11.3	CAG
Lao People's Democratic Republic				
CH_ARIS_C_ABI	49.0	SRA: South-eastern Asia	49.3, 49.3, 53.3, 38.3, 56.3	SRA: South-eastern Asia
CH_DIAT_C_ADV	66.6	SRA: South-eastern Asia	63.3, 67.5, 65.0, 67.9, 71.0	SRA: South-eastern Asia
HA_CPHT_MW_EVT	17.2	SRA: South-eastern Asia	8.0, 11.4, 14.0, 17.5, 31.2	SRA: South-eastern Asia
HA_STIS_W_STI	2.4	SRA: South-eastern Asia	2.0, 2.4, 2.0, 3.6, 3.8	SRA: South-eastern Asia
HF.DYN.CONM.ZS	54.3	CE (2017)	46.1, 55.1, 58.9, 60.1, 50.7	CE (2017)
HF.STA.ANV4.ZS	62.6	CE (2017)	32.6, 53.9, 68.2, 83.3, 91.1	CE (2017)
KNAUL	9.0	SRA: South-eastern Asia	7.1, 8.3, 8.9, 10.1, 11.5	CAG
LORA	42.7	SRA: South-eastern Asia	33.6, 39.6, 42.4, 47.8, 54.7	CAG
MEYER	3.0	CE	2.4, 2.8, 3.0, 3.4, 3.8	CAG
ML_FEVT_C_ADV	59.6	SRA: South-eastern Asia	51.8, 55.6, 56.8, 67.9, 71.2	SRA: South-eastern Asia
RH_ANCP_W_SKP	90.8	SRA: South-eastern Asia	82.2, 88.5, 92.5, 95.6, 98.6	SRA: South-eastern Asia
RH_DELA_C_SKP	78.7	SRA: South-eastern Asia	56.2, 71.6, 82.7, 91.4, 100.00	SRA: South-eastern Asia
RH_PCMT_W_DY2	73.8	SRA: South-eastern Asia	57.1, 66.8, 76.3, 80.4, 92.2	SRA: South-eastern Asia
SH.HIV.PMTC.ZS	41.0	CE (2019)	32.3, 38.0, 40.7, 45.9, 52.6	CAG
SH.TBS.DTEC.ZS	61.0	CE (2019)	48.0, 56.6, 60.6, 68.3, 78.2	CAG
STEPS_BL_GLUCOSE	4.3	SRA: South-eastern Asia	3.4, 4.0, 4.3, 4.8, 5.5	CAG
STEPS_BP_CHOLESTEROL	22.3	CE (2008)	17.6, 20.7, 22.2, 25.0, 28.6	CAG
Malawi				
CH_ARIS_C_ABI	43.2	CE (2015)	45.3, 36.6, 46.4, 41.8, 46.8	CE (2015)
CH_DIAT_C_ADV	66.7	CE (2015)	67.8, 62.9, 68.4, 70.2, 64.4	CE (2015)
HA_CPHT_MW_EVT	76.4	CE (2015)	76.0, 75.8, 74.3, 76.8, 78.6	CE (2015)
HA_STIS_W_STI	2.8	CE (2015)	2.9, 2.5, 3.1, 2.9, 2.5	CE (2015)
HF.DYN.CONM.ZS	58.1	CE (2015)	53.3, 57.9, 58.7, 59.6, 60.6	CE (2015)
HF.STA.ANV4.ZS	48.3	CE (2015)	45.3, 46.4, 45.4, 49.8, 58.2	CE (2015)
KNAUL	11.0	IRA: Eastern Africa	10.9, 10.3, 11.2, 11.3, 11.5	CAG
LORA	11.2	IRA: Eastern Africa	11.1, 10.6, 11.4, 11.5, 11.8	CAG
MEYER	22.2	IRA: Eastern Africa	21.9, 20.8, 22.6, 22.7, 23.2	CAG
ML_FEVT_C_ADV	54.4	CE (2017)	58.8, 46.9, 57.2, 52.6, 57.0	CE (2017)
RH_ANCP_W_SKP	97.9	CE (2017)	96.7, 97.4, 98.6, 99.3, 98.2	CE (2017)
RH_DELA_C_SKP	90.7	CE (2015)	87.6, 89.8, 89.7, 92.9, 95.5	CE (2015)
RH_PCMT_W_DY2	42.4	CE (2015)	38.7, 38.9, 42.3, 47.0, 48.2	CE (2015)
SH.HIV.PMTC.ZS	100.0	CE (2019)	99.0, 94.0, 100.0, 100.0, 100.0	CAG
SH.TBS.DTEC.ZS	62.0	CE (2019)	61.4, 58.3, 63.1, 63.4, 64.8	CAG
STEPS_BL_GLUCOSE	5.6	CE (2009)	5.5, 5.3, 5.7, 5.7, 5.9	CAG
STEPS_BP_CHOLESTEROL	8.7	CE (2009)	8.6, 8.2, 8.9, 8.9, 9.1	CAG

Utilization input	Total population		Quintile gradient	
Code	Estimate (%)	Source	Estimates (Q1 to Q5, %)	Source
Mali				
CH_ARIS_C_ABI	23.9	CE (2018)	25.3, 19.9, 7.4, 39.6, 23.5	CE (2018)
CH_DIAT_C_ADV	49.0	CE (2018)	46.6, 44.8, 47.7, 51.3, 58.0	CE (2018)
HA_CPHT_MW_EVT	16.6	CE (2018)	6.2, 8.2, 10.2, 18.1, 35.4	CE (2018)
HA_STIS_W_STI	13.6	CE (2018)	11.5, 10.8, 11.3, 13.6, 19.8	CE (2018)
HF.DYN.CONM.ZS	17.5	CE (2015)	5.2, 9.7, 14.7, 22.8, 34.4	CE (2015)
HF.STA.ANV4.ZS	39.0	CE (2015)	21.3, 28.7, 35.5, 45.3, 68.4	CE (2015)
KNAUL	0.2	IRA: Western Africa	0.1, 0.2, 0.2, 0.2, 0.3	CAG
LORA	65.0	IRA: Western Africa	48.1, 50.0, 52.9, 77.2, 96.5	CAG
MEYER	35.0	CE	25.9, 26.9, 28.5, 41.6, 52.0	CAG
ML_FEVT_C_ADV	52.8	CE (2018)	49.2, 44.2, 48.5, 59.1, 71.8	CE (2018)
RH_ANCP_W_SKP	79.4	CE (2018)	68.5, 69.1, 77.2, 87.4, 97.2	CE (2018)
RH_DELA_C_SKP	69.0	CE (2018)	47.2, 55.8, 63.6, 86.7, 97.2	CE (2018)
RH_PCMT_W_DY2	56.1	CE (2018)	47.3, 47.6, 48.1, 63.8, 77.9	CE (2018)
SH.HIV.PMTC.ZS	21.0	CE (2019)	15.5, 16.1, 17.1, 24.9, 31.2	CAG
SH.TBS.DTEC.ZS	67.0	CE (2019)	49.5, 51.5, 54.6, 79.5, 99.5	CAG
STEPS_BL_GLUCOSE	10.7	IRA: Western Africa	7.9, 8.2, 8.7, 12.7, 15.9	CAG
STEPS_BP_CHOLESTEROL	15.9	CE (2007)	11.8, 12.2, 12.9, 18.9, 23.6	CAG
Mozambique				
CH_ARIS_C_ABI	57.2	CE (2015)	50.1, 48.7, 49.9, 74.2, 64.5	CE (2015)
CH_DIAT_C_ADV	59.6	CE (2015)	45.2, 50.7, 71.9, 73.7, 61.8	CE (2015)
HA_CPHT_MW_EVT	51.6	CE (2015)	36.0, 40.0, 47.8, 59.4, 68.3	CE (2015)
HA_STIS_W_STI	3.7	CE (2015)	2.5, 2.1, 2.6, 3.9, 6.7	CE (2015)
HF.DYN.CONM.ZS	11.3	CE (2011)	2.9, 5.4, 7.0, 13.9, 29.8	CE (2011)
HF.STA.ANV4.ZS	48.6	CE (2011)	36.9, 43.0, 50.2, 55.7, 64.9	CE (2011)
KNAUL	11.0	IRA: Eastern Africa	8.0, 8.7, 10.2, 12.9, 16.0	CAG
LORA	11.2	IRA: Eastern Africa	8.2, 8.9, 10.5, 13.2, 16.4	CAG
MEYER	22.2	IRA: Eastern Africa	16.1, 17.5, 20.6, 26.0, 32.2	CAG
ML_FEVT_C_ADV	68.6	CE (2018)	57.9, 67.2, 71.0, 78.0, 82.7	CE (2018)
RH_ANCP_W_SKP	93.7	CE (2018)	93.3, 89.0, 91.8, 98.6, 99.2	CE (2018)
RH_DELA_C_SKP	56.2	CE (2011)	34.2, 39.8, 53.3, 75.5, 90.6	CE (2011)
RH_PCMT_W_DY2	47.0	IRA: Eastern Africa	36.8, 40.8, 46.0, 51.5, 65.6	IRA: Eastern Africa
SH.HIV.PMTC.ZS	100.0	CE (2019)	72.5, 79.1, 93.0, 100,0, 100.0	CAG
SH.TBS.DTEC.ZS	88.0	CE (2019)	63.8, 69.6, 81.8, 100.0, 100.0	CAG
STEPS_BL_GLUCOSE	3.8	CE (2005)	2.8, 3.0, 3.5, 4.5, 5.5	CAG
STEPS_BP_CHOLESTEROL	34.9	CE (2005)	25.3, 27.6, 32.4, 41.0, 50.7	CAG
Myanmar				
CH_ARIS_C_ABI	47.6	CE (2016)	47.8, 47.9, 51.7, 37.2, 54.6	SRA: South-eastern Asia
CH_DIAT_C_ADV	66.6	CE (2016)	61.4, 64.4, 61.7, 75.6, 81.9	CE (2016)

Utilization input	Total population		Quintile gradient	
Code	Estimate (%)	Source	Estimates (Q1 to Q5, %)	Source
HA_CPHT_MW_EVT	21.1	CE (2016)	10.9, 15.2, 16.4, 23.1, 38.0	CE (2016)
HA_STIS_W_STI	0.1	CE (2016)	0.1, 0.1, 0.0, 0.3, 0.2	CE (2016)
HF.DYN.CONM.ZS	51.3	CE (2015)	46.5, 49.7, 50.2, 54.9, 55.8	CE (2015)
HF.STA.ANV4.ZS	59.6	CE (2015)	38.4, 52.4, 61.1, 71.5, 89.9	CE (2015)
KNAUL	9.0	SRA: South-eastern Asia	7.4, 8.3, 8.0, 11.6, 12.5	CAG
LORA	49.0	CE	40.2, 45.4, 43.5, 63.0, 68.0	CAG
MEYER	3.0	SRA: South-eastern Asia	2.5, 2.8, 2.7, 3.9, 4.2	CAG
ML_FEVT_C_ADV	66.7	CE (2016)	62.5, 65.9, 58.4, 77.5, 76.7	CE (2016)
RH_ANCP_W_SKP	82.9	CE (2016)	69.7, 78.0, 86.5, 91.1, 99.1	CE (2016)
RH_DELA_C_SKP	64.9	CE (2016)	39.6, 55.9, 69.9, 82.3, 98.4	CE (2016)
RH_PCMT_W_DY2	71.2	CE (2016)	58.0, 66.5, 77.1, 74.7, 89.0	CE (2016)
SH.HIV.PMTC.ZS	85.0	CE (2019)	69.7, 78.7, 75.4, 100.0, 100.0	CAG
SH.TBS.DTEC.ZS	77.0	CE (2019)	63.1, 71.3, 68.3, 99.0, 100.0	CAG
STEPS_BL_GLUCOSE	5.9	CE (2014)	4.8, 5.5, 5.2, 7.6, 8.2	CAG
STEPS_BP_CHOLESTEROL	36.7	CE (2014)	30.1, 34.0, 32.6, 47.2, 51.0	CAG
Nepal				
CH_ARIS_C_ABI	41.4	CE (2016)	33.8, 52.1, 32.6, 49.9, 37.1	SRA: Southern Asia
CH_DIAT_C_ADV	70.1	CE (2016)	54.8, 67.1, 84.6, 69.0, 72.4	CE (2016)
HA_CPHT_MW_EVT	15.7	CE (2016)	11.2, 10.8, 15.0, 17.4, 21.1	CE (2016)
HA_STIS_W_STI	0.4	CE (2016)	0.4, 0.3, 0.3, 0.5, 0.5	CE (2016)
HF.DYN.CONM.ZS	42.8	CE (2016)	41.4, 45.2, 42.6, 41.7, 43.0	CE (2016)
HF.STA.ANV4.ZS	71.5	CE (2016)	64.5, 65.6, 70.7, 77.0, 84.1	CE (2016)
KNAUL	2.1	SRA: Southern Asia	1.7, 2.0, 2.0, 2.3, 2.5	CAG
LORA	10.0	CE	8.3, 9.3, 9.7, 11.1, 11.8	CAG
MEYER	35.5	SRA: Southern Asia	29.4, 33.2, 34.5, 39.3, 41.7	CAG
ML_FEVT_C_ADV	75.3	SRA: Southern Asia	71.7, 74.8, 74.9, 76.6, 80.2	SRA: Southern Asia
RH_ANCP_W_SKP	85.6	CE (2016)	77.0, 83.8, 85.2, 89.9, 94.6	CE (2016)
RH_DELA_C_SKP	62.7	CE (2016)	38.5, 53.9, 66.3, 73.6, 90.3	CE (2016)
RH_PCMT_W_DY2	56.7	CE (2016)	36.7, 49.7, 55.5, 68.6, 81.2	CE (2016)
SH.HIV.PMTC.ZS	51.0	CE (2019)	42.3, 47.6, 49.5, 56.4, 59.9	CAG
SH.TBS.DTEC.ZS	46.0	CE (2019)	38.1, 43.0, 44.7, 50.9, 54.1	CAG
STEPS_BL_GLUCOSE	21.3	CE (2019)	17.7, 19.9, 20.7, 23.6, 25.0	CAG
STEPS_BP_CHOLESTEROL	11.1	CE (2019)	9.2, 10.4, 10.8, 12.3, 13.0	CAG
Niger				
CH_ARIS_C_ABI	9.7	CE (2012)	6.4, 4.9, 2.1, 6.4, 31.9	CE (2012)
CH_DIAT_C_ADV	60.4	CE (2012)	47.6, 55.5, 68.3, 66.1, 61.7	CE (2012)
HA_CPHT_MW_EVT	15.9	CE (2012)	5.4, 8.0, 10.1, 14.4, 34.5	CE (2012)
HA_STIS_W_STI	1.5	CE (2012)	1.3, 1.2, 1.0, 1.7, 2.0	CE (2012)
HF.DYN.CONM.ZS	12.2	CE (2012)	8.6, 7.8, 8.4, 13.0, 23.7	CE (2012)

Utilization input	Total population		Quintile gradient	
Code	Estimate (%)	Source	Estimates (Q1 to Q5, %)	Source
HF.STA.ANV4.ZS	33.7	CE (2012)	24.9, 32.3, 32.1, 32.8, 47.8	CE (2012)
KNAUL	0.2	IRA: Western Africa	0.1, 0.2, 0.2, 0.2, 0.3	CAG
LORA	65.0	IRA: Western Africa	44.7, 49.8, 51.6, 65.0, 100.0	CAG
MEYER	27.6	IRA: Western Africa	19.0, 21.1, 21.9, 27.6, 48.1	CAG
ML_FEVT_C_ADV	62.2	CE (2012)	51.3, 57.8, 63.7, 68.5, 71.1	CE (2012)
RH_ANCP_W_SKP	84.0	CE (2012)	73.1, 80.7, 85.0, 85.3, 96.3	CE (2012)
RH_DELA_C_SKP	32.3	CE (2012)	14.0, 22.1, 24.2, 31.9, 73.3	CE (2012)
RH_PCMT_W_DY2	36.9	CE (2012)	23.9, 28.0, 31.9, 39.5, 63.1	CE (2012)
SH.HIV.PMTC.ZS	43.0	CE (2019)	29.6, 32.9, 34.1, 43.0, 75.0	CAG
SH.TBS.DTEC.ZS	59.0	CE (2019)	40.6, 45.2, 46.8, 59.0, 100.0	CAG
STEPS_BL_GLUCOSE	22.5	CE (2007)	15.5, 17.2, 17.9, 22.5, 39.3	CAG
STEPS_BP_CHOLESTEROL	36.3	CE (2007)	25.0, 27.8, 28.8, 36.3, 63.3	CAG
Nigeria				
CH_ARIS_C_ABI	27.3	CE (2018)	27.1, 24.7, 31.2, 21.6, 33.8	CE (2018)
CH_DIAT_C_ADV	64.8	CE (2018)	60.1, 63.2, 66.4, 65.4, 81.3	CE (2018)
HA_CPHT_MW_EVT	26.0	CE (2013)	4.8, 12.8, 23.3, 32.2, 48.5	CE (2013)
HA_STIS_W_STI	7.7	CE (2018)	5.8, 8.1, 9.2, 8.0, 7.5	CE (2018)
HF.DYN.CONM.ZS	12.7	CE (2016)	3.1, 7.3, 11.3, 17.7, 24.7	CE (2016)
HF.STA.ANV4.ZS	49.7	CE (2016)	21.7, 34.7, 50.6, 68.2, 84.5	CE (2016)
KNAUL	0.2	CE	0.1, 0.2, 0.2, 0.2, 0.3	CAG
LORA	65.0	CE	37.7, 49.3, 67.0, 79.2, 99.3	CAG
MEYER	4.0	CE	2.3, 3.0, 4.1, 4.9, 6.1	CAG
ML_FEVT_C_ADV	72.8	CE (2018)	67.8, 70.4, 72.4, 79.1, 85.2	CE (2018)
RH_ANCP_W_SKP	66.3	CE (2018)	40.3, 53.0, 72.8, 83.5, 92.7	CE (2018)
RH_DELA_C_SKP	44.8	CE (2018)	12.7, 23.8, 46.9, 67.9, 87.1	CE (2018)
RH_PCMT_W_DY2	41.8	CE (2018)	18.1, 24.6, 41.9, 61.4, 74.5	CE (2018)
SH.HIV.PMTC.ZS	43.0	CE (2019)	24.9, 32.6, 44.3, 52.4, 65.7	CAG
SH.TBS.DTEC.ZS	27.0	CE (2019)	15.6, 20.5, 27.8, 32.9, 41.2	CAG
STEPS_BL_GLUCOSE	10.7	IRA: Western Africa	6.2, 8.1, 11.0, 13.0, 16.3	CAG
STEPS_BP_CHOLESTEROL	34.8	CE (2003)	20.2, 26.4, 35.9, 42.4, 53.2	CAG
Samoa				
CH_ARIS_C_ABI	31.1	RA: Oceania	17.1, 20.2, 40.7, 28.6, 48.2	RA: Oceania
CH_DIAT_C_ADV	37.7	RA: Oceania	34.9, 30.3, 29.5, 40.9, 51.2	RA: Oceania
HA_CPHT_MW_EVT	25.5	RA: Oceania	14.8, 19.8, 23.3, 29.2, 37.4	RA: Oceania
HA_STIS_W_STI	4.5	RA: Oceania	2.7, 3.5, 5.0, 5.3, 5.8	RA: Oceania
HF.DYN.CONM.ZS	40.6	RA: Oceania	30.9, 43.0, 38.3, 42.6, 47.9	RA: Oceania
HF.STA.ANV4.ZS	72.5	IGA: Lower middle income	55.3, 65.0, 72.9, 82.0, 93.3	IGA: Lower middle income
KNAUL	4.0	IGA: Lower middle income	2.7, 3.3, 4.0, 4.5, 5.6	CAG
LORA	36.2	IGA: Lower middle income	24.0, 29.4, 36.4, 41.1, 50.5	CAG

Utilization input	Total population		Quintile gradient	
Code	Estimate (%)	Source	Estimates (Q1 to Q5, %)	Source
MEYER	31.9	IGA: Lower middle income	21.2, 26.0, 32.1, 36.2, 44.5	CAG
ML_FEVT_C_ADV	49.5	RA: Oceania	31.4, 44.4, 47.8, 62.0, 65.6	RA: Oceania
RH_ANCP_W_SKP	74.5	RA: Oceania	56.0, 65.5, 78.0, 83.0, 93.5	RA: Oceania
RH_DELA_C_SKP	56.8	RA: Oceania	32.8, 39.2, 56.8, 70.1, 91.7	RA: Oceania
RH_PCMT_W_DY2	45.5	RA: Oceania	22.7, 32.1, 45.1, 56.6, 73.7	RA: Oceania
SH.HIV.PMTC.ZS	90.5	RA: Oceania	60.1, 73.6, 91.1, 100.0, 100.0	CAG
SH.TBS.DTEC.ZS	87.0	CE (2019)	57.8, 70.8, 87.6, 98.8, 100.0	CAG
STEPS_BL_GLUCOSE	22.1	CE (2002)	14.7, 18.0, 22.3, 25.1, 30.8	CAG
STEPS_BP_CHOLESTEROL	21.1	CE (2002)	14.0, 17.2, 21.2, 24.0, 29.4	CAG
São Tomé & Príncipe				
CH_ARIS_C_ABI	42.5	IRA: Middle Africa	30.4, 38.2, 42.6, 51.8, 54.7	IRA: Middle Africa
CH_DIAT_C_ADV	51.3	IRA: Middle Africa	43.7, 47.2, 53.7, 56.5, 56.6	IRA: Middle Africa
HA_CPHT_MW_EVT	45.2	IRA: Middle Africa	24.1, 34.8, 41.7, 47.2, 67.2	IRA: Middle Africa
HA_STIS_W_STI	5.1	IRA: Middle Africa	3.1, 4.0, 5.1, 6.2, 6.4	IRA: Middle Africa
HF.DYN.CONM.ZS	42.3	CE (2014)	41.9, 42.2, 45.0, 45.0, 38.0	CE (2014)
HF.STA.ANV4.ZS	88.3	CE (2014)	77.1, 86.1, 86.9, 95.0, 98.8	CE (2014)
KNAUL	5.6	SRA: Sub-Saharan Africa	4.1, 5.0, 5.7, 6.3, 7.2	CAG
LORA	8.0	IRA: Middle Africa	5.9, 7.1, 8.1, 9.0, 10.3	CAG
MEYER	68.0	IRA: Middle Africa	49.9, 60.5, 68.7, 76.2, 87.2	CAG
ML_FEVT_C_ADV	56.1	IRA: Middle Africa	42.9, 53.5, 57.1, 62.0, 70.5	IRA: Middle Africa
RH_ANCP_W_SKP	81.7	IRA: Middle Africa	68.0, 77.9, 83.9, 87.2, 95.9	IRA: Middle Africa
RH_DELA_C_SKP	58.6	IRA: Middle Africa	31.9, 46.9, 59.4, 67.9, 99.1	IRA: Middle Africa
RH_PCMT_W_DY2	39.2	IRA: Middle Africa	24.9, 33.0, 39.9, 45.1, 60.6	IRA: Middle Africa
SH.HIV.PMTC.ZS	58.9	IRA: Middle Africa	43.2, 52.4, 59.5, 65.9, 75.5	CAG
SH.TBS.DTEC.ZS	57.0	CE (2019)	41.8, 50.7, 57.6, 63.8, 73.1	CAG
STEPS_BL_GLUCOSE	6.5	CE (2008)	4.8, 5.8, 6.6, 7.3, 8.3	CAG
STEPS_BP_CHOLESTEROL	38.6	CE (2008)	28.3, 34.3, 39.0, 43.2, 49.5	CAG
Senegal				
CH_ARIS_C_ABI	32.0	CE (2018)	18.3, 47.1, 39.0, 50.0, 9.4	CE (2018)
CH_DIAT_C_ADV	46.3	CE (2018)	44.2, 42.1, 46.4, 45.8, 59.1	CE (2018)
HA_CPHT_MW_EVT	35.0	CE (2017)	23.4, 29.2, 36.1, 39.1, 43.0	CE (2017)
HA_STIS_W_STI	3.3	CE (2017)	2.1, 3.8, 3.4, 3.7, 3.5	CE (2017)
HF.DYN.CONM.ZS	21.2	CE (2015)	12.0, 15.3, 21.8, 26.1, 32.2	CE (2015)
HF.STA.ANV4.ZS	60.1	CE (2017)	39.5, 51.2, 62.2, 66.6, 80.4	CE (2017)
KNAUL	0.2	IRA: Western Africa	0.1, 0.2, 0.2, 0.2, 0.2	CAG
LORA	65.0	IRA: Western Africa	47.7, 63.3, 69.0, 74.6, 73.0	CAG
MEYER	77.0	CE	56.5, 74.9, 81.7, 88.4, 86.5	CAG
ML_FEVT_C_ADV	50.0	CE (2019)	46.1, 44.2, 47.6, 55.2, 58.3	CE (2019)
RH_ANCP_W_SKP	97.6	CE (2019)	92.9, 97.7, 98.9, 99.4, 99.9	CE (2019)

Utilization input	Total population		Quintile gradient	
Code	Estimate (%)	Source	Estimates (Q1 to Q5, %)	Source
RH_DELA_C_SKP	76.9	CE (2019)	49.5, 68.0, 88.4, 93.0, 94.7	CE (2019)
RH_PCMT_W_DY2	80.3	CE (2019)	61.9, 82.2, 92.6, 82.1, 87.8	CE (2019)
SH.HIV.PMTC.ZS	72.0	CE (2019)	52.8, 70.1, 76.4, 82.7, 80.9	CAG
SH.TBS.DTEC.ZS	70.0	CE (2019)	51.4, 68.1, 74.3, 80.4, 78.6	CAG
STEPS_BL_GLUCOSE	3.4	CE (2015)	2.5, 3.3, 3.6, 3.9, 3.8	CAG
STEPS_BP_CHOLESTEROL	20.0	CE (2015)	14.7, 19.5, 21.2, 23.0, 22.5	CAG
Sierra Leone				
CH_ARIS_C_ABI	41.5	CE (2019)	31.1, 40.5, 35.8, 46.9, 61.0	IRA: Western Africa
CH_DIAT_C_ADV	74.6	CE (2019)	76.6, 73.0, 79.3, 71.8, 71.6	CE (2019)
HA_CPHT_MW_EVT	33.9	CE (2013)	28.6, 29.6, 30.3, 34.9, 42.3	CE (2013)
HA_STIS_W_STI	11.9	CE (2019)	8.0, 9.5, 9.8, 15.7, 15.3	CE (2019)
HF.DYN.CONM.ZS	24.4	CE (2017)	16.8, 18.4, 22.2, 34.7, 32.3	CE (2017)
HF.STA.ANV4.ZS	84.4	CE (2017)	79.7, 81.4, 85.2, 85.4, 94.3	CE (2017)
KNAUL	0.2	IRA: Western Africa	0.2, 0.2, 0.2, 0.2, 0.2	CAG
LORA	65.0	IRA: Western Africa	57.5, 60.1, 62.4, 71.0, 75.1	CAG
MEYER	27.6	IRA: Western Africa	24.4, 25.5, 26.5, 30.2, 31.9	CAG
ML_FEVT_C_ADV	75.4	CE (2019)	76.6, 75.1, 76.0, 72.5, 76.7	CE (2019)
RH_ANCP_W_SKP	98.1	CE (2019)	98.7, 98.9, 98.8, 98.5, 94.6	CE (2019)
RH_DELA_C_SKP	88.5	CE (2019)	84.4, 83.1, 88.2, 93.4, 96.8	CE (2019)
RH_PCMT_W_DY2	86.0	CE (2019)	81.4, 82.8, 87.4, 88.8, 92.2	CE (2019)
SH.HIV.PMTC.ZS	58.0	CE (2019)	51.3, 53.7, 55.7, 63.4, 67.0	CAG
SH.TBS.DTEC.ZS	77.0	CE (2019)	68.1, 71.2, 73.9, 84.2, 89.0	CAG
STEPS_BL_GLUCOSE	10.7	IRA: Western Africa	9.5, 9.9, 10.3, 11.7, 12.4	CAG
STEPS_BP_CHOLESTEROL	34.8	CE (2009)	30.8, 32.2, 33.4, 38.0, 40.2	CAG
Tajikistan				
CH_ARIS_C_ABI	75.1	CE (2017)	65.7, 84.7, 70.6, 78.5, 77.3	RA: Asia
CH_DIAT_C_ADV	52.6	CE (2017)	49.1, 51.4, 53.8, 59.2, 48.3	CE (2017)
HA_CPHT_MW_EVT	20.8	CE (2017)	11.3, 13.1, 21.4, 26.2, 31.4	CE (2017)
HA_STIS_W_STI	0.9	CE (2017)	1.0, 0.4, 0.8, 0.4, 1.7	CE (2017)
HF.DYN.CONM.ZS	25.8	CE (2012)	23.2, 22.7, 23.7, 26.1, 33.3	CE (2012)
HF.STA.ANV4.ZS	64.0	CE (2017)	46.9, 52.7, 64.0, 72.0, 83.7	CE (2017)
KNAUL	7.3	RA: Asia	6.4, 6.5, 7.1, 7.6, 8.9	CAG
LORA	60.0	SRA: Central Asia	52.5, 53.4, 58.0, 62.4, 73.0	CAG
MEYER	30.3	RA: Asia	26.5, 27.0, 29.3, 31.5, 36.9	CAG
ML_FEVT_C_ADV	44.0	CE (2017)	36.1, 45.9, 37.2, 57.7, 49.4	CE (2017)
RH_ANCP_W_SKP	92.9	CE (2017)	85.9, 92.5, 93.8, 95.9, 96.3	CE (2017)
RH_DELA_C_SKP	95.2	CE (2017)	90.9, 93.5, 95.8, 97.7, 98.1	CE (2017)
RH_PCMT_W_DY2	91.8	CE (2017)	87.4, 91.3, 91.8, 93.8, 94.4	CE (2017)
SH.HIV.PMTC.ZS	88.0	CE (2019)	77.0, 78.3, 85.0, 91.5, 100.0	CAG

Utilization input	Total population		Quintile gradient	
Code	Estimate (%)	Source	Estimates (Q1 to Q5, %)	Source
SH.TBS.DTEC.ZS	74.0	CE (2019)	64.7, 65.8, 71.5, 77.0, 90.0	CAG
STEPS_BL_GLUCOSE	8.0	RA: Asia	7.0, 7.1, 7.7, 8.3, 9.7	CAG
STEPS_BP_CHOLESTEROL	24.1	RA: Asia	21.1, 21.4, 23.3, 25.0, 29.3	CAG
Tanzania				
CH_ARIS_C_ABI	63.5	CE (2015)	61.0, 49.6, 65.9, 69.5, 67.2	CE (2015)
CH_DIAT_C_ADV	70.6	CE (2015)	64.5, 73.4, 72.1, 69.5, 72.9	CE (2015)
HA_CPHT_MW_EVT	58.5	CE (2012)	49.2, 54.7, 57.0, 60.1, 66.7	CE (2012)
HA_STIS_W_STI	3.1	CE (2012)	2.3, 3.3, 2.6, 3.3, 3.5	CE (2012)
HF.DYN.CONM.ZS	32.0	CE (2015)	20.5, 28.1, 36.1, 40.0, 35.5	CE (2015)
HF.STA.ANV4.ZS	48.0	CE (2015)	37.3, 40.4, 45.4, 56.1, 68.5	CE (2015)
KNAUL	11.0	IRA: Eastern Africa	8.9, 10.1, 10.8, 12.2, 13.2	CAG
LORA	11.2	IRA: Eastern Africa	9.1, 10.4, 11.1, 12.5, 13.5	CAG
MEYER	0.0	CE	0.0, 0.0, 0.0, 0.0, 0.0	CAG
ML_FEVT_C_ADV	75.4	CE (2017)	68.4, 77.0, 75.0, 81.9, 76.3	CE (2017)
RH_ANCP_W_SKP	97.9	CE (2017)	95.5, 98.2, 98.7, 97.7, 99.7	CE (2017)
RH_DELA_C_SKP	64.7	CE (2015)	43.7, 52.7, 61.7, 80.2, 96.2	CE (2015)
RH_PCMT_W_DY2	34.3	CE (2015)	22.3, 28.5, 32.0, 41.2, 53.7	CE (2015)
SH.HIV.PMTC.ZS	92.0	CE (2019)	74.4, 84.7, 90.5, 100.0, 100.0	CAG
SH.TBS.DTEC.ZS	59.0	CE (2019)	47.7, 54.3, 58.0, 65.6, 70.7	CAG
STEPS_BL_GLUCOSE	9.1	CE (2012)	7.4, 8.4, 9.0, 10.1, 10.9	CAG
STEPS_BP_CHOLESTEROL	26.0	CE (2012)	21.0, 23.9, 25.6, 28.9, 31.1	CAG
Uganda				
CH_ARIS_C_ABI	44.4	CE (2016)	37.3, 45.8, 45.0, 43.1, 63.0	CE (2016)
CH_DIAT_C_ADV	69.3	CE (2016)	72.9, 68.6, 68.1, 66.3, 69.2	CE (2016)
HA_CPHT_MW_EVT	79.0	CE (2016)	75.6, 75.4, 76.7, 78.4, 86.6	CE (2016)
HA_STIS_W_STI	11.6	CE (2016)	9.3, 11.5, 12.5, 12.9, 11.8	CE (2016)
HF.DYN.CONM.ZS	34.8	CE (2016)	22.4, 32.2, 35.9, 40.3, 42.2	CE (2016)
HF.STA.ANV4.ZS	61.0	CE (2016)	54.1, 59.3, 61.2, 65.0, 67.2	CE (2016)
KNAUL	11.0	CE	9.9, 10.6, 11.0, 11.3, 12.6	CAG
LORA	11.2	IRA: Eastern Africa	10.1, 10.8, 11.2, 11.6, 12.9	CAG
MEYER	0.0	CE	0.0, 0.0, 0.0, 0.0, 0.0	CAG
ML_FEVT_C_ADV	87.0	CE (2018)	84.8, 86.0, 89.2, 86.0, 92.3	CE (2018)
RH_ANCP_W_SKP	95.5	CE (2018)	95.4, 95.3, 95.0, 93.9, 97.9	CE (2018)
RH_DELA_C_SKP	75.8	CE (2016)	66.1, 67.0, 73.4, 80.8, 94.5	CE (2016)
RH_PCMT_W_DY2	54.3	CE (2016)	50.4, 47.4, 50.0, 53.1, 71.5	CE (2016)
SH.HIV.PMTC.ZS	100.0	CE (2019)	89.5, 96.1, 99.9, 100.0, 100.0	CAG
SH.TBS.DTEC.ZS	75.0	CE (2019)	67.2, 72.1, 74.9, 77.0, 86.2	CAG
STEPS_BL_GLUCOSE	1.4	CE (2014)	1.3, 1.3, 1.4, 1.4, 1.6	CAG
STEPS_BP_CHOLESTEROL	6.7	CE (2014)	6.0, 6.4, 6.7, 6.9, 7.7	CAG

Utilization input	Total population		Quintile gradient	
Code	Estimate (%)	Source	Estimates (Q1 to Q5, %)	Source
Viet Nam				
CH_ARIS_C_ABI	49.0	SRA: South-eastern Asia	49.3, 49.3, 53.3, 38.3, 56.3	SRA: South-eastern Asia
CH_DIAT_C_ADV	66.6	SRA: South-eastern Asia	63.3, 67.5, 65.0, 67.9, 71.0	SRA: South-eastern Asia
HA_CPHT_MW_EVT	17.2	SRA: South-eastern Asia	8.0, 11.4, 14.0, 17.5, 31.2	SRA: South-eastern Asia
HA_STIS_W_STI	2.4	SRA: South-eastern Asia	2.0, 2.4, 2.0, 3.6, 3.8	SRA: South-eastern Asia
HF.DYN.CONM.ZS	60.0	CE (2013)	64.4, 62.3, 57.8, 56.0, 60.0	CE (2013)
HF.STA.ANV4.ZS	74.5	CE (2013)	39.3, 68.5, 78.0, 90.0, 96.6	CE (2013)
KNAUL	9.0	CE	7.3, 8.4, 8.8, 9.8, 11.5	CAG
LORA	42.7	SRA: South-eastern Asia	34.6, 39.9, 41.7, 46.5, 54.3	CAG
MEYER	3.0	SRA: South-eastern Asia	2.4, 2.8, 2.9, 3.3, 3.8	CAG
ML_FEVT_C_ADV	59.6	SRA: South-eastern Asia	51.8, 55.6, 56.8, 67.9, 71.2	SRA: South-eastern Asia
RH_ANCP_W_SKP	90.8	SRA: South-eastern Asia	82.2, 88.5, 92.5, 95.6, 98.6	SRA: South-eastern Asia
RH_DELA_C_SKP	78.7	SRA: South-eastern Asia	56.2, 71.6, 82.7, 91.4, 100.0	SRA: South-eastern Asia
RH_PCMT_W_DY2	73.8	SRA: South-eastern Asia	57.1, 66.8, 76.3, 80.4, 92.2	SRA: South-eastern Asia
SH.HIV.PMTC.ZS	86.0	CE (2019)	69.7, 80.5, 84.0, 93.8, 100.0	CAG
SH.TBS.DTEC.ZS	60.0	CE (2019)	48.6, 56.1, 58.6, 65.4, 76.4	CAG
STEPS_BL_GLUCOSE	4.1	CE (2015)	3.3, 3.8, 4.0, 4.5, 5.2	CAG
STEPS_BP_CHOLESTEROL	30.2	CE (2015)	24.5, 28.3, 29.5, 32.9, 38.4	CAG
Zimbabwe				
CH_ARIS_C_ABI	40.1	CE (2015)	34.8, 37.6, 37.1, 31.9, 65.9	CE (2015)
CH_DIAT_C_ADV	42.3	CE (2015)	39.7, 42.9, 39.9, 40.2, 53.1	CE (2015)
HA_CPHT_MW_EVT	72.3	CE (2015)	70.2, 70.2, 70.4, 74.7, 74.7	CE (2015)
HA_STIS_W_STI	2.2	CE (2015)	2.4, 2.4, 2.0, 2.4, 1.8	CE (2015)
HF.DYN.CONM.ZS	65.8	CE (2015)	62.2, 60.5, 64.7, 68.9, 71.8	CE (2015)
HF.STA.ANV4.ZS	74.3	CE (2015)	70.8, 72.7, 75.1, 72.4, 85.0	CE (2015)
KNAUL	11.0	IRA: Eastern Africa	10.3, 10.6, 10.6, 11.2, 12.9	CAG
LORA	11.2	IRA: Eastern Africa	10.5, 10.8, 10.8, 11.4, 13.2	CAG
MEYER	22.2	IRA: Eastern Africa	20.7, 21.3, 21.3, 22.5, 26.0	CAG
ML_FEVT_C_ADV	50.7	CE (2015)	48.9, 45.1, 45.1, 51.1, 63.7	CE (2015)
RH_ANCP_W_SKP	92.0	CE (2015)	88.0, 91.1, 92.1, 92.6, 98.3	CE (2015)
RH_DELA_C_SKP	80.1	CE (2015)	65.9, 73.9, 80.0, 89.5, 96.4	CE (2015)
RH_PCMT_W_DY2	56.5	CE (2015)	46.7, 51.8, 56.1, 64.2, 68.6	CE (2015)
SH.HIV.PMTC.ZS	91.0	CE (2019)	84.9, 87.6, 87.6, 92.4, 100.0	CAG
SH.TBS.DTEC.ZS	72.0	CE (2019)	67.2, 69.3, 69.3, 73.1, 84.4	CAG
STEPS_BL_GLUCOSE	5.2	IRA: Eastern Africa	4.8, 5.0, 5.0, 5.2, 6.1	CAG
STEPS_BP_CHOLESTEROL	15.3	IRA: Eastern Africa	14.3, 14.8, 14.8, 15.6, 18.0	CAG

CAG country average gradient; CE country estimate, IGA income group average, IRA intermediate region average, RA regional average, SRA sub-region average

Simulated income distributions (proxied with GNI per capita, Gini coefficient)

Table A.7. Source of country estimate for gross national income (GNI) per capita and Gini coefficient. Data was sourced from the World Bank's World Development Indicators (WDI) database.

	GNI per capita		Gini	
**Country**	Estimate (2016 USD)	Source	Estimate	Source
Afghanistan	$558	CE (2014, NHA year)	35.8	SRA: Southern Asia
Armenia	$3,330	CE (2016, NHA year)	32.5	CE (2016, NHA year)
Benin	$1,081	CE (2013, NHA year)	37.8	CE (2018, MR year)
Burkina Faso	$602	CE (2016, NHA year)	47.3	CE (2018, MR year)
Burundi	$230	CE (2013, NHA year)	38.6	CE (2013, NHA year)
Cabo Verde	$2,772	CE (2016, NHA year)	42.4	CE (2015, MR year)
Cambodia	$903	CE (2014, NHA year)	38.1	SRA: South-eastern Asia
Cameroon	$1,258	CE (2011, NHA year)	46.6	CE (2014, MR year)
Congo, Democratic Republic of the	$443	CE (2018, NHA year)	42.1	CE (2012, MR year)
Congo, Republic of the	$2,746	CE (2015, NHA year)	48.9	CE (2011, MR year)
Côte d'Ivoire	$1,789	CE (2014, NHA year)	37.2	CE (2018, MR year)
Ethiopia	$345	CE (2011, NHA year)	35.0	CE (2015, MR year)
Gambia, Republic of the	$549	CE (2015, NHA year)	35.9	CE (2015, NHA year)
Ghana	$1,718	CE (2015, NHA year)	43.5	CE (2016, MR year)
Guinea	$655	CE (2014, NHA year)	29.6	CE (2018, MR year)
Haiti	$1,267	CE (2014, NHA year)	41.1	CE (2012, MR year)
Kenya	$1,293	CE (2016, NHA year)	40.8	CE (2015, MR year)
Lao People's Democratic Republic	$1,213	CE (2012, NHA year)	36.0	CE (2012, NHA year)
Malawi	$310	CE (2015, NHA year)	38.5	CE (2019, MR year)
Mali	$717	CE (2014, NHA year)	36.1	CE (2018, MR year)
Mozambique	$567	CE (2015, NHA year)	54.0	CE (2014, MR year)
Myanmar	$1,081	CE (2018, NHA year)	30.7	CE (2017, MR year)
Nepal	$779	CE (2016, NHA year)	32.8	CE (2010, MR year)
Niger	$496	CE (2015, NHA year)	37.3	CE (2018, MR year)
Nigeria	$2,152	CE (2016, NHA year)	35.1	CE (2018, MR year)
Samoa	$3,508	CE (2015, NHA year)	38.7	CE (2013, MR year)
São Tomé & Príncipe	$1,267	CE (2013, NHA year)	40.7	CE (2017, MR year)
Senegal	$1,187	CE (2013, NHA year)	38.1	CE (2018, MR year)
Sierra Leone	$585	CE (2013, NHA year)	35.7	CE (2018, MR year)
Tajikistan	$1,178	CE (2013, NHA year)	34.0	CE (2015, MR year)
Tanzania	$868	CE (2015, NHA year)	40.5	CE (2018, MR year)
Uganda	$709	CE (2016, NHA year)	42.8	CE (2016, NHA year)
Viet Nam	$2,179	CE (2015, NHA year)	35.7	CE (2018, MR year)
Zimbabwe	$620	CE (2010, NHA year)	50.3	CE (2019, MR year)

CE country estimate, *MR* most recent, *NHA* national health account, *SRA* sub-region average

Linkage between diseases, health services, and utilization indicators

Table A.8 Linkage between diseases, health services, and intervention utilization proxy indicators. Diseases and disease groups were adapted from National Health Accounts (NHA) data, available from the WHO Global Health Expenditure database.[20] Health services were adapted from the Disease Control Priorities, 3rd edition (DCP3).[23] Utilization proxy indicators were sourced from Demographic and Health Surveys (DHS)[24], STEPwise Approach to NCD Risk Factor Surveillance (STEPS)[25] reports, the World Bank's World Development Indicators (WDI) and Health Equity and Financial Protection Indicators (HEFPI) databases[2], and the published literature (three publications: Knaul et al., 2018; Lora et al., 2012; Meyer et al., 2010).[9, 49, 50]

Health services		Utilization		
Code	Description	Code	Description	Source
Childhood health				
**Childhood health**				
HC42	Acute pharyngitis treatment	CH_ARIS_C_ABI	Percent of children with symptoms of acute respiratory infection (ARI) who received antibiotics	DHS
Infectious & parasitic diseases				
**Diarrheal diseases**				
HC12	Diagnosis & treatment of infections (IMCI)	CH_DIAT_C_ADV	Percent of children with diarrhea who were taken to a health facility	DHS
**HIV/AIDS & other sexually transmitted diseases**				
HC17	Syndromic management of STIs	HA_STIS_W_STI	Percent of women reporting a sexually transmitted infection (STI)	DHS
HC13	ART & viral load monitoring	HA_CPHT_MW_EVT	Percent of population ever receiving an HIV test	DHS
HC23	HIV, STIs, hepatitis testing & counseling	HA_CPHT_MW_EVT	Percent of population ever receiving an HIV test	DHS
HC8	HIV & syphilis PMTCT	SH.HIV.PMTC.ZS	Percent of pregnant women with HIV who are on antiretroviral therapy (ART) for prevention of mother-to-child-transmission (PMTCT)	WDI
**Malaria**				
C7	Intermittent preventive treatment (pregnancy)	RH_ANCP_W_SKP	Percent of women with a prior live birth who received antenatal care from a skilled provider	DHS
**Tuberculosis**				
HC27	Diagnosis & treatment of TB	SH.TBS.DTEC.ZS	Tuberculosis (TB) case detection rate (all forms of TB)	WDI
**Other infectious & parasitic diseases**				
HC30	Management & referrals for fever (IMAI)	ML_FEVT_C_ADV	Percent of children with fever for whom advice or treatment was sought	DHS
Noncommunicable diseases (NCDs)				
**Cardiovascular diseases**				
HC38	Aspirin for acute myocardial infarction	STEPS_BP_CHOLESTEROL	Percentage with raised total cholesterol, currently on medication for raised cholesterol, raised blood pressure, or currently on medication for raised blood pressure	STEPS

Health services		Utilization		
Code	Description	Code	Description	Source
HC43	Management of ischemic heart disease	STEPS_BP_CHOLESTEROL	Percentage with raised total cholesterol, currently on medication for raised cholesterol, raised blood pressure, or currently on medication for raised blood pressure	STEPS
HC44	Management of heart failure	STEPS_BP_CHOLESTEROL	Percentage with raised total cholesterol, currently on medication for raised cholesterol, raised blood pressure, or currently on medication for raised blood pressure	STEPS
**Endocrine & metabolic disorders**				
HC40	Screening & management of diabetes	STEPS_BL_GLUCOSE	Percent of population with raised blood glucose or currently on medication for raised blood glucose	STEPS
**Mental/behavioral disorders & neurological conditions**				
HC49	Management of bipolar disorder	LORA	Percent of individuals with schizophrenic disorders who receive treatment	Lora et al., 2012
HC50	Management of depression	LORA	Percent of individuals with schizophrenic disorders who receive treatment	Lora et al., 2012
HC51	Management of epilepsy	MEYER	Percent of individuals with epilepsy who receive treatment	Meyer et al., 2010
HC52	Management of schizophrenia	LORA	Percent of individuals with schizophrenic disorders who receive treatment	Lora et al., 2012
HC66	Psychosocial support & counseling	LORA	Percent of individuals with schizophrenic disorders who receive treatment	Lora et al., 2012
**Other NCDs**				
HC47	Palliative care	KNAUL	Percent of individuals with health conditions most associated with serious health-related suffering whose healthcare needs are met	Knaul et al., 2018
Reproductive health				
**Family planning**				
HC4	Contraceptives	HF.DYN.CONM.ZS	Percent of women ages 15-49 years using modern contraceptive methods	HEFPI
**Maternal conditions**				
HC3	Treatment of premature membrane rupture	RH_DELA_C_SKP	Percent of women with a prior live birth who received assistance during delivery from a skilled provider	DHS
HC5	Kangaroo mother care counseling	RH_PCMT_W_DY2	Percent of women giving birth who had their first postnatal checkup 1-2 days after birth	DHS
HC7	Medical abortion	RH_ANCP_W_SKP	Percent of women with a prior live birth who received antenatal care from a skilled provider	DHS
C5	Antenatal tetanus immunization	HF.STA.ANV4.ZS	Pregnant women receiving prenatal care of at least four visits (% of pregnant women)	HEFPI
HC11	Basic emergency newborn & obstetric care	RH_DELA_C_SKP	Percent of women with a prior live birth who received assistance during delivery from a skilled provider	DHS

Health services		Utilization		
Code	Description	Code	Description	Source
HC2	Post-abortion care	RH_DELA_C_SKP	Percent of women with a prior live birth who received assistance during delivery from a skilled provider	DHS
**Perinatal conditions**				
C13	Cotrimoxazole for HIV-exposed children	CH_ARIS_C_ABI	Percent of children with symptoms of acute respiratory infection (ARI) who received antibiotics	DHS
HC1	Antibiotics for neonatal pneumonia	CH_ARIS_C_ABI	Percent of children with symptoms of acute respiratory infection (ARI) who received antibiotics	DHS
HC6	Neonatal sepsis, pneumonia, meningitis	CH_ARIS_C_ABI	Percent of children with symptoms of acute respiratory infection (ARI) who received antibiotics	DHS

B. Full model results

Table B.1. Full model results: Afghanistan.

Afghanistan				CHE risk 10% threshold (25% threshold)					
Disease category, disease, intervention	Cost ($)	OOP (%)	OOP ($)	Q1	Q2	Q3	Q4	Q5	Total
Childhood health				0.0 (0.0)	0.0 (0.0)	0.0 (0.0)	0.0 (0.0)	0.0 (0.0)	0.0 (0.0)
Childhood health				0.0 (0.0)	0.0 (0.0)	0.0 (0.0)	0.0 (0.0)	0.0 (0.0)	0.0 (0.0)
HC42 Acute pharyngitis treatment	$0.17	39.3%	$0.07	0.0 (0.0)	0.0 (0.0)	0.0 (0.0)	0.0 (0.0)	0.0 (0.0)	0.0 (0.0)
Infectious & parasitic diseases				3.5 (0.4)	0.0 (0.0)	0.0 (0.0)	0.0 (0.0)	0.0 (0.0)	0.7 (0.1)
Diarrheal diseases				0.7 (0.1)	0.0 (0.0)	0.0 (0.0)	0.0 (0.0)	0.0 (0.0)	0.1 (0.0)
HC12 Diagnosis & treatment of infections (IMCI)	$4.79	85.8%	$4.11	0.7 (0.1)	0.0 (0.0)	0.0 (0.0)	0.0 (0.0)	0.0 (0.0)	0.1 (0.0)
HIV/AIDS & other sexually transmitted diseases				1.4 (0.2)	0.0 (0.0)	0.0 (0.0)	0.0 (0.0)	0.0 (0.0)	0.3 (0.0)
HC13 ART & viral load monitoring	$71.45	13.9%	$9.90	0.1 (0.0)	0.0 (0.0)	0.0 (0.0)	0.0 (0.0)	0.0 (0.0)	0.0 (0.0)
HC17 Syndromic management of STI	$5.67	13.9%	$0.79	0.0 (0.0)	0.0 (0.0)	0.0 (0.0)	0.0 (0.0)	0.0 (0.0)	0.0 (0.0)
HC23 HIV, STIs, hepatitis testing & counseling	$4.31	13.9%	$0.60	0.0 (0.0)	0.0 (0.0)	0.0 (0.0)	0.0 (0.0)	0.0 (0.0)	0.0 (0.0)
HC8 HIV & syphilis PMTCT	$176.35	13.9%	$24.43	5.6 (0.7)	0.0 (0.0)	0.0 (0.0)	0.0 (0.0)	0.0 (0.0)	1.1 (0.1)
Malaria				0.0 (0.0)	0.0 (0.0)	0.0 (0.0)	0.0 (0.0)	0.0 (0.0)	0.0 (0.0)
C7 Intermittent preventive treatment (pregnancy)	$0.45	33.2%	$0.15	0.0 (0.0)	0.0 (0.0)	0.0 (0.0)	0.0 (0.0)	0.0 (0.0)	0.0 (0.0)
Tuberculosis				21.3 (2.4)	0.0 (0.0)	0.0 (0.0)	0.0 (0.0)	0.0 (0.0)	4.3 (0.5)
HC27 Diagnosis & treatment of TB	$135.09	12.6%	$17.05	21.3 (2.4)	0.0 (0.0)	0.0 (0.0)	0.0 (0.0)	0.0 (0.0)	4.3 (0.5)
Other infectious & parasitic diseases				0.1 (0.0)	0.0 (0.0)	0.0 (0.0)	0.0 (0.0)	0.0 (0.0)	0.0 (0.0)
HC30 Management & referrals for fever (IMAI)	$3.11	70.0%	$2.18	0.1 (0.0)	0.0 (0.0)	0.0 (0.0)	0.0 (0.0)	0.0 (0.0)	0.0 (0.0)
Noncommunicable diseases (NCDs)				5.9 (3.5)	4.9 (1.4)	3.3 (1.1)	2.5 (1.1)	2.2 (0.0)	3.7 (1.4)
Cardiovascular diseases				5.8 (5.5)	6.2 (3.1)	6.3 (3.1)	5.7 (3.8)	5.3 (0.0)	5.9 (3.1)
HC38 Aspirin for acute myocardial infarction	$0.03	78.4%	$0.02	0.0 (0.0)	0.0 (0.0)	0.0 (0.0)	0.0 (0.0)	0.0 (0.0)	0.0 (0.0)
HC43 Management of ischemic heart disease	$83.97	78.4%	$65.87	8.7 (8.0)	9.3 (0.0)	9.4 (0.0)	5.1 (0.0)	0.0 (0.0)	6.5 (1.6)
HC44 Management of heart failure	$249.96	78.4%	$196.09	8.6 (8.6)	9.4 (9.4)	9.4 (9.4)	12.1 (11.3)	16.0 (0.1)	11.1 (7.8)
Endocrine & metabolic disorders				16.8 (7.4)	18.0 (0.0)	5.2 (0.0)	0.0 (0.0)	0.0 (0.0)	8.0 (1.5)
HC40 Screening & management of diabetes	$64.16	71.9%	$46.12	16.8 (7.4)	18.0 (0.0)	5.2 (0.0)	0.0 (0.0)	0.0 (0.0)	8.0 (1.5)
Mental/behavioral disorders & neurological conditions				4.9 (2.1)	2.5 (0.8)	1.7 (0.4)	1.5 (0.0)	1.1 (0.0)	2.3 (0.7)
HC49 Management of bipolar disorder	$184.57	66.0%	$121.89	4.0 (4.0)	4.2 (4.2)	4.2 (1.9)	5.4 (0.0)	5.5 (0.0)	4.6 (2.0)
HC50 Management of depression	$16.11	66.0%	$10.64	0.5 (0.1)	0.0 (0.0)	0.0 (0.0)	0.0 (0.0)	0.0 (0.0)	0.1 (0.0)
HC51 Management of epilepsy	$27.53	66.0%	$18.18	12.1 (1.4)	0.0 (0.0)	0.0 (0.0)	0.0 (0.0)	0.0 (0.0)	2.4 (0.3)
HC52 Management of schizophrenia	$99.43	66.0%	$65.67	3.9 (3.6)	4.2 (0.0)	4.2 (0.0)	2.2 (0.0)	0.0 (0.0)	2.9 (0.7)
HC66 Psychosocial support & counseling	$64.63	66.0%	$42.68	3.9 (1.4)	4.1 (0.0)	0.3 (0.0)	0.0 (0.0)	0.0 (0.0)	1.7 (0.3)
Other NCDs				0.2 (0.1)	0.2 (0.0)	0.1 (0.0)	0.0 (0.0)	0.0 (0.0)	0.1 (0.0)
HC47 Palliative care	$64.63	83.4%	$53.91	0.2 (0.1)	0.2 (0.0)	0.1 (0.0)	0.0 (0.0)	0.0 (0.0)	0.1 (0.0)
Reproductive health				3.1 (1.6)	4.0 (0.0)	3.2 (0.0)	0.0 (0.0)	0.0 (0.0)	2.0 (0.3)
Family planning				0.2 (0.0)	0.0 (0.0)	0.0 (0.0)	0.0 (0.0)	0.0 (0.0)	0.0 (0.0)

**Afghanistan**				**CHE risk** 10% threshold (25% threshold)					
Disease category, disease, intervention	Cost ($)	OOP (%)	OOP ($)	Q1	Q2	Q3	Q4	Q5	Total
HC4 Contraceptives	$4.97	83.2%	$4.14	0.2 (0.0)	0.0 (0.0)	0.0 (0.0)	0.0 (0.0)	0.0 (0.0)	0.0 (0.0)
Maternal conditions				4.6 (2.6)	6.6 (0.0)	5.3 (0.0)	0.0 (0.0)	0.0 (0.0)	3.3 (0.5)
C5 Antenatal tetanus immunization	$0.39	75.2%	$0.29	0.0 (0.0)	0.0 (0.0)	0.0 (0.0)	0.0 (0.0)	0.0 (0.0)	0.0 (0.0)
HC11 Basic emergency newborn & obstetric care	$69.14	75.2%	$51.99	27.0 (15.4)	39.7 (0.0)	31.5 (0.0)	0.0 (0.0)	0.0 (0.0)	19.7 (3.1)
HC2 Post-abortion care	$4.54	75.2%	$3.42	0.2 (0.0)	0.0 (0.0)	0.0 (0.0)	0.0 (0.0)	0.0 (0.0)	0.0 (0.0)
HC3 Treatment of premature membrane rupture	$4.04	75.2%	$3.04	0.1 (0.0)	0.0 (0.0)	0.0 (0.0)	0.0 (0.0)	0.0 (0.0)	0.0 (0.0)
HC5 Kangaroo mother care counseling	$2.33	75.2%	$1.75	0.0 (0.0)	0.0 (0.0)	0.0 (0.0)	0.0 (0.0)	0.0 (0.0)	0.0 (0.0)
HC7 Medical abortion	$4.81	75.2%	$3.61	0.4 (0.0)	0.0 (0.0)	0.0 (0.0)	0.0 (0.0)	0.0 (0.0)	0.1 (0.0)
Perinatal conditions				0.9 (0.1)	0.0 (0.0)	0.0 (0.0)	0.0 (0.0)	0.0 (0.0)	0.2 (0.0)
C13 Cotrimoxazole for HIV-exposed children	$6.55	84.1%	$5.51	1.3 (0.1)	0.0 (0.0)	0.0 (0.0)	0.0 (0.0)	0.0 (0.0)	0.3 (0.0)
HC1 Antibiotics for neonatal pneumonia	$6.22	84.1%	$5.24	1.2 (0.1)	0.0 (0.0)	0.0 (0.0)	0.0 (0.0)	0.0 (0.0)	0.2 (0.0)
HC6 Neonatal sepsis, pneumonia, meningitis	$2.66	84.1%	$2.24	0.1 (0.0)	0.0 (0.0)	0.0 (0.0)	0.0 (0.0)	0.0 (0.0)	0.0 (0.0)

Table B.2. Full model results: Armenia.

**Armenia**				**CHE risk** 10% threshold (25% threshold)					
Disease category, disease, intervention	Cost ($)	OOP (%)	OOP ($)	Q1	Q2	Q3	Q4	Q5	Total
Childhood health				0.0 (0.0)	0.0 (0.0)	0.0 (0.0)	0.0 (0.0)	0.0 (0.0)	0.0 (0.0)
Childhood health				0.0 (0.0)	0.0 (0.0)	0.0 (0.0)	0.0 (0.0)	0.0 (0.0)	0.0 (0.0)
HC42 Acute pharyngitis treatment	$0.24	45.3%	$0.11	0.0 (0.0)	0.0 (0.0)	0.0 (0.0)	0.0 (0.0)	0.0 (0.0)	0.0 (0.0)
Infectious & parasitic diseases				2.1 (0.2)	0.0 (0.0)	0.0 (0.0)	0.0 (0.0)	0.0 (0.0)	0.4 (0.0)
Diarrheal diseases				0.0 (0.0)	0.0 (0.0)	0.0 (0.0)	0.0 (0.0)	0.0 (0.0)	0.0 (0.0)
HC12 Diagnosis & treatment of infections (IMCI)	$10.29	45.6%	$4.70	0.0 (0.0)	0.0 (0.0)	0.0 (0.0)	0.0 (0.0)	0.0 (0.0)	0.0 (0.0)
HIV/AIDS & other sexually transmitted diseases				4.1 (0.4)	0.0 (0.0)	0.0 (0.0)	0.0 (0.0)	0.0 (0.0)	0.8 (0.1)
HC13 ART & viral load monitoring	$121.57	32.6%	$39.66	0.2 (0.0)	0.0 (0.0)	0.0 (0.0)	0.0 (0.0)	0.0 (0.0)	0.0 (0.0)
HC17 Syndromic management of STI	$10.69	32.6%	$3.49	0.0 (0.0)	0.0 (0.0)	0.0 (0.0)	0.0 (0.0)	0.0 (0.0)	0.0 (0.0)
HC23 HIV, STIs, hepatitis testing & counseling	$6.08	32.6%	$1.98	0.0 (0.0)	0.0 (0.0)	0.0 (0.0)	0.0 (0.0)	0.0 (0.0)	0.0 (0.0)
HC8 HIV & syphilis PMTCT	$313.51	32.6%	$102.29	16.3 (1.5)	0.0 (0.0)	0.0 (0.0)	0.0 (0.0)	0.0 (0.0)	3.3 (0.3)
Malaria				0.0 (0.0)	0.0 (0.0)	0.0 (0.0)	0.0 (0.0)	0.0 (0.0)	0.0 (0.0)
C7 Intermittent preventive treatment (pregnancy)	$1.02	15.0%	$0.15	0.0 (0.0)	0.0 (0.0)	0.0 (0.0)	0.0 (0.0)	0.0 (0.0)	0.0 (0.0)
Tuberculosis				0.3 (0.0)	0.0 (0.0)	0.0 (0.0)	0.0 (0.0)	0.0 (0.0)	0.1 (0.0)
HC27 Diagnosis & treatment of TB	$175.65	12.0%	$21.04	0.3 (0.0)	0.0 (0.0)	0.0 (0.0)	0.0 (0.0)	0.0 (0.0)	0.1 (0.0)
Other infectious & parasitic diseases				0.0 (0.0)	0.0 (0.0)	0.0 (0.0)	0.0 (0.0)	0.0 (0.0)	0.0 (0.0)
HC30 Management & referrals for fever (IMAI)	$6.83	31.0%	$2.12	0.0 (0.0)	0.0 (0.0)	0.0 (0.0)	0.0 (0.0)	0.0 (0.0)	0.0 (0.0)
Noncommunicable diseases (NCDs)				15.9 (2.1)	2.1 (0.0)	0.0 (0.0)	0.0 (0.0)	0.0 (0.0)	3.6 (0.4)
Cardiovascular diseases				11.4 (2.5)	7.0 (0.0)	0.0 (0.0)	0.0 (0.0)	0.0 (0.0)	3.7 (0.5)
HC38 Aspirin for acute myocardial infarction	$0.05	72.4%	$0.04	0.0 (0.0)	0.0 (0.0)	0.0 (0.0)	0.0 (0.0)	0.0 (0.0)	0.0 (0.0)
HC43 Management of ischemic heart disease	$190.17	72.4%	$137.66	13.2 (1.4)	0.0 (0.0)	0.0 (0.0)	0.0 (0.0)	0.0 (0.0)	2.6 (0.3)
HC44 Management of heart failure	$342.48	72.4%	$247.91	21.0 (6.2)	21.0 (0.0)	0.1 (0.0)	0.0 (0.0)	0.0 (0.0)	8.4 (1.2)

Armenia				CHE risk 10% threshold (25% threshold)					
Disease category, disease, intervention	Cost ($)	OOP (%)	OOP ($)	Q1	Q2	Q3	Q4	Q5	Total
Endocrine & metabolic disorders				0.2 (0.0)	0.0 (0.0)	0.0 (0.0)	0.0 (0.0)	0.0 (0.0)	0.0 (0.0)
HC40 Screening & management of diabetes	$92.52	52.4%	$48.44	0.2 (0.0)	0.0 (0.0)	0.0 (0.0)	0.0 (0.0)	0.0 (0.0)	0.0 (0.0)
Mental/behavioral disorders & neurological conditions				24.9 (2.7)	0.0 (0.0)	0.0 (0.0)	0.0 (0.0)	0.0 (0.0)	5.0 (0.5)
HC49 Management of bipolar disorder	$365.39	42.5%	$155.18	68.7 (7.6)	0.0 (0.0)	0.0 (0.0)	0.0 (0.0)	0.0 (0.0)	13.7 (1.5)
HC50 Management of depression	$48.02	42.5%	$20.40	0.3 (0.0)	0.0 (0.0)	0.0 (0.0)	0.0 (0.0)	0.0 (0.0)	0.1 (0.0)
HC51 Management of epilepsy	$53.73	42.5%	$22.82	0.1 (0.0)	0.0 (0.0)	0.0 (0.0)	0.0 (0.0)	0.0 (0.0)	0.0 (0.0)
HC52 Management of schizophrenia	$329.34	42.5%	$139.87	55.1 (5.7)	0.0 (0.0)	0.0 (0.0)	0.0 (0.0)	0.0 (0.0)	11.0 (1.1)
HC66 Psychosocial support & counseling	$21.32	42.5%	$9.05	0.0 (0.0)	0.0 (0.0)	0.0 (0.0)	0.0 (0.0)	0.0 (0.0)	0.0 (0.0)
Other NCDs				0.0 (0.0)	0.0 (0.0)	0.0 (0.0)	0.0 (0.0)	0.0 (0.0)	0.0 (0.0)
HC47 Palliative care	$21.32	87.8%	$18.72	0.0 (0.0)	0.0 (0.0)	0.0 (0.0)	0.0 (0.0)	0.0 (0.0)	0.0 (0.0)
Reproductive health				0.3 (0.0)	0.0 (0.0)	0.0 (0.0)	0.0 (0.0)	0.0 (0.0)	0.1 (0.0)
Family planning				0.0 (0.0)	0.0 (0.0)	0.0 (0.0)	0.0 (0.0)	0.0 (0.0)	0.0 (0.0)
HC4 Contraceptives	$10.32	67.6%	$6.98	0.0 (0.0)	0.0 (0.0)	0.0 (0.0)	0.0 (0.0)	0.0 (0.0)	0.0 (0.0)
Maternal conditions				0.5 (0.0)	0.0 (0.0)	0.0 (0.0)	0.0 (0.0)	0.0 (0.0)	0.1 (0.0)
C5 Antenatal tetanus immunization	$0.44	28.0%	$0.12	0.0 (0.0)	0.0 (0.0)	0.0 (0.0)	0.0 (0.0)	0.0 (0.0)	0.0 (0.0)
HC11 Basic emergency newborn & obstetric care	$145.10	28.0%	$40.69	2.8 (0.2)	0.0 (0.0)	0.0 (0.0)	0.0 (0.0)	0.0 (0.0)	0.6 (0.0)
HC2 Post-abortion care	$8.23	28.0%	$2.31	0.0 (0.0)	0.0 (0.0)	0.0 (0.0)	0.0 (0.0)	0.0 (0.0)	0.0 (0.0)
HC3 Treatment of premature membrane rupture	$4.66	28.0%	$1.31	0.0 (0.0)	0.0 (0.0)	0.0 (0.0)	0.0 (0.0)	0.0 (0.0)	0.0 (0.0)
HC5 Kangaroo mother care counseling	$4.61	28.0%	$1.29	0.0 (0.0)	0.0 (0.0)	0.0 (0.0)	0.0 (0.0)	0.0 (0.0)	0.0 (0.0)
HC7 Medical abortion	$5.52	28.0%	$1.55	0.0 (0.0)	0.0 (0.0)	0.0 (0.0)	0.0 (0.0)	0.0 (0.0)	0.0 (0.0)
Perinatal conditions				0.0 (0.0)	0.0 (0.0)	0.0 (0.0)	0.0 (0.0)	0.0 (0.0)	0.0 (0.0)
C13 Cotrimoxazole for HIV-exposed children	$11.95	0.0%	$0.01	0.0 (0.0)	0.0 (0.0)	0.0 (0.0)	0.0 (0.0)	0.0 (0.0)	0.0 (0.0)
HC1 Antibiotics for neonatal pneumonia	$6.61	0.0%	$0.00	0.0 (0.0)	0.0 (0.0)	0.0 (0.0)	0.0 (0.0)	0.0 (0.0)	0.0 (0.0)
HC6 Neonatal sepsis, pneumonia, meningitis	$3.51	0.0%	$0.00	0.0 (0.0)	0.0 (0.0)	0.0 (0.0)	0.0 (0.0)	0.0 (0.0)	0.0 (0.0)

Table B.3. Full model results: Benin.

Benin				CHE risk 10% threshold (25% threshold)					
Disease category, disease, intervention	Cost ($)	OOP (%)	OOP ($)	Q1	Q2	Q3	Q4	Q5	Total
Childhood health				0.0 (0.0)	0.0 (0.0)	0.0 (0.0)	0.0 (0.0)	0.0 (0.0)	0.0 (0.0)
Childhood health				0.0 (0.0)	0.0 (0.0)	0.0 (0.0)	0.0 (0.0)	0.0 (0.0)	0.0 (0.0)
HC42 Acute pharyngitis treatment	$0.24	42.3%	$0.10	0.0 (0.0)	0.0 (0.0)	0.0 (0.0)	0.0 (0.0)	0.0 (0.0)	0.0 (0.0)
Infectious & parasitic diseases				0.7 (0.1)	0.0 (0.0)	0.0 (0.0)	0.0 (0.0)	0.0 (0.0)	0.1 (0.0)
Diarrheal diseases				0.4 (0.0)	0.0 (0.0)	0.0 (0.0)	0.0 (0.0)	0.0 (0.0)	0.1 (0.0)
HC12 Diagnosis & treatment of infections (IMCI)	$10.29	65.9%	$6.79	0.4 (0.0)	0.0 (0.0)	0.0 (0.0)	0.0 (0.0)	0.0 (0.0)	0.1 (0.0)
HIV/AIDS & other sexually transmitted diseases				1.3 (0.1)	0.0 (0.0)	0.0 (0.0)	0.0 (0.0)	0.0 (0.0)	0.3 (0.0)
HC13 ART & viral load monitoring	$121.57	4.8%	$5.89	0.1 (0.0)	0.0 (0.0)	0.0 (0.0)	0.0 (0.0)	0.0 (0.0)	0.0 (0.0)
HC17 Syndromic management of STI	$10.69	4.8%	$0.52	0.0 (0.0)	0.0 (0.0)	0.0 (0.0)	0.0 (0.0)	0.0 (0.0)	0.0 (0.0)
HC23 HIV, STIs, hepatitis testing & counseling	$6.08	4.8%	$0.29	0.0 (0.0)	0.0 (0.0)	0.0 (0.0)	0.0 (0.0)	0.0 (0.0)	0.0 (0.0)

Benin				CHE risk<br>10% threshold (25% threshold)					
Disease category, disease, intervention	Cost ($)	OOP (%)	OOP ($)	Q1	Q2	Q3	Q4	Q5	Total
HC8 HIV & syphilis PMTCT	$313.51	4.8%	$15.18	5.1 (0.5)	0.0 (0.0)	0.0 (0.0)	0.0 (0.0)	0.0 (0.0)	1.0 (0.1)
Malaria				0.0 (0.0)	0.0 (0.0)	0.0 (0.0)	0.0 (0.0)	0.0 (0.0)	0.0 (0.0)
C7 Intermittent preventive treatment (pregnancy)	$1.02	42.6%	$0.44	0.0 (0.0)	0.0 (0.0)	0.0 (0.0)	0.0 (0.0)	0.0 (0.0)	0.0 (0.0)
Tuberculosis				0.0 (0.0)	0.0 (0.0)	0.0 (0.0)	0.0 (0.0)	0.0 (0.0)	0.0 (0.0)
HC27 Diagnosis & treatment of TB	$175.65	0.3%	$0.53	0.0 (0.0)	0.0 (0.0)	0.0 (0.0)	0.0 (0.0)	0.0 (0.0)	0.0 (0.0)
Other infectious & parasitic diseases				0.1 (0.0)	0.0 (0.0)	0.0 (0.0)	0.0 (0.0)	0.0 (0.0)	0.0 (0.0)
HC30 Management & referrals for fever (IMAI)	$6.83	48.4%	$3.31	0.1 (0.0)	0.0 (0.0)	0.0 (0.0)	0.0 (0.0)	0.0 (0.0)	0.0 (0.0)
Noncommunicable diseases (NCDs)				12.8 (10.4)	12.9 (9.0)	13.3 (0.0)	15.0 (0.0)	6.2 (0.0)	12.0 (3.9)
Cardiovascular diseases				2.1 (1.9)	2.7 (1.3)	2.8 (0.1)	1.8 (0.0)	1.1 (0.0)	2.1 (0.7)
HC38 Aspirin for acute myocardial infarction	$0.05	59.8%	$0.03	0.0 (0.0)	0.0 (0.0)	0.0 (0.0)	0.0 (0.0)	0.0 (0.0)	0.0 (0.0)
HC43 Management of ischemic heart disease	$190.17	59.8%	$113.79	3.2 (2.5)	4.0 (0.0)	4.2 (0.0)	0.4 (0.0)	0.0 (0.0)	2.4 (0.5)
HC44 Management of heart failure	$342.48	59.8%	$204.92	3.1 (3.1)	4.0 (4.0)	4.3 (0.3)	4.9 (0.0)	3.3 (0.0)	3.9 (1.5)
Endocrine & metabolic disorders				9.0 (1.7)	2.7 (0.0)	0.0 (0.0)	0.0 (0.0)	0.0 (0.0)	2.3 (0.3)
HC40 Screening & management of diabetes	$92.52	63.7%	$58.90	9.0 (1.7)	2.7 (0.0)	0.0 (0.0)	0.0 (0.0)	0.0 (0.0)	2.3 (0.3)
Mental/behavioral disorders & neurological conditions				22.5 (19.3)	23.7 (17.3)	25.0 (0.0)	28.9 (0.0)	11.7 (0.0)	22.4 (7.3)
HC49 Management of bipolar disorder	$365.39	52.2%	$190.86	47.3 (47.3)	59.2 (51.1)	62.5 (0.1)	72.3 (0.0)	38.7 (0.0)	56.0 (19.7)
HC50 Management of depression	$48.02	52.2%	$25.09	10.7 (1.3)	0.0 (0.0)	0.0 (0.0)	0.0 (0.0)	0.0 (0.0)	2.1 (0.3)
HC51 Management of epilepsy	$53.73	52.2%	$28.07	5.8 (0.7)	0.0 (0.0)	0.0 (0.0)	0.0 (0.0)	0.0 (0.0)	1.2 (0.1)
HC52 Management of schizophrenia	$329.34	52.2%	$172.03	47.2 (47.2)	59.1 (35.2)	62.2 (0.0)	72.2 (0.0)	20.0 (0.0)	52.1 (16.5)
HC66 Psychosocial support & counseling	$21.32	52.2%	$11.14	1.6 (0.2)	0.0 (0.0)	0.0 (0.0)	0.0 (0.0)	0.0 (0.0)	0.3 (0.0)
Other NCDs				0.0 (0.0)	0.0 (0.0)	0.0 (0.0)	0.0 (0.0)	0.0 (0.0)	0.0 (0.0)
HC47 Palliative care	$21.32	37.2%	$7.94	0.0 (0.0)	0.0 (0.0)	0.0 (0.0)	0.0 (0.0)	0.0 (0.0)	0.0 (0.0)
Reproductive health				5.8 (2.0)	6.4 (0.0)	0.0 (0.0)	0.0 (0.0)	0.0 (0.0)	2.4 (0.4)
Family planning				0.0 (0.0)	0.0 (0.0)	0.0 (0.0)	0.0 (0.0)	0.0 (0.0)	0.0 (0.0)
HC4 Contraceptives	$10.32	22.9%	$2.36	0.0 (0.0)	0.0 (0.0)	0.0 (0.0)	0.0 (0.0)	0.0 (0.0)	0.0 (0.0)
Maternal conditions				9.6 (3.3)	10.7 (0.0)	0.0 (0.0)	0.0 (0.0)	0.0 (0.0)	4.1 (0.7)
C5 Antenatal tetanus immunization	$0.44	52.6%	$0.23	0.0 (0.0)	0.0 (0.0)	0.0 (0.0)	0.0 (0.0)	0.0 (0.0)	0.0 (0.0)
HC11 Basic emergency newborn & obstetric care	$145.10	52.6%	$76.26	57.3 (19.7)	64.2 (0.0)	0.1 (0.0)	0.0 (0.0)	0.0 (0.0)	24.3 (3.9)
HC2 Post-abortion care	$8.23	52.6%	$4.33	0.2 (0.0)	0.0 (0.0)	0.0 (0.0)	0.0 (0.0)	0.0 (0.0)	0.0 (0.0)
HC3 Treatment of premature membrane rupture	$4.66	52.6%	$2.45	0.0 (0.0)	0.0 (0.0)	0.0 (0.0)	0.0 (0.0)	0.0 (0.0)	0.0 (0.0)
HC5 Kangaroo mother care counseling	$4.61	52.6%	$2.42	0.0 (0.0)	0.0 (0.0)	0.0 (0.0)	0.0 (0.0)	0.0 (0.0)	0.0 (0.0)
HC7 Medical abortion	$5.52	52.6%	$2.90	0.1 (0.0)	0.0 (0.0)	0.0 (0.0)	0.0 (0.0)	0.0 (0.0)	0.0 (0.0)
Perinatal conditions				0.0 (0.0)	0.0 (0.0)	0.0 (0.0)	0.0 (0.0)	0.0 (0.0)	0.0 (0.0)
C13 Cotrimoxazole for HIV-exposed children	$11.95	53.1%	$6.34	0.1 (0.0)	0.0 (0.0)	0.0 (0.0)	0.0 (0.0)	0.0 (0.0)	0.0 (0.0)
HC1 Antibiotics for neonatal pneumonia	$6.61	53.1%	$3.51	0.0 (0.0)	0.0 (0.0)	0.0 (0.0)	0.0 (0.0)	0.0 (0.0)	0.0 (0.0)
HC6 Neonatal sepsis, pneumonia, meningitis	$3.51	53.1%	$1.86	0.0 (0.0)	0.0 (0.0)	0.0 (0.0)	0.0 (0.0)	0.0 (0.0)	0.0 (0.0)

Table B.4. Full model results: Burkina Faso.

**Burkina Faso**				**CHE risk** 10% threshold (25% threshold)					
Disease category, disease, intervention	Cost ($)	OOP (%)	OOP ($)	Q1	Q2	Q3	Q4	Q5	Total
Childhood health				0.0 (0.0)	0.0 (0.0)	0.0 (0.0)	0.0 (0.0)	0.0 (0.0)	0.0 (0.0)
Childhood health				0.0 (0.0)	0.0 (0.0)	0.0 (0.0)	0.0 (0.0)	0.0 (0.0)	0.0 (0.0)
HC42 Acute pharyngitis treatment	$0.17	42.3%	$0.07	0.0 (0.0)	0.0 (0.0)	0.0 (0.0)	0.0 (0.0)	0.0 (0.0)	0.0 (0.0)
Infectious & parasitic diseases				5.3 (3.8)	5.5 (0.0)	4.0 (0.0)	0.0 (0.0)	0.0 (0.0)	3.0 (0.8)
Diarrheal diseases				1.1 (0.2)	0.0 (0.0)	0.0 (0.0)	0.0 (0.0)	0.0 (0.0)	0.2 (0.0)
HC12 Diagnosis & treatment of infections (IMCI)	$4.79	65.9%	$3.16	1.1 (0.2)	0.0 (0.0)	0.0 (0.0)	0.0 (0.0)	0.0 (0.0)	0.2 (0.0)
HIV/AIDS & other sexually transmitted diseases				0.7 (0.1)	0.0 (0.0)	0.0 (0.0)	0.0 (0.0)	0.0 (0.0)	0.1 (0.0)
HC13 ART & viral load monitoring	$71.45	2.7%	$1.93	0.1 (0.0)	0.0 (0.0)	0.0 (0.0)	0.0 (0.0)	0.0 (0.0)	0.0 (0.0)
HC17 Syndromic management of STI	$5.67	2.7%	$0.15	0.0 (0.0)	0.0 (0.0)	0.0 (0.0)	0.0 (0.0)	0.0 (0.0)	0.0 (0.0)
HC23 HIV, STIs, hepatitis testing & counseling	$4.31	2.7%	$0.12	0.0 (0.0)	0.0 (0.0)	0.0 (0.0)	0.0 (0.0)	0.0 (0.0)	0.0 (0.0)
HC8 HIV & syphilis PMTCT	$176.35	2.7%	$4.77	2.8 (0.4)	0.0 (0.0)	0.0 (0.0)	0.0 (0.0)	0.0 (0.0)	0.6 (0.1)
Malaria				0.0 (0.0)	0.0 (0.0)	0.0 (0.0)	0.0 (0.0)	0.0 (0.0)	0.0 (0.0)
C7 Intermittent preventive treatment (pregnancy)	$0.45	26.7%	$0.12	0.0 (0.0)	0.0 (0.0)	0.0 (0.0)	0.0 (0.0)	0.0 (0.0)	0.0 (0.0)
Tuberculosis				38.2 (29.3)	43.9 (0.0)	31.8 (0.0)	0.0 (0.0)	0.0 (0.0)	22.8 (5.9)
HC27 Diagnosis & treatment of TB	$135.09	40.3%	$54.44	38.2 (29.3)	43.9 (0.0)	31.8 (0.0)	0.0 (0.0)	0.0 (0.0)	22.8 (5.9)
Other infectious & parasitic diseases				0.3 (0.0)	0.0 (0.0)	0.0 (0.0)	0.0 (0.0)	0.0 (0.0)	0.1 (0.0)
HC30 Management & referrals for fever (IMAI)	$3.11	48.4%	$1.51	0.3 (0.0)	0.0 (0.0)	0.0 (0.0)	0.0 (0.0)	0.0 (0.0)	0.1 (0.0)
Noncommunicable diseases (NCDs)				20.6 (13.7)	19.0 (6.5)	12.6 (2.1)	9.8 (0.0)	4.3 (0.0)	13.2 (4.5)
Cardiovascular diseases				12.2 (10.2)	14.0 (7.0)	10.9 (6.9)	8.9 (0.1)	9.3 (0.0)	11.1 (4.8)
HC38 Aspirin for acute myocardial infarction	$0.03	59.8%	$0.02	0.0 (0.0)	0.0 (0.0)	0.0 (0.0)	0.0 (0.0)	0.0 (0.0)	0.0 (0.0)
HC43 Management of ischemic heart disease	$83.97	59.8%	$50.24	18.3 (12.3)	21.1 (0.0)	10.3 (0.0)	0.0 (0.0)	0.0 (0.0)	9.9 (2.5)
HC44 Management of heart failure	$249.96	59.8%	$149.56	18.3 (18.3)	20.9 (20.9)	22.4 (20.8)	26.8 (0.2)	27.8 (0.0)	23.3 (12.1)
Endocrine & metabolic disorders				8.1 (3.8)	8.4 (0.0)	0.1 (0.0)	0.0 (0.0)	0.0 (0.0)	3.3 (0.8)
HC40 Screening & management of diabetes	$64.16	63.7%	$40.85	8.1 (3.8)	8.4 (0.0)	0.1 (0.0)	0.0 (0.0)	0.0 (0.0)	3.3 (0.8)
Mental/behavioral disorders & neurological conditions				32.1 (20.4)	28.0 (8.8)	18.6 (0.0)	14.3 (0.0)	3.0 (0.0)	19.2 (5.9)
HC49 Management of bipolar disorder	$184.57	52.2%	$96.41	48.7 (48.7)	56.1 (44.1)	60.1 (0.0)	71.4 (0.0)	14.8 (0.0)	50.2 (18.6)
HC50 Management of depression	$16.11	52.2%	$8.42	6.8 (1.1)	0.0 (0.0)	0.0 (0.0)	0.0 (0.0)	0.0 (0.0)	1.4 (0.2)
HC51 Management of epilepsy	$27.53	52.2%	$14.38	7.8 (1.4)	0.0 (0.0)	0.0 (0.0)	0.0 (0.0)	0.0 (0.0)	1.6 (0.3)
HC52 Management of schizophrenia	$99.43	52.2%	$51.94	48.8 (34.6)	56.2 (0.0)	32.7 (0.0)	0.0 (0.0)	0.0 (0.0)	27.5 (6.9)
HC66 Psychosocial support & counseling	$64.63	52.2%	$33.76	48.6 (16.4)	27.6 (0.0)	0.0 (0.0)	0.0 (0.0)	0.0 (0.0)	15.2 (3.3)
Other NCDs				0.1 (0.0)	0.0 (0.0)	0.0 (0.0)	0.0 (0.0)	0.0 (0.0)	0.0 (0.0)
HC47 Palliative care	$64.63	37.2%	$24.06	0.1 (0.0)	0.0 (0.0)	0.0 (0.0)	0.0 (0.0)	0.0 (0.0)	0.0 (0.0)
Reproductive health				4.2 (1.5)	3.2 (0.0)	0.0 (0.0)	0.0 (0.0)	0.0 (0.0)	1.5 (0.3)
Family planning				0.0 (0.0)	0.0 (0.0)	0.0 (0.0)	0.0 (0.0)	0.0 (0.0)	0.0 (0.0)
HC4 Contraceptives	$4.97	3.3%	$0.17	0.0 (0.0)	0.0 (0.0)	0.0 (0.0)	0.0 (0.0)	0.0 (0.0)	0.0 (0.0)
Maternal conditions				6.7 (2.5)	5.3 (0.0)	0.0 (0.0)	0.0 (0.0)	0.0 (0.0)	2.4 (0.5)
C5 Antenatal tetanus immunization	$0.39	52.6%	$0.20	0.0 (0.0)	0.0 (0.0)	0.0 (0.0)	0.0 (0.0)	0.0 (0.0)	0.0 (0.0)
HC11 Basic emergency newborn & obstetric care	$69.14	52.6%	$36.34	38.3 (14.7)	31.6 (0.0)	0.0 (0.0)	0.0 (0.0)	0.0 (0.0)	14.0 (2.9)

Burkina Faso				CHE risk<br>10% threshold (25% threshold)					
Disease category, disease, intervention	Cost ($)	OOP (%)	OOP ($)	Q1	Q2	Q3	Q4	Q5	Total
HC2 Post-abortion care	$4.54	52.6%	$2.39	0.4 (0.1)	0.0 (0.0)	0.0 (0.0)	0.0 (0.0)	0.0 (0.0)	0.1 (0.0)
HC3 Treatment of premature membrane rupture	$4.04	52.6%	$2.12	0.4 (0.1)	0.0 (0.0)	0.0 (0.0)	0.0 (0.0)	0.0 (0.0)	0.1 (0.0)
HC5 Kangaroo mother care counseling	$2.33	52.6%	$1.22	0.1 (0.0)	0.0 (0.0)	0.0 (0.0)	0.0 (0.0)	0.0 (0.0)	0.0 (0.0)
HC7 Medical abortion	$4.81	52.6%	$2.53	1.0 (0.2)	0.0 (0.0)	0.0 (0.0)	0.0 (0.0)	0.0 (0.0)	0.2 (0.0)
Perinatal conditions				0.4 (0.1)	0.0 (0.0)	0.0 (0.0)	0.0 (0.0)	0.0 (0.0)	0.1 (0.0)
C13 Cotrimoxazole for HIV-exposed children	$6.55	53.1%	$3.48	0.6 (0.1)	0.0 (0.0)	0.0 (0.0)	0.0 (0.0)	0.0 (0.0)	0.1 (0.0)
HC1 Antibiotics for neonatal pneumonia	$6.22	53.1%	$3.30	0.6 (0.1)	0.0 (0.0)	0.0 (0.0)	0.0 (0.0)	0.0 (0.0)	0.1 (0.0)
HC6 Neonatal sepsis, pneumonia, meningitis	$2.66	53.1%	$1.41	0.1 (0.0)	0.0 (0.0)	0.0 (0.0)	0.0 (0.0)	0.0 (0.0)	0.0 (0.0)

Table B.5. Full model results: Burundi.

Burundi				CHE risk<br>10% threshold (25% threshold)					
Disease category, disease, intervention	Cost ($)	OOP (%)	OOP ($)	Q1	Q2	Q3	Q4	Q5	Total
Childhood health				0.0 (0.0)	0.0 (0.0)	0.0 (0.0)	0.0 (0.0)	0.0 (0.0)	0.0 (0.0)
Childhood health				0.0 (0.0)	0.0 (0.0)	0.0 (0.0)	0.0 (0.0)	0.0 (0.0)	0.0 (0.0)
HC42 Acute pharyngitis treatment	$0.17	39.3%	$0.07	0.0 (0.0)	0.0 (0.0)	0.0 (0.0)	0.0 (0.0)	0.0 (0.0)	0.0 (0.0)
Infectious & parasitic diseases				5.3 (0.8)	0.0 (0.0)	0.0 (0.0)	0.0 (0.0)	0.0 (0.0)	1.1 (0.2)
Diarrheal diseases				2.1 (0.2)	0.0 (0.0)	0.0 (0.0)	0.0 (0.0)	0.0 (0.0)	0.4 (0.0)
HC12 Diagnosis & treatment of infections (IMCI)	$4.79	48.3%	$2.32	2.1 (0.2)	0.0 (0.0)	0.0 (0.0)	0.0 (0.0)	0.0 (0.0)	0.4 (0.0)
HIV/AIDS & other sexually transmitted diseases				0.0 (0.0)	0.0 (0.0)	0.0 (0.0)	0.0 (0.0)	0.0 (0.0)	0.0 (0.0)
HC13 ART & viral load monitoring	$71.45	0.2%	$0.11	0.0 (0.0)	0.0 (0.0)	0.0 (0.0)	0.0 (0.0)	0.0 (0.0)	0.0 (0.0)
HC17 Syndromic management of STI	$5.67	0.2%	$0.01	0.0 (0.0)	0.0 (0.0)	0.0 (0.0)	0.0 (0.0)	0.0 (0.0)	0.0 (0.0)
HC23 HIV, STIs, hepatitis testing & counseling	$4.31	0.2%	$0.01	0.0 (0.0)	0.0 (0.0)	0.0 (0.0)	0.0 (0.0)	0.0 (0.0)	0.0 (0.0)
HC8 HIV & syphilis PMTCT	$176.35	0.2%	$0.28	0.0 (0.0)	0.0 (0.0)	0.0 (0.0)	0.0 (0.0)	0.0 (0.0)	0.0 (0.0)
Malaria				0.0 (0.0)	0.0 (0.0)	0.0 (0.0)	0.0 (0.0)	0.0 (0.0)	0.0 (0.0)
C7 Intermittent preventive treatment (pregnancy)	$0.45	35.9%	$0.16	0.0 (0.0)	0.0 (0.0)	0.0 (0.0)	0.0 (0.0)	0.0 (0.0)	0.0 (0.0)
Tuberculosis				39.9 (5.8)	0.0 (0.0)	0.0 (0.0)	0.0 (0.0)	0.0 (0.0)	8.0 (1.2)
HC27 Diagnosis & treatment of TB	$135.09	7.2%	$9.71	39.9 (5.8)	0.0 (0.0)	0.0 (0.0)	0.0 (0.0)	0.0 (0.0)	8.0 (1.2)
Other infectious & parasitic diseases				0.1 (0.0)	0.0 (0.0)	0.0 (0.0)	0.0 (0.0)	0.0 (0.0)	0.0 (0.0)
HC30 Management & referrals for fever (IMAI)	$3.11	22.6%	$0.70	0.1 (0.0)	0.0 (0.0)	0.0 (0.0)	0.0 (0.0)	0.0 (0.0)	0.0 (0.0)
Noncommunicable diseases (NCDs)				7.9 (6.0)	6.8 (4.2)	6.0 (2.7)	5.2 (2.0)	4.3 (1.8)	6.0 (3.3)
Cardiovascular diseases				9.2 (9.2)	9.6 (9.6)	9.9 (6.5)	10.3 (5.1)	10.5 (5.4)	9.9 (7.2)
HC38 Aspirin for acute myocardial infarction	$0.03	56.5%	$0.02	0.0 (0.0)	0.0 (0.0)	0.0 (0.0)	0.0 (0.0)	0.0 (0.0)	0.0 (0.0)
HC43 Management of ischemic heart disease	$83.97	56.5%	$47.48	13.8 (13.8)	14.4 (14.4)	14.8 (4.8)	15.5 (0.0)	12.7 (0.0)	14.2 (6.6)
HC44 Management of heart failure	$249.96	56.5%	$141.32	13.9 (13.9)	14.3 (14.3)	14.8 (14.8)	15.3 (15.3)	18.9 (16.1)	15.5 (14.9)
Endocrine & metabolic disorders				4.7 (4.7)	4.9 (2.3)	5.0 (0.0)	5.2 (0.0)	0.6 (0.0)	4.1 (1.4)
HC40 Screening & management of diabetes	$64.16	53.9%	$34.58	4.7 (4.7)	4.9 (2.3)	5.0 (0.0)	5.2 (0.0)	0.6 (0.0)	4.1 (1.4)
Mental/behavioral disorders & neurological conditions				7.3 (3.8)	4.8 (2.1)	2.9 (1.4)	3.0 (1.0)	2.1 (0.3)	4.0 (1.7)
HC49 Management of bipolar disorder	$184.57	50.5%	$93.19	4.4 (4.4)	4.6 (4.6)	4.8 (4.8)	5.1 (5.1)	6.1 (1.5)	5.0 (4.1)

Burundi				CHE risk 10% threshold (25% threshold)					
Disease category, disease, intervention	Cost ($)	OOP (%)	OOP ($)	Q1	Q2	Q3	Q4	Q5	Total
HC50 Management of depression	$16.11	50.5%	$8.14	2.6 (0.4)	0.0 (0.0)	0.0 (0.0)	0.0 (0.0)	0.0 (0.0)	0.5 (0.1)
HC51 Management of epilepsy	$27.53	50.5%	$13.90	20.3 (5.2)	10.1 (0.0)	0.0 (0.0)	0.0 (0.0)	0.0 (0.0)	6.1 (1.0)
HC52 Management of schizophrenia	$99.43	50.5%	$50.20	4.6 (4.6)	4.6 (4.6)	4.9 (2.4)	5.1 (0.0)	4.6 (0.0)	4.7 (2.3)
HC66 Psychosocial support & counseling	$64.63	50.5%	$32.63	4.6 (4.6)	4.6 (1.6)	4.8 (0.0)	4.8 (0.0)	0.0 (0.0)	3.8 (1.2)
Other NCDs				10.0 (8.2)	10.3 (0.0)	10.5 (0.0)	1.4 (0.0)	0.0 (0.0)	6.4 (1.6)
HC47 Palliative care	$64.63	38.0%	$24.59	10.0 (8.2)	10.3 (0.0)	10.5 (0.0)	1.4 (0.0)	0.0 (0.0)	6.4 (1.6)
Reproductive health				3.6 (0.5)	0.0 (0.0)	0.0 (0.0)	0.0 (0.0)	0.0 (0.0)	0.7 (0.1)
Family planning				0.0 (0.0)	0.0 (0.0)	0.0 (0.0)	0.0 (0.0)	0.0 (0.0)	0.0 (0.0)
HC4 Contraceptives	$4.97	0.3%	$0.01	0.0 (0.0)	0.0 (0.0)	0.0 (0.0)	0.0 (0.0)	0.0 (0.0)	0.0 (0.0)
Maternal conditions				5.6 (0.7)	0.0 (0.0)	0.0 (0.0)	0.0 (0.0)	0.0 (0.0)	1.1 (0.1)
C5 Antenatal tetanus immunization	$0.39	10.2%	$0.04	0.0 (0.0)	0.0 (0.0)	0.0 (0.0)	0.0 (0.0)	0.0 (0.0)	0.0 (0.0)
HC11 Basic emergency newborn & obstetric care	$69.14	10.2%	$7.04	33.7 (4.4)	0.0 (0.0)	0.0 (0.0)	0.0 (0.0)	0.0 (0.0)	6.7 (0.9)
HC2 Post-abortion care	$4.54	10.2%	$0.46	0.1 (0.0)	0.0 (0.0)	0.0 (0.0)	0.0 (0.0)	0.0 (0.0)	0.0 (0.0)
HC3 Treatment of premature membrane rupture	$4.04	10.2%	$0.41	0.0 (0.0)	0.0 (0.0)	0.0 (0.0)	0.0 (0.0)	0.0 (0.0)	0.0 (0.0)
HC5 Kangaroo mother care counseling	$2.33	10.2%	$0.24	0.0 (0.0)	0.0 (0.0)	0.0 (0.0)	0.0 (0.0)	0.0 (0.0)	0.0 (0.0)
HC7 Medical abortion	$4.81	10.2%	$0.49	0.1 (0.0)	0.0 (0.0)	0.0 (0.0)	0.0 (0.0)	0.0 (0.0)	0.0 (0.0)
Perinatal conditions				0.7 (0.1)	0.0 (0.0)	0.0 (0.0)	0.0 (0.0)	0.0 (0.0)	0.1 (0.0)
C13 Cotrimoxazole for HIV-exposed children	$6.55	38.6%	$2.53	1.0 (0.1)	0.0 (0.0)	0.0 (0.0)	0.0 (0.0)	0.0 (0.0)	0.2 (0.0)
HC1 Antibiotics for neonatal pneumonia	$6.22	38.6%	$2.40	0.9 (0.1)	0.0 (0.0)	0.0 (0.0)	0.0 (0.0)	0.0 (0.0)	0.2 (0.0)
HC6 Neonatal sepsis, pneumonia, meningitis	$2.66	38.6%	$1.03	0.1 (0.0)	0.0 (0.0)	0.0 (0.0)	0.0 (0.0)	0.0 (0.0)	0.0 (0.0)

Table B.6. Full model results: Cabo Verde.

Cabo Verde				CHE risk 10% threshold (25% threshold)					
Disease category, disease, intervention	Cost ($)	OOP (%)	OOP ($)	Q1	Q2	Q3	Q4	Q5	Total
Childhood health				0.0 (0.0)	0.0 (0.0)	0.0 (0.0)	0.0 (0.0)	0.0 (0.0)	0.0 (0.0)
Childhood health				0.0 (0.0)	0.0 (0.0)	0.0 (0.0)	0.0 (0.0)	0.0 (0.0)	0.0 (0.0)
HC42 Acute pharyngitis treatment	$0.24	42.3%	$0.10	0.0 (0.0)	0.0 (0.0)	0.0 (0.0)	0.0 (0.0)	0.0 (0.0)	0.0 (0.0)
Infectious & parasitic diseases				4.9 (0.7)	0.0 (0.0)	0.0 (0.0)	0.0 (0.0)	0.0 (0.0)	1.0 (0.1)
Diarrheal diseases				0.1 (0.0)	0.0 (0.0)	0.0 (0.0)	0.0 (0.0)	0.0 (0.0)	0.0 (0.0)
HC12 Diagnosis & treatment of infections (IMCI)	$10.29	53.4%	$5.49	0.1 (0.0)	0.0 (0.0)	0.0 (0.0)	0.0 (0.0)	0.0 (0.0)	0.0 (0.0)
HIV/AIDS & other sexually transmitted diseases				4.6 (0.7)	0.0 (0.0)	0.0 (0.0)	0.0 (0.0)	0.0 (0.0)	0.9 (0.1)
HC13 ART & viral load monitoring	$121.57	22.9%	$27.81	0.8 (0.1)	0.0 (0.0)	0.0 (0.0)	0.0 (0.0)	0.0 (0.0)	0.2 (0.0)
HC17 Syndromic management of STI	$10.69	22.9%	$2.44	0.0 (0.0)	0.0 (0.0)	0.0 (0.0)	0.0 (0.0)	0.0 (0.0)	0.0 (0.0)
HC23 HIV, STIs, hepatitis testing & counseling	$6.08	22.9%	$1.39	0.0 (0.0)	0.0 (0.0)	0.0 (0.0)	0.0 (0.0)	0.0 (0.0)	0.0 (0.0)
HC8 HIV & syphilis PMTCT	$313.51	22.9%	$71.72	17.5 (2.6)	0.0 (0.0)	0.0 (0.0)	0.0 (0.0)	0.0 (0.0)	3.5 (0.5)
Malaria				0.0 (0.0)	0.0 (0.0)	0.0 (0.0)	0.0 (0.0)	0.0 (0.0)	0.0 (0.0)
C7 Intermittent preventive treatment (pregnancy)	$1.02	53.2%	$0.54	0.0 (0.0)	0.0 (0.0)	0.0 (0.0)	0.0 (0.0)	0.0 (0.0)	0.0 (0.0)
Tuberculosis				21.0 (3.1)	0.0 (0.0)	0.0 (0.0)	0.0 (0.0)	0.0 (0.0)	4.2 (0.6)

**Cabo Verde**				**CHE risk**<br>10% threshold (25% threshold)					
Disease category, disease, intervention	Cost ($)	OOP (%)	OOP ($)	Q1	Q2	Q3	Q4	Q5	Total
HC27 Diagnosis & treatment of TB	$175.65	40.3%	$70.79	21.0 (3.1)	0.0 (0.0)	0.0 (0.0)	0.0 (0.0)	0.0 (0.0)	4.2 (0.6)
Other infectious & parasitic diseases				0.0 (0.0)	0.0 (0.0)	0.0 (0.0)	0.0 (0.0)	0.0 (0.0)	0.0 (0.0)
HC30 Management & referrals for fever (IMAI)	$6.83	49.8%	$3.40	0.0 (0.0)	0.0 (0.0)	0.0 (0.0)	0.0 (0.0)	0.0 (0.0)	0.0 (0.0)
Noncommunicable diseases (NCDs)				0.5 (0.1)	0.0 (0.0)	0.0 (0.0)	0.0 (0.0)	0.0 (0.0)	0.1 (0.0)
Cardiovascular diseases				0.9 (0.1)	0.0 (0.0)	0.0 (0.0)	0.0 (0.0)	0.0 (0.0)	0.2 (0.0)
HC38 Aspirin for acute myocardial infarction	$0.05	11.9%	$0.01	0.0 (0.0)	0.0 (0.0)	0.0 (0.0)	0.0 (0.0)	0.0 (0.0)	0.0 (0.0)
HC43 Management of ischemic heart disease	$190.17	11.9%	$22.58	0.6 (0.1)	0.0 (0.0)	0.0 (0.0)	0.0 (0.0)	0.0 (0.0)	0.1 (0.0)
HC44 Management of heart failure	$342.48	11.9%	$40.67	2.1 (0.3)	0.0 (0.0)	0.0 (0.0)	0.0 (0.0)	0.0 (0.0)	0.4 (0.1)
Endocrine & metabolic disorders				0.1 (0.0)	0.0 (0.0)	0.0 (0.0)	0.0 (0.0)	0.0 (0.0)	0.0 (0.0)
HC40 Screening & management of diabetes	$92.52	16.8%	$15.55	0.1 (0.0)	0.0 (0.0)	0.0 (0.0)	0.0 (0.0)	0.0 (0.0)	0.0 (0.0)
Mental/behavioral disorders & neurological conditions				0.4 (0.1)	0.0 (0.0)	0.0 (0.0)	0.0 (0.0)	0.0 (0.0)	0.1 (0.0)
HC49 Management of bipolar disorder	$365.39	5.5%	$20.16	1.2 (0.2)	0.0 (0.0)	0.0 (0.0)	0.0 (0.0)	0.0 (0.0)	0.2 (0.0)
HC50 Management of depression	$48.02	5.5%	$2.65	0.0 (0.0)	0.0 (0.0)	0.0 (0.0)	0.0 (0.0)	0.0 (0.0)	0.0 (0.0)
HC51 Management of epilepsy	$53.73	5.5%	$2.96	0.0 (0.0)	0.0 (0.0)	0.0 (0.0)	0.0 (0.0)	0.0 (0.0)	0.0 (0.0)
HC52 Management of schizophrenia	$329.34	5.5%	$18.17	1.0 (0.1)	0.0 (0.0)	0.0 (0.0)	0.0 (0.0)	0.0 (0.0)	0.2 (0.0)
HC66 Psychosocial support & counseling	$21.32	5.5%	$1.18	0.0 (0.0)	0.0 (0.0)	0.0 (0.0)	0.0 (0.0)	0.0 (0.0)	0.0 (0.0)
Other NCDs				0.0 (0.0)	0.0 (0.0)	0.0 (0.0)	0.0 (0.0)	0.0 (0.0)	0.0 (0.0)
HC47 Palliative care	$21.32	15.0%	$3.20	0.0 (0.0)	0.0 (0.0)	0.0 (0.0)	0.0 (0.0)	0.0 (0.0)	0.0 (0.0)
Reproductive health				0.0 (0.0)	0.0 (0.0)	0.0 (0.0)	0.0 (0.0)	0.0 (0.0)	0.0 (0.0)
Family planning				0.0 (0.0)	0.0 (0.0)	0.0 (0.0)	0.0 (0.0)	0.0 (0.0)	0.0 (0.0)
HC4 Contraceptives	$10.32	22.9%	$2.36	0.0 (0.0)	0.0 (0.0)	0.0 (0.0)	0.0 (0.0)	0.0 (0.0)	0.0 (0.0)
Maternal conditions				0.0 (0.0)	0.0 (0.0)	0.0 (0.0)	0.0 (0.0)	0.0 (0.0)	0.0 (0.0)
C5 Antenatal tetanus immunization	$0.44	5.8%	$0.03	0.0 (0.0)	0.0 (0.0)	0.0 (0.0)	0.0 (0.0)	0.0 (0.0)	0.0 (0.0)
HC11 Basic emergency newborn & obstetric care	$145.10	5.8%	$8.42	0.1 (0.0)	0.0 (0.0)	0.0 (0.0)	0.0 (0.0)	0.0 (0.0)	0.0 (0.0)
HC2 Post-abortion care	$8.23	5.8%	$0.48	0.0 (0.0)	0.0 (0.0)	0.0 (0.0)	0.0 (0.0)	0.0 (0.0)	0.0 (0.0)
HC3 Treatment of premature membrane rupture	$4.66	5.8%	$0.27	0.0 (0.0)	0.0 (0.0)	0.0 (0.0)	0.0 (0.0)	0.0 (0.0)	0.0 (0.0)
HC5 Kangaroo mother care counseling	$4.61	5.8%	$0.27	0.0 (0.0)	0.0 (0.0)	0.0 (0.0)	0.0 (0.0)	0.0 (0.0)	0.0 (0.0)
HC7 Medical abortion	$5.52	5.8%	$0.32	0.0 (0.0)	0.0 (0.0)	0.0 (0.0)	0.0 (0.0)	0.0 (0.0)	0.0 (0.0)
Perinatal conditions				0.0 (0.0)	0.0 (0.0)	0.0 (0.0)	0.0 (0.0)	0.0 (0.0)	0.0 (0.0)
C13 Cotrimoxazole for HIV-exposed children	$11.95	1.2%	$0.14	0.0 (0.0)	0.0 (0.0)	0.0 (0.0)	0.0 (0.0)	0.0 (0.0)	0.0 (0.0)
HC1 Antibiotics for neonatal pneumonia	$6.61	1.2%	$0.08	0.0 (0.0)	0.0 (0.0)	0.0 (0.0)	0.0 (0.0)	0.0 (0.0)	0.0 (0.0)
HC6 Neonatal sepsis, pneumonia, meningitis	$3.51	1.2%	$0.04	0.0 (0.0)	0.0 (0.0)	0.0 (0.0)	0.0 (0.0)	0.0 (0.0)	0.0 (0.0)

Table B.7. Full model results: Cambodia.

**Cambodia**				**CHE risk**<br>10% threshold (25% threshold)					
Disease category, disease, intervention	Cost ($)	OOP (%)	OOP ($)	Q1	Q2	Q3	Q4	Q5	Total
Childhood health				0.0 (0.0)	0.0 (0.0)	0.0 (0.0)	0.0 (0.0)	0.0 (0.0)	0.0 (0.0)
Childhood health				0.0 (0.0)	0.0 (0.0)	0.0 (0.0)	0.0 (0.0)	0.0 (0.0)	0.0 (0.0)

Cambodia				CHE risk 10% threshold (25% threshold)					
Disease category, disease, intervention	Cost ($)	OOP (%)	OOP ($)	Q1	Q2	Q3	Q4	Q5	Total
HC42 Acute pharyngitis treatment	$0.17	39.3%	$0.07	0.0 (0.0)	0.0 (0.0)	0.0 (0.0)	0.0 (0.0)	0.0 (0.0)	0.0 (0.0)
Infectious & parasitic diseases				6.8 (1.0)	0.0 (0.0)	0.0 (0.0)	0.0 (0.0)	0.0 (0.0)	1.4 (0.2)
Diarrheal diseases				0.3 (0.0)	0.0 (0.0)	0.0 (0.0)	0.0 (0.0)	0.0 (0.0)	0.1 (0.0)
HC12 Diagnosis & treatment of infections (IMCI)	$4.79	73.8%	$3.54	0.3 (0.0)	0.0 (0.0)	0.0 (0.0)	0.0 (0.0)	0.0 (0.0)	0.1 (0.0)
HIV/AIDS & other sexually transmitted diseases				0.5 (0.1)	0.0 (0.0)	0.0 (0.0)	0.0 (0.0)	0.0 (0.0)	0.1 (0.0)
HC13 ART & viral load monitoring	$71.45	4.5%	$3.19	0.1 (0.0)	0.0 (0.0)	0.0 (0.0)	0.0 (0.0)	0.0 (0.0)	0.0 (0.0)
HC17 Syndromic management of STI	$5.67	4.5%	$0.25	0.0 (0.0)	0.0 (0.0)	0.0 (0.0)	0.0 (0.0)	0.0 (0.0)	0.0 (0.0)
HC23 HIV, STIs, hepatitis testing & counseling	$4.31	4.5%	$0.19	0.0 (0.0)	0.0 (0.0)	0.0 (0.0)	0.0 (0.0)	0.0 (0.0)	0.0 (0.0)
HC8 HIV & syphilis PMTCT	$176.35	4.5%	$7.87	1.9 (0.2)	0.0 (0.0)	0.0 (0.0)	0.0 (0.0)	0.0 (0.0)	0.4 (0.0)
Malaria				0.0 (0.0)	0.0 (0.0)	0.0 (0.0)	0.0 (0.0)	0.0 (0.0)	0.0 (0.0)
C7 Intermittent preventive treatment (pregnancy)	$0.45	5.1%	$0.02	0.0 (0.0)	0.0 (0.0)	0.0 (0.0)	0.0 (0.0)	0.0 (0.0)	0.0 (0.0)
Tuberculosis				52.1 (7.7)	0.0 (0.0)	0.0 (0.0)	0.0 (0.0)	0.0 (0.0)	10.4 (1.5)
HC27 Diagnosis & treatment of TB	$135.09	30.1%	$40.65	52.1 (7.7)	0.0 (0.0)	0.0 (0.0)	0.0 (0.0)	0.0 (0.0)	10.4 (1.5)
Other infectious & parasitic diseases				0.0 (0.0)	0.0 (0.0)	0.0 (0.0)	0.0 (0.0)	0.0 (0.0)	0.0 (0.0)
HC30 Management & referrals for fever (IMAI)	$3.11	44.7%	$1.39	0.0 (0.0)	0.0 (0.0)	0.0 (0.0)	0.0 (0.0)	0.0 (0.0)	0.0 (0.0)
Noncommunicable diseases (NCDs)				17.4 (10.5)	14.8 (5.3)	9.3 (1.3)	6.6 (0.0)	3.4 (0.0)	10.3 (3.4)
Cardiovascular diseases				13.0 (9.2)	13.4 (6.7)	7.6 (4.2)	7.1 (0.0)	5.8 (0.0)	9.4 (4.0)
HC38 Aspirin for acute myocardial infarction	$0.03	82.2%	$0.02	0.0 (0.0)	0.0 (0.0)	0.0 (0.0)	0.0 (0.0)	0.0 (0.0)	0.0 (0.0)
HC43 Management of ischemic heart disease	$83.97	82.2%	$69.03	19.4 (8.0)	20.2 (0.0)	2.0 (0.0)	0.0 (0.0)	0.0 (0.0)	8.3 (1.6)
HC44 Management of heart failure	$249.96	82.2%	$205.49	19.5 (19.5)	20.2 (20.2)	20.7 (12.7)	21.4 (0.0)	17.3 (0.0)	19.8 (10.5)
Endocrine & metabolic disorders				2.6 (0.5)	0.9 (0.0)	0.0 (0.0)	0.0 (0.0)	0.0 (0.0)	0.7 (0.1)
HC40 Screening & management of diabetes	$64.16	79.2%	$50.81	2.6 (0.5)	0.9 (0.0)	0.0 (0.0)	0.0 (0.0)	0.0 (0.0)	0.7 (0.1)
Mental/behavioral disorders & neurological conditions				24.8 (14.9)	20.6 (6.6)	14.1 (0.0)	8.8 (0.0)	3.4 (0.0)	14.3 (4.3)
HC49 Management of bipolar disorder	$184.57	83.7%	$154.54	40.0 (40.0)	42.1 (33.0)	42.7 (0.0)	44.2 (0.0)	17.0 (0.0)	37.2 (14.6)
HC50 Management of depression	$16.11	83.7%	$13.49	3.4 (0.4)	0.0 (0.0)	0.0 (0.0)	0.0 (0.0)	0.0 (0.0)	0.7 (0.1)
HC51 Management of epilepsy	$27.53	83.7%	$23.05	0.8 (0.1)	0.0 (0.0)	0.0 (0.0)	0.0 (0.0)	0.0 (0.0)	0.2 (0.0)
HC52 Management of schizophrenia	$99.43	83.7%	$83.26	40.0 (24.4)	41.9 (0.0)	27.8 (0.0)	0.0 (0.0)	0.0 (0.0)	21.9 (4.9)
HC66 Psychosocial support & counseling	$64.63	83.7%	$54.12	40.0 (9.8)	19.0 (0.0)	0.0 (0.0)	0.0 (0.0)	0.0 (0.0)	11.8 (2.0)
Other NCDs				8.4 (1.9)	3.4 (0.0)	0.0 (0.0)	0.0 (0.0)	0.0 (0.0)	2.4 (0.4)
HC47 Palliative care	$64.63	81.2%	$52.48	8.4 (1.9)	3.4 (0.0)	0.0 (0.0)	0.0 (0.0)	0.0 (0.0)	2.4 (0.4)
Reproductive health				8.1 (2.2)	5.4 (0.0)	0.0 (0.0)	0.0 (0.0)	0.0 (0.0)	2.7 (0.4)
Family planning				0.1 (0.0)	0.0 (0.0)	0.0 (0.0)	0.0 (0.0)	0.0 (0.0)	0.0 (0.0)
HC4 Contraceptives	$4.97	71.8%	$3.57	0.1 (0.0)	0.0 (0.0)	0.0 (0.0)	0.0 (0.0)	0.0 (0.0)	0.0 (0.0)
Maternal conditions				13.3 (3.7)	9.0 (0.0)	0.0 (0.0)	0.0 (0.0)	0.0 (0.0)	4.5 (0.7)
C5 Antenatal tetanus immunization	$0.39	83.0%	$0.32	0.0 (0.0)	0.0 (0.0)	0.0 (0.0)	0.0 (0.0)	0.0 (0.0)	0.0 (0.0)
HC11 Basic emergency newborn & obstetric care	$69.14	83.0%	$57.40	79.2 (22.2)	54.0 (0.0)	0.0 (0.0)	0.0 (0.0)	0.0 (0.0)	26.6 (4.4)
HC2 Post-abortion care	$4.54	83.0%	$3.77	0.3 (0.0)	0.0 (0.0)	0.0 (0.0)	0.0 (0.0)	0.0 (0.0)	0.1 (0.0)
HC3 Treatment of premature membrane rupture	$4.04	83.0%	$3.35	0.2 (0.0)	0.0 (0.0)	0.0 (0.0)	0.0 (0.0)	0.0 (0.0)	0.0 (0.0)
HC5 Kangaroo mother care counseling	$2.33	83.0%	$1.93	0.1 (0.0)	0.0 (0.0)	0.0 (0.0)	0.0 (0.0)	0.0 (0.0)	0.0 (0.0)
HC7 Medical abortion	$4.81	83.0%	$3.99	0.4 (0.0)	0.0 (0.0)	0.0 (0.0)	0.0 (0.0)	0.0 (0.0)	0.1 (0.0)

Cambodia				CHE risk 10% threshold (25% threshold)					
Disease category, disease, intervention	Cost ($)	OOP (%)	OOP ($)	Q1	Q2	Q3	Q4	Q5	Total
Perinatal conditions				0.2 (0.0)	0.0 (0.0)	0.0 (0.0)	0.0 (0.0)	0.0 (0.0)	0.0 (0.0)
C13 Cotrimoxazole for HIV-exposed children	$6.55	55.2%	$3.62	0.3 (0.0)	0.0 (0.0)	0.0 (0.0)	0.0 (0.0)	0.0 (0.0)	0.1 (0.0)
HC1 Antibiotics for neonatal pneumonia	$6.22	55.2%	$3.44	0.3 (0.0)	0.0 (0.0)	0.0 (0.0)	0.0 (0.0)	0.0 (0.0)	0.1 (0.0)
HC6 Neonatal sepsis, pneumonia, meningitis	$2.66	55.2%	$1.47	0.0 (0.0)	0.0 (0.0)	0.0 (0.0)	0.0 (0.0)	0.0 (0.0)	0.0 (0.0)

Table B.8. Full model results: Cameroon.

Cameroon				CHE risk 10% threshold (25% threshold)					
Disease category, disease, intervention	Cost ($)	OOP (%)	OOP ($)	Q1	Q2	Q3	Q4	Q5	Total
Childhood health				0.0 (0.0)	0.0 (0.0)	0.0 (0.0)	0.0 (0.0)	0.0 (0.0)	0.0 (0.0)
Childhood health				0.0 (0.0)	0.0 (0.0)	0.0 (0.0)	0.0 (0.0)	0.0 (0.0)	0.0 (0.0)
HC42 Acute pharyngitis treatment	$0.24	44.5%	$0.11	0.0 (0.0)	0.0 (0.0)	0.0 (0.0)	0.0 (0.0)	0.0 (0.0)	0.0 (0.0)
Infectious & parasitic diseases				12.2 (4.9)	11.4 (0.0)	0.8 (0.0)	0.0 (0.0)	0.0 (0.0)	4.9 (1.0)
Diarrheal diseases				0.3 (0.0)	0.0 (0.0)	0.0 (0.0)	0.0 (0.0)	0.0 (0.0)	0.1 (0.0)
HC12 Diagnosis & treatment of infections (IMCI)	$10.29	37.1%	$3.82	0.3 (0.0)	0.0 (0.0)	0.0 (0.0)	0.0 (0.0)	0.0 (0.0)	0.1 (0.0)
HIV/AIDS & other sexually transmitted diseases				15.9 (6.8)	16.3 (0.0)	1.5 (0.0)	0.0 (0.0)	0.0 (0.0)	6.7 (1.4)
HC13 ART & viral load monitoring	$121.57	29.0%	$35.23	17.0 (3.0)	0.0 (0.0)	0.0 (0.0)	0.0 (0.0)	0.0 (0.0)	3.4 (0.6)
HC17 Syndromic management of STI	$10.69	29.0%	$3.10	0.0 (0.0)	0.0 (0.0)	0.0 (0.0)	0.0 (0.0)	0.0 (0.0)	0.0 (0.0)
HC23 HIV, STIs, hepatitis testing & counseling	$6.08	29.0%	$1.76	0.0 (0.0)	0.0 (0.0)	0.0 (0.0)	0.0 (0.0)	0.0 (0.0)	0.0 (0.0)
HC8 HIV & syphilis PMTCT	$313.51	29.0%	$90.86	46.4 (24.0)	65.1 (0.0)	6.0 (0.0)	0.0 (0.0)	0.0 (0.0)	23.5 (4.8)
Malaria				0.0 (0.0)	0.0 (0.0)	0.0 (0.0)	0.0 (0.0)	0.0 (0.0)	0.0 (0.0)
C7 Intermittent preventive treatment (pregnancy)	$1.02	48.1%	$0.49	0.0 (0.0)	0.0 (0.0)	0.0 (0.0)	0.0 (0.0)	0.0 (0.0)	0.0 (0.0)
Tuberculosis				33.6 (11.8)	26.1 (0.0)	0.0 (0.0)	0.0 (0.0)	0.0 (0.0)	11.9 (2.4)
HC27 Diagnosis & treatment of TB	$175.65	41.5%	$72.89	33.6 (11.8)	26.1 (0.0)	0.0 (0.0)	0.0 (0.0)	0.0 (0.0)	11.9 (2.4)
Other infectious & parasitic diseases				0.1 (0.0)	0.0 (0.0)	0.0 (0.0)	0.0 (0.0)	0.0 (0.0)	0.0 (0.0)
HC30 Management & referrals for fever (IMAI)	$6.83	26.9%	$1.84	0.1 (0.0)	0.0 (0.0)	0.0 (0.0)	0.0 (0.0)	0.0 (0.0)	0.0 (0.0)
Noncommunicable diseases (NCDs)				0.6 (0.1)	0.0 (0.0)	0.0 (0.0)	0.0 (0.0)	0.0 (0.0)	0.1 (0.0)
Cardiovascular diseases				1.8 (0.3)	0.0 (0.0)	0.0 (0.0)	0.0 (0.0)	0.0 (0.0)	0.4 (0.1)
HC38 Aspirin for acute myocardial infarction	$0.05	8.7%	$0.00	0.0 (0.0)	0.0 (0.0)	0.0 (0.0)	0.0 (0.0)	0.0 (0.0)	0.0 (0.0)
HC43 Management of ischemic heart disease	$190.17	8.7%	$16.47	1.3 (0.2)	0.0 (0.0)	0.0 (0.0)	0.0 (0.0)	0.0 (0.0)	0.3 (0.0)
HC44 Management of heart failure	$342.48	8.7%	$29.67	4.0 (0.7)	0.0 (0.0)	0.0 (0.0)	0.0 (0.0)	0.0 (0.0)	0.8 (0.1)
Endocrine & metabolic disorders				0.3 (0.0)	0.0 (0.0)	0.0 (0.0)	0.0 (0.0)	0.0 (0.0)	0.1 (0.0)
HC40 Screening & management of diabetes	$92.52	8.9%	$8.22	0.3 (0.0)	0.0 (0.0)	0.0 (0.0)	0.0 (0.0)	0.0 (0.0)	0.1 (0.0)
Mental/behavioral disorders & neurological conditions				0.1 (0.0)	0.0 (0.0)	0.0 (0.0)	0.0 (0.0)	0.0 (0.0)	0.0 (0.0)
HC49 Management of bipolar disorder	$365.39	2.3%	$8.25	0.2 (0.0)	0.0 (0.0)	0.0 (0.0)	0.0 (0.0)	0.0 (0.0)	0.0 (0.0)
HC50 Management of depression	$48.02	2.3%	$1.08	0.0 (0.0)	0.0 (0.0)	0.0 (0.0)	0.0 (0.0)	0.0 (0.0)	0.0 (0.0)
HC51 Management of epilepsy	$53.73	2.3%	$1.21	0.0 (0.0)	0.0 (0.0)	0.0 (0.0)	0.0 (0.0)	0.0 (0.0)	0.0 (0.0)
HC52 Management of schizophrenia	$329.34	2.3%	$7.44	0.1 (0.0)	0.0 (0.0)	0.0 (0.0)	0.0 (0.0)	0.0 (0.0)	0.0 (0.0)
HC66 Psychosocial support & counseling	$21.32	2.3%	$0.48	0.0 (0.0)	0.0 (0.0)	0.0 (0.0)	0.0 (0.0)	0.0 (0.0)	0.0 (0.0)

Cameroon				CHE risk 10% threshold (25% threshold)					
Disease category, disease, intervention	Cost ($)	OOP (%)	OOP ($)	Q1	Q2	Q3	Q4	Q5	Total
Other NCDs				0.1 (0.0)	0.0 (0.0)	0.0 (0.0)	0.0 (0.0)	0.0 (0.0)	0.0 (0.0)
HC47 Palliative care	$21.32	44.4%	$9.46	0.1 (0.0)	0.0 (0.0)	0.0 (0.0)	0.0 (0.0)	0.0 (0.0)	0.0 (0.0)
Reproductive health				3.2 (2.2)	6.2 (0.0)	4.8 (0.0)	0.0 (0.0)	0.0 (0.0)	2.8 (0.4)
Family planning				0.0 (0.0)	0.0 (0.0)	0.0 (0.0)	0.0 (0.0)	0.0 (0.0)	0.0 (0.0)
HC4 Contraceptives	$10.32	1.2%	$0.12	0.0 (0.0)	0.0 (0.0)	0.0 (0.0)	0.0 (0.0)	0.0 (0.0)	0.0 (0.0)
Maternal conditions				5.3 (3.7)	10.3 (0.0)	8.0 (0.0)	0.0 (0.0)	0.0 (0.0)	4.7 (0.7)
C5 Antenatal tetanus immunization	$0.44	76.4%	$0.33	0.0 (0.0)	0.0 (0.0)	0.0 (0.0)	0.0 (0.0)	0.0 (0.0)	0.0 (0.0)
HC11 Basic emergency newborn & obstetric care	$145.10	76.4%	$110.80	30.5 (22.0)	61.9 (0.0)	47.8 (0.0)	0.0 (0.0)	0.0 (0.0)	28.0 (4.4)
HC2 Post-abortion care	$8.23	76.4%	$6.28	0.5 (0.1)	0.0 (0.0)	0.0 (0.0)	0.0 (0.0)	0.0 (0.0)	0.1 (0.0)
HC3 Treatment of premature membrane rupture	$4.66	76.4%	$3.56	0.1 (0.0)	0.0 (0.0)	0.0 (0.0)	0.0 (0.0)	0.0 (0.0)	0.0 (0.0)
HC5 Kangaroo mother care counseling	$4.61	76.4%	$3.52	0.2 (0.0)	0.0 (0.0)	0.0 (0.0)	0.0 (0.0)	0.0 (0.0)	0.0 (0.0)
HC7 Medical abortion	$5.52	76.4%	$4.21	0.5 (0.1)	0.0 (0.0)	0.0 (0.0)	0.0 (0.0)	0.0 (0.0)	0.1 (0.0)
Perinatal conditions				0.2 (0.0)	0.0 (0.0)	0.0 (0.0)	0.0 (0.0)	0.0 (0.0)	0.0 (0.0)
C13 Cotrimoxazole for HIV-exposed children	$11.95	49.4%	$5.91	0.4 (0.1)	0.0 (0.0)	0.0 (0.0)	0.0 (0.0)	0.0 (0.0)	0.1 (0.0)
HC1 Antibiotics for neonatal pneumonia	$6.61	49.4%	$3.27	0.1 (0.0)	0.0 (0.0)	0.0 (0.0)	0.0 (0.0)	0.0 (0.0)	0.0 (0.0)
HC6 Neonatal sepsis, pneumonia, meningitis	$3.51	49.4%	$1.74	0.0 (0.0)	0.0 (0.0)	0.0 (0.0)	0.0 (0.0)	0.0 (0.0)	0.0 (0.0)

Table B.9. Full model results: Democratic Republic of the Congo.

Democratic Republic of the Congo				CHE risk 10% threshold (25% threshold)					
Disease category, disease, intervention	Cost ($)	OOP (%)	OOP ($)	Q1	Q2	Q3	Q4	Q5	Total
Childhood health				0.0 (0.0)	0.0 (0.0)	0.0 (0.0)	0.0 (0.0)	0.0 (0.0)	0.0 (0.0)
Childhood health				0.0 (0.0)	0.0 (0.0)	0.0 (0.0)	0.0 (0.0)	0.0 (0.0)	0.0 (0.0)
HC42 Acute pharyngitis treatment	$0.17	45.3%	$0.08	0.0 (0.0)	0.0 (0.0)	0.0 (0.0)	0.0 (0.0)	0.0 (0.0)	0.0 (0.0)
Infectious & parasitic diseases				2.1 (0.3)	0.0 (0.0)	0.0 (0.0)	0.0 (0.0)	0.0 (0.0)	0.4 (0.1)
Diarrheal diseases				1.3 (0.2)	0.0 (0.0)	0.0 (0.0)	0.0 (0.0)	0.0 (0.0)	0.3 (0.0)
HC12 Diagnosis & treatment of infections (IMCI)	$4.79	74.1%	$3.55	1.3 (0.2)	0.0 (0.0)	0.0 (0.0)	0.0 (0.0)	0.0 (0.0)	0.3 (0.0)
HIV/AIDS & other sexually transmitted diseases				0.2 (0.0)	0.0 (0.0)	0.0 (0.0)	0.0 (0.0)	0.0 (0.0)	0.0 (0.0)
HC13 ART & viral load monitoring	$71.45	1.7%	$1.21	0.1 (0.0)	0.0 (0.0)	0.0 (0.0)	0.0 (0.0)	0.0 (0.0)	0.0 (0.0)
HC17 Syndromic management of STI	$5.67	1.7%	$0.10	0.0 (0.0)	0.0 (0.0)	0.0 (0.0)	0.0 (0.0)	0.0 (0.0)	0.0 (0.0)
HC23 HIV, STIs, hepatitis testing & counseling	$4.31	1.7%	$0.07	0.0 (0.0)	0.0 (0.0)	0.0 (0.0)	0.0 (0.0)	0.0 (0.0)	0.0 (0.0)
HC8 HIV & syphilis PMTCT	$176.35	1.7%	$2.98	0.6 (0.1)	0.0 (0.0)	0.0 (0.0)	0.0 (0.0)	0.0 (0.0)	0.1 (0.0)
Malaria				0.0 (0.0)	0.0 (0.0)	0.0 (0.0)	0.0 (0.0)	0.0 (0.0)	0.0 (0.0)
C7 Intermittent preventive treatment (pregnancy)	$0.45	53.3%	$0.24	0.0 (0.0)	0.0 (0.0)	0.0 (0.0)	0.0 (0.0)	0.0 (0.0)	0.0 (0.0)
Tuberculosis				14.8 (2.1)	0.0 (0.0)	0.0 (0.0)	0.0 (0.0)	0.0 (0.0)	3.0 (0.4)
HC27 Diagnosis & treatment of TB	$135.09	8.3%	$11.22	14.8 (2.1)	0.0 (0.0)	0.0 (0.0)	0.0 (0.0)	0.0 (0.0)	3.0 (0.4)
Other infectious & parasitic diseases				0.1 (0.0)	0.0 (0.0)	0.0 (0.0)	0.0 (0.0)	0.0 (0.0)	0.0 (0.0)
HC30 Management & referrals for fever (IMAI)	$3.11	42.0%	$1.31	0.1 (0.0)	0.0 (0.0)	0.0 (0.0)	0.0 (0.0)	0.0 (0.0)	0.0 (0.0)
Noncommunicable diseases (NCDs)				1.8 (0.4)	0.5 (0.0)	0.0 (0.0)	0.0 (0.0)	0.0 (0.0)	0.5 (0.1)

Democratic Republic of the Congo				CHE risk 10% threshold (25% threshold)					
Disease category, disease, intervention	Cost ($)	OOP (%)	OOP ($)	Q1	Q2	Q3	Q4	Q5	Total
Cardiovascular diseases				4.4 (0.9)	0.6 (0.0)	0.0 (0.0)	0.0 (0.0)	0.0 (0.0)	1.0 (0.2)
HC38 Aspirin for acute myocardial infarction	$0.03	8.7%	$0.00	0.0 (0.0)	0.0 (0.0)	0.0 (0.0)	0.0 (0.0)	0.0 (0.0)	0.0 (0.0)
HC43 Management of ischemic heart disease	$83.97	8.7%	$7.27	1.6 (0.2)	0.0 (0.0)	0.0 (0.0)	0.0 (0.0)	0.0 (0.0)	0.3 (0.0)
HC44 Management of heart failure	$249.96	8.7%	$21.65	11.6 (2.4)	1.8 (0.0)	0.0 (0.0)	0.0 (0.0)	0.0 (0.0)	2.7 (0.5)
Endocrine & metabolic disorders				0.8 (0.1)	0.0 (0.0)	0.0 (0.0)	0.0 (0.0)	0.0 (0.0)	0.2 (0.0)
HC40 Screening & management of diabetes	$64.16	8.9%	$5.70	0.8 (0.1)	0.0 (0.0)	0.0 (0.0)	0.0 (0.0)	0.0 (0.0)	0.2 (0.0)
Mental/behavioral disorders & neurological conditions				0.1 (0.0)	0.0 (0.0)	0.0 (0.0)	0.0 (0.0)	0.0 (0.0)	0.0 (0.0)
HC49 Management of bipolar disorder	$184.57	2.3%	$4.17	0.2 (0.0)	0.0 (0.0)	0.0 (0.0)	0.0 (0.0)	0.0 (0.0)	0.0 (0.0)
HC50 Management of depression	$16.11	2.3%	$0.36	0.0 (0.0)	0.0 (0.0)	0.0 (0.0)	0.0 (0.0)	0.0 (0.0)	0.0 (0.0)
HC51 Management of epilepsy	$27.53	2.3%	$0.62	0.0 (0.0)	0.0 (0.0)	0.0 (0.0)	0.0 (0.0)	0.0 (0.0)	0.0 (0.0)
HC52 Management of schizophrenia	$99.43	2.3%	$2.25	0.1 (0.0)	0.0 (0.0)	0.0 (0.0)	0.0 (0.0)	0.0 (0.0)	0.0 (0.0)
HC66 Psychosocial support & counseling	$64.63	2.3%	$1.46	0.0 (0.0)	0.0 (0.0)	0.0 (0.0)	0.0 (0.0)	0.0 (0.0)	0.0 (0.0)
Other NCDs				3.7 (1.3)	3.5 (0.0)	0.0 (0.0)	0.0 (0.0)	0.0 (0.0)	1.4 (0.3)
HC47 Palliative care	$64.63	44.4%	$28.68	3.7 (1.3)	3.5 (0.0)	0.0 (0.0)	0.0 (0.0)	0.0 (0.0)	1.4 (0.3)
Reproductive health				3.7 (3.2)	4.7 (0.4)	5.9 (0.0)	3.0 (0.0)	0.0 (0.0)	3.5 (0.7)
Family planning				0.0 (0.0)	0.0 (0.0)	0.0 (0.0)	0.0 (0.0)	0.0 (0.0)	0.0 (0.0)
HC4 Contraceptives	$4.97	1.2%	$0.06	0.0 (0.0)	0.0 (0.0)	0.0 (0.0)	0.0 (0.0)	0.0 (0.0)	0.0 (0.0)
Maternal conditions				6.0 (5.4)	7.8 (0.6)	9.9 (0.0)	5.0 (0.0)	0.0 (0.0)	5.7 (1.2)
C5 Antenatal tetanus immunization	$0.39	76.4%	$0.29	0.0 (0.0)	0.0 (0.0)	0.0 (0.0)	0.0 (0.0)	0.0 (0.0)	0.0 (0.0)
HC11 Basic emergency newborn & obstetric care	$69.14	76.4%	$52.80	31.9 (31.8)	46.9 (3.9)	59.4 (0.0)	30.3 (0.0)	0.0 (0.0)	33.7 (7.1)
HC2 Post-abortion care	$4.54	76.4%	$3.47	0.9 (0.1)	0.0 (0.0)	0.0 (0.0)	0.0 (0.0)	0.0 (0.0)	0.2 (0.0)
HC3 Treatment of premature membrane rupture	$4.04	76.4%	$3.08	0.7 (0.1)	0.0 (0.0)	0.0 (0.0)	0.0 (0.0)	0.0 (0.0)	0.1 (0.0)
HC5 Kangaroo mother care counseling	$2.33	76.4%	$1.78	0.2 (0.0)	0.0 (0.0)	0.0 (0.0)	0.0 (0.0)	0.0 (0.0)	0.0 (0.0)
HC7 Medical abortion	$4.81	76.4%	$3.67	2.1 (0.3)	0.0 (0.0)	0.0 (0.0)	0.0 (0.0)	0.0 (0.0)	0.4 (0.1)
Perinatal conditions				0.5 (0.1)	0.0 (0.0)	0.0 (0.0)	0.0 (0.0)	0.0 (0.0)	0.1 (0.0)
C13 Cotrimoxazole for HIV-exposed children	$6.55	49.4%	$3.24	0.7 (0.1)	0.0 (0.0)	0.0 (0.0)	0.0 (0.0)	0.0 (0.0)	0.1 (0.0)
HC1 Antibiotics for neonatal pneumonia	$6.22	49.4%	$3.08	0.6 (0.1)	0.0 (0.0)	0.0 (0.0)	0.0 (0.0)	0.0 (0.0)	0.1 (0.0)
HC6 Neonatal sepsis, pneumonia, meningitis	$2.66	49.4%	$1.31	0.1 (0.0)	0.0 (0.0)	0.0 (0.0)	0.0 (0.0)	0.0 (0.0)	0.0 (0.0)

Table B.10. Full model results: Republic of the Congo.

Republic of the Congo				CHE risk 10% threshold (25% threshold)					
Disease category, disease, intervention	Cost ($)	OOP (%)	OOP ($)	Q1	Q2	Q3	Q4	Q5	Total
Childhood health				0.0 (0.0)	0.0 (0.0)	0.0 (0.0)	0.0 (0.0)	0.0 (0.0)	0.0 (0.0)
Childhood health				0.0 (0.0)	0.0 (0.0)	0.0 (0.0)	0.0 (0.0)	0.0 (0.0)	0.0 (0.0)
HC42 Acute pharyngitis treatment	$0.24	46.1%	$0.11	0.0 (0.0)	0.0 (0.0)	0.0 (0.0)	0.0 (0.0)	0.0 (0.0)	0.0 (0.0)
Infectious & parasitic diseases				0.0 (0.0)	0.0 (0.0)	0.0 (0.0)	0.0 (0.0)	0.0 (0.0)	0.0 (0.0)
Diarrheal diseases				0.0 (0.0)	0.0 (0.0)	0.0 (0.0)	0.0 (0.0)	0.0 (0.0)	0.0 (0.0)
HC12 Diagnosis & treatment of infections (IMCI)	$10.29	28.4%	$2.93	0.0 (0.0)	0.0 (0.0)	0.0 (0.0)	0.0 (0.0)	0.0 (0.0)	0.0 (0.0)

Republic of the Congo				CHE risk 10% threshold (25% threshold)					
Disease category, disease, intervention	Cost ($)	OOP (%)	OOP ($)	Q1	Q2	Q3	Q4	Q5	Total
HIV/AIDS & other sexually transmitted diseases				0.0 (0.0)	0.0 (0.0)	0.0 (0.0)	0.0 (0.0)	0.0 (0.0)	0.0 (0.0)
HC13 ART & viral load monitoring	$121.57	1.7%	$2.12	0.0 (0.0)	0.0 (0.0)	0.0 (0.0)	0.0 (0.0)	0.0 (0.0)	0.0 (0.0)
HC17 Syndromic management of STI	$10.69	1.7%	$0.19	0.0 (0.0)	0.0 (0.0)	0.0 (0.0)	0.0 (0.0)	0.0 (0.0)	0.0 (0.0)
HC23 HIV, STIs, hepatitis testing & counseling	$6.08	1.7%	$0.11	0.0 (0.0)	0.0 (0.0)	0.0 (0.0)	0.0 (0.0)	0.0 (0.0)	0.0 (0.0)
HC8 HIV & syphilis PMTCT	$313.51	1.7%	$5.47	0.0 (0.0)	0.0 (0.0)	0.0 (0.0)	0.0 (0.0)	0.0 (0.0)	0.0 (0.0)
Malaria				0.0 (0.0)	0.0 (0.0)	0.0 (0.0)	0.0 (0.0)	0.0 (0.0)	0.0 (0.0)
C7 Intermittent preventive treatment (pregnancy)	$1.02	31.5%	$0.32	0.0 (0.0)	0.0 (0.0)	0.0 (0.0)	0.0 (0.0)	0.0 (0.0)	0.0 (0.0)
Tuberculosis				0.2 (0.0)	0.0 (0.0)	0.0 (0.0)	0.0 (0.0)	0.0 (0.0)	0.0 (0.0)
HC27 Diagnosis & treatment of TB	$175.65	3.4%	$5.95	0.2 (0.0)	0.0 (0.0)	0.0 (0.0)	0.0 (0.0)	0.0 (0.0)	0.0 (0.0)
Other infectious & parasitic diseases				0.0 (0.0)	0.0 (0.0)	0.0 (0.0)	0.0 (0.0)	0.0 (0.0)	0.0 (0.0)
HC30 Management & referrals for fever (IMAI)	$6.83	26.9%	$1.84	0.0 (0.0)	0.0 (0.0)	0.0 (0.0)	0.0 (0.0)	0.0 (0.0)	0.0 (0.0)
Noncommunicable diseases (NCDs)				0.3 (0.1)	0.0 (0.0)	0.0 (0.0)	0.0 (0.0)	0.0 (0.0)	0.1 (0.0)
Cardiovascular diseases				1.0 (0.2)	0.0 (0.0)	0.0 (0.0)	0.0 (0.0)	0.0 (0.0)	0.2 (0.0)
HC38 Aspirin for acute myocardial infarction	$0.05	8.7%	$0.00	0.0 (0.0)	0.0 (0.0)	0.0 (0.0)	0.0 (0.0)	0.0 (0.0)	0.0 (0.0)
HC43 Management of ischemic heart disease	$190.17	8.7%	$16.47	0.7 (0.1)	0.0 (0.0)	0.0 (0.0)	0.0 (0.0)	0.0 (0.0)	0.1 (0.0)
HC44 Management of heart failure	$342.48	8.7%	$29.67	2.2 (0.4)	0.0 (0.0)	0.0 (0.0)	0.0 (0.0)	0.0 (0.0)	0.4 (0.1)
Endocrine & metabolic disorders				0.1 (0.0)	0.0 (0.0)	0.0 (0.0)	0.0 (0.0)	0.0 (0.0)	0.0 (0.0)
HC40 Screening & management of diabetes	$92.52	8.9%	$8.22	0.1 (0.0)	0.0 (0.0)	0.0 (0.0)	0.0 (0.0)	0.0 (0.0)	0.0 (0.0)
Mental/behavioral disorders & neurological conditions				0.0 (0.0)	0.0 (0.0)	0.0 (0.0)	0.0 (0.0)	0.0 (0.0)	0.0 (0.0)
HC49 Management of bipolar disorder	$365.39	2.3%	$8.25	0.0 (0.0)	0.0 (0.0)	0.0 (0.0)	0.0 (0.0)	0.0 (0.0)	0.0 (0.0)
HC50 Management of depression	$48.02	2.3%	$1.08	0.0 (0.0)	0.0 (0.0)	0.0 (0.0)	0.0 (0.0)	0.0 (0.0)	0.0 (0.0)
HC51 Management of epilepsy	$53.73	2.3%	$1.21	0.0 (0.0)	0.0 (0.0)	0.0 (0.0)	0.0 (0.0)	0.0 (0.0)	0.0 (0.0)
HC52 Management of schizophrenia	$329.34	2.3%	$7.44	0.0 (0.0)	0.0 (0.0)	0.0 (0.0)	0.0 (0.0)	0.0 (0.0)	0.0 (0.0)
HC66 Psychosocial support & counseling	$21.32	2.3%	$0.48	0.0 (0.0)	0.0 (0.0)	0.0 (0.0)	0.0 (0.0)	0.0 (0.0)	0.0 (0.0)
Other NCDs				0.0 (0.0)	0.0 (0.0)	0.0 (0.0)	0.0 (0.0)	0.0 (0.0)	0.0 (0.0)
HC47 Palliative care	$21.32	44.4%	$9.46	0.0 (0.0)	0.0 (0.0)	0.0 (0.0)	0.0 (0.0)	0.0 (0.0)	0.0 (0.0)
Reproductive health				3.1 (0.6)	0.0 (0.0)	0.0 (0.0)	0.0 (0.0)	0.0 (0.0)	0.6 (0.1)
Family planning				0.0 (0.0)	0.0 (0.0)	0.0 (0.0)	0.0 (0.0)	0.0 (0.0)	0.0 (0.0)
HC4 Contraceptives	$10.32	1.2%	$0.12	0.0 (0.0)	0.0 (0.0)	0.0 (0.0)	0.0 (0.0)	0.0 (0.0)	0.0 (0.0)
Maternal conditions				5.1 (1.1)	0.1 (0.0)	0.0 (0.0)	0.0 (0.0)	0.0 (0.0)	1.0 (0.2)
C5 Antenatal tetanus immunization	$0.44	76.4%	$0.33	0.0 (0.0)	0.0 (0.0)	0.0 (0.0)	0.0 (0.0)	0.0 (0.0)	0.0 (0.0)
HC11 Basic emergency newborn & obstetric care	$145.10	76.4%	$110.80	30.1 (6.4)	0.4 (0.0)	0.0 (0.0)	0.0 (0.0)	0.0 (0.0)	6.1 (1.3)
HC2 Post-abortion care	$8.23	76.4%	$6.28	0.2 (0.0)	0.0 (0.0)	0.0 (0.0)	0.0 (0.0)	0.0 (0.0)	0.0 (0.0)
HC3 Treatment of premature membrane rupture	$4.66	76.4%	$3.56	0.0 (0.0)	0.0 (0.0)	0.0 (0.0)	0.0 (0.0)	0.0 (0.0)	0.0 (0.0)
HC5 Kangaroo mother care counseling	$4.61	76.4%	$3.52	0.0 (0.0)	0.0 (0.0)	0.0 (0.0)	0.0 (0.0)	0.0 (0.0)	0.0 (0.0)
HC7 Medical abortion	$5.52	76.4%	$4.21	0.1 (0.0)	0.0 (0.0)	0.0 (0.0)	0.0 (0.0)	0.0 (0.0)	0.0 (0.0)
Perinatal conditions				0.1 (0.0)	0.0 (0.0)	0.0 (0.0)	0.0 (0.0)	0.0 (0.0)	0.0 (0.0)
C13 Cotrimoxazole for HIV-exposed children	$11.95	49.4%	$5.91	0.1 (0.0)	0.0 (0.0)	0.0 (0.0)	0.0 (0.0)	0.0 (0.0)	0.0 (0.0)
HC1 Antibiotics for neonatal pneumonia	$6.61	49.4%	$3.27	0.0 (0.0)	0.0 (0.0)	0.0 (0.0)	0.0 (0.0)	0.0 (0.0)	0.0 (0.0)
HC6 Neonatal sepsis, pneumonia, meningitis	$3.51	49.4%	$1.74	0.0 (0.0)	0.0 (0.0)	0.0 (0.0)	0.0 (0.0)	0.0 (0.0)	0.0 (0.0)

Table B.11. Full model results: Côte d'Ivoire.

**Côte d'Ivoire**				**CHE risk** 10% threshold (25% threshold)					
Disease category, disease, intervention	Cost ($)	OOP (%)	OOP ($)	Q1	Q2	Q3	Q4	Q5	Total
Childhood health				0.0 (0.0)	0.0 (0.0)	0.0 (0.0)	0.0 (0.0)	0.0 (0.0)	0.0 (0.0)
Childhood health				0.0 (0.0)	0.0 (0.0)	0.0 (0.0)	0.0 (0.0)	0.0 (0.0)	0.0 (0.0)
HC42 Acute pharyngitis treatment	$0.24	42.3%	$0.10	0.0 (0.0)	0.0 (0.0)	0.0 (0.0)	0.0 (0.0)	0.0 (0.0)	0.0 (0.0)
Infectious & parasitic diseases				0.2 (0.0)	0.0 (0.0)	0.0 (0.0)	0.0 (0.0)	0.0 (0.0)	0.0 (0.0)
Diarrheal diseases				0.3 (0.0)	0.0 (0.0)	0.0 (0.0)	0.0 (0.0)	0.0 (0.0)	0.1 (0.0)
HC12 Diagnosis & treatment of infections (IMCI)	$10.29	91.7%	$9.44	0.3 (0.0)	0.0 (0.0)	0.0 (0.0)	0.0 (0.0)	0.0 (0.0)	0.1 (0.0)
HIV/AIDS & other sexually transmitted diseases				0.1 (0.0)	0.0 (0.0)	0.0 (0.0)	0.0 (0.0)	0.0 (0.0)	0.0 (0.0)
HC13 ART & viral load monitoring	$121.57	3.5%	$4.25	0.0 (0.0)	0.0 (0.0)	0.0 (0.0)	0.0 (0.0)	0.0 (0.0)	0.0 (0.0)
HC17 Syndromic management of STI	$10.69	3.5%	$0.37	0.0 (0.0)	0.0 (0.0)	0.0 (0.0)	0.0 (0.0)	0.0 (0.0)	0.0 (0.0)
HC23 HIV, STIs, hepatitis testing & counseling	$6.08	3.5%	$0.21	0.0 (0.0)	0.0 (0.0)	0.0 (0.0)	0.0 (0.0)	0.0 (0.0)	0.0 (0.0)
HC8 HIV & syphilis PMTCT	$313.51	3.5%	$10.97	0.5 (0.0)	0.0 (0.0)	0.0 (0.0)	0.0 (0.0)	0.0 (0.0)	0.1 (0.0)
Malaria				0.0 (0.0)	0.0 (0.0)	0.0 (0.0)	0.0 (0.0)	0.0 (0.0)	0.0 (0.0)
C7 Intermittent preventive treatment (pregnancy)	$1.02	75.8%	$0.78	0.0 (0.0)	0.0 (0.0)	0.0 (0.0)	0.0 (0.0)	0.0 (0.0)	0.0 (0.0)
Tuberculosis				0.5 (0.0)	0.0 (0.0)	0.0 (0.0)	0.0 (0.0)	0.0 (0.0)	0.1 (0.0)
HC27 Diagnosis & treatment of TB	$175.65	6.6%	$11.63	0.5 (0.0)	0.0 (0.0)	0.0 (0.0)	0.0 (0.0)	0.0 (0.0)	0.1 (0.0)
Other infectious & parasitic diseases				0.0 (0.0)	0.0 (0.0)	0.0 (0.0)	0.0 (0.0)	0.0 (0.0)	0.0 (0.0)
HC30 Management & referrals for fever (IMAI)	$6.83	59.8%	$4.09	0.0 (0.0)	0.0 (0.0)	0.0 (0.0)	0.0 (0.0)	0.0 (0.0)	0.0 (0.0)
Noncommunicable diseases (NCDs)				14.9 (9.8)	14.7 (0.5)	13.9 (0.0)	3.6 (0.0)	0.0 (0.0)	9.4 (2.0)
Cardiovascular diseases				12.9 (9.0)	13.8 (1.6)	9.0 (0.0)	9.0 (0.0)	0.0 (0.0)	8.9 (2.1)
HC38 Aspirin for acute myocardial infarction	$0.05	71.3%	$0.04	0.0 (0.0)	0.0 (0.0)	0.0 (0.0)	0.0 (0.0)	0.0 (0.0)	0.0 (0.0)
HC43 Management of ischemic heart disease	$190.17	71.3%	$135.69	19.3 (7.6)	20.5 (0.0)	1.7 (0.0)	0.0 (0.0)	0.0 (0.0)	8.3 (1.5)
HC44 Management of heart failure	$342.48	71.3%	$244.36	19.3 (19.3)	21.0 (4.8)	25.2 (0.0)	27.0 (0.0)	0.0 (0.0)	18.5 (4.8)
Endocrine & metabolic disorders				7.8 (1.3)	0.8 (0.0)	0.0 (0.0)	0.0 (0.0)	0.0 (0.0)	1.7 (0.3)
HC40 Screening & management of diabetes	$92.52	98.5%	$91.16	7.8 (1.3)	0.8 (0.0)	0.0 (0.0)	0.0 (0.0)	0.0 (0.0)	1.7 (0.3)
Mental/behavioral disorders & neurological conditions				20.5 (13.9)	21.0 (0.0)	22.5 (0.0)	1.9 (0.0)	0.0 (0.0)	13.1 (2.8)
HC49 Management of bipolar disorder	$365.39	52.2%	$190.86	48.5 (38.0)	52.3 (0.0)	62.8 (0.0)	9.3 (0.0)	0.0 (0.0)	34.6 (7.6)
HC50 Management of depression	$48.02	52.2%	$25.09	3.3 (0.3)	0.0 (0.0)	0.0 (0.0)	0.0 (0.0)	0.0 (0.0)	0.7 (0.1)
HC51 Management of epilepsy	$53.73	52.2%	$28.07	1.8 (0.2)	0.0 (0.0)	0.0 (0.0)	0.0 (0.0)	0.0 (0.0)	0.4 (0.0)
HC52 Management of schizophrenia	$329.34	52.2%	$172.03	48.3 (30.8)	52.5 (0.0)	49.5 (0.0)	0.0 (0.0)	0.0 (0.0)	30.0 (6.2)
HC66 Psychosocial support & counseling	$21.32	52.2%	$11.14	0.4 (0.0)	0.0 (0.0)	0.0 (0.0)	0.0 (0.0)	0.0 (0.0)	0.1 (0.0)
Other NCDs				0.0 (0.0)	0.0 (0.0)	0.0 (0.0)	0.0 (0.0)	0.0 (0.0)	0.0 (0.0)
HC47 Palliative care	$21.32	37.2%	$7.94	0.0 (0.0)	0.0 (0.0)	0.0 (0.0)	0.0 (0.0)	0.0 (0.0)	0.0 (0.0)
Reproductive health				3.7 (1.2)	3.4 (0.0)	0.0 (0.0)	0.0 (0.0)	0.0 (0.0)	1.4 (0.2)
Family planning				0.0 (0.0)	0.0 (0.0)	0.0 (0.0)	0.0 (0.0)	0.0 (0.0)	0.0 (0.0)
HC4 Contraceptives	$10.32	0.1%	$0.01	0.0 (0.0)	0.0 (0.0)	0.0 (0.0)	0.0 (0.0)	0.0 (0.0)	0.0 (0.0)
Maternal conditions				6.1 (2.0)	5.7 (0.0)	0.0 (0.0)	0.0 (0.0)	0.0 (0.0)	2.4 (0.4)
C5 Antenatal tetanus immunization	$0.44	85.8%	$0.37	0.0 (0.0)	0.0 (0.0)	0.0 (0.0)	0.0 (0.0)	0.0 (0.0)	0.0 (0.0)
HC11 Basic emergency newborn & obstetric care	$145.10	85.8%	$124.53	36.5 (12.0)	34.1 (0.0)	0.0 (0.0)	0.0 (0.0)	0.0 (0.0)	14.1 (2.4)

Côte d'Ivoire<br>Disease category, disease, intervention	Cost ($)	OOP (%)	OOP ($)	CHE risk<br>10% threshold (25% threshold)<br>Q1	Q2	Q3	Q4	Q5	Total
HC2 Post-abortion care	$8.23	85.8%	$7.06	0.1 (0.0)	0.0 (0.0)	0.0 (0.0)	0.0 (0.0)	0.0 (0.0)	0.0 (0.0)
HC3 Treatment of premature membrane rupture	$4.66	85.8%	$4.00	0.0 (0.0)	0.0 (0.0)	0.0 (0.0)	0.0 (0.0)	0.0 (0.0)	0.0 (0.0)
HC5 Kangaroo mother care counseling	$4.61	85.8%	$3.96	0.0 (0.0)	0.0 (0.0)	0.0 (0.0)	0.0 (0.0)	0.0 (0.0)	0.0 (0.0)
HC7 Medical abortion	$5.52	85.8%	$4.73	0.1 (0.0)	0.0 (0.0)	0.0 (0.0)	0.0 (0.0)	0.0 (0.0)	0.0 (0.0)
Perinatal conditions				0.0 (0.0)	0.0 (0.0)	0.0 (0.0)	0.0 (0.0)	0.0 (0.0)	0.0 (0.0)
C13 Cotrimoxazole for HIV-exposed children	$11.95	67.0%	$8.01	0.1 (0.0)	0.0 (0.0)	0.0 (0.0)	0.0 (0.0)	0.0 (0.0)	0.0 (0.0)
HC1 Antibiotics for neonatal pneumonia	$6.61	67.0%	$4.43	0.0 (0.0)	0.0 (0.0)	0.0 (0.0)	0.0 (0.0)	0.0 (0.0)	0.0 (0.0)
HC6 Neonatal sepsis, pneumonia, meningitis	$3.51	67.0%	$2.35	0.0 (0.0)	0.0 (0.0)	0.0 (0.0)	0.0 (0.0)	0.0 (0.0)	0.0 (0.0)

Table B.12. Full model results: Ethiopia.

Ethiopia<br>Disease category, disease, intervention	Cost ($)	OOP (%)	OOP ($)	CHE risk<br>10% threshold (25% threshold)<br>Q1	Q2	Q3	Q4	Q5	Total
Childhood health				0.0 (0.0)	0.0 (0.0)	0.0 (0.0)	0.0 (0.0)	0.0 (0.0)	0.0 (0.0)
Childhood health				0.0 (0.0)	0.0 (0.0)	0.0 (0.0)	0.0 (0.0)	0.0 (0.0)	0.0 (0.0)
HC42 Acute pharyngitis treatment	$0.17	51.5%	$0.09	0.0 (0.0)	0.0 (0.0)	0.0 (0.0)	0.0 (0.0)	0.0 (0.0)	0.0 (0.0)
Infectious & parasitic diseases				6.4 (6.2)	6.9 (2.3)	7.9 (0.0)	9.9 (0.0)	0.8 (0.0)	6.4 (1.7)
Diarrheal diseases				0.2 (0.0)	0.0 (0.0)	0.0 (0.0)	0.0 (0.0)	0.0 (0.0)	0.0 (0.0)
HC12 Diagnosis & treatment of infections (IMCI)	$4.79	42.4%	$2.03	0.2 (0.0)	0.0 (0.0)	0.0 (0.0)	0.0 (0.0)	0.0 (0.0)	0.0 (0.0)
HIV/AIDS & other sexually transmitted diseases				0.4 (0.0)	0.0 (0.0)	0.0 (0.0)	0.0 (0.0)	0.0 (0.0)	0.1 (0.0)
HC13 ART & viral load monitoring	$71.45	2.2%	$1.58	0.1 (0.0)	0.0 (0.0)	0.0 (0.0)	0.0 (0.0)	0.0 (0.0)	0.0 (0.0)
HC17 Syndromic management of STI	$5.67	2.2%	$0.13	0.0 (0.0)	0.0 (0.0)	0.0 (0.0)	0.0 (0.0)	0.0 (0.0)	0.0 (0.0)
HC23 HIV, STIs, hepatitis testing & counseling	$4.31	2.2%	$0.10	0.0 (0.0)	0.0 (0.0)	0.0 (0.0)	0.0 (0.0)	0.0 (0.0)	0.0 (0.0)
HC8 HIV & syphilis PMTCT	$176.35	2.2%	$3.90	1.6 (0.1)	0.0 (0.0)	0.0 (0.0)	0.0 (0.0)	0.0 (0.0)	0.3 (0.0)
Malaria				0.0 (0.0)	0.0 (0.0)	0.0 (0.0)	0.0 (0.0)	0.0 (0.0)	0.0 (0.0)
C7 Intermittent preventive treatment (pregnancy)	$0.45	14.6%	$0.07	0.0 (0.0)	0.0 (0.0)	0.0 (0.0)	0.0 (0.0)	0.0 (0.0)	0.0 (0.0)
Tuberculosis				49.6 (49.6)	55.4 (18.4)	63.3 (0.0)	78.9 (0.0)	6.0 (0.0)	50.6 (13.6)
HC27 Diagnosis & treatment of TB	$135.09	37.5%	$50.59	49.6 (49.6)	55.4 (18.4)	63.3 (0.0)	78.9 (0.0)	6.0 (0.0)	50.6 (13.6)
Other infectious & parasitic diseases				0.0 (0.0)	0.0 (0.0)	0.0 (0.0)	0.0 (0.0)	0.0 (0.0)	0.0 (0.0)
HC30 Management & referrals for fever (IMAI)	$3.11	19.8%	$0.62	0.0 (0.0)	0.0 (0.0)	0.0 (0.0)	0.0 (0.0)	0.0 (0.0)	0.0 (0.0)
Noncommunicable diseases (NCDs)				6.6 (3.9)	4.8 (1.7)	5.1 (1.5)	3.8 (0.8)	2.6 (0.3)	4.6 (1.6)
Cardiovascular diseases				2.6 (2.6)	2.9 (1.7)	3.3 (1.6)	3.9 (2.1)	3.0 (0.9)	3.2 (1.8)
HC38 Aspirin for acute myocardial infarction	$0.03	56.5%	$0.02	0.0 (0.0)	0.0 (0.0)	0.0 (0.0)	0.0 (0.0)	0.0 (0.0)	0.0 (0.0)
HC43 Management of ischemic heart disease	$83.97	56.5%	$47.48	3.9 (3.9)	4.4 (0.8)	5.1 (0.0)	5.5 (0.0)	0.0 (0.0)	3.8 (0.9)
HC44 Management of heart failure	$249.96	56.5%	$141.32	3.9 (3.9)	4.4 (4.4)	4.9 (4.9)	6.3 (6.3)	8.9 (2.8)	5.7 (4.5)
Endocrine & metabolic disorders				4.2 (2.7)	4.7 (0.0)	4.7 (0.0)	0.0 (0.0)	0.0 (0.0)	2.7 (0.5)
HC40 Screening & management of diabetes	$64.16	53.9%	$34.58	4.2 (2.7)	4.7 (0.0)	4.7 (0.0)	0.0 (0.0)	0.0 (0.0)	2.7 (0.5)
Mental/behavioral disorders & neurological conditions				9.2 (4.5)	5.1 (2.3)	5.3 (2.0)	4.9 (0.3)	3.4 (0.0)	5.6 (1.8)
HC49 Management of bipolar disorder	$184.57	50.5%	$93.19	7.7 (7.7)	8.5 (8.5)	9.9 (9.9)	12.4 (1.7)	16.3 (0.0)	11.0 (5.6)

Ethiopia				CHE risk 10% threshold (25% threshold)					
Disease category, disease, intervention	Cost ($)	OOP (%)	OOP ($)	Q1	Q2	Q3	Q4	Q5	Total
HC50 Management of depression	$16.11	50.5%	$8.14	1.5 (0.1)	0.0 (0.0)	0.0 (0.0)	0.0 (0.0)	0.0 (0.0)	0.3 (0.0)
HC51 Management of epilepsy	$27.53	50.5%	$13.90	21.4 (2.6)	0.0 (0.0)	0.0 (0.0)	0.0 (0.0)	0.0 (0.0)	4.3 (0.5)
HC52 Management of schizophrenia	$99.43	50.5%	$50.20	7.7 (7.7)	8.6 (2.7)	9.8 (0.0)	12.1 (0.0)	0.8 (0.0)	7.8 (2.1)
HC66 Psychosocial support & counseling	$64.63	50.5%	$32.63	7.7 (4.4)	8.5 (0.0)	6.9 (0.0)	0.0 (0.0)	0.0 (0.0)	4.6 (0.9)
Other NCDs				7.7 (5.8)	8.6 (0.0)	9.8 (0.0)	1.8 (0.0)	0.0 (0.0)	5.6 (1.2)
HC47 Palliative care	$64.63	57.7%	$37.32	7.7 (5.8)	8.6 (0.0)	9.8 (0.0)	1.8 (0.0)	0.0 (0.0)	5.6 (1.2)
Reproductive health				1.4 (0.3)	1.0 (0.0)	0.0 (0.0)	0.0 (0.0)	0.0 (0.0)	0.5 (0.1)
Family planning				0.0 (0.0)	0.0 (0.0)	0.0 (0.0)	0.0 (0.0)	0.0 (0.0)	0.0 (0.0)
HC4 Contraceptives	$4.97	0.3%	$0.01	0.0 (0.0)	0.0 (0.0)	0.0 (0.0)	0.0 (0.0)	0.0 (0.0)	0.0 (0.0)
Maternal conditions				2.4 (0.5)	1.7 (0.0)	0.0 (0.0)	0.0 (0.0)	0.0 (0.0)	0.8 (0.1)
C5 Antenatal tetanus immunization	$0.39	29.9%	$0.12	0.0 (0.0)	0.0 (0.0)	0.0 (0.0)	0.0 (0.0)	0.0 (0.0)	0.0 (0.0)
HC11 Basic emergency newborn & obstetric care	$69.14	29.9%	$20.66	14.0 (2.9)	10.4 (0.0)	0.0 (0.0)	0.0 (0.0)	0.0 (0.0)	4.9 (0.6)
HC2 Post-abortion care	$4.54	29.9%	$1.36	0.0 (0.0)	0.0 (0.0)	0.0 (0.0)	0.0 (0.0)	0.0 (0.0)	0.0 (0.0)
HC3 Treatment of premature membrane rupture	$4.04	29.9%	$1.21	0.0 (0.0)	0.0 (0.0)	0.0 (0.0)	0.0 (0.0)	0.0 (0.0)	0.0 (0.0)
HC5 Kangaroo mother care counseling	$2.33	29.9%	$0.70	0.0 (0.0)	0.0 (0.0)	0.0 (0.0)	0.0 (0.0)	0.0 (0.0)	0.0 (0.0)
HC7 Medical abortion	$4.81	29.9%	$1.44	0.1 (0.0)	0.0 (0.0)	0.0 (0.0)	0.0 (0.0)	0.0 (0.0)	0.0 (0.0)
Perinatal conditions				0.1 (0.0)	0.0 (0.0)	0.0 (0.0)	0.0 (0.0)	0.0 (0.0)	0.0 (0.0)
C13 Cotrimoxazole for HIV-exposed children	$6.55	38.6%	$2.53	0.2 (0.0)	0.0 (0.0)	0.0 (0.0)	0.0 (0.0)	0.0 (0.0)	0.0 (0.0)
HC1 Antibiotics for neonatal pneumonia	$6.22	38.6%	$2.40	0.1 (0.0)	0.0 (0.0)	0.0 (0.0)	0.0 (0.0)	0.0 (0.0)	0.0 (0.0)
HC6 Neonatal sepsis, pneumonia, meningitis	$2.66	38.6%	$1.03	0.0 (0.0)	0.0 (0.0)	0.0 (0.0)	0.0 (0.0)	0.0 (0.0)	0.0 (0.0)

Table B.13. Full model results: Republic of the Gambia.

Republic of the Gambia				CHE risk 10% threshold (25% threshold)					
Disease category, disease, intervention	Cost ($)	OOP (%)	OOP ($)	Q1	Q2	Q3	Q4	Q5	Total
Childhood health				0.0 (0.0)	0.0 (0.0)	0.0 (0.0)	0.0 (0.0)	0.0 (0.0)	0.0 (0.0)
Childhood health				0.0 (0.0)	0.0 (0.0)	0.0 (0.0)	0.0 (0.0)	0.0 (0.0)	0.0 (0.0)
HC42 Acute pharyngitis treatment	$0.17	42.3%	$0.07	0.0 (0.0)	0.0 (0.0)	0.0 (0.0)	0.0 (0.0)	0.0 (0.0)	0.0 (0.0)
Infectious & parasitic diseases				6.4 (6.2)	6.9 (2.3)	7.9 (0.0)	9.9 (0.0)	0.8 (0.0)	6.4 (1.7)
Diarrheal diseases				0.2 (0.0)	0.0 (0.0)	0.0 (0.0)	0.0 (0.0)	0.0 (0.0)	0.0 (0.0)
HC12 Diagnosis & treatment of infections (IMCI)	$4.79	65.9%	$3.16	0.2 (0.0)	0.0 (0.0)	0.0 (0.0)	0.0 (0.0)	0.0 (0.0)	0.0 (0.0)
HIV/AIDS & other sexually transmitted diseases				0.4 (0.0)	0.0 (0.0)	0.0 (0.0)	0.0 (0.0)	0.0 (0.0)	0.1 (0.0)
HC13 ART & viral load monitoring	$71.45	22.9%	$16.35	0.1 (0.0)	0.0 (0.0)	0.0 (0.0)	0.0 (0.0)	0.0 (0.0)	0.0 (0.0)
HC17 Syndromic management of STI	$5.67	22.9%	$1.30	0.0 (0.0)	0.0 (0.0)	0.0 (0.0)	0.0 (0.0)	0.0 (0.0)	0.0 (0.0)
HC23 HIV, STIs, hepatitis testing & counseling	$4.31	22.9%	$0.99	0.0 (0.0)	0.0 (0.0)	0.0 (0.0)	0.0 (0.0)	0.0 (0.0)	0.0 (0.0)
HC8 HIV & syphilis PMTCT	$176.35	22.9%	$40.34	1.6 (0.1)	0.0 (0.0)	0.0 (0.0)	0.0 (0.0)	0.0 (0.0)	0.3 (0.0)
Malaria				0.0 (0.0)	0.0 (0.0)	0.0 (0.0)	0.0 (0.0)	0.0 (0.0)	0.0 (0.0)
C7 Intermittent preventive treatment (pregnancy)	$0.45	53.2%	$0.24	0.0 (0.0)	0.0 (0.0)	0.0 (0.0)	0.0 (0.0)	0.0 (0.0)	0.0 (0.0)
Tuberculosis				49.6 (49.6)	55.4 (18.4)	63.3 (0.0)	78.9 (0.0)	6.0 (0.0)	50.6 (13.6)

**Republic of the Gambia**				**CHE risk** 10% threshold (25% threshold)					
Disease category, disease, intervention	Cost ($)	OOP (%)	OOP ($)	Q1	Q2	Q3	Q4	Q5	Total
HC27 Diagnosis & treatment of TB	$135.09	40.3%	$54.44	49.6 (49.6)	55.4 (18.4)	63.3 (0.0)	78.9 (0.0)	6.0 (0.0)	50.6 (13.6)
Other infectious & parasitic diseases				0.0 (0.0)	0.0 (0.0)	0.0 (0.0)	0.0 (0.0)	0.0 (0.0)	0.0 (0.0)
HC30 Management & referrals for fever (IMAI)	$3.11	11.8%	$0.37	0.0 (0.0)	0.0 (0.0)	0.0 (0.0)	0.0 (0.0)	0.0 (0.0)	0.0 (0.0)
Noncommunicable diseases (NCDs)				6.6 (3.9)	4.8 (1.7)	5.1 (1.5)	3.8 (0.8)	2.6 (0.3)	4.6 (1.6)
Cardiovascular diseases				2.6 (2.6)	2.9 (1.7)	3.3 (1.6)	3.9 (2.1)	3.0 (0.9)	3.2 (1.8)
HC38 Aspirin for acute myocardial infarction	$0.03	59.8%	$0.02	0.0 (0.0)	0.0 (0.0)	0.0 (0.0)	0.0 (0.0)	0.0 (0.0)	0.0 (0.0)
HC43 Management of ischemic heart disease	$83.97	59.8%	$50.24	3.9 (3.9)	4.4 (0.8)	5.1 (0.0)	5.5 (0.0)	0.0 (0.0)	3.8 (0.9)
HC44 Management of heart failure	$249.96	59.8%	$149.56	3.9 (3.9)	4.4 (4.4)	4.9 (4.9)	6.3 (6.3)	8.9 (2.8)	5.7 (4.5)
Endocrine & metabolic disorders				4.2 (2.7)	4.7 (0.0)	4.7 (0.0)	0.0 (0.0)	0.0 (0.0)	2.7 (0.5)
HC40 Screening & management of diabetes	$64.16	63.7%	$40.85	4.2 (2.7)	4.7 (0.0)	4.7 (0.0)	0.0 (0.0)	0.0 (0.0)	2.7 (0.5)
Mental/behavioral disorders & neurological conditions				9.2 (4.5)	5.1 (2.3)	5.3 (2.0)	4.9 (0.3)	3.4 (0.0)	5.6 (1.8)
HC49 Management of bipolar disorder	$184.57	52.2%	$96.41	7.7 (7.7)	8.5 (8.5)	9.9 (9.9)	12.4 (1.7)	16.3 (0.0)	11.0 (5.6)
HC50 Management of depression	$16.11	52.2%	$8.42	1.5 (0.1)	0.0 (0.0)	0.0 (0.0)	0.0 (0.0)	0.0 (0.0)	0.3 (0.0)
HC51 Management of epilepsy	$27.53	52.2%	$14.38	21.4 (2.6)	0.0 (0.0)	0.0 (0.0)	0.0 (0.0)	0.0 (0.0)	4.3 (0.5)
HC52 Management of schizophrenia	$99.43	52.2%	$51.94	7.7 (7.7)	8.6 (2.7)	9.8 (0.0)	12.1 (0.0)	0.8 (0.0)	7.8 (2.1)
HC66 Psychosocial support & counseling	$64.63	52.2%	$33.76	7.7 (4.4)	8.5 (0.0)	6.9 (0.0)	0.0 (0.0)	0.0 (0.0)	4.6 (0.9)
Other NCDs				7.7 (5.8)	8.6 (0.0)	9.8 (0.0)	1.8 (0.0)	0.0 (0.0)	5.6 (1.2)
HC47 Palliative care	$64.63	37.2%	$24.06	7.7 (5.8)	8.6 (0.0)	9.8 (0.0)	1.8 (0.0)	0.0 (0.0)	5.6 (1.2)
Reproductive health				1.4 (0.3)	1.0 (0.0)	0.0 (0.0)	0.0 (0.0)	0.0 (0.0)	0.5 (0.1)
Family planning				0.0 (0.0)	0.0 (0.0)	0.0 (0.0)	0.0 (0.0)	0.0 (0.0)	0.0 (0.0)
HC4 Contraceptives	$4.97	22.9%	$1.14	0.0 (0.0)	0.0 (0.0)	0.0 (0.0)	0.0 (0.0)	0.0 (0.0)	0.0 (0.0)
Maternal conditions				2.4 (0.5)	1.7 (0.0)	0.0 (0.0)	0.0 (0.0)	0.0 (0.0)	0.8 (0.1)
C5 Antenatal tetanus immunization	$0.39	52.6%	$0.20	0.0 (0.0)	0.0 (0.0)	0.0 (0.0)	0.0 (0.0)	0.0 (0.0)	0.0 (0.0)
HC11 Basic emergency newborn & obstetric care	$69.14	52.6%	$36.34	14.0 (2.9)	10.4 (0.0)	0.0 (0.0)	0.0 (0.0)	0.0 (0.0)	4.9 (0.6)
HC2 Post-abortion care	$4.54	52.6%	$2.39	0.0 (0.0)	0.0 (0.0)	0.0 (0.0)	0.0 (0.0)	0.0 (0.0)	0.0 (0.0)
HC3 Treatment of premature membrane rupture	$4.04	52.6%	$2.12	0.0 (0.0)	0.0 (0.0)	0.0 (0.0)	0.0 (0.0)	0.0 (0.0)	0.0 (0.0)
HC5 Kangaroo mother care counseling	$2.33	52.6%	$1.22	0.0 (0.0)	0.0 (0.0)	0.0 (0.0)	0.0 (0.0)	0.0 (0.0)	0.0 (0.0)
HC7 Medical abortion	$4.81	52.6%	$2.53	0.1 (0.0)	0.0 (0.0)	0.0 (0.0)	0.0 (0.0)	0.0 (0.0)	0.0 (0.0)
Perinatal conditions				0.1 (0.0)	0.0 (0.0)	0.0 (0.0)	0.0 (0.0)	0.0 (0.0)	0.0 (0.0)
C13 Cotrimoxazole for HIV-exposed children	$6.55	53.1%	$3.48	0.2 (0.0)	0.0 (0.0)	0.0 (0.0)	0.0 (0.0)	0.0 (0.0)	0.0 (0.0)
HC1 Antibiotics for neonatal pneumonia	$6.22	53.1%	$3.30	0.1 (0.0)	0.0 (0.0)	0.0 (0.0)	0.0 (0.0)	0.0 (0.0)	0.0 (0.0)
HC6 Neonatal sepsis, pneumonia, meningitis	$2.66	53.1%	$1.41	0.0 (0.0)	0.0 (0.0)	0.0 (0.0)	0.0 (0.0)	0.0 (0.0)	0.0 (0.0)

Table B.14. Full model results: Ghana.

**Ghana**				**CHE risk** 10% threshold (25% threshold)					
Disease category, disease, intervention	Cost ($)	OOP (%)	OOP ($)	Q1	Q2	Q3	Q4	Q5	Total
Childhood health				0.0 (0.0)	0.0 (0.0)	0.0 (0.0)	0.0 (0.0)	0.0 (0.0)	0.0 (0.0)
Childhood health				0.0 (0.0)	0.0 (0.0)	0.0 (0.0)	0.0 (0.0)	0.0 (0.0)	0.0 (0.0)

**Ghana**				**CHE risk**<br>10% threshold (25% threshold)					
Disease category, disease, intervention	Cost ($)	OOP (%)	OOP ($)	Q1	Q2	Q3	Q4	Q5	Total
HC42 Acute pharyngitis treatment	$0.24	42.3%	$0.10	0.0 (0.0)	0.0 (0.0)	0.0 (0.0)	0.0 (0.0)	0.0 (0.0)	0.0 (0.0)
Infectious & parasitic diseases				0.0 (0.0)	0.0 (0.0)	0.0 (0.0)	0.0 (0.0)	0.0 (0.0)	0.0 (0.0)
Diarrheal diseases				0.3 (0.0)	0.0 (0.0)	0.0 (0.0)	0.0 (0.0)	0.0 (0.0)	0.1 (0.0)
HC12 Diagnosis & treatment of infections (IMCI)	$10.29	50.3%	$5.18	0.3 (0.0)	0.0 (0.0)	0.0 (0.0)	0.0 (0.0)	0.0 (0.0)	0.1 (0.0)
HIV/AIDS & other sexually transmitted diseases				0.0 (0.0)	0.0 (0.0)	0.0 (0.0)	0.0 (0.0)	0.0 (0.0)	0.0 (0.0)
HC13 ART & viral load monitoring	$121.57	0.9%	$1.14	0.0 (0.0)	0.0 (0.0)	0.0 (0.0)	0.0 (0.0)	0.0 (0.0)	0.0 (0.0)
HC17 Syndromic management of STI	$10.69	0.9%	$0.10	0.0 (0.0)	0.0 (0.0)	0.0 (0.0)	0.0 (0.0)	0.0 (0.0)	0.0 (0.0)
HC23 HIV, STIs, hepatitis testing & counseling	$6.08	0.9%	$0.06	0.0 (0.0)	0.0 (0.0)	0.0 (0.0)	0.0 (0.0)	0.0 (0.0)	0.0 (0.0)
HC8 HIV & syphilis PMTCT	$313.51	0.9%	$2.94	0.1 (0.0)	0.0 (0.0)	0.0 (0.0)	0.0 (0.0)	0.0 (0.0)	0.0 (0.0)
Malaria				0.0 (0.0)	0.0 (0.0)	0.0 (0.0)	0.0 (0.0)	0.0 (0.0)	0.0 (0.0)
C7 Intermittent preventive treatment (pregnancy)	$1.02	30.4%	$0.31	0.0 (0.0)	0.0 (0.0)	0.0 (0.0)	0.0 (0.0)	0.0 (0.0)	0.0 (0.0)
Tuberculosis				0.0 (0.0)	0.0 (0.0)	0.0 (0.0)	0.0 (0.0)	0.0 (0.0)	0.0 (0.0)
HC27 Diagnosis & treatment of TB	$175.65	0.6%	$1.12	0.0 (0.0)	0.0 (0.0)	0.0 (0.0)	0.0 (0.0)	0.0 (0.0)	0.0 (0.0)
Other infectious & parasitic diseases				0.0 (0.0)	0.0 (0.0)	0.0 (0.0)	0.0 (0.0)	0.0 (0.0)	0.0 (0.0)
HC30 Management & referrals for fever (IMAI)	$6.83	25.6%	$1.75	0.0 (0.0)	0.0 (0.0)	0.0 (0.0)	0.0 (0.0)	0.0 (0.0)	0.0 (0.0)
Noncommunicable diseases (NCDs)				18.3 (11.1)	19.4 (1.5)	7.8 (0.0)	3.5 (0.0)	0.0 (0.0)	9.8 (2.5)
Cardiovascular diseases				21.1 (16.1)	23.3 (4.9)	14.7 (0.0)	11.6 (0.0)	0.0 (0.0)	14.1 (4.2)
HC38 Aspirin for acute myocardial infarction	$0.05	70.1%	$0.04	0.0 (0.0)	0.0 (0.0)	0.0 (0.0)	0.0 (0.0)	0.0 (0.0)	0.0 (0.0)
HC43 Management of ischemic heart disease	$190.17	70.1%	$133.39	31.7 (16.8)	35.1 (0.0)	7.8 (0.0)	0.0 (0.0)	0.0 (0.0)	14.9 (3.4)
HC44 Management of heart failure	$342.48	70.1%	$240.22	31.5 (31.5)	34.9 (14.6)	36.4 (0.0)	34.8 (0.0)	0.0 (0.0)	27.5 (9.2)
Endocrine & metabolic disorders				1.4 (0.2)	0.0 (0.0)	0.0 (0.0)	0.0 (0.0)	0.0 (0.0)	0.3 (0.0)
HC40 Screening & management of diabetes	$92.52	46.3%	$42.79	1.4 (0.2)	0.0 (0.0)	0.0 (0.0)	0.0 (0.0)	0.0 (0.0)	0.3 (0.0)
Mental/behavioral disorders & neurological conditions				23.6 (12.4)	24.7 (0.0)	6.7 (0.0)	0.0 (0.0)	0.0 (0.0)	11.0 (2.5)
HC49 Management of bipolar disorder	$365.39	39.0%	$142.59	55.8 (33.5)	61.8 (0.0)	25.9 (0.0)	0.0 (0.0)	0.0 (0.0)	28.7 (6.7)
HC50 Management of depression	$48.02	39.0%	$18.74	3.6 (0.5)	0.0 (0.0)	0.0 (0.0)	0.0 (0.0)	0.0 (0.0)	0.7 (0.1)
HC51 Management of epilepsy	$53.73	39.0%	$20.97	1.9 (0.3)	0.0 (0.0)	0.0 (0.0)	0.0 (0.0)	0.0 (0.0)	0.4 (0.1)
HC52 Management of schizophrenia	$329.34	39.0%	$128.52	56.2 (27.7)	61.8 (0.0)	7.5 (0.0)	0.0 (0.0)	0.0 (0.0)	25.1 (5.5)
HC66 Psychosocial support & counseling	$21.32	39.0%	$8.32	0.6 (0.1)	0.0 (0.0)	0.0 (0.0)	0.0 (0.0)	0.0 (0.0)	0.1 (0.0)
Other NCDs				0.0 (0.0)	0.0 (0.0)	0.0 (0.0)	0.0 (0.0)	0.0 (0.0)	0.0 (0.0)
HC47 Palliative care	$21.32	70.3%	$15.00	0.0 (0.0)	0.0 (0.0)	0.0 (0.0)	0.0 (0.0)	0.0 (0.0)	0.0 (0.0)
Reproductive health				5.2 (1.6)	3.2 (0.0)	0.0 (0.0)	0.0 (0.0)	0.0 (0.0)	1.7 (0.3)
Family planning				0.0 (0.0)	0.0 (0.0)	0.0 (0.0)	0.0 (0.0)	0.0 (0.0)	0.0 (0.0)
HC4 Contraceptives	$10.32	22.9%	$2.36	0.0 (0.0)	0.0 (0.0)	0.0 (0.0)	0.0 (0.0)	0.0 (0.0)	0.0 (0.0)
Maternal conditions				8.5 (2.6)	5.3 (0.0)	0.0 (0.0)	0.0 (0.0)	0.0 (0.0)	2.8 (0.5)
C5 Antenatal tetanus immunization	$0.44	68.9%	$0.30	0.0 (0.0)	0.0 (0.0)	0.0 (0.0)	0.0 (0.0)	0.0 (0.0)	0.0 (0.0)
HC11Basic emergency newborn & obstetric care	$145.10	68.9%	$100.02	50.6 (15.7)	31.9 (0.0)	0.0 (0.0)	0.0 (0.0)	0.0 (0.0)	16.5 (3.1)
HC2 Post-abortion care	$8.23	68.9%	$5.67	0.2 (0.0)	0.0 (0.0)	0.0 (0.0)	0.0 (0.0)	0.0 (0.0)	0.0 (0.0)
HC3 Treatment of premature membrane rupture	$4.66	68.9%	$3.22	0.1 (0.0)	0.0 (0.0)	0.0 (0.0)	0.0 (0.0)	0.0 (0.0)	0.0 (0.0)
HC5 Kangaroo mother care counseling	$4.61	68.9%	$3.18	0.1 (0.0)	0.0 (0.0)	0.0 (0.0)	0.0 (0.0)	0.0 (0.0)	0.0 (0.0)
HC7 Medical abortion	$5.52	68.9%	$3.80	0.2 (0.0)	0.0 (0.0)	0.0 (0.0)	0.0 (0.0)	0.0 (0.0)	0.0 (0.0)

Ghana				CHE risk 10% threshold (25% threshold)					
Disease category, disease, intervention	Cost ($)	OOP (%)	OOP ($)	Q1	Q2	Q3	Q4	Q5	Total
Perinatal conditions				0.2 (0.0)	0.0 (0.0)	0.0 (0.0)	0.0 (0.0)	0.0 (0.0)	0.0 (0.0)
C13 Cotrimoxazole for HIV-exposed children	$11.95	77.3%	$9.23	0.5 (0.1)	0.0 (0.0)	0.0 (0.0)	0.0 (0.0)	0.0 (0.0)	0.1 (0.0)
HC1 Antibiotics for neonatal pneumonia	$6.61	77.3%	$5.11	0.1 (0.0)	0.0 (0.0)	0.0 (0.0)	0.0 (0.0)	0.0 (0.0)	0.0 (0.0)
HC6 Neonatal sepsis, pneumonia, meningitis	$3.51	77.3%	$2.71	0.0 (0.0)	0.0 (0.0)	0.0 (0.0)	0.0 (0.0)	0.0 (0.0)	0.0 (0.0)

Table B.15. Full model results: Guinea.

Guinea				CHE risk 10% threshold (25% threshold)					
Disease category, disease, intervention	Cost ($)	OOP (%)	OOP ($)	Q1	Q2	Q3	Q4	Q5	Total
Childhood health				0.0 (0.0)	0.0 (0.0)	0.0 (0.0)	0.0 (0.0)	0.0 (0.0)	0.0 (0.0)
Childhood health				0.0 (0.0)	0.0 (0.0)	0.0 (0.0)	0.0 (0.0)	0.0 (0.0)	0.0 (0.0)
HC42 Acute pharyngitis treatment	$0.17	42.3%	$0.07	0.0 (0.0)	0.0 (0.0)	0.0 (0.0)	0.0 (0.0)	0.0 (0.0)	0.0 (0.0)
Infectious & parasitic diseases				12.0 (8.7)	15.3 (2.6)	16.2 (0.0)	9.7 (0.0)	3.4 (0.0)	11.3 (2.3)
Diarrheal diseases				0.1 (0.0)	0.0 (0.0)	0.0 (0.0)	0.0 (0.0)	0.0 (0.0)	0.0 (0.0)
HC12 Diagnosis & treatment of infections (IMCI)	$4.79	74.8%	$3.58	0.1 (0.0)	0.0 (0.0)	0.0 (0.0)	0.0 (0.0)	0.0 (0.0)	0.0 (0.0)
HIV/AIDS & other sexually transmitted diseases				12.0 (10.8)	14.8 (5.3)	15.8 (0.0)	19.2 (0.0)	6.8 (0.0)	13.7 (3.2)
HC13 ART & viral load monitoring	$71.45	58.7%	$41.95	5.6 (0.9)	3.0 (0.0)	0.0 (0.0)	0.0 (0.0)	0.0 (0.0)	1.7 (0.2)
HC17 Syndromic management of STI	$5.67	58.7%	$3.33	0.0 (0.0)	0.0 (0.0)	0.0 (0.0)	0.0 (0.0)	0.0 (0.0)	0.0 (0.0)
HC23 HIV, STIs, hepatitis testing & counseling	$4.31	58.7%	$2.53	0.0 (0.0)	0.0 (0.0)	0.0 (0.0)	0.0 (0.0)	0.0 (0.0)	0.0 (0.0)
HC8 HIV & syphilis PMTCT	$176.35	58.7%	$103.55	42.4 (42.4)	56.4 (21.1)	63.2 (0.0)	76.8 (0.0)	27.3 (0.0)	53.2 (12.7)
Malaria				0.0 (0.0)	0.0 (0.0)	0.0 (0.0)	0.0 (0.0)	0.0 (0.0)	0.0 (0.0)
C7 Intermittent preventive treatment (pregnancy)	$0.45	51.3%	$0.23	0.0 (0.0)	0.0 (0.0)	0.0 (0.0)	0.0 (0.0)	0.0 (0.0)	0.0 (0.0)
Tuberculosis				47.7 (25.9)	63.3 (0.0)	66.4 (0.0)	1.1 (0.0)	0.0 (0.0)	35.7 (5.2)
HC27 Diagnosis & treatment of TB	$135.09	49.8%	$67.29	47.7 (25.9)	63.3 (0.0)	66.4 (0.0)	1.1 (0.0)	0.0 (0.0)	35.7 (5.2)
Other infectious & parasitic diseases				0.0 (0.0)	0.0 (0.0)	0.0 (0.0)	0.0 (0.0)	0.0 (0.0)	0.0 (0.0)
HC30 Management & referrals for fever (IMAI)	$3.11	52.5%	$1.64	0.0 (0.0)	0.0 (0.0)	0.0 (0.0)	0.0 (0.0)	0.0 (0.0)	0.0 (0.0)
Noncommunicable diseases (NCDs)				14.6 (6.8)	13.2 (2.0)	7.7 (0.5)	8.8 (0.0)	2.3 (0.0)	9.3 (1.8)
Cardiovascular diseases				4.3 (2.7)	5.5 (2.8)	3.2 (1.7)	3.9 (0.0)	3.9 (0.0)	4.2 (1.4)
HC38 Aspirin for acute myocardial infarction	$0.03	59.8%	$0.02	0.0 (0.0)	0.0 (0.0)	0.0 (0.0)	0.0 (0.0)	0.0 (0.0)	0.0 (0.0)
HC43 Management of ischemic heart disease	$83.97	59.8%	$50.24	6.5 (1.7)	7.9 (0.0)	0.1 (0.0)	0.0 (0.0)	0.0 (0.0)	2.9 (0.3)
HC44 Management of heart failure	$249.96	59.8%	$149.56	6.4 (6.4)	8.5 (8.5)	9.6 (5.1)	11.6 (0.0)	11.8 (0.0)	9.6 (4.0)
Endocrine & metabolic disorders				7.0 (1.1)	3.1 (0.0)	0.0 (0.0)	0.0 (0.0)	0.0 (0.0)	2.0 (0.2)
HC40 Screening & management of diabetes	$64.16	63.7%	$40.85	7.0 (1.1)	3.1 (0.0)	0.0 (0.0)	0.0 (0.0)	0.0 (0.0)	2.0 (0.2)
Mental/behavioral disorders & neurological conditions				25.3 (11.7)	22.4 (2.2)	13.4 (0.0)	15.3 (0.0)	2.2 (0.0)	15.7 (2.8)
HC49 Management of bipolar disorder	$184.57	52.2%	$96.41	42.7 (42.7)	56.4 (11.1)	63.2 (0.0)	76.6 (0.0)	11.0 (0.0)	50.0 (10.7)
HC50 Management of depression	$16.11	52.2%	$8.42	0.9 (0.1)	0.0 (0.0)	0.0 (0.0)	0.0 (0.0)	0.0 (0.0)	0.2 (0.0)
HC51 Management of epilepsy	$27.53	52.2%	$14.38	1.9 (0.1)	0.0 (0.0)	0.0 (0.0)	0.0 (0.0)	0.0 (0.0)	0.4 (0.0)
HC52 Management of schizophrenia	$99.43	52.2%	$51.94	42.5 (12.0)	55.7 (0.0)	3.9 (0.0)	0.0 (0.0)	0.0 (0.0)	20.4 (2.4)
HC66 Psychosocial support & counseling	$64.63	52.2%	$33.76	38.5 (3.7)	0.1 (0.0)	0.0 (0.0)	0.0 (0.0)	0.0 (0.0)	7.7 (0.7)

Guinea				CHE risk 10% threshold (25% threshold)					
Disease category, disease, intervention	Cost ($)	OOP (%)	OOP ($)	Q1	Q2	Q3	Q4	Q5	Total
Other NCDs				0.1 (0.0)	0.0 (0.0)	0.0 (0.0)	0.0 (0.0)	0.0 (0.0)	0.0 (0.0)
HC47 Palliative care	$64.63	37.2%	$24.06	0.1 (0.0)	0.0 (0.0)	0.0 (0.0)	0.0 (0.0)	0.0 (0.0)	0.0 (0.0)
Reproductive health				2.8 (0.3)	0.3 (0.0)	0.0 (0.0)	0.0 (0.0)	0.0 (0.0)	0.6 (0.1)
Family planning				0.0 (0.0)	0.0 (0.0)	0.0 (0.0)	0.0 (0.0)	0.0 (0.0)	0.0 (0.0)
HC4 Contraceptives	$4.97	22.9%	$1.14	0.0 (0.0)	0.0 (0.0)	0.0 (0.0)	0.0 (0.0)	0.0 (0.0)	0.0 (0.0)
Maternal conditions				4.7 (0.5)	0.5 (0.0)	0.0 (0.0)	0.0 (0.0)	0.0 (0.0)	1.0 (0.1)
C5 Antenatal tetanus immunization	$0.39	52.6%	$0.20	0.0 (0.0)	0.0 (0.0)	0.0 (0.0)	0.0 (0.0)	0.0 (0.0)	0.0 (0.0)
HC11Basic emergency newborn & obstetric care	$69.14	52.6%	$36.34	28.3 (3.1)	2.9 (0.0)	0.0 (0.0)	0.0 (0.0)	0.0 (0.0)	6.2 (0.6)
HC2 Post-abortion care	$4.54	52.6%	$2.39	0.0 (0.0)	0.0 (0.0)	0.0 (0.0)	0.0 (0.0)	0.0 (0.0)	0.0 (0.0)
HC3 Treatment of premature membrane rupture	$4.04	52.6%	$2.12	0.0 (0.0)	0.0 (0.0)	0.0 (0.0)	0.0 (0.0)	0.0 (0.0)	0.0 (0.0)
HC5 Kangaroo mother care counseling	$2.33	52.6%	$1.22	0.0 (0.0)	0.0 (0.0)	0.0 (0.0)	0.0 (0.0)	0.0 (0.0)	0.0 (0.0)
HC7 Medical abortion	$4.81	52.6%	$2.53	0.0 (0.0)	0.0 (0.0)	0.0 (0.0)	0.0 (0.0)	0.0 (0.0)	0.0 (0.0)
Perinatal conditions				0.0 (0.0)	0.0 (0.0)	0.0 (0.0)	0.0 (0.0)	0.0 (0.0)	0.0 (0.0)
C13 Cotrimoxazole for HIV-exposed children	$6.55	53.1%	$3.48	0.0 (0.0)	0.0 (0.0)	0.0 (0.0)	0.0 (0.0)	0.0 (0.0)	0.0 (0.0)
HC1 Antibiotics for neonatal pneumonia	$6.22	53.1%	$3.30	0.0 (0.0)	0.0 (0.0)	0.0 (0.0)	0.0 (0.0)	0.0 (0.0)	0.0 (0.0)
HC6 Neonatal sepsis, pneumonia, meningitis	$2.66	53.1%	$1.41	0.0 (0.0)	0.0 (0.0)	0.0 (0.0)	0.0 (0.0)	0.0 (0.0)	0.0 (0.0)

Table B.16. Full model results: Haiti.

Haiti				CHE risk 10% threshold (25% threshold)					
Disease category, disease, intervention	Cost ($)	OOP (%)	OOP ($)	Q1	Q2	Q3	Q4	Q5	Total
Childhood health				0.0 (0.0)	0.0 (0.0)	0.0 (0.0)	0.0 (0.0)	0.0 (0.0)	0.0 (0.0)
Childhood health				0.0 (0.0)	0.0 (0.0)	0.0 (0.0)	0.0 (0.0)	0.0 (0.0)	0.0 (0.0)
HC42 Acute pharyngitis treatment	$0.24	45.3%	$0.11	0.0 (0.0)	0.0 (0.0)	0.0 (0.0)	0.0 (0.0)	0.0 (0.0)	0.0 (0.0)
Infectious & parasitic diseases				0.1 (0.0)	0.0 (0.0)	0.0 (0.0)	0.0 (0.0)	0.0 (0.0)	0.0 (0.0)
Diarrheal diseases				0.4 (0.0)	0.0 (0.0)	0.0 (0.0)	0.0 (0.0)	0.0 (0.0)	0.1 (0.0)
HC12 Diagnosis & treatment of infections (IMCI)	$10.29	73.2%	$7.53	0.4 (0.0)	0.0 (0.0)	0.0 (0.0)	0.0 (0.0)	0.0 (0.0)	0.1 (0.0)
HIV/AIDS & other sexually transmitted diseases				0.1 (0.0)	0.0 (0.0)	0.0 (0.0)	0.0 (0.0)	0.0 (0.0)	0.0 (0.0)
HC13 ART & viral load monitoring	$121.57	1.9%	$2.29	0.0 (0.0)	0.0 (0.0)	0.0 (0.0)	0.0 (0.0)	0.0 (0.0)	0.0 (0.0)
HC17 Syndromic management of STI	$10.69	1.9%	$0.20	0.0 (0.0)	0.0 (0.0)	0.0 (0.0)	0.0 (0.0)	0.0 (0.0)	0.0 (0.0)
HC23 HIV, STIs, hepatitis testing & counseling	$6.08	1.9%	$0.11	0.0 (0.0)	0.0 (0.0)	0.0 (0.0)	0.0 (0.0)	0.0 (0.0)	0.0 (0.0)
HC8 HIV & syphilis PMTCT	$313.51	1.9%	$5.89	0.5 (0.0)	0.0 (0.0)	0.0 (0.0)	0.0 (0.0)	0.0 (0.0)	0.1 (0.0)
Malaria				0.0 (0.0)	0.0 (0.0)	0.0 (0.0)	0.0 (0.0)	0.0 (0.0)	0.0 (0.0)
C7 Intermittent preventive treatment (pregnancy)	$1.02	21.2%	$0.22	0.0 (0.0)	0.0 (0.0)	0.0 (0.0)	0.0 (0.0)	0.0 (0.0)	0.0 (0.0)
Tuberculosis				0.0 (0.0)	0.0 (0.0)	0.0 (0.0)	0.0 (0.0)	0.0 (0.0)	0.0 (0.0)
HC27 Diagnosis & treatment of TB	$175.65	1.2%	$2.14	0.0 (0.0)	0.0 (0.0)	0.0 (0.0)	0.0 (0.0)	0.0 (0.0)	0.0 (0.0)
Other infectious & parasitic diseases				0.0 (0.0)	0.0 (0.0)	0.0 (0.0)	0.0 (0.0)	0.0 (0.0)	0.0 (0.0)
HC30 Management & referrals for fever (IMAI)	$6.83	17.2%	$1.18	0.0 (0.0)	0.0 (0.0)	0.0 (0.0)	0.0 (0.0)	0.0 (0.0)	0.0 (0.0)
Noncommunicable diseases (NCDs)				4.3 (3.6)	4.6 (2.2)	5.3 (0.6)	3.6 (0.0)	2.0 (0.0)	4.0 (1.3)

Haiti				CHE risk 10% threshold (25% threshold)					
Disease category, disease, intervention	Cost ($)	OOP (%)	OOP ($)	Q1	Q2	Q3	Q4	Q5	Total
Cardiovascular diseases				12.1 (11.5)	14.6 (7.3)	17.8 (2.2)	12.0 (0.0)	6.8 (0.0)	12.7 (4.2)
HC38 Aspirin for acute myocardial infarction	$0.05	73.2%	$0.04	0.0 (0.0)	0.0 (0.0)	0.0 (0.0)	0.0 (0.0)	0.0 (0.0)	0.0 (0.0)
HC43 Management of ischemic heart disease	$190.17	73.2%	$139.19	18.1 (16.6)	21.9 (0.1)	26.6 (0.0)	6.3 (0.0)	0.0 (0.0)	14.6 (3.3)
HC44 Management of heart failure	$342.48	73.2%	$250.66	18.0 (18.0)	21.9 (21.9)	26.7 (6.5)	29.7 (0.0)	20.4 (0.0)	23.4 (9.3)
Endocrine & metabolic disorders				7.1 (1.7)	2.2 (0.0)	0.0 (0.0)	0.0 (0.0)	0.0 (0.0)	1.9 (0.3)
HC40 Screening & management of diabetes	$92.52	72.9%	$67.42	7.1 (1.7)	2.2 (0.0)	0.0 (0.0)	0.0 (0.0)	0.0 (0.0)	1.9 (0.3)
Mental/behavioral disorders & neurological conditions				0.0 (0.0)	0.0 (0.0)	0.0 (0.0)	0.0 (0.0)	0.0 (0.0)	0.0 (0.0)
HC49 Management of bipolar disorder	$365.39	0.4%	$1.43	0.0 (0.0)	0.0 (0.0)	0.0 (0.0)	0.0 (0.0)	0.0 (0.0)	0.0 (0.0)
HC50 Management of depression	$48.02	0.4%	$0.19	0.0 (0.0)	0.0 (0.0)	0.0 (0.0)	0.0 (0.0)	0.0 (0.0)	0.0 (0.0)
HC51 Management of epilepsy	$53.73	0.4%	$0.21	0.0 (0.0)	0.0 (0.0)	0.0 (0.0)	0.0 (0.0)	0.0 (0.0)	0.0 (0.0)
HC52 Management of schizophrenia	$329.34	0.4%	$1.29	0.0 (0.0)	0.0 (0.0)	0.0 (0.0)	0.0 (0.0)	0.0 (0.0)	0.0 (0.0)
HC66 Psychosocial support & counseling	$21.32	0.4%	$0.08	0.0 (0.0)	0.0 (0.0)	0.0 (0.0)	0.0 (0.0)	0.0 (0.0)	0.0 (0.0)
Other NCDs				0.0 (0.0)	0.0 (0.0)	0.0 (0.0)	0.0 (0.0)	0.0 (0.0)	0.0 (0.0)
HC47 Palliative care	$21.32	51.1%	$10.90	0.0 (0.0)	0.0 (0.0)	0.0 (0.0)	0.0 (0.0)	0.0 (0.0)	0.0 (0.0)
Reproductive health				0.1 (0.0)	0.0 (0.0)	0.0 (0.0)	0.0 (0.0)	0.0 (0.0)	0.0 (0.0)
Family planning				0.7 (0.1)	0.0 (0.0)	0.0 (0.0)	0.0 (0.0)	0.0 (0.0)	0.1 (0.0)
HC4 Contraceptives	$10.32	92.0%	$9.50	0.7 (0.1)	0.0 (0.0)	0.0 (0.0)	0.0 (0.0)	0.0 (0.0)	0.1 (0.0)
Maternal conditions				0.0 (0.0)	0.0 (0.0)	0.0 (0.0)	0.0 (0.0)	0.0 (0.0)	0.0 (0.0)
C5 Antenatal tetanus immunization	$0.44	1.6%	$0.01	0.0 (0.0)	0.0 (0.0)	0.0 (0.0)	0.0 (0.0)	0.0 (0.0)	0.0 (0.0)
HC11 Basic emergency newborn & obstetric care	$145.10	1.6%	$2.29	0.0 (0.0)	0.0 (0.0)	0.0 (0.0)	0.0 (0.0)	0.0 (0.0)	0.0 (0.0)
HC2 Post-abortion care	$8.23	1.6%	$0.13	0.0 (0.0)	0.0 (0.0)	0.0 (0.0)	0.0 (0.0)	0.0 (0.0)	0.0 (0.0)
HC3 Treatment of premature membrane rupture	$4.66	1.6%	$0.07	0.0 (0.0)	0.0 (0.0)	0.0 (0.0)	0.0 (0.0)	0.0 (0.0)	0.0 (0.0)
HC5 Kangaroo mother care counseling	$4.61	1.6%	$0.07	0.0 (0.0)	0.0 (0.0)	0.0 (0.0)	0.0 (0.0)	0.0 (0.0)	0.0 (0.0)
HC7 Medical abortion	$5.52	1.6%	$0.09	0.0 (0.0)	0.0 (0.0)	0.0 (0.0)	0.0 (0.0)	0.0 (0.0)	0.0 (0.0)
Perinatal conditions				0.1 (0.0)	0.0 (0.0)	0.0 (0.0)	0.0 (0.0)	0.0 (0.0)	0.0 (0.0)
C13 Cotrimoxazole for HIV-exposed children	$11.95	45.9%	$5.48	0.1 (0.0)	0.0 (0.0)	0.0 (0.0)	0.0 (0.0)	0.0 (0.0)	0.0 (0.0)
HC1 Antibiotics for neonatal pneumonia	$6.61	45.9%	$3.03	0.0 (0.0)	0.0 (0.0)	0.0 (0.0)	0.0 (0.0)	0.0 (0.0)	0.0 (0.0)
HC6 Neonatal sepsis, pneumonia, meningitis	$3.51	45.9%	$1.61	0.0 (0.0)	0.0 (0.0)	0.0 (0.0)	0.0 (0.0)	0.0 (0.0)	0.0 (0.0)

Table B.17. Full model results: Kenya.

Kenya				CHE risk 10% threshold (25% threshold)					
Disease category, disease, intervention	Cost ($)	OOP (%)	OOP ($)	Q1	Q2	Q3	Q4	Q5	Total
Childhood health				0.0 (0.0)	0.0 (0.0)	0.0 (0.0)	0.0 (0.0)	0.0 (0.0)	0.0 (0.0)
Childhood health				0.0 (0.0)	0.0 (0.0)	0.0 (0.0)	0.0 (0.0)	0.0 (0.0)	0.0 (0.0)
HC42 Acute pharyngitis treatment	$0.24	39.3%	$0.09	0.0 (0.0)	0.0 (0.0)	0.0 (0.0)	0.0 (0.0)	0.0 (0.0)	0.0 (0.0)
Infectious & parasitic diseases				2.6 (0.3)	0.0 (0.0)	0.0 (0.0)	0.0 (0.0)	0.0 (0.0)	0.5 (0.1)
Diarrheal diseases				0.1 (0.0)	0.0 (0.0)	0.0 (0.0)	0.0 (0.0)	0.0 (0.0)	0.0 (0.0)
HC12 Diagnosis & treatment of infections (IMCI)	$10.29	30.6%	$3.15	0.1 (0.0)	0.0 (0.0)	0.0 (0.0)	0.0 (0.0)	0.0 (0.0)	0.0 (0.0)

Kenya				CHE risk 10% threshold (25% threshold)					
Disease category, disease, intervention	Cost ($)	OOP (%)	OOP ($)	Q1	Q2	Q3	Q4	Q5	Total
HIV/AIDS & other sexually transmitted diseases				5.0 (0.6)	0.0 (0.0)	0.0 (0.0)	0.0 (0.0)	0.0 (0.0)	1.0 (0.1)
HC13 ART & viral load monitoring	$121.57	9.0%	$10.94	1.9 (0.2)	0.0 (0.0)	0.0 (0.0)	0.0 (0.0)	0.0 (0.0)	0.4 (0.0)
HC17 Syndromic management of STI	$10.69	9.0%	$0.96	0.0 (0.0)	0.0 (0.0)	0.0 (0.0)	0.0 (0.0)	0.0 (0.0)	0.0 (0.0)
HC23 HIV, STIs, hepatitis testing & counseling	$6.08	9.0%	$0.55	0.0 (0.0)	0.0 (0.0)	0.0 (0.0)	0.0 (0.0)	0.0 (0.0)	0.0 (0.0)
HC8 HIV & syphilis PMTCT	$313.51	9.0%	$28.22	18.0 (2.4)	0.0 (0.0)	0.0 (0.0)	0.0 (0.0)	0.0 (0.0)	3.6 (0.5)
Malaria				0.0 (0.0)	0.0 (0.0)	0.0 (0.0)	0.0 (0.0)	0.0 (0.0)	0.0 (0.0)
C7 Intermittent preventive treatment (pregnancy)	$1.02	21.5%	$0.22	0.0 (0.0)	0.0 (0.0)	0.0 (0.0)	0.0 (0.0)	0.0 (0.0)	0.0 (0.0)
Tuberculosis				0.5 (0.1)	0.0 (0.0)	0.0 (0.0)	0.0 (0.0)	0.0 (0.0)	0.1 (0.0)
HC27 Diagnosis & treatment of TB	$175.65	3.9%	$6.85	0.5 (0.1)	0.0 (0.0)	0.0 (0.0)	0.0 (0.0)	0.0 (0.0)	0.1 (0.0)
Other infectious & parasitic diseases				0.0 (0.0)	0.0 (0.0)	0.0 (0.0)	0.0 (0.0)	0.0 (0.0)	0.0 (0.0)
HC30 Management & referrals for fever (IMAI)	$6.83	16.7%	$1.14	0.0 (0.0)	0.0 (0.0)	0.0 (0.0)	0.0 (0.0)	0.0 (0.0)	0.0 (0.0)
Noncommunicable diseases (NCDs)				4.3 (3.2)	4.2 (1.2)	3.7 (0.0)	3.1 (0.0)	0.1 (0.0)	3.1 (0.9)
Cardiovascular diseases				5.5 (4.3)	6.5 (1.7)	4.7 (0.0)	3.8 (0.0)	0.2 (0.0)	4.2 (1.2)
HC38 Aspirin for acute myocardial infarction	$0.05	56.5%	$0.03	0.0 (0.0)	0.0 (0.0)	0.0 (0.0)	0.0 (0.0)	0.0 (0.0)	0.0 (0.0)
HC43 Management of ischemic heart disease	$190.17	56.5%	$107.52	8.3 (4.5)	9.8 (0.0)	3.9 (0.0)	0.0 (0.0)	0.0 (0.0)	4.4 (0.9)
HC44 Management of heart failure	$342.48	56.5%	$193.63	8.4 (8.4)	9.8 (5.1)	10.1 (0.0)	11.3 (0.0)	0.7 (0.0)	8.1 (2.7)
Endocrine & metabolic disorders				3.0 (0.5)	0.0 (0.0)	0.0 (0.0)	0.0 (0.0)	0.0 (0.0)	0.6 (0.1)
HC40 Screening & management of diabetes	$92.52	53.9%	$49.86	3.0 (0.5)	0.0 (0.0)	0.0 (0.0)	0.0 (0.0)	0.0 (0.0)	0.6 (0.1)
Mental/behavioral disorders & neurological conditions				4.5 (3.8)	4.4 (1.3)	4.6 (0.0)	4.0 (0.0)	0.0 (0.0)	3.5 (1.0)
HC49 Management of bipolar disorder	$365.39	50.5%	$184.49	9.2 (9.2)	10.9 (4.5)	11.4 (0.0)	11.8 (0.0)	0.1 (0.0)	8.7 (2.7)
HC50 Management of depression	$48.02	50.5%	$24.25	1.6 (0.2)	0.0 (0.0)	0.0 (0.0)	0.0 (0.0)	0.0 (0.0)	0.3 (0.0)
HC51 Management of epilepsy	$53.73	50.5%	$27.13	2.6 (0.3)	0.0 (0.0)	0.0 (0.0)	0.0 (0.0)	0.0 (0.0)	0.5 (0.1)
HC52 Management of schizophrenia	$329.34	50.5%	$166.29	9.1 (9.1)	11.0 (2.1)	11.4 (0.0)	8.3 (0.0)	0.0 (0.0)	8.0 (2.2)
HC66 Psychosocial support & counseling	$21.32	50.5%	$10.76	0.3 (0.0)	0.0 (0.0)	0.0 (0.0)	0.0 (0.0)	0.0 (0.0)	0.1 (0.0)
Other NCDs				0.3 (0.0)	0.0 (0.0)	0.0 (0.0)	0.0 (0.0)	0.0 (0.0)	0.1 (0.0)
HC47 Palliative care	$21.32	57.7%	$12.31	0.3 (0.0)	0.0 (0.0)	0.0 (0.0)	0.0 (0.0)	0.0 (0.0)	0.1 (0.0)
Reproductive health				1.9 (0.3)	0.0 (0.0)	0.0 (0.0)	0.0 (0.0)	0.0 (0.0)	0.4 (0.1)
Family planning				0.0 (0.0)	0.0 (0.0)	0.0 (0.0)	0.0 (0.0)	0.0 (0.0)	0.0 (0.0)
HC4 Contraceptives	$10.32	0.3%	$0.03	0.0 (0.0)	0.0 (0.0)	0.0 (0.0)	0.0 (0.0)	0.0 (0.0)	0.0 (0.0)
Maternal conditions				3.2 (0.5)	0.0 (0.0)	0.0 (0.0)	0.0 (0.0)	0.0 (0.0)	0.6 (0.1)
C5 Antenatal tetanus immunization	$0.44	29.9%	$0.13	0.0 (0.0)	0.0 (0.0)	0.0 (0.0)	0.0 (0.0)	0.0 (0.0)	0.0 (0.0)
HC11 Basic emergency newborn & obstetric care	$145.10	29.9%	$43.36	19.0 (2.8)	0.0 (0.0)	0.0 (0.0)	0.0 (0.0)	0.0 (0.0)	3.8 (0.6)
HC2 Post-abortion care	$8.23	29.9%	$2.46	0.0 (0.0)	0.0 (0.0)	0.0 (0.0)	0.0 (0.0)	0.0 (0.0)	0.0 (0.0)
HC3 Treatment of premature membrane rupture	$4.66	29.9%	$1.39	0.0 (0.0)	0.0 (0.0)	0.0 (0.0)	0.0 (0.0)	0.0 (0.0)	0.0 (0.0)
HC5 Kangaroo mother care counseling	$4.61	29.9%	$1.38	0.0 (0.0)	0.0 (0.0)	0.0 (0.0)	0.0 (0.0)	0.0 (0.0)	0.0 (0.0)
HC7 Medical abortion	$5.52	29.9%	$1.65	0.0 (0.0)	0.0 (0.0)	0.0 (0.0)	0.0 (0.0)	0.0 (0.0)	0.0 (0.0)
Perinatal conditions				0.1 (0.0)	0.0 (0.0)	0.0 (0.0)	0.0 (0.0)	0.0 (0.0)	0.0 (0.0)
C13 Cotrimoxazole for HIV-exposed children	$11.95	38.6%	$4.61	0.2 (0.0)	0.0 (0.0)	0.0 (0.0)	0.0 (0.0)	0.0 (0.0)	0.0 (0.0)
HC1 Antibiotics for neonatal pneumonia	$6.61	38.6%	$2.55	0.0 (0.0)	0.0 (0.0)	0.0 (0.0)	0.0 (0.0)	0.0 (0.0)	0.0 (0.0)
HC6 Neonatal sepsis, pneumonia, meningitis	$3.51	38.6%	$1.35	0.0 (0.0)	0.0 (0.0)	0.0 (0.0)	0.0 (0.0)	0.0 (0.0)	0.0 (0.0)

Table B.18. Full model results: Lao People's Democratic Republic.

**Lao People's Democratic Republic**				**CHE risk** 10% threshold (25% threshold)					
Disease category, disease, intervention	Cost ($)	OOP (%)	OOP ($)	Q1	Q2	Q3	Q4	Q5	Total
Childhood health				0.0 (0.0)	0.0 (0.0)	0.0 (0.0)	0.0 (0.0)	0.0 (0.0)	0.0 (0.0)
Childhood health				0.0 (0.0)	0.0 (0.0)	0.0 (0.0)	0.0 (0.0)	0.0 (0.0)	0.0 (0.0)
HC42 Acute pharyngitis treatment	$0.24	45.3%	$0.11	0.0 (0.0)	0.0 (0.0)	0.0 (0.0)	0.0 (0.0)	0.0 (0.0)	0.0 (0.0)
Infectious & parasitic diseases				4.9 (0.6)	0.0 (0.0)	0.0 (0.0)	0.0 (0.0)	0.0 (0.0)	1.0 (0.1)
Diarrheal diseases				0.3 (0.0)	0.0 (0.0)	0.0 (0.0)	0.0 (0.0)	0.0 (0.0)	0.1 (0.0)
HC12 Diagnosis & treatment of infections (IMCI)	$10.29	59.2%	$6.10	0.3 (0.0)	0.0 (0.0)	0.0 (0.0)	0.0 (0.0)	0.0 (0.0)	0.1 (0.0)
HIV/AIDS & other sexually transmitted diseases				0.3 (0.0)	0.0 (0.0)	0.0 (0.0)	0.0 (0.0)	0.0 (0.0)	0.1 (0.0)
HC13 ART & viral load monitoring	$121.57	4.5%	$5.43	0.0 (0.0)	0.0 (0.0)	0.0 (0.0)	0.0 (0.0)	0.0 (0.0)	0.0 (0.0)
HC17 Syndromic management of STI	$10.69	4.5%	$0.48	0.0 (0.0)	0.0 (0.0)	0.0 (0.0)	0.0 (0.0)	0.0 (0.0)	0.0 (0.0)
HC23 HIV, STIs, hepatitis testing & counseling	$6.08	4.5%	$0.27	0.0 (0.0)	0.0 (0.0)	0.0 (0.0)	0.0 (0.0)	0.0 (0.0)	0.0 (0.0)
HC8 HIV & syphilis PMTCT	$313.51	4.5%	$13.99	1.2 (0.1)	0.0 (0.0)	0.0 (0.0)	0.0 (0.0)	0.0 (0.0)	0.2 (0.0)
Malaria				0.0 (0.0)	0.0 (0.0)	0.0 (0.0)	0.0 (0.0)	0.0 (0.0)	0.0 (0.0)
C7 Intermittent preventive treatment (pregnancy)	$1.02	5.9%	$0.06	0.0 (0.0)	0.0 (0.0)	0.0 (0.0)	0.0 (0.0)	0.0 (0.0)	0.0 (0.0)
Tuberculosis				37.9 (5.0)	0.0 (0.0)	0.0 (0.0)	0.0 (0.0)	0.0 (0.0)	7.6 (1.0)
HC27 Diagnosis & treatment of TB	$175.65	30.1%	$52.85	37.9 (5.0)	0.0 (0.0)	0.0 (0.0)	0.0 (0.0)	0.0 (0.0)	7.6 (1.0)
Other infectious & parasitic diseases				0.0 (0.0)	0.0 (0.0)	0.0 (0.0)	0.0 (0.0)	0.0 (0.0)	0.0 (0.0)
HC30 Management & referrals for fever (IMAI)	$6.83	32.7%	$2.24	0.0 (0.0)	0.0 (0.0)	0.0 (0.0)	0.0 (0.0)	0.0 (0.0)	0.0 (0.0)
Noncommunicable diseases (NCDs)				11.7 (10.0)	12.0 (9.5)	12.9 (1.2)	12.4 (0.0)	8.2 (0.0)	11.5 (4.1)
Cardiovascular diseases				11.7 (10.3)	13.7 (6.8)	14.7 (0.8)	9.5 (0.0)	5.9 (0.0)	11.1 (3.6)
HC38 Aspirin for acute myocardial infarction	$0.05	68.8%	$0.03	0.0 (0.0)	0.0 (0.0)	0.0 (0.0)	0.0 (0.0)	0.0 (0.0)	0.0 (0.0)
HC43 Management of ischemic heart disease	$190.17	68.8%	$130.83	17.5 (13.5)	20.6 (0.0)	22.0 (0.0)	3.4 (0.0)	0.0 (0.0)	12.7 (2.7)
HC44 Management of heart failure	$342.48	68.8%	$235.61	17.5 (17.5)	20.6 (20.5)	22.2 (2.4)	25.0 (0.0)	17.8 (0.0)	20.6 (8.1)
Endocrine & metabolic disorders				3.3 (0.4)	0.1 (0.0)	0.0 (0.0)	0.0 (0.0)	0.0 (0.0)	0.7 (0.1)
HC40 Screening & management of diabetes	$92.52	64.3%	$59.49	3.3 (0.4)	0.1 (0.0)	0.0 (0.0)	0.0 (0.0)	0.0 (0.0)	0.7 (0.1)
Mental/behavioral disorders & neurological conditions				15.7 (13.7)	15.8 (15.0)	17.0 (1.8)	19.1 (0.0)	12.9 (0.0)	16.1 (6.1)
HC49 Management of bipolar disorder	$365.39	66.8%	$244.09	33.5 (33.5)	39.4 (39.4)	42.6 (9.0)	47.9 (0.0)	36.5 (0.0)	40.0 (16.4)
HC50 Management of depression	$48.02	66.8%	$32.08	9.1 (1.0)	0.0 (0.0)	0.0 (0.0)	0.0 (0.0)	0.0 (0.0)	1.8 (0.2)
HC51 Management of epilepsy	$53.73	66.8%	$35.89	0.9 (0.1)	0.0 (0.0)	0.0 (0.0)	0.0 (0.0)	0.0 (0.0)	0.2 (0.0)
HC52 Management of schizophrenia	$329.34	66.8%	$220.01	33.6 (33.6)	39.5 (35.5)	42.5 (0.2)	47.6 (0.0)	27.8 (0.0)	38.2 (13.9)
HC66 Psychosocial support & counseling	$21.32	66.8%	$14.24	1.3 (0.1)	0.0 (0.0)	0.0 (0.0)	0.0 (0.0)	0.0 (0.0)	0.3 (0.0)
Other NCDs				0.7 (0.1)	0.0 (0.0)	0.0 (0.0)	0.0 (0.0)	0.0 (0.0)	0.1 (0.0)
HC47 Palliative care	$21.32	97.6%	$20.81	0.7 (0.1)	0.0 (0.0)	0.0 (0.0)	0.0 (0.0)	0.0 (0.0)	0.1 (0.0)
Reproductive health				5.7 (1.3)	3.7 (0.0)	0.0 (0.0)	0.0 (0.0)	0.0 (0.0)	1.9 (0.3)
Family planning				0.1 (0.0)	0.0 (0.0)	0.0 (0.0)	0.0 (0.0)	0.0 (0.0)	0.0 (0.0)
HC4 Contraceptives	$10.32	48.0%	$4.95	0.1 (0.0)	0.0 (0.0)	0.0 (0.0)	0.0 (0.0)	0.0 (0.0)	0.0 (0.0)
Maternal conditions				9.4 (2.2)	6.1 (0.0)	0.0 (0.0)	0.0 (0.0)	0.0 (0.0)	3.1 (0.4)
C5 Antenatal tetanus immunization	$0.44	52.3%	$0.23	0.0 (0.0)	0.0 (0.0)	0.0 (0.0)	0.0 (0.0)	0.0 (0.0)	0.0 (0.0)
HC11 Basic emergency newborn & obstetric care	$145.10	52.3%	$75.91	56.1 (13.4)	36.7 (0.0)	0.0 (0.0)	0.0 (0.0)	0.0 (0.0)	18.6 (2.7)

Lao People's Democratic Republic				CHE risk 10% threshold (25% threshold)					
Disease category, disease, intervention	Cost ($)	OOP (%)	OOP ($)	Q1	Q2	Q3	Q4	Q5	Total
HC2 Post-abortion care	$8.23	52.3%	$4.31	0.1 (0.0)	0.0 (0.0)	0.0 (0.0)	0.0 (0.0)	0.0 (0.0)	0.0 (0.0)
HC3 Treatment of premature membrane rupture	$4.66	52.3%	$2.44	0.0 (0.0)	0.0 (0.0)	0.0 (0.0)	0.0 (0.0)	0.0 (0.0)	0.0 (0.0)
HC5 Kangaroo mother care counseling	$4.61	52.3%	$2.41	0.0 (0.0)	0.0 (0.0)	0.0 (0.0)	0.0 (0.0)	0.0 (0.0)	0.0 (0.0)
HC7 Medical abortion	$5.52	52.3%	$2.89	0.1 (0.0)	0.0 (0.0)	0.0 (0.0)	0.0 (0.0)	0.0 (0.0)	0.0 (0.0)
Perinatal conditions				0.0 (0.0)	0.0 (0.0)	0.0 (0.0)	0.0 (0.0)	0.0 (0.0)	0.0 (0.0)
C13 Cotrimoxazole for HIV-exposed children	$11.95	39.6%	$4.73	0.1 (0.0)	0.0 (0.0)	0.0 (0.0)	0.0 (0.0)	0.0 (0.0)	0.0 (0.0)
HC1 Antibiotics for neonatal pneumonia	$6.61	39.6%	$2.62	0.0 (0.0)	0.0 (0.0)	0.0 (0.0)	0.0 (0.0)	0.0 (0.0)	0.0 (0.0)
HC6 Neonatal sepsis, pneumonia, meningitis	$3.51	39.6%	$1.39	0.0 (0.0)	0.0 (0.0)	0.0 (0.0)	0.0 (0.0)	0.0 (0.0)	0.0 (0.0)

Table B.19. Full model results: Malawi.

Malawi				CHE risk 10% threshold (25% threshold)					
Disease category, disease, intervention	Cost ($)	OOP (%)	OOP ($)	Q1	Q2	Q3	Q4	Q5	Total
Childhood health				0.0 (0.0)	0.0 (0.0)	0.0 (0.0)	0.0 (0.0)	0.0 (0.0)	0.0 (0.0)
Childhood health				0.0 (0.0)	0.0 (0.0)	0.0 (0.0)	0.0 (0.0)	0.0 (0.0)	0.0 (0.0)
HC42 Acute pharyngitis treatment	$0.17	39.3%	$0.07	0.0 (0.0)	0.0 (0.0)	0.0 (0.0)	0.0 (0.0)	0.0 (0.0)	0.0 (0.0)
Infectious & parasitic diseases				22.1 (5.2)	8.0 (0.0)	0.0 (0.0)	0.0 (0.0)	0.0 (0.0)	6.0 (1.0)
Diarrheal diseases				0.0 (0.0)	0.0 (0.0)	0.0 (0.0)	0.0 (0.0)	0.0 (0.0)	0.0 (0.0)
HC12 Diagnosis & treatment of infections (IMCI)	$4.79	6.9%	$0.33	0.0 (0.0)	0.0 (0.0)	0.0 (0.0)	0.0 (0.0)	0.0 (0.0)	0.0 (0.0)
HIV/AIDS & other sexually transmitted diseases				28.9 (5.8)	6.8 (0.0)	0.0 (0.0)	0.0 (0.0)	0.0 (0.0)	7.2 (1.2)
HC13 ART & viral load monitoring	$71.45	9.7%	$6.97	16.7 (2.1)	0.0 (0.0)	0.0 (0.0)	0.0 (0.0)	0.0 (0.0)	3.3 (0.4)
HC17 Syndromic management of STI	$5.67	9.7%	$0.55	0.0 (0.0)	0.0 (0.0)	0.0 (0.0)	0.0 (0.0)	0.0 (0.0)	0.0 (0.0)
HC23 HIV, STIs, hepatitis testing & counseling	$4.31	9.7%	$0.42	0.0 (0.0)	0.0 (0.0)	0.0 (0.0)	0.0 (0.0)	0.0 (0.0)	0.0 (0.0)
HC8 HIV & syphilis PMTCT	$176.35	9.7%	$17.19	99.0 (21.2)	27.4 (0.0)	0.0 (0.0)	0.0 (0.0)	0.0 (0.0)	25.3 (4.2)
Malaria				0.0 (0.0)	0.0 (0.0)	0.0 (0.0)	0.0 (0.0)	0.0 (0.0)	0.0 (0.0)
C7 Intermittent preventive treatment (pregnancy)	$0.45	12.9%	$0.06	0.0 (0.0)	0.0 (0.0)	0.0 (0.0)	0.0 (0.0)	0.0 (0.0)	0.0 (0.0)
Tuberculosis				61.4 (18.1)	36.7 (0.0)	0.0 (0.0)	0.0 (0.0)	0.0 (0.0)	19.6 (3.6)
HC27 Diagnosis & treatment of TB	$135.09	14.7%	$19.89	61.4 (18.1)	36.7 (0.0)	0.0 (0.0)	0.0 (0.0)	0.0 (0.0)	19.6 (3.6)
Other infectious & parasitic diseases				0.0 (0.0)	0.0 (0.0)	0.0 (0.0)	0.0 (0.0)	0.0 (0.0)	0.0 (0.0)
HC30 Management & referrals for fever (IMAI)	$3.11	10.3%	$0.32	0.0 (0.0)	0.0 (0.0)	0.0 (0.0)	0.0 (0.0)	0.0 (0.0)	0.0 (0.0)
Noncommunicable diseases (NCDs)				8.4 (5.7)	5.4 (3.0)	5.8 (2.0)	4.3 (1.4)	2.4 (0.4)	5.3 (2.5)
Cardiovascular diseases				5.7 (5.7)	5.5 (4.1)	5.9 (2.9)	5.9 (2.9)	3.4 (1.5)	5.3 (3.4)
HC38 Aspirin for acute myocardial infarction	$0.03	56.5%	$0.02	0.0 (0.0)	0.0 (0.0)	0.0 (0.0)	0.0 (0.0)	0.0 (0.0)	0.0 (0.0)
HC43 Management of ischemic heart disease	$83.97	56.5%	$47.48	8.5 (8.5)	8.2 (4.3)	9.0 (0.0)	8.9 (0.0)	1.2 (0.0)	7.2 (2.5)
HC44 Management of heart failure	$249.96	56.5%	$141.32	8.7 (8.7)	8.2 (8.2)	8.8 (8.8)	8.8 (8.8)	9.1 (4.5)	8.7 (7.8)
Endocrine & metabolic disorders				5.6 (5.0)	5.3 (0.0)	5.6 (0.0)	1.5 (0.0)	0.0 (0.0)	3.6 (1.0)
HC40 Screening & management of diabetes	$64.16	53.9%	$34.58	5.6 (5.0)	5.3 (0.0)	5.6 (0.0)	1.5 (0.0)	0.0 (0.0)	3.6 (1.0)
Mental/behavioral disorders & neurological conditions				11.3 (6.9)	6.4 (3.5)	6.8 (2.3)	4.8 (1.1)	2.8 (0.0)	6.4 (2.8)
HC49 Management of bipolar disorder	$184.57	50.5%	$93.19	11.2 (11.2)	10.6 (10.6)	11.4 (11.4)	11.5 (5.5)	11.1 (0.0)	11.1 (7.7)

**Malawi**				**CHE risk** 10% threshold (25% threshold)					
Disease category, disease, intervention	Cost ($)	OOP (%)	OOP ($)	Q1	Q2	Q3	Q4	Q5	Total
HC50 Management of depression	$16.11	50.5%	$8.14	3.4 (0.4)	0.0 (0.0)	0.0 (0.0)	0.0 (0.0)	0.0 (0.0)	0.7 (0.1)
HC51 Management of epilepsy	$27.53	50.5%	$13.90	19.6 (3.0)	0.0 (0.0)	0.0 (0.0)	0.0 (0.0)	0.0 (0.0)	3.9 (0.6)
HC52 Management of schizophrenia	$99.43	50.5%	$50.20	11.1 (11.1)	10.6 (6.9)	11.5 (0.0)	11.4 (0.0)	3.0 (0.0)	9.5 (3.6)
HC66 Psychosocial support & counseling	$64.63	50.5%	$32.63	11.1 (8.8)	10.7 (0.0)	11.3 (0.0)	0.9 (0.0)	0.0 (0.0)	6.8 (1.8)
Other NCDs				4.7 (0.6)	0.0 (0.0)	0.0 (0.0)	0.0 (0.0)	0.0 (0.0)	0.9 (0.1)
HC47 Palliative care	$64.63	14.8%	$9.56	4.7 (0.6)	0.0 (0.0)	0.0 (0.0)	0.0 (0.0)	0.0 (0.0)	0.9 (0.1)
Reproductive health				9.1 (2.8)	6.5 (0.0)	0.0 (0.0)	0.0 (0.0)	0.0 (0.0)	3.1 (0.6)
Family planning				0.0 (0.0)	0.0 (0.0)	0.0 (0.0)	0.0 (0.0)	0.0 (0.0)	0.0 (0.0)
HC4 Contraceptives	$4.97	0.3%	$0.01	0.0 (0.0)	0.0 (0.0)	0.0 (0.0)	0.0 (0.0)	0.0 (0.0)	0.0 (0.0)
Maternal conditions				14.8 (4.7)	10.9 (0.0)	0.0 (0.0)	0.0 (0.0)	0.0 (0.0)	5.1 (0.9)
C5 Antenatal tetanus immunization	$0.39	29.9%	$0.12	0.0 (0.0)	0.0 (0.0)	0.0 (0.0)	0.0 (0.0)	0.0 (0.0)	0.0 (0.0)
HC11 Basic emergency newborn & obstetric care	$69.14	29.9%	$20.66	87.6 (27.9)	65.3 (0.0)	0.0 (0.0)	0.0 (0.0)	0.0 (0.0)	30.6 (5.6)
HC2 Post-abortion care	$4.54	29.9%	$1.36	0.4 (0.0)	0.0 (0.0)	0.0 (0.0)	0.0 (0.0)	0.0 (0.0)	0.1 (0.0)
HC3 Treatment of premature membrane rupture	$4.04	29.9%	$1.21	0.3 (0.0)	0.0 (0.0)	0.0 (0.0)	0.0 (0.0)	0.0 (0.0)	0.1 (0.0)
HC5 Kangaroo mother care counseling	$2.33	29.9%	$0.70	0.0 (0.0)	0.0 (0.0)	0.0 (0.0)	0.0 (0.0)	0.0 (0.0)	0.0 (0.0)
HC7 Medical abortion	$4.81	29.9%	$1.44	0.5 (0.0)	0.0 (0.0)	0.0 (0.0)	0.0 (0.0)	0.0 (0.0)	0.1 (0.0)
Perinatal conditions				0.6 (0.1)	0.0 (0.0)	0.0 (0.0)	0.0 (0.0)	0.0 (0.0)	0.1 (0.0)
C13 Cotrimoxazole for HIV-exposed children	$6.55	38.6%	$2.53	1.0 (0.1)	0.0 (0.0)	0.0 (0.0)	0.0 (0.0)	0.0 (0.0)	0.2 (0.0)
HC1 Antibiotics for neonatal pneumonia	$6.22	38.6%	$2.40	0.8 (0.1)	0.0 (0.0)	0.0 (0.0)	0.0 (0.0)	0.0 (0.0)	0.2 (0.0)
HC6 Neonatal sepsis, pneumonia, meningitis	$2.66	38.6%	$1.03	0.1 (0.0)	0.0 (0.0)	0.0 (0.0)	0.0 (0.0)	0.0 (0.0)	0.0 (0.0)

Table B.20. Full model results: Mali.

**Mali**				**CHE risk** 10% threshold (25% threshold)					
Disease category, disease, intervention	Cost ($)	OOP (%)	OOP ($)	Q1	Q2	Q3	Q4	Q5	Total
Childhood health				0.0 (0.0)	0.0 (0.0)	0.0 (0.0)	0.0 (0.0)	0.0 (0.0)	0.0 (0.0)
Childhood health				0.0 (0.0)	0.0 (0.0)	0.0 (0.0)	0.0 (0.0)	0.0 (0.0)	0.0 (0.0)
HC42 Acute pharyngitis treatment	$0.17	42.3%	$0.07	0.0 (0.0)	0.0 (0.0)	0.0 (0.0)	0.0 (0.0)	0.0 (0.0)	0.0 (0.0)
Infectious & parasitic diseases				8.2 (2.7)	6.8 (0.0)	0.4 (0.0)	0.0 (0.0)	0.0 (0.0)	3.1 (0.5)
Diarrheal diseases				0.1 (0.0)	0.0 (0.0)	0.0 (0.0)	0.0 (0.0)	0.0 (0.0)	0.0 (0.0)
HC12 Diagnosis & treatment of infections (IMCI)	$4.79	49.2%	$2.36	0.1 (0.0)	0.0 (0.0)	0.0 (0.0)	0.0 (0.0)	0.0 (0.0)	0.0 (0.0)
HIV/AIDS & other sexually transmitted diseases				4.1 (0.8)	1.1 (0.0)	0.0 (0.0)	0.0 (0.0)	0.0 (0.0)	1.0 (0.2)
HC13 ART & viral load monitoring	$71.45	22.9%	$16.35	1.2 (0.1)	0.0 (0.0)	0.0 (0.0)	0.0 (0.0)	0.0 (0.0)	0.2 (0.0)
HC17 Syndromic management of STI	$5.67	22.9%	$1.30	0.0 (0.0)	0.0 (0.0)	0.0 (0.0)	0.0 (0.0)	0.0 (0.0)	0.0 (0.0)
HC23 HIV, STIs, hepatitis testing & counseling	$4.31	22.9%	$0.99	0.0 (0.0)	0.0 (0.0)	0.0 (0.0)	0.0 (0.0)	0.0 (0.0)	0.0 (0.0)
HC8 HIV & syphilis PMTCT	$176.35	22.9%	$40.34	15.3 (2.9)	4.3 (0.0)	0.0 (0.0)	0.0 (0.0)	0.0 (0.0)	3.9 (0.6)
Malaria				0.0 (0.0)	0.0 (0.0)	0.0 (0.0)	0.0 (0.0)	0.0 (0.0)	0.0 (0.0)
C7 Intermittent preventive treatment (pregnancy)	$0.45	59.5%	$0.27	0.0 (0.0)	0.0 (0.0)	0.0 (0.0)	0.0 (0.0)	0.0 (0.0)	0.0 (0.0)
Tuberculosis				49.3 (18.3)	50.5 (0.0)	2.8 (0.0)	0.0 (0.0)	0.0 (0.0)	20.5 (3.7)

Mali				CHE risk 10% threshold (25% threshold)					
Disease category, disease, intervention	Cost ($)	OOP (%)	OOP ($)	Q1	Q2	Q3	Q4	Q5	Total
HC27 Diagnosis & treatment of TB	$135.09	40.3%	$54.44	49.3 (18.3)	50.5 (0.0)	2.8 (0.0)	0.0 (0.0)	0.0 (0.0)	20.5 (3.7)
Other infectious & parasitic diseases				0.0 (0.0)	0.0 (0.0)	0.0 (0.0)	0.0 (0.0)	0.0 (0.0)	0.0 (0.0)
HC30 Management & referrals for fever (IMAI)	$3.11	51.5%	$1.60	0.0 (0.0)	0.0 (0.0)	0.0 (0.0)	0.0 (0.0)	0.0 (0.0)	0.0 (0.0)
Noncommunicable diseases (NCDs)				19.1 (12.3)	17.0 (6.2)	12.6 (2.6)	11.1 (0.2)	8.8 (0.0)	13.7 (4.3)
Cardiovascular diseases				7.8 (6.0)	8.2 (4.1)	6.6 (4.3)	6.3 (0.6)	7.2 (0.0)	7.2 (3.0)
HC38 Aspirin for acute myocardial infarction	$0.03	76.3%	$0.02	0.0 (0.0)	0.0 (0.0)	0.0 (0.0)	0.0 (0.0)	0.0 (0.0)	0.0 (0.0)
HC43 Management of ischemic heart disease	$83.97	76.3%	$64.09	11.7 (6.2)	12.2 (0.0)	6.9 (0.0)	0.0 (0.0)	0.0 (0.0)	6.2 (1.2)
HC44 Management of heart failure	$249.96	76.3%	$190.77	11.7 (11.7)	12.3 (12.3)	13.0 (13.0)	18.8 (1.9)	21.6 (0.0)	15.5 (7.8)
Endocrine & metabolic disorders				8.0 (1.6)	2.4 (0.0)	0.0 (0.0)	0.0 (0.0)	0.0 (0.0)	2.1 (0.3)
HC40 Screening & management of diabetes	$64.16	63.7%	$40.85	8.0 (1.6)	2.4 (0.0)	0.0 (0.0)	0.0 (0.0)	0.0 (0.0)	2.1 (0.3)
Mental/behavioral disorders & neurological conditions				31.8 (20.7)	28.5 (10.0)	21.2 (2.6)	18.4 (0.0)	13.2 (0.0)	22.6 (6.7)
HC49 Management of bipolar disorder	$184.57	79.1%	$146.07	48.2 (48.2)	49.9 (49.9)	52.9 (13.1)	77.2 (0.0)	66.1 (0.0)	58.8 (22.2)
HC50 Management of depression	$16.11	79.1%	$12.75	5.3 (0.5)	0.0 (0.0)	0.0 (0.0)	0.0 (0.0)	0.0 (0.0)	1.1 (0.1)
HC51 Management of epilepsy	$27.53	79.1%	$21.79	9.6 (1.1)	0.0 (0.0)	0.0 (0.0)	0.0 (0.0)	0.0 (0.0)	1.9 (0.2)
HC52 Management of schizophrenia	$99.43	79.1%	$78.69	48.0 (38.3)	49.9 (0.0)	53.1 (0.0)	15.0 (0.0)	0.0 (0.0)	33.2 (7.7)
HC66 Psychosocial support & counseling	$64.63	79.1%	$51.15	48.1 (15.6)	42.9 (0.0)	0.0 (0.0)	0.0 (0.0)	0.0 (0.0)	18.2 (3.1)
Other NCDs				0.0 (0.0)	0.0 (0.0)	0.0 (0.0)	0.0 (0.0)	0.0 (0.0)	0.0 (0.0)
HC47 Palliative care	$64.63	16.5%	$10.63	0.0 (0.0)	0.0 (0.0)	0.0 (0.0)	0.0 (0.0)	0.0 (0.0)	0.0 (0.0)
Reproductive health				2.6 (0.3)	0.0 (0.0)	0.0 (0.0)	0.0 (0.0)	0.0 (0.0)	0.5 (0.1)
Family planning				0.0 (0.0)	0.0 (0.0)	0.0 (0.0)	0.0 (0.0)	0.0 (0.0)	0.0 (0.0)
HC4 Contraceptives	$4.97	22.9%	$1.14	0.0 (0.0)	0.0 (0.0)	0.0 (0.0)	0.0 (0.0)	0.0 (0.0)	0.0 (0.0)
Maternal conditions				4.3 (0.5)	0.0 (0.0)	0.0 (0.0)	0.0 (0.0)	0.0 (0.0)	0.9 (0.1)
C5 Antenatal tetanus immunization	$0.39	37.5%	$0.14	0.0 (0.0)	0.0 (0.0)	0.0 (0.0)	0.0 (0.0)	0.0 (0.0)	0.0 (0.0)
HC11 Basic emergency newborn & obstetric care	$69.14	37.5%	$25.91	25.6 (3.1)	0.0 (0.0)	0.0 (0.0)	0.0 (0.0)	0.0 (0.0)	5.1 (0.6)
HC2 Post-abortion care	$4.54	37.5%	$1.70	0.0 (0.0)	0.0 (0.0)	0.0 (0.0)	0.0 (0.0)	0.0 (0.0)	0.0 (0.0)
HC3 Treatment of premature membrane rupture	$4.04	37.5%	$1.51	0.0 (0.0)	0.0 (0.0)	0.0 (0.0)	0.0 (0.0)	0.0 (0.0)	0.0 (0.0)
HC5 Kangaroo mother care counseling	$2.33	37.5%	$0.87	0.0 (0.0)	0.0 (0.0)	0.0 (0.0)	0.0 (0.0)	0.0 (0.0)	0.0 (0.0)
HC7 Medical abortion	$4.81	37.5%	$1.80	0.0 (0.0)	0.0 (0.0)	0.0 (0.0)	0.0 (0.0)	0.0 (0.0)	0.0 (0.0)
Perinatal conditions				0.1 (0.0)	0.0 (0.0)	0.0 (0.0)	0.0 (0.0)	0.0 (0.0)	0.0 (0.0)
C13 Cotrimoxazole for HIV-exposed children	$6.55	53.1%	$3.48	0.1 (0.0)	0.0 (0.0)	0.0 (0.0)	0.0 (0.0)	0.0 (0.0)	0.0 (0.0)
HC1 Antibiotics for neonatal pneumonia	$6.22	53.1%	$3.30	0.1 (0.0)	0.0 (0.0)	0.0 (0.0)	0.0 (0.0)	0.0 (0.0)	0.0 (0.0)
HC6 Neonatal sepsis, pneumonia, meningitis	$2.66	53.1%	$1.41	0.0 (0.0)	0.0 (0.0)	0.0 (0.0)	0.0 (0.0)	0.0 (0.0)	0.0 (0.0)

Table B.21. Full model results: Mozambique.

Mozambique				CHE risk 10% threshold (25% threshold)					
Disease category, disease, intervention	Cost ($)	OOP (%)	OOP ($)	Q1	Q2	Q3	Q4	Q5	Total
Childhood health				0.0 (0.0)	0.0 (0.0)	0.0 (0.0)	0.0 (0.0)	0.0 (0.0)	0.0 (0.0)
Childhood health				0.0 (0.0)	0.0 (0.0)	0.0 (0.0)	0.0 (0.0)	0.0 (0.0)	0.0 (0.0)

**Mozambique**				CHE risk<br>10% threshold (25% threshold)					
Disease category, disease, intervention	Cost ($)	OOP (%)	OOP ($)	Q1	Q2	Q3	Q4	Q5	Total
HC42 Acute pharyngitis treatment	$0.17	39.3%	$0.07	0.0 (0.0)	0.0 (0.0)	0.0 (0.0)	0.0 (0.0)	0.0 (0.0)	0.0 (0.0)
Infectious & parasitic diseases				11.2 (2.5)	0.0 (0.0)	0.0 (0.0)	0.0 (0.0)	0.0 (0.0)	2.2 (0.5)
Diarrheal diseases				0.8 (0.2)	0.0 (0.0)	0.0 (0.0)	0.0 (0.0)	0.0 (0.0)	0.2 (0.0)
HC12 Diagnosis & treatment of infections (IMCI)	$4.79	42.4%	$2.03	0.8 (0.2)	0.0 (0.0)	0.0 (0.0)	0.0 (0.0)	0.0 (0.0)	0.2 (0.0)
HIV/AIDS & other sexually transmitted diseases				11.3 (2.5)	0.0 (0.0)	0.0 (0.0)	0.0 (0.0)	0.0 (0.0)	2.3 (0.5)
HC13 ART & viral load monitoring	$71.45	8.5%	$6.09	4.5 (0.9)	0.0 (0.0)	0.0 (0.0)	0.0 (0.0)	0.0 (0.0)	0.9 (0.2)
HC17 Syndromic management of STI	$5.67	8.5%	$0.48	0.0 (0.0)	0.0 (0.0)	0.0 (0.0)	0.0 (0.0)	0.0 (0.0)	0.0 (0.0)
HC23 HIV, STIs, hepatitis testing & counseling	$4.31	8.5%	$0.37	0.0 (0.0)	0.0 (0.0)	0.0 (0.0)	0.0 (0.0)	0.0 (0.0)	0.0 (0.0)
HC8 HIV & syphilis PMTCT	$176.35	8.5%	$15.04	40.8 (9.1)	0.0 (0.0)	0.0 (0.0)	0.0 (0.0)	0.0 (0.0)	8.2 (1.8)
Malaria				0.0 (0.0)	0.0 (0.0)	0.0 (0.0)	0.0 (0.0)	0.0 (0.0)	0.0 (0.0)
C7 Intermittent preventive treatment (pregnancy)	$0.45	3.5%	$0.02	0.0 (0.0)	0.0 (0.0)	0.0 (0.0)	0.0 (0.0)	0.0 (0.0)	0.0 (0.0)
Tuberculosis				43.6 (10.0)	0.0 (0.0)	0.0 (0.0)	0.0 (0.0)	0.0 (0.0)	8.7 (2.0)
HC27 Diagnosis & treatment of TB	$135.09	12.7%	$17.10	43.6 (10.0)	0.0 (0.0)	0.0 (0.0)	0.0 (0.0)	0.0 (0.0)	8.7 (2.0)
Other infectious & parasitic diseases				0.0 (0.0)	0.0 (0.0)	0.0 (0.0)	0.0 (0.0)	0.0 (0.0)	0.0 (0.0)
HC30 Management & referrals for fever (IMAI)	$3.11	1.3%	$0.04	0.0 (0.0)	0.0 (0.0)	0.0 (0.0)	0.0 (0.0)	0.0 (0.0)	0.0 (0.0)
Noncommunicable diseases (NCDs)				10.4 (8.9)	9.4 (6.3)	10.8 (4.3)	10.6 (4.2)	6.4 (1.5)	9.5 (5.1)
Cardiovascular diseases				16.8 (16.8)	18.5 (15.9)	21.7 (10.9)	26.6 (13.6)	16.9 (5.2)	20.1 (12.5)
HC38 Aspirin for acute myocardial infarction	$0.03	100.0%	$0.03	0.0 (0.0)	0.0 (0.0)	0.0 (0.0)	0.0 (0.0)	0.0 (0.0)	0.0 (0.0)
HC43 Management of ischemic heart disease	$83.97	100.0%	$83.97	25.1 (25.1)	27.5 (20.0)	32.6 (0.0)	38.7 (0.0)	0.5 (0.0)	24.9 (9.0)
HC44 Management of heart failure	$249.96	100.0%	$249.96	25.2 (25.2)	27.9 (27.9)	32.6 (32.6)	40.9 (40.9)	50.3 (15.5)	35.4 (28.4)
Endocrine & metabolic disorders				2.7 (2.7)	3.0 (0.6)	3.5 (0.0)	1.4 (0.0)	0.0 (0.0)	2.1 (0.7)
HC40 Screening & management of diabetes	$64.16	100.0%	$64.16	2.7 (2.7)	3.0 (0.6)	3.5 (0.0)	1.4 (0.0)	0.0 (0.0)	2.1 (0.7)
Mental/behavioral disorders & neurological conditions				8.7 (5.6)	5.4 (2.9)	5.7 (2.1)	4.8 (0.2)	2.6 (0.0)	5.5 (2.2)
HC49 Management of bipolar disorder	$184.57	79.6%	$147.00	8.0 (8.0)	9.0 (9.0)	10.4 (10.4)	13.2 (1.2)	13.2 (0.0)	10.8 (5.7)
HC50 Management of depression	$16.11	79.6%	$12.83	3.6 (0.8)	0.0 (0.0)	0.0 (0.0)	0.0 (0.0)	0.0 (0.0)	0.7 (0.2)
HC51 Management of epilepsy	$27.53	79.6%	$21.93	15.6 (3.9)	0.3 (0.0)	0.0 (0.0)	0.0 (0.0)	0.0 (0.0)	3.2 (0.8)
HC52 Management of schizophrenia	$99.43	79.6%	$79.19	8.1 (8.1)	8.8 (5.4)	10.4 (0.0)	10.8 (0.0)	0.0 (0.0)	7.6 (2.7)
HC66 Psychosocial support & counseling	$64.63	79.6%	$51.48	8.1 (7.3)	8.9 (0.0)	7.8 (0.0)	0.0 (0.0)	0.0 (0.0)	5.0 (1.5)
Other NCDs				7.9 (7.9)	8.7 (0.6)	10.2 (0.0)	0.7 (0.0)	0.0 (0.0)	5.5 (1.7)
HC47 Palliative care	$64.63	89.6%	$57.90	7.9 (7.9)	8.7 (0.6)	10.2 (0.0)	0.7 (0.0)	0.0 (0.0)	5.5 (1.7)
Reproductive health				0.3 (0.1)	0.0 (0.0)	0.0 (0.0)	0.0 (0.0)	0.0 (0.0)	0.1 (0.0)
Family planning				0.0 (0.0)	0.0 (0.0)	0.0 (0.0)	0.0 (0.0)	0.0 (0.0)	0.0 (0.0)
HC4 Contraceptives	$4.97	0.6%	$0.03	0.0 (0.0)	0.0 (0.0)	0.0 (0.0)	0.0 (0.0)	0.0 (0.0)	0.0 (0.0)
Maternal conditions				0.0 (0.0)	0.0 (0.0)	0.0 (0.0)	0.0 (0.0)	0.0 (0.0)	0.0 (0.0)
C5 Antenatal tetanus immunization	$0.39	0.1%	$0.00	0.0 (0.0)	0.0 (0.0)	0.0 (0.0)	0.0 (0.0)	0.0 (0.0)	0.0 (0.0)
HC11Basic emergency newborn & obstetric care	$69.14	0.1%	$0.09	0.0 (0.0)	0.0 (0.0)	0.0 (0.0)	0.0 (0.0)	0.0 (0.0)	0.0 (0.0)
HC2 Post-abortion care	$4.54	0.1%	$0.01	0.0 (0.0)	0.0 (0.0)	0.0 (0.0)	0.0 (0.0)	0.0 (0.0)	0.0 (0.0)
HC3 Treatment of premature membrane rupture	$4.04	0.1%	$0.01	0.0 (0.0)	0.0 (0.0)	0.0 (0.0)	0.0 (0.0)	0.0 (0.0)	0.0 (0.0)
HC5 Kangaroo mother care counseling	$2.33	0.1%	$0.00	0.0 (0.0)	0.0 (0.0)	0.0 (0.0)	0.0 (0.0)	0.0 (0.0)	0.0 (0.0)
HC7 Medical abortion	$4.81	0.1%	$0.01	0.0 (0.0)	0.0 (0.0)	0.0 (0.0)	0.0 (0.0)	0.0 (0.0)	0.0 (0.0)

Mozambique				CHE risk 10% threshold (25% threshold)					
Disease category, disease, intervention	Cost ($)	OOP (%)	OOP ($)	Q1	Q2	Q3	Q4	Q5	Total
Perinatal conditions				0.9 (0.2)	0.0 (0.0)	0.0 (0.0)	0.0 (0.0)	0.0 (0.0)	0.2 (0.0)
C13 Cotrimoxazole for HIV-exposed children	$6.55	38.6%	$2.53	1.3 (0.3)	0.0 (0.0)	0.0 (0.0)	0.0 (0.0)	0.0 (0.0)	0.3 (0.1)
HC1 Antibiotics for neonatal pneumonia	$6.22	38.6%	$2.40	1.2 (0.2)	0.0 (0.0)	0.0 (0.0)	0.0 (0.0)	0.0 (0.0)	0.2 (0.0)
HC6 Neonatal sepsis, pneumonia, meningitis	$2.66	38.6%	$1.03	0.3 (0.0)	0.0 (0.0)	0.0 (0.0)	0.0 (0.0)	0.0 (0.0)	0.1 (0.0)

Table B.22. Full model results: Myanmar.

Myanmar				CHE risk 10% threshold (25% threshold)					
Disease category, disease, intervention	Cost ($)	OOP (%)	OOP ($)	Q1	Q2	Q3	Q4	Q5	Total
Childhood health				0.0 (0.0)	0.0 (0.0)	0.0 (0.0)	0.0 (0.0)	0.0 (0.0)	0.0 (0.0)
Childhood health				0.0 (0.0)	0.0 (0.0)	0.0 (0.0)	0.0 (0.0)	0.0 (0.0)	0.0 (0.0)
HC42 Acute pharyngitis treatment	$0.24	45.3%	$0.11	0.0 (0.0)	0.0 (0.0)	0.0 (0.0)	0.0 (0.0)	0.0 (0.0)	0.0 (0.0)
Infectious & parasitic diseases				6.9 (0.7)	0.0 (0.0)	0.0 (0.0)	0.0 (0.0)	0.0 (0.0)	1.4 (0.1)
Diarrheal diseases				0.4 (0.0)	0.0 (0.0)	0.0 (0.0)	0.0 (0.0)	0.0 (0.0)	0.1 (0.0)
HC12 Diagnosis & treatment of infections (IMCI)	$10.29	85.0%	$8.75	0.4 (0.0)	0.0 (0.0)	0.0 (0.0)	0.0 (0.0)	0.0 (0.0)	0.1 (0.0)
HIV/AIDS & other sexually transmitted diseases				0.4 (0.0)	0.0 (0.0)	0.0 (0.0)	0.0 (0.0)	0.0 (0.0)	0.1 (0.0)
HC13 ART & viral load monitoring	$121.57	4.2%	$5.10	0.0 (0.0)	0.0 (0.0)	0.0 (0.0)	0.0 (0.0)	0.0 (0.0)	0.0 (0.0)
HC17 Syndromic management of STI	$10.69	4.2%	$0.45	0.0 (0.0)	0.0 (0.0)	0.0 (0.0)	0.0 (0.0)	0.0 (0.0)	0.0 (0.0)
HC23 HIV, STIs, hepatitis testing & counseling	$6.08	4.2%	$0.26	0.0 (0.0)	0.0 (0.0)	0.0 (0.0)	0.0 (0.0)	0.0 (0.0)	0.0 (0.0)
HC8 HIV & syphilis PMTCT	$313.51	4.2%	$13.16	1.5 (0.1)	0.0 (0.0)	0.0 (0.0)	0.0 (0.0)	0.0 (0.0)	0.3 (0.0)
Malaria				0.0 (0.0)	0.0 (0.0)	0.0 (0.0)	0.0 (0.0)	0.0 (0.0)	0.0 (0.0)
C7 Intermittent preventive treatment (pregnancy)	$1.02	6.7%	$0.07	0.0 (0.0)	0.0 (0.0)	0.0 (0.0)	0.0 (0.0)	0.0 (0.0)	0.0 (0.0)
Tuberculosis				53.2 (5.4)	0.0 (0.0)	0.0 (0.0)	0.0 (0.0)	0.0 (0.0)	10.6 (1.1)
HC27 Diagnosis & treatment of TB	$175.65	30.1%	$52.85	53.2 (5.4)	0.0 (0.0)	0.0 (0.0)	0.0 (0.0)	0.0 (0.0)	10.6 (1.1)
Other infectious & parasitic diseases				0.0 (0.0)	0.0 (0.0)	0.0 (0.0)	0.0 (0.0)	0.0 (0.0)	0.0 (0.0)
HC30 Management & referrals for fever (IMAI)	$6.83	61.9%	$4.23	0.0 (0.0)	0.0 (0.0)	0.0 (0.0)	0.0 (0.0)	0.0 (0.0)	0.0 (0.0)
Noncommunicable diseases (NCDs)				16.0 (14.3)	16.4 (13.9)	15.2 (8.1)	22.0 (1.6)	18.1 (0.0)	17.6 (7.6)
Cardiovascular diseases				20.1 (20.1)	22.7 (16.2)	21.7 (10.8)	31.5 (5.4)	21.6 (0.0)	23.5 (10.5)
HC38 Aspirin for acute myocardial infarction	$0.05	90.4%	$0.05	0.0 (0.0)	0.0 (0.0)	0.0 (0.0)	0.0 (0.0)	0.0 (0.0)	0.0 (0.0)
HC43 Management of ischemic heart disease	$190.17	90.4%	$171.91	30.1 (30.1)	34.0 (14.5)	32.4 (0.0)	47.2 (0.0)	15.6 (0.0)	31.9 (8.9)
HC44 Management of heart failure	$342.48	90.4%	$309.60	30.1 (30.1)	34.1 (34.1)	32.5 (32.5)	47.2 (16.1)	49.2 (0.0)	38.6 (22.6)
Endocrine & metabolic disorders				4.8 (1.5)	5.4 (0.0)	0.3 (0.0)	0.0 (0.0)	0.0 (0.0)	2.1 (0.3)
HC40 Screening & management of diabetes	$92.52	91.7%	$84.82	4.8 (1.5)	5.4 (0.0)	0.3 (0.0)	0.0 (0.0)	0.0 (0.0)	2.1 (0.3)
Mental/behavioral disorders & neurological conditions				19.0 (16.3)	18.2 (18.2)	17.4 (9.6)	25.2 (0.0)	23.2 (0.0)	20.6 (8.8)
HC49 Management of bipolar disorder	$365.39	71.2%	$259.99	40.1 (40.1)	45.5 (45.5)	43.6 (32.0)	63.0 (0.0)	60.9 (0.0)	50.6 (23.5)
HC50 Management of depression	$48.02	71.2%	$34.17	12.3 (1.0)	0.0 (0.0)	0.0 (0.0)	0.0 (0.0)	0.0 (0.0)	2.5 (0.2)
HC51 Management of epilepsy	$53.73	71.2%	$38.23	1.0 (0.1)	0.0 (0.0)	0.0 (0.0)	0.0 (0.0)	0.0 (0.0)	0.2 (0.0)
HC52 Management of schizophrenia	$329.34	71.2%	$234.34	40.2 (40.2)	45.4 (45.4)	43.6 (16.2)	62.9 (0.0)	55.3 (0.0)	49.5 (20.4)
HC66 Psychosocial support & counseling	$21.32	71.2%	$15.17	1.3 (0.1)	0.0 (0.0)	0.0 (0.0)	0.0 (0.0)	0.0 (0.0)	0.3 (0.0)

Myanmar				CHE risk 10% threshold (25% threshold)					
Disease category, disease, intervention	Cost ($)	OOP (%)	OOP ($)	Q1	Q2	Q3	Q4	Q5	Total
Other NCDs				0.2 (0.0)	0.0 (0.0)	0.0 (0.0)	0.0 (0.0)	0.0 (0.0)	0.0 (0.0)
HC47 Palliative care	$21.32	64.8%	$13.81	0.2 (0.0)	0.0 (0.0)	0.0 (0.0)	0.0 (0.0)	0.0 (0.0)	0.0 (0.0)
Reproductive health				4.0 (3.3)	5.6 (0.0)	7.0 (0.0)	4.5 (0.0)	0.0 (0.0)	4.2 (0.7)
Family planning				0.1 (0.0)	0.0 (0.0)	0.0 (0.0)	0.0 (0.0)	0.0 (0.0)	0.0 (0.0)
HC4 Contraceptives	$10.32	59.9%	$6.18	0.1 (0.0)	0.0 (0.0)	0.0 (0.0)	0.0 (0.0)	0.0 (0.0)	0.0 (0.0)
Maternal conditions				6.7 (5.5)	9.3 (0.0)	11.6 (0.0)	7.4 (0.0)	0.0 (0.0)	7.0 (1.1)
C5 Antenatal tetanus immunization	$0.44	90.7%	$0.40	0.0 (0.0)	0.0 (0.0)	0.0 (0.0)	0.0 (0.0)	0.0 (0.0)	0.0 (0.0)
HC11 Basic emergency newborn & obstetric care	$145.10	90.7%	$131.55	39.8 (33.3)	55.9 (0.0)	69.9 (0.0)	44.6 (0.0)	0.0 (0.0)	42.0 (6.7)
HC2 Post-abortion care	$8.23	90.7%	$7.46	0.2 (0.0)	0.0 (0.0)	0.0 (0.0)	0.0 (0.0)	0.0 (0.0)	0.0 (0.0)
HC3 Treatment of premature membrane rupture	$4.66	90.7%	$4.23	0.0 (0.0)	0.0 (0.0)	0.0 (0.0)	0.0 (0.0)	0.0 (0.0)	0.0 (0.0)
HC5 Kangaroo mother care counseling	$4.61	90.7%	$4.18	0.0 (0.0)	0.0 (0.0)	0.0 (0.0)	0.0 (0.0)	0.0 (0.0)	0.0 (0.0)
HC7 Medical abortion	$5.52	90.7%	$5.00	0.1 (0.0)	0.0 (0.0)	0.0 (0.0)	0.0 (0.0)	0.0 (0.0)	0.0 (0.0)
Perinatal conditions				0.1 (0.0)	0.0 (0.0)	0.0 (0.0)	0.0 (0.0)	0.0 (0.0)	0.0 (0.0)
C13 Cotrimoxazole for HIV-exposed children	$11.95	70.8%	$8.46	0.3 (0.0)	0.0 (0.0)	0.0 (0.0)	0.0 (0.0)	0.0 (0.0)	0.1 (0.0)
HC1 Antibiotics for neonatal pneumonia	$6.61	70.8%	$4.68	0.0 (0.0)	0.0 (0.0)	0.0 (0.0)	0.0 (0.0)	0.0 (0.0)	0.0 (0.0)
HC6 Neonatal sepsis, pneumonia, meningitis	$3.51	70.8%	$2.48	0.0 (0.0)	0.0 (0.0)	0.0 (0.0)	0.0 (0.0)	0.0 (0.0)	0.0 (0.0)

Table B.23. Full model results: Nepal.

Nepal				CHE risk 10% threshold (25% threshold)					
Disease category, disease, intervention	Cost ($)	OOP (%)	OOP ($)	Q1	Q2	Q3	Q4	Q5	Total
Childhood health				0.0 (0.0)	0.0 (0.0)	0.0 (0.0)	0.0 (0.0)	0.0 (0.0)	0.0 (0.0)
Childhood health				0.0 (0.0)	0.0 (0.0)	0.0 (0.0)	0.0 (0.0)	0.0 (0.0)	0.0 (0.0)
HC42 Acute pharyngitis treatment	$0.17	39.3%	$0.07	0.0 (0.0)	0.0 (0.0)	0.0 (0.0)	0.0 (0.0)	0.0 (0.0)	0.0 (0.0)
Infectious & parasitic diseases				2.5 (0.2)	0.0 (0.0)	0.0 (0.0)	0.0 (0.0)	0.0 (0.0)	0.5 (0.0)
Diarrheal diseases				0.2 (0.0)	0.0 (0.0)	0.0 (0.0)	0.0 (0.0)	0.0 (0.0)	0.0 (0.0)
HC12 Diagnosis & treatment of infections (IMCI)	$4.79	85.8%	$4.11	0.2 (0.0)	0.0 (0.0)	0.0 (0.0)	0.0 (0.0)	0.0 (0.0)	0.0 (0.0)
HIV/AIDS & other sexually transmitted diseases				3.7 (0.4)	0.0 (0.0)	0.0 (0.0)	0.0 (0.0)	0.0 (0.0)	0.7 (0.1)
HC13 ART & viral load monitoring	$71.45	13.9%	$9.90	0.4 (0.0)	0.0 (0.0)	0.0 (0.0)	0.0 (0.0)	0.0 (0.0)	0.1 (0.0)
HC17 Syndromic management of STI	$5.67	13.9%	$0.79	0.0 (0.0)	0.0 (0.0)	0.0 (0.0)	0.0 (0.0)	0.0 (0.0)	0.0 (0.0)
HC23 HIV, STIs, hepatitis testing & counseling	$4.31	13.9%	$0.60	0.0 (0.0)	0.0 (0.0)	0.0 (0.0)	0.0 (0.0)	0.0 (0.0)	0.0 (0.0)
HC8 HIV & syphilis PMTCT	$176.35	13.9%	$24.43	14.3 (1.4)	0.0 (0.0)	0.0 (0.0)	0.0 (0.0)	0.0 (0.0)	2.9 (0.3)
Malaria				0.0 (0.0)	0.0 (0.0)	0.0 (0.0)	0.0 (0.0)	0.0 (0.0)	0.0 (0.0)
C7 Intermittent preventive treatment (pregnancy)	$0.45	33.2%	$0.15	0.0 (0.0)	0.0 (0.0)	0.0 (0.0)	0.0 (0.0)	0.0 (0.0)	0.0 (0.0)
Tuberculosis				5.4 (0.5)	0.0 (0.0)	0.0 (0.0)	0.0 (0.0)	0.0 (0.0)	1.1 (0.1)
HC27 Diagnosis & treatment of TB	$135.09	12.6%	$17.05	5.4 (0.5)	0.0 (0.0)	0.0 (0.0)	0.0 (0.0)	0.0 (0.0)	1.1 (0.1)
Other infectious & parasitic diseases				0.0 (0.0)	0.0 (0.0)	0.0 (0.0)	0.0 (0.0)	0.0 (0.0)	0.0 (0.0)
HC30 Management & referrals for fever (IMAI)	$3.11	54.8%	$1.71	0.0 (0.0)	0.0 (0.0)	0.0 (0.0)	0.0 (0.0)	0.0 (0.0)	0.0 (0.0)
Noncommunicable diseases (NCDs)				6.8 (3.0)	4.8 (1.4)	2.7 (1.0)	2.3 (0.0)	1.5 (0.0)	3.6 (1.1)

Nepal				CHE risk 10% threshold (25% threshold)					
Disease category, disease, intervention	Cost ($)	OOP (%)	OOP ($)	Q1	Q2	Q3	Q4	Q5	Total
Cardiovascular diseases				6.1 (4.3)	6.9 (3.5)	4.7 (3.2)	4.1 (0.0)	3.9 (0.0)	5.2 (2.2)
HC38 Aspirin for acute myocardial infarction	$0.03	78.4%	$0.02	0.0 (0.0)	0.0 (0.0)	0.0 (0.0)	0.0 (0.0)	0.0 (0.0)	0.0 (0.0)
HC43 Management of ischemic heart disease	$83.97	78.4%	$65.87	9.2 (3.7)	10.4 (0.0)	3.4 (0.0)	0.0 (0.0)	0.0 (0.0)	4.6 (0.7)
HC44 Management of heart failure	$249.96	78.4%	$196.09	9.2 (9.2)	10.4 (10.4)	10.7 (9.6)	12.3 (0.0)	11.8 (0.0)	10.9 (5.8)
Endocrine & metabolic disorders				17.6 (3.0)	5.9 (0.0)	0.0 (0.0)	0.0 (0.0)	0.0 (0.0)	4.7 (0.6)
HC40 Screening & management of diabetes	$64.16	71.9%	$46.12	17.6 (3.0)	5.9 (0.0)	0.0 (0.0)	0.0 (0.0)	0.0 (0.0)	4.7 (0.6)
Mental/behavioral disorders & neurological conditions				6.0 (2.6)	4.0 (0.8)	2.5 (0.0)	2.2 (0.0)	0.6 (0.0)	3.1 (0.7)
HC49 Management of bipolar disorder	$184.57	66.0%	$121.89	8.3 (8.3)	9.3 (4.1)	9.7 (0.0)	11.2 (0.0)	2.8 (0.0)	8.3 (2.5)
HC50 Management of depression	$16.11	66.0%	$10.64	0.3 (0.0)	0.0 (0.0)	0.0 (0.0)	0.0 (0.0)	0.0 (0.0)	0.1 (0.0)
HC51 Management of epilepsy	$27.53	66.0%	$18.18	4.8 (0.4)	0.0 (0.0)	0.0 (0.0)	0.0 (0.0)	0.0 (0.0)	1.0 (0.1)
HC52 Management of schizophrenia	$99.43	66.0%	$65.67	8.3 (3.3)	9.4 (0.0)	3.0 (0.0)	0.0 (0.0)	0.0 (0.0)	4.1 (0.7)
HC66 Psychosocial support & counseling	$64.63	66.0%	$42.68	8.3 (1.2)	1.2 (0.0)	0.0 (0.0)	0.0 (0.0)	0.0 (0.0)	1.9 (0.2)
Other NCDs				1.7 (0.5)	1.4 (0.0)	0.0 (0.0)	0.0 (0.0)	0.0 (0.0)	0.6 (0.1)
HC47 Palliative care	$64.63	83.4%	$53.91	1.7 (0.5)	1.4 (0.0)	0.0 (0.0)	0.0 (0.0)	0.0 (0.0)	0.6 (0.1)
Reproductive health				3.9 (0.9)	3.3 (0.0)	0.0 (0.0)	0.0 (0.0)	0.0 (0.0)	1.4 (0.2)
Family planning				0.1 (0.0)	0.0 (0.0)	0.0 (0.0)	0.0 (0.0)	0.0 (0.0)	0.0 (0.0)
HC4 Contraceptives	$4.97	83.2%	$4.14	0.1 (0.0)	0.0 (0.0)	0.0 (0.0)	0.0 (0.0)	0.0 (0.0)	0.0 (0.0)
Maternal conditions				6.5 (1.5)	5.4 (0.0)	0.0 (0.0)	0.0 (0.0)	0.0 (0.0)	2.4 (0.3)
C5 Antenatal tetanus immunization	$0.39	75.2%	$0.29	0.0 (0.0)	0.0 (0.0)	0.0 (0.0)	0.0 (0.0)	0.0 (0.0)	0.0 (0.0)
HC11 Basic emergency newborn & obstetric care	$69.14	75.2%	$51.99	38.5 (8.8)	32.5 (0.0)	0.0 (0.0)	0.0 (0.0)	0.0 (0.0)	14.2 (1.8)
HC2 Post-abortion care	$4.54	75.2%	$3.42	0.1 (0.0)	0.0 (0.0)	0.0 (0.0)	0.0 (0.0)	0.0 (0.0)	0.0 (0.0)
HC3 Treatment of premature membrane rupture	$4.04	75.2%	$3.04	0.0 (0.0)	0.0 (0.0)	0.0 (0.0)	0.0 (0.0)	0.0 (0.0)	0.0 (0.0)
HC5 Kangaroo mother care counseling	$2.33	75.2%	$1.75	0.0 (0.0)	0.0 (0.0)	0.0 (0.0)	0.0 (0.0)	0.0 (0.0)	0.0 (0.0)
HC7 Medical abortion	$4.81	75.2%	$3.61	0.1 (0.0)	0.0 (0.0)	0.0 (0.0)	0.0 (0.0)	0.0 (0.0)	0.0 (0.0)
Perinatal conditions				0.1 (0.0)	0.0 (0.0)	0.0 (0.0)	0.0 (0.0)	0.0 (0.0)	0.0 (0.0)
C13 Cotrimoxazole for HIV-exposed children	$6.55	84.1%	$5.51	0.2 (0.0)	0.0 (0.0)	0.0 (0.0)	0.0 (0.0)	0.0 (0.0)	0.0 (0.0)
HC1 Antibiotics for neonatal pneumonia	$6.22	84.1%	$5.24	0.2 (0.0)	0.0 (0.0)	0.0 (0.0)	0.0 (0.0)	0.0 (0.0)	0.0 (0.0)
HC6 Neonatal sepsis, pneumonia, meningitis	$2.66	84.1%	$2.24	0.0 (0.0)	0.0 (0.0)	0.0 (0.0)	0.0 (0.0)	0.0 (0.0)	0.0 (0.0)

Table B.24. Full model results: Niger.

Niger				CHE risk 10% threshold (25% threshold)					
Disease category, disease, intervention	Cost ($)	OOP (%)	OOP ($)	Q1	Q2	Q3	Q4	Q5	Total
Childhood health				0.0 (0.0)	0.0 (0.0)	0.0 (0.0)	0.0 (0.0)	0.0 (0.0)	0.0 (0.0)
Childhood health				0.0 (0.0)	0.0 (0.0)	0.0 (0.0)	0.0 (0.0)	0.0 (0.0)	0.0 (0.0)
HC42 Acute pharyngitis treatment	$0.17	42.3%	$0.07	0.0 (0.0)	0.0 (0.0)	0.0 (0.0)	0.0 (0.0)	0.0 (0.0)	0.0 (0.0)
Infectious & parasitic diseases				9.0 (5.0)	6.8 (0.0)	5.8 (0.0)	1.5 (0.0)	0.0 (0.0)	4.6 (1.0)
Diarrheal diseases				0.6 (0.1)	0.0 (0.0)	0.0 (0.0)	0.0 (0.0)	0.0 (0.0)	0.1 (0.0)
HC12 Diagnosis & treatment of infections (IMCI)	$4.79	76.2%	$3.65	0.6 (0.1)	0.0 (0.0)	0.0 (0.0)	0.0 (0.0)	0.0 (0.0)	0.1 (0.0)

Niger				CHE risk 10% threshold (25% threshold)					
Disease category, disease, intervention	Cost ($)	OOP (%)	OOP ($)	Q1	Q2	Q3	Q4	Q5	Total
HIV/AIDS & other sexually transmitted diseases				7.7 (1.5)	2.3 (0.0)	0.0 (0.0)	0.0 (0.0)	0.0 (0.0)	2.0 (0.3)
HC13 ART & viral load monitoring	$71.45	15.7%	$11.23	1.1 (0.1)	0.0 (0.0)	0.0 (0.0)	0.0 (0.0)	0.0 (0.0)	0.2 (0.0)
HC17 Syndromic management of STI	$5.67	15.7%	$0.89	0.0 (0.0)	0.0 (0.0)	0.0 (0.0)	0.0 (0.0)	0.0 (0.0)	0.0 (0.0)
HC23 HIV, STIs, hepatitis testing & counseling	$4.31	15.7%	$0.68	0.0 (0.0)	0.0 (0.0)	0.0 (0.0)	0.0 (0.0)	0.0 (0.0)	0.0 (0.0)
HC8 HIV & syphilis PMTCT	$176.35	15.7%	$27.72	29.5 (5.9)	9.2 (0.0)	0.0 (0.0)	0.0 (0.0)	0.0 (0.0)	7.8 (1.2)
Malaria				0.0 (0.0)	0.0 (0.0)	0.0 (0.0)	0.0 (0.0)	0.0 (0.0)	0.0 (0.0)
C7 Intermittent preventive treatment (pregnancy)	$0.45	59.2%	$0.27	0.0 (0.0)	0.0 (0.0)	0.0 (0.0)	0.0 (0.0)	0.0 (0.0)	0.0 (0.0)
Tuberculosis				40.7 (33.8)	45.2 (0.0)	46.8 (0.0)	11.7 (0.0)	0.0 (0.0)	28.9 (6.8)
HC27 Diagnosis & treatment of TB	$135.09	40.3%	$54.44	40.7 (33.8)	45.2 (0.0)	46.8 (0.0)	11.7 (0.0)	0.0 (0.0)	28.9 (6.8)
Other infectious & parasitic diseases				0.1 (0.0)	0.0 (0.0)	0.0 (0.0)	0.0 (0.0)	0.0 (0.0)	0.0 (0.0)
HC30 Management & referrals for fever (IMAI)	$3.11	48.2%	$1.50	0.1 (0.0)	0.0 (0.0)	0.0 (0.0)	0.0 (0.0)	0.0 (0.0)	0.0 (0.0)
Noncommunicable diseases (NCDs)				21.9 (17.5)	22.3 (8.7)	20.1 (7.3)	17.1 (2.6)	14.8 (0.0)	19.2 (7.2)
Cardiovascular diseases				16.6 (15.1)	18.5 (9.3)	19.2 (9.6)	14.3 (8.7)	20.6 (0.0)	17.9 (8.5)
HC38 Aspirin for acute myocardial infarction	$0.03	64.7%	$0.02	0.0 (0.0)	0.0 (0.0)	0.0 (0.0)	0.0 (0.0)	0.0 (0.0)	0.0 (0.0)
HC43 Management of ischemic heart disease	$83.97	64.7%	$54.29	24.9 (20.6)	27.9 (0.0)	28.9 (0.0)	6.8 (0.0)	0.0 (0.0)	17.7 (4.1)
HC44 Management of heart failure	$249.96	64.7%	$161.61	24.8 (24.8)	27.8 (27.8)	28.8 (28.8)	36.3 (26.0)	61.8 (0.0)	35.9 (21.5)
Endocrine & metabolic disorders				15.4 (15.3)	17.3 (1.0)	17.8 (0.0)	13.3 (0.0)	0.0 (0.0)	12.8 (3.3)
HC40 Screening & management of diabetes	$64.16	96.3%	$61.76	15.4 (15.3)	17.3 (1.0)	17.8 (0.0)	13.3 (0.0)	0.0 (0.0)	12.8 (3.3)
Mental/behavioral disorders & neurological conditions				30.7 (22.9)	29.9 (11.7)	25.2 (8.9)	22.9 (0.0)	17.3 (0.0)	25.2 (8.7)
HC49 Management of bipolar disorder	$184.57	66.1%	$122.09	44.6 (44.6)	50.0 (50.0)	51.8 (44.3)	65.1 (0.1)	86.4 (0.0)	59.6 (27.8)
HC50 Management of depression	$16.11	66.1%	$10.66	8.2 (0.9)	0.0 (0.0)	0.0 (0.0)	0.0 (0.0)	0.0 (0.0)	1.6 (0.2)
HC51 Management of epilepsy	$27.53	66.1%	$18.21	11.1 (1.4)	0.0 (0.0)	0.0 (0.0)	0.0 (0.0)	0.0 (0.0)	2.2 (0.3)
HC52 Management of schizophrenia	$99.43	66.1%	$65.77	44.6 (44.6)	49.7 (8.5)	51.6 (0.0)	49.6 (0.0)	0.0 (0.0)	39.1 (10.6)
HC66 Psychosocial support & counseling	$64.63	66.1%	$42.75	44.7 (23.0)	50.0 (0.0)	22.5 (0.0)	0.0 (0.0)	0.0 (0.0)	23.4 (4.6)
Other NCDs				0.1 (0.0)	0.0 (0.0)	0.0 (0.0)	0.0 (0.0)	0.0 (0.0)	0.0 (0.0)
HC47 Palliative care	$64.63	37.2%	$24.06	0.1 (0.0)	0.0 (0.0)	0.0 (0.0)	0.0 (0.0)	0.0 (0.0)	0.0 (0.0)
Reproductive health				0.6 (0.1)	0.0 (0.0)	0.0 (0.0)	0.0 (0.0)	0.0 (0.0)	0.1 (0.0)
Family planning				0.0 (0.0)	0.0 (0.0)	0.0 (0.0)	0.0 (0.0)	0.0 (0.0)	0.0 (0.0)
HC4 Contraceptives	$4.97	22.9%	$1.14	0.0 (0.0)	0.0 (0.0)	0.0 (0.0)	0.0 (0.0)	0.0 (0.0)	0.0 (0.0)
Maternal conditions				1.0 (0.1)	0.0 (0.0)	0.0 (0.0)	0.0 (0.0)	0.0 (0.0)	0.2 (0.0)
C5 Antenatal tetanus immunization	$0.39	23.0%	$0.09	0.0 (0.0)	0.0 (0.0)	0.0 (0.0)	0.0 (0.0)	0.0 (0.0)	0.0 (0.0)
HC11 Basic emergency newborn & obstetric care	$69.14	23.0%	$15.89	6.2 (0.7)	0.0 (0.0)	0.0 (0.0)	0.0 (0.0)	0.0 (0.0)	1.2 (0.1)
HC2 Post-abortion care	$4.54	23.0%	$1.04	0.0 (0.0)	0.0 (0.0)	0.0 (0.0)	0.0 (0.0)	0.0 (0.0)	0.0 (0.0)
HC3 Treatment of premature membrane rupture	$4.04	23.0%	$0.93	0.0 (0.0)	0.0 (0.0)	0.0 (0.0)	0.0 (0.0)	0.0 (0.0)	0.0 (0.0)
HC5 Kangaroo mother care counseling	$2.33	23.0%	$0.53	0.0 (0.0)	0.0 (0.0)	0.0 (0.0)	0.0 (0.0)	0.0 (0.0)	0.0 (0.0)
HC7 Medical abortion	$4.81	23.0%	$1.10	0.1 (0.0)	0.0 (0.0)	0.0 (0.0)	0.0 (0.0)	0.0 (0.0)	0.0 (0.0)
Perinatal conditions				0.0 (0.0)	0.0 (0.0)	0.0 (0.0)	0.0 (0.0)	0.0 (0.0)	0.0 (0.0)
C13 Cotrimoxazole for HIV-exposed children	$6.55	30.9%	$2.03	0.0 (0.0)	0.0 (0.0)	0.0 (0.0)	0.0 (0.0)	0.0 (0.0)	0.0 (0.0)
HC1 Antibiotics for neonatal pneumonia	$6.22	30.9%	$1.92	0.0 (0.0)	0.0 (0.0)	0.0 (0.0)	0.0 (0.0)	0.0 (0.0)	0.0 (0.0)
HC6 Neonatal sepsis, pneumonia, meningitis	$2.66	30.9%	$0.82	0.0 (0.0)	0.0 (0.0)	0.0 (0.0)	0.0 (0.0)	0.0 (0.0)	0.0 (0.0)

Table B.25. Full model results: Nigeria.

Nigeria				CHE risk 10% threshold (25% threshold)					
Disease category, disease, intervention	Cost ($)	OOP (%)	OOP ($)	Q1	Q2	Q3	Q4	Q5	Total
Childhood health				0.0 (0.0)	0.0 (0.0)	0.0 (0.0)	0.0 (0.0)	0.0 (0.0)	0.0 (0.0)
Childhood health				0.0 (0.0)	0.0 (0.0)	0.0 (0.0)	0.0 (0.0)	0.0 (0.0)	0.0 (0.0)
HC42 Acute pharyngitis treatment	$0.24	42.3%	$0.10	0.0 (0.0)	0.0 (0.0)	0.0 (0.0)	0.0 (0.0)	0.0 (0.0)	0.0 (0.0)
Infectious & parasitic diseases				9.0 (5.0)	6.8 (0.0)	5.8 (0.0)	1.5 (0.0)	0.0 (0.0)	4.6 (1.0)
Diarrheal diseases				0.6 (0.1)	0.0 (0.0)	0.0 (0.0)	0.0 (0.0)	0.0 (0.0)	0.1 (0.0)
HC12 Diagnosis & treatment of infections (IMCI)	$10.29	79.4%	$8.17	0.6 (0.1)	0.0 (0.0)	0.0 (0.0)	0.0 (0.0)	0.0 (0.0)	0.1 (0.0)
HIV/AIDS & other sexually transmitted diseases				7.7 (1.5)	2.3 (0.0)	0.0 (0.0)	0.0 (0.0)	0.0 (0.0)	2.0 (0.3)
HC13 ART & viral load monitoring	$121.57	30.7%	$37.33	1.1 (0.1)	0.0 (0.0)	0.0 (0.0)	0.0 (0.0)	0.0 (0.0)	0.2 (0.0)
HC17 Syndromic management of STI	$10.69	30.7%	$3.28	0.0 (0.0)	0.0 (0.0)	0.0 (0.0)	0.0 (0.0)	0.0 (0.0)	0.0 (0.0)
HC23 HIV, STIs, hepatitis testing & counseling	$6.08	30.7%	$1.87	0.0 (0.0)	0.0 (0.0)	0.0 (0.0)	0.0 (0.0)	0.0 (0.0)	0.0 (0.0)
HC8 HIV & syphilis PMTCT	$313.51	30.7%	$96.26	29.5 (5.9)	9.2 (0.0)	0.0 (0.0)	0.0 (0.0)	0.0 (0.0)	7.8 (1.2)
Malaria				0.0 (0.0)	0.0 (0.0)	0.0 (0.0)	0.0 (0.0)	0.0 (0.0)	0.0 (0.0)
C7 Intermittent preventive treatment (pregnancy)	$1.02	84.2%	$0.86	0.0 (0.0)	0.0 (0.0)	0.0 (0.0)	0.0 (0.0)	0.0 (0.0)	0.0 (0.0)
Tuberculosis				40.7 (33.8)	45.2 (0.0)	46.8 (0.0)	11.7 (0.0)	0.0 (0.0)	28.9 (6.8)
HC27 Diagnosis & treatment of TB	$175.65	95.4%	$167.54	40.7 (33.8)	45.2 (0.0)	46.8 (0.0)	11.7 (0.0)	0.0 (0.0)	28.9 (6.8)
Other infectious & parasitic diseases				0.1 (0.0)	0.0 (0.0)	0.0 (0.0)	0.0 (0.0)	0.0 (0.0)	0.0 (0.0)
HC30 Management & referrals for fever (IMAI)	$6.83	74.9%	$5.12	0.1 (0.0)	0.0 (0.0)	0.0 (0.0)	0.0 (0.0)	0.0 (0.0)	0.0 (0.0)
Noncommunicable diseases (NCDs)				21.9 (17.5)	22.3 (8.7)	20.1 (7.3)	17.1 (2.6)	14.8 (0.0)	19.2 (7.2)
Cardiovascular diseases				16.6 (15.1)	18.5 (9.3)	19.2 (9.6)	14.3 (8.7)	20.6 (0.0)	17.9 (8.5)
HC38 Aspirin for acute myocardial infarction	$0.05	59.8%	$0.03	0.0 (0.0)	0.0 (0.0)	0.0 (0.0)	0.0 (0.0)	0.0 (0.0)	0.0 (0.0)
HC43 Management of ischemic heart disease	$190.17	59.8%	$113.79	24.9 (20.6)	27.9 (0.0)	28.9 (0.0)	6.8 (0.0)	0.0 (0.0)	17.7 (4.1)
HC44 Management of heart failure	$342.48	59.8%	$204.92	24.8 (24.8)	27.8 (27.8)	28.8 (28.8)	36.3 (26.0)	61.8 (0.0)	35.9 (21.5)
Endocrine & metabolic disorders				15.4 (15.3)	17.3 (1.0)	17.8 (0.0)	13.3 (0.0)	0.0 (0.0)	12.8 (3.3)
HC40 Screening & management of diabetes	$92.52	63.7%	$58.90	15.4 (15.3)	17.3 (1.0)	17.8 (0.0)	13.3 (0.0)	0.0 (0.0)	12.8 (3.3)
Mental/behavioral disorders & neurological conditions				30.7 (22.9)	29.9 (11.7)	25.2 (8.9)	22.9 (0.0)	17.3 (0.0)	25.2 (8.7)
HC49 Management of bipolar disorder	$365.39	52.2%	$190.86	44.6 (44.6)	50.0 (50.0)	51.8 (44.3)	65.1 (0.1)	86.4 (0.0)	59.6 (27.8)
HC50 Management of depression	$48.02	52.2%	$25.09	8.2 (0.9)	0.0 (0.0)	0.0 (0.0)	0.0 (0.0)	0.0 (0.0)	1.6 (0.2)
HC51 Management of epilepsy	$53.73	52.2%	$28.07	11.1 (1.4)	0.0 (0.0)	0.0 (0.0)	0.0 (0.0)	0.0 (0.0)	2.2 (0.3)
HC52 Management of schizophrenia	$329.34	52.2%	$172.03	44.6 (44.6)	49.7 (8.5)	51.6 (0.0)	49.6 (0.0)	0.0 (0.0)	39.1 (10.6)
HC66 Psychosocial support & counseling	$21.32	52.2%	$11.14	44.7 (23.0)	50.0 (0.0)	22.5 (0.0)	0.0 (0.0)	0.0 (0.0)	23.4 (4.6)
Other NCDs				0.1 (0.0)	0.0 (0.0)	0.0 (0.0)	0.0 (0.0)	0.0 (0.0)	0.0 (0.0)
HC47 Palliative care	$21.32	37.2%	$7.94	0.1 (0.0)	0.0 (0.0)	0.0 (0.0)	0.0 (0.0)	0.0 (0.0)	0.0 (0.0)
Reproductive health				0.6 (0.1)	0.0 (0.0)	0.0 (0.0)	0.0 (0.0)	0.0 (0.0)	0.1 (0.0)
Family planning				0.0 (0.0)	0.0 (0.0)	0.0 (0.0)	0.0 (0.0)	0.0 (0.0)	0.0 (0.0)
HC4 Contraceptives	$10.32	30.3%	$3.12	0.0 (0.0)	0.0 (0.0)	0.0 (0.0)	0.0 (0.0)	0.0 (0.0)	0.0 (0.0)
Maternal conditions				1.0 (0.1)	0.0 (0.0)	0.0 (0.0)	0.0 (0.0)	0.0 (0.0)	0.2 (0.0)
C5 Antenatal tetanus immunization	$0.44	69.4%	$0.30	0.0 (0.0)	0.0 (0.0)	0.0 (0.0)	0.0 (0.0)	0.0 (0.0)	0.0 (0.0)
HC11 Basic emergency newborn & obstetric care	$145.10	69.4%	$100.65	6.2 (0.7)	0.0 (0.0)	0.0 (0.0)	0.0 (0.0)	0.0 (0.0)	1.2 (0.1)

Nigeria				CHE risk 10% threshold (25% threshold)					
Disease category, disease, intervention	Cost ($)	OOP (%)	OOP ($)	Q1	Q2	Q3	Q4	Q5	Total
HC2 Post-abortion care	$8.23	69.4%	$5.71	0.0 (0.0)	0.0 (0.0)	0.0 (0.0)	0.0 (0.0)	0.0 (0.0)	0.0 (0.0)
HC3 Treatment of premature membrane rupture	$4.66	69.4%	$3.24	0.0 (0.0)	0.0 (0.0)	0.0 (0.0)	0.0 (0.0)	0.0 (0.0)	0.0 (0.0)
HC5 Kangaroo mother care counseling	$4.61	69.4%	$3.20	0.0 (0.0)	0.0 (0.0)	0.0 (0.0)	0.0 (0.0)	0.0 (0.0)	0.0 (0.0)
HC7 Medical abortion	$5.52	69.4%	$3.83	0.1 (0.0)	0.0 (0.0)	0.0 (0.0)	0.0 (0.0)	0.0 (0.0)	0.0 (0.0)
Perinatal conditions				0.0 (0.0)	0.0 (0.0)	0.0 (0.0)	0.0 (0.0)	0.0 (0.0)	0.0 (0.0)
C13 Cotrimoxazole for HIV-exposed children	$11.95	67.9%	$8.11	0.0 (0.0)	0.0 (0.0)	0.0 (0.0)	0.0 (0.0)	0.0 (0.0)	0.0 (0.0)
HC1 Antibiotics for neonatal pneumonia	$6.61	67.9%	$4.49	0.0 (0.0)	0.0 (0.0)	0.0 (0.0)	0.0 (0.0)	0.0 (0.0)	0.0 (0.0)
HC6 Neonatal sepsis, pneumonia, meningitis	$3.51	67.9%	$2.38	0.0 (0.0)	0.0 (0.0)	0.0 (0.0)	0.0 (0.0)	0.0 (0.0)	0.0 (0.0)

Table B.26. Full model results: Samoa.

Samoa				CHE risk 10% threshold (25% threshold)					
Disease category, disease, intervention	Cost ($)	OOP (%)	OOP ($)	Q1	Q2	Q3	Q4	Q5	Total
Childhood health				0.0 (0.0)	0.0 (0.0)	0.0 (0.0)	0.0 (0.0)	0.0 (0.0)	0.0 (0.0)
Childhood health				0.0 (0.0)	0.0 (0.0)	0.0 (0.0)	0.0 (0.0)	0.0 (0.0)	0.0 (0.0)
HC42 Acute pharyngitis treatment	$0.24	45.3%	$0.11	0.0 (0.0)	0.0 (0.0)	0.0 (0.0)	0.0 (0.0)	0.0 (0.0)	0.0 (0.0)
Infectious & parasitic diseases				0.8 (0.1)	0.0 (0.0)	0.0 (0.0)	0.0 (0.0)	0.0 (0.0)	0.2 (0.0)
Diarrheal diseases				0.0 (0.0)	0.0 (0.0)	0.0 (0.0)	0.0 (0.0)	0.0 (0.0)	0.0 (0.0)
HC12 Diagnosis & treatment of infections (IMCI)	$10.29	54.8%	$5.64	0.0 (0.0)	0.0 (0.0)	0.0 (0.0)	0.0 (0.0)	0.0 (0.0)	0.0 (0.0)
HIV/AIDS & other sexually transmitted diseases				0.8 (0.1)	0.0 (0.0)	0.0 (0.0)	0.0 (0.0)	0.0 (0.0)	0.2 (0.0)
HC13 ART & viral load monitoring	$121.57	13.4%	$16.30	0.1 (0.0)	0.0 (0.0)	0.0 (0.0)	0.0 (0.0)	0.0 (0.0)	0.0 (0.0)
HC17 Syndromic management of STI	$10.69	13.4%	$1.43	0.0 (0.0)	0.0 (0.0)	0.0 (0.0)	0.0 (0.0)	0.0 (0.0)	0.0 (0.0)
HC23 HIV, STIs, hepatitis testing & counseling	$6.08	13.4%	$0.82	0.0 (0.0)	0.0 (0.0)	0.0 (0.0)	0.0 (0.0)	0.0 (0.0)	0.0 (0.0)
HC8 HIV & syphilis PMTCT	$313.51	13.4%	$42.04	3.2 (0.3)	0.0 (0.0)	0.0 (0.0)	0.0 (0.0)	0.0 (0.0)	0.6 (0.1)
Malaria				0.0 (0.0)	0.0 (0.0)	0.0 (0.0)	0.0 (0.0)	0.0 (0.0)	0.0 (0.0)
C7 Intermittent preventive treatment (pregnancy)	$1.02	36.9%	$0.38	0.0 (0.0)	0.0 (0.0)	0.0 (0.0)	0.0 (0.0)	0.0 (0.0)	0.0 (0.0)
Tuberculosis				2.9 (0.3)	0.0 (0.0)	0.0 (0.0)	0.0 (0.0)	0.0 (0.0)	0.6 (0.1)
HC27 Diagnosis & treatment of TB	$175.65	23.3%	$40.86	2.9 (0.3)	0.0 (0.0)	0.0 (0.0)	0.0 (0.0)	0.0 (0.0)	0.6 (0.1)
Other infectious & parasitic diseases				0.0 (0.0)	0.0 (0.0)	0.0 (0.0)	0.0 (0.0)	0.0 (0.0)	0.0 (0.0)
HC30 Management & referrals for fever (IMAI)	$6.83	18.8%	$1.29	0.0 (0.0)	0.0 (0.0)	0.0 (0.0)	0.0 (0.0)	0.0 (0.0)	0.0 (0.0)
Noncommunicable diseases (NCDs)				0.2 (0.0)	0.0 (0.0)	0.0 (0.0)	0.0 (0.0)	0.0 (0.0)	0.0 (0.0)
Cardiovascular diseases				0.3 (0.0)	0.0 (0.0)	0.0 (0.0)	0.0 (0.0)	0.0 (0.0)	0.1 (0.0)
HC38 Aspirin for acute myocardial infarction	$0.05	12.7%	$0.01	0.0 (0.0)	0.0 (0.0)	0.0 (0.0)	0.0 (0.0)	0.0 (0.0)	0.0 (0.0)
HC43 Management of ischemic heart disease	$190.17	12.7%	$24.25	0.2 (0.0)	0.0 (0.0)	0.0 (0.0)	0.0 (0.0)	0.0 (0.0)	0.0 (0.0)
HC44 Management of heart failure	$342.48	12.7%	$43.66	0.8 (0.1)	0.0 (0.0)	0.0 (0.0)	0.0 (0.0)	0.0 (0.0)	0.2 (0.0)
Endocrine & metabolic disorders				0.0 (0.0)	0.0 (0.0)	0.0 (0.0)	0.0 (0.0)	0.0 (0.0)	0.0 (0.0)
HC40 Screening & management of diabetes	$92.52	5.9%	$5.42	0.0 (0.0)	0.0 (0.0)	0.0 (0.0)	0.0 (0.0)	0.0 (0.0)	0.0 (0.0)
Mental/behavioral disorders & neurological conditions				0.2 (0.0)	0.0 (0.0)	0.0 (0.0)	0.0 (0.0)	0.0 (0.0)	0.0 (0.0)
HC49 Management of bipolar disorder	$365.39	8.5%	$31.03	0.6 (0.1)	0.0 (0.0)	0.0 (0.0)	0.0 (0.0)	0.0 (0.0)	0.1 (0.0)

Samoa				CHE risk 10% threshold (25% threshold)					
Disease category, disease, intervention	Cost ($)	OOP (%)	OOP ($)	Q1	Q2	Q3	Q4	Q5	Total
HC50 Management of depression	$48.02	8.5%	$4.08	0.0 (0.0)	0.0 (0.0)	0.0 (0.0)	0.0 (0.0)	0.0 (0.0)	0.0 (0.0)
HC51 Management of epilepsy	$53.73	8.5%	$4.56	0.0 (0.0)	0.0 (0.0)	0.0 (0.0)	0.0 (0.0)	0.0 (0.0)	0.0 (0.0)
HC52 Management of schizophrenia	$329.34	8.5%	$27.97	0.5 (0.0)	0.0 (0.0)	0.0 (0.0)	0.0 (0.0)	0.0 (0.0)	0.1 (0.0)
HC66 Psychosocial support & counseling	$21.32	8.5%	$1.81	0.0 (0.0)	0.0 (0.0)	0.0 (0.0)	0.0 (0.0)	0.0 (0.0)	0.0 (0.0)
Other NCDs				0.0 (0.0)	0.0 (0.0)	0.0 (0.0)	0.0 (0.0)	0.0 (0.0)	0.0 (0.0)
HC47 Palliative care	$21.32	11.4%	$2.43	0.0 (0.0)	0.0 (0.0)	0.0 (0.0)	0.0 (0.0)	0.0 (0.0)	0.0 (0.0)
Reproductive health				0.0 (0.0)	0.0 (0.0)	0.0 (0.0)	0.0 (0.0)	0.0 (0.0)	0.0 (0.0)
Family planning				0.0 (0.0)	0.0 (0.0)	0.0 (0.0)	0.0 (0.0)	0.0 (0.0)	0.0 (0.0)
HC4 Contraceptives	$10.32	2.5%	$0.26	0.0 (0.0)	0.0 (0.0)	0.0 (0.0)	0.0 (0.0)	0.0 (0.0)	0.0 (0.0)
Maternal conditions				0.0 (0.0)	0.0 (0.0)	0.0 (0.0)	0.0 (0.0)	0.0 (0.0)	0.0 (0.0)
C5 Antenatal tetanus immunization	$0.44	2.2%	$0.01	0.0 (0.0)	0.0 (0.0)	0.0 (0.0)	0.0 (0.0)	0.0 (0.0)	0.0 (0.0)
HC11 Basic emergency newborn & obstetric care	$145.10	2.2%	$3.23	0.0 (0.0)	0.0 (0.0)	0.0 (0.0)	0.0 (0.0)	0.0 (0.0)	0.0 (0.0)
HC2 Post-abortion care	$8.23	2.2%	$0.18	0.0 (0.0)	0.0 (0.0)	0.0 (0.0)	0.0 (0.0)	0.0 (0.0)	0.0 (0.0)
HC3 Treatment of premature membrane rupture	$4.66	2.2%	$0.10	0.0 (0.0)	0.0 (0.0)	0.0 (0.0)	0.0 (0.0)	0.0 (0.0)	0.0 (0.0)
HC5 Kangaroo mother care counseling	$4.61	2.2%	$0.10	0.0 (0.0)	0.0 (0.0)	0.0 (0.0)	0.0 (0.0)	0.0 (0.0)	0.0 (0.0)
HC7 Medical abortion	$5.52	2.2%	$0.12	0.0 (0.0)	0.0 (0.0)	0.0 (0.0)	0.0 (0.0)	0.0 (0.0)	0.0 (0.0)
Perinatal conditions				0.0 (0.0)	0.0 (0.0)	0.0 (0.0)	0.0 (0.0)	0.0 (0.0)	0.0 (0.0)
C13 Cotrimoxazole for HIV-exposed children	$11.95	14.7%	$1.76	0.0 (0.0)	0.0 (0.0)	0.0 (0.0)	0.0 (0.0)	0.0 (0.0)	0.0 (0.0)
HC1 Antibiotics for neonatal pneumonia	$6.61	14.7%	$0.97	0.0 (0.0)	0.0 (0.0)	0.0 (0.0)	0.0 (0.0)	0.0 (0.0)	0.0 (0.0)
HC6 Neonatal sepsis, pneumonia, meningitis	$3.51	14.7%	$0.52	0.0 (0.0)	0.0 (0.0)	0.0 (0.0)	0.0 (0.0)	0.0 (0.0)	0.0 (0.0)

Table B.27. Full model results: São Tomé & Príncipe.

São Tomé & Príncipe				CHE risk 10% threshold (25% threshold)					
Disease category, disease, intervention	Cost ($)	OOP (%)	OOP ($)	Q1	Q2	Q3	Q4	Q5	Total
Childhood health				0.0 (0.0)	0.0 (0.0)	0.0 (0.0)	0.0 (0.0)	0.0 (0.0)	0.0 (0.0)
Childhood health				0.0 (0.0)	0.0 (0.0)	0.0 (0.0)	0.0 (0.0)	0.0 (0.0)	0.0 (0.0)
HC42 Acute pharyngitis treatment	$0.24	45.3%	$0.11	0.0 (0.0)	0.0 (0.0)	0.0 (0.0)	0.0 (0.0)	0.0 (0.0)	0.0 (0.0)
Infectious & parasitic diseases				0.0 (0.0)	0.0 (0.0)	0.0 (0.0)	0.0 (0.0)	0.0 (0.0)	0.0 (0.0)
Diarrheal diseases				0.0 (0.0)	0.0 (0.0)	0.0 (0.0)	0.0 (0.0)	0.0 (0.0)	0.0 (0.0)
HC12 Diagnosis & treatment of infections (IMCI)	$10.29	8.8%	$0.90	0.0 (0.0)	0.0 (0.0)	0.0 (0.0)	0.0 (0.0)	0.0 (0.0)	0.0 (0.0)
HIV/AIDS & other sexually transmitted diseases				0.1 (0.0)	0.0 (0.0)	0.0 (0.0)	0.0 (0.0)	0.0 (0.0)	0.0 (0.0)
HC13 ART & viral load monitoring	$121.57	1.6%	$1.94	0.0 (0.0)	0.0 (0.0)	0.0 (0.0)	0.0 (0.0)	0.0 (0.0)	0.0 (0.0)
HC17 Syndromic management of STI	$10.69	1.6%	$0.17	0.0 (0.0)	0.0 (0.0)	0.0 (0.0)	0.0 (0.0)	0.0 (0.0)	0.0 (0.0)
HC23 HIV, STIs, hepatitis testing & counseling	$6.08	1.6%	$0.10	0.0 (0.0)	0.0 (0.0)	0.0 (0.0)	0.0 (0.0)	0.0 (0.0)	0.0 (0.0)
HC8 HIV & syphilis PMTCT	$313.51	1.6%	$5.00	0.2 (0.0)	0.0 (0.0)	0.0 (0.0)	0.0 (0.0)	0.0 (0.0)	0.0 (0.0)
Malaria				0.0 (0.0)	0.0 (0.0)	0.0 (0.0)	0.0 (0.0)	0.0 (0.0)	0.0 (0.0)
C7 Intermittent preventive treatment (pregnancy)	$1.02	8.1%	$0.08	0.0 (0.0)	0.0 (0.0)	0.0 (0.0)	0.0 (0.0)	0.0 (0.0)	0.0 (0.0)
Tuberculosis				0.0 (0.0)	0.0 (0.0)	0.0 (0.0)	0.0 (0.0)	0.0 (0.0)	0.0 (0.0)

São Tomé & Príncipe				CHE risk 10% threshold (25% threshold)					
Disease category, disease, intervention	Cost ($)	OOP (%)	OOP ($)	Q1	Q2	Q3	Q4	Q5	Total
HC27 Diagnosis & treatment of TB	$175.65	0.1%	$0.23	0.0 (0.0)	0.0 (0.0)	0.0 (0.0)	0.0 (0.0)	0.0 (0.0)	0.0 (0.0)
Other infectious & parasitic diseases				0.0 (0.0)	0.0 (0.0)	0.0 (0.0)	0.0 (0.0)	0.0 (0.0)	0.0 (0.0)
HC30 Management & referrals for fever (IMAI)	$6.83	11.9%	$0.81	0.0 (0.0)	0.0 (0.0)	0.0 (0.0)	0.0 (0.0)	0.0 (0.0)	0.0 (0.0)
Noncommunicable diseases (NCDs)				1.0 (0.1)	0.0 (0.0)	0.0 (0.0)	0.0 (0.0)	0.0 (0.0)	0.2 (0.0)
Cardiovascular diseases				3.3 (0.4)	0.0 (0.0)	0.0 (0.0)	0.0 (0.0)	0.0 (0.0)	0.7 (0.1)
HC38 Aspirin for acute myocardial infarction	$0.05	8.7%	$0.00	0.0 (0.0)	0.0 (0.0)	0.0 (0.0)	0.0 (0.0)	0.0 (0.0)	0.0 (0.0)
HC43 Management of ischemic heart disease	$190.17	8.7%	$16.47	2.2 (0.3)	0.0 (0.0)	0.0 (0.0)	0.0 (0.0)	0.0 (0.0)	0.4 (0.1)
HC44 Management of heart failure	$342.48	8.7%	$29.67	7.7 (1.0)	0.0 (0.0)	0.0 (0.0)	0.0 (0.0)	0.0 (0.0)	1.5 (0.2)
Endocrine & metabolic disorders				0.1 (0.0)	0.0 (0.0)	0.0 (0.0)	0.0 (0.0)	0.0 (0.0)	0.0 (0.0)
HC40 Screening & management of diabetes	$92.52	8.9%	$8.22	0.1 (0.0)	0.0 (0.0)	0.0 (0.0)	0.0 (0.0)	0.0 (0.0)	0.0 (0.0)
Mental/behavioral disorders & neurological conditions				0.0 (0.0)	0.0 (0.0)	0.0 (0.0)	0.0 (0.0)	0.0 (0.0)	0.0 (0.0)
HC49 Management of bipolar disorder	$365.39	2.3%	$8.25	0.1 (0.0)	0.0 (0.0)	0.0 (0.0)	0.0 (0.0)	0.0 (0.0)	0.0 (0.0)
HC50 Management of depression	$48.02	2.3%	$1.08	0.0 (0.0)	0.0 (0.0)	0.0 (0.0)	0.0 (0.0)	0.0 (0.0)	0.0 (0.0)
HC51 Management of epilepsy	$53.73	2.3%	$1.21	0.0 (0.0)	0.0 (0.0)	0.0 (0.0)	0.0 (0.0)	0.0 (0.0)	0.0 (0.0)
HC52 Management of schizophrenia	$329.34	2.3%	$7.44	0.1 (0.0)	0.0 (0.0)	0.0 (0.0)	0.0 (0.0)	0.0 (0.0)	0.0 (0.0)
HC66 Psychosocial support & counseling	$21.32	2.3%	$0.48	0.0 (0.0)	0.0 (0.0)	0.0 (0.0)	0.0 (0.0)	0.0 (0.0)	0.0 (0.0)
Other NCDs				0.1 (0.0)	0.0 (0.0)	0.0 (0.0)	0.0 (0.0)	0.0 (0.0)	0.0 (0.0)
HC47 Palliative care	$21.32	44.4%	$9.46	0.1 (0.0)	0.0 (0.0)	0.0 (0.0)	0.0 (0.0)	0.0 (0.0)	0.0 (0.0)
Reproductive health				3.3 (1.9)	4.7 (0.0)	3.1 (0.0)	0.0 (0.0)	0.0 (0.0)	2.2 (0.4)
Family planning				0.0 (0.0)	0.0 (0.0)	0.0 (0.0)	0.0 (0.0)	0.0 (0.0)	0.0 (0.0)
HC4 Contraceptives	$10.32	1.2%	$0.12	0.0 (0.0)	0.0 (0.0)	0.0 (0.0)	0.0 (0.0)	0.0 (0.0)	0.0 (0.0)
Maternal conditions				5.4 (3.2)	7.8 (0.0)	5.2 (0.0)	0.0 (0.0)	0.0 (0.0)	3.7 (0.6)
C5 Antenatal tetanus immunization	$0.44	76.4%	$0.33	0.0 (0.0)	0.0 (0.0)	0.0 (0.0)	0.0 (0.0)	0.0 (0.0)	0.0 (0.0)
HC11 Basic emergency newborn & obstetric care	$145.10	76.4%	$110.80	31.8 (19.2)	46.9 (0.0)	31.3 (0.0)	0.0 (0.0)	0.0 (0.0)	22.0 (3.8)
HC2 Post-abortion care	$8.23	76.4%	$6.28	0.3 (0.0)	0.0 (0.0)	0.0 (0.0)	0.0 (0.0)	0.0 (0.0)	0.1 (0.0)
HC3 Treatment of premature membrane rupture	$4.66	76.4%	$3.56	0.1 (0.0)	0.0 (0.0)	0.0 (0.0)	0.0 (0.0)	0.0 (0.0)	0.0 (0.0)
HC5 Kangaroo mother care counseling	$4.61	76.4%	$3.52	0.1 (0.0)	0.0 (0.0)	0.0 (0.0)	0.0 (0.0)	0.0 (0.0)	0.0 (0.0)
HC7 Medical abortion	$5.52	76.4%	$4.21	0.2 (0.0)	0.0 (0.0)	0.0 (0.0)	0.0 (0.0)	0.0 (0.0)	0.0 (0.0)
Perinatal conditions				0.1 (0.0)	0.0 (0.0)	0.0 (0.0)	0.0 (0.0)	0.0 (0.0)	0.0 (0.0)
C13 Cotrimoxazole for HIV-exposed children	$11.95	49.4%	$5.91	0.2 (0.0)	0.0 (0.0)	0.0 (0.0)	0.0 (0.0)	0.0 (0.0)	0.0 (0.0)
HC1 Antibiotics for neonatal pneumonia	$6.61	49.4%	$3.27	0.0 (0.0)	0.0 (0.0)	0.0 (0.0)	0.0 (0.0)	0.0 (0.0)	0.0 (0.0)
HC6 Neonatal sepsis, pneumonia, meningitis	$3.51	49.4%	$1.74	0.0 (0.0)	0.0 (0.0)	0.0 (0.0)	0.0 (0.0)	0.0 (0.0)	0.0 (0.0)

Table B.28. Full model results: Senegal.

Senegal				CHE risk 10% threshold (25% threshold)					
Disease category, disease, intervention	Cost ($)	OOP (%)	OOP ($)	Q1	Q2	Q3	Q4	Q5	Total
Childhood health				0.0 (0.0)	0.0 (0.0)	0.0 (0.0)	0.0 (0.0)	0.0 (0.0)	0.0 (0.0)
Childhood health				0.0 (0.0)	0.0 (0.0)	0.0 (0.0)	0.0 (0.0)	0.0 (0.0)	0.0 (0.0)

Senegal				CHE risk<br>10% threshold (25% threshold)					
Disease category, disease, intervention	Cost ($)	OOP (%)	OOP ($)	Q1	Q2	Q3	Q4	Q5	Total
HC42 Acute pharyngitis treatment	$0.24	42.3%	$0.10	0.0 (0.0)	0.0 (0.0)	0.0 (0.0)	0.0 (0.0)	0.0 (0.0)	0.0 (0.0)
Infectious & parasitic diseases				15.0 (8.2)	17.3 (0.0)	12.0 (0.0)	0.1 (0.0)	0.0 (0.0)	8.9 (1.6)
Diarrheal diseases				0.2 (0.0)	0.0 (0.0)	0.0 (0.0)	0.0 (0.0)	0.0 (0.0)	0.0 (0.0)
HC12 Diagnosis & treatment of infections (IMCI)	$10.29	52.4%	$5.40	0.2 (0.0)	0.0 (0.0)	0.0 (0.0)	0.0 (0.0)	0.0 (0.0)	0.0 (0.0)
HIV/AIDS & other sexually transmitted diseases				17.1 (10.0)	17.5 (0.0)	17.6 (0.0)	0.2 (0.0)	0.0 (0.0)	10.5 (2.0)
HC13 ART & viral load monitoring	$121.57	38.1%	$46.26	15.8 (2.2)	0.0 (0.0)	0.0 (0.0)	0.0 (0.0)	0.0 (0.0)	3.2 (0.4)
HC17 Syndromic management of STI	$10.69	38.1%	$4.07	0.0 (0.0)	0.0 (0.0)	0.0 (0.0)	0.0 (0.0)	0.0 (0.0)	0.0 (0.0)
HC23 HIV, STIs, hepatitis testing & counseling	$6.08	38.1%	$2.31	0.0 (0.0)	0.0 (0.0)	0.0 (0.0)	0.0 (0.0)	0.0 (0.0)	0.0 (0.0)
HC8 HIV & syphilis PMTCT	$313.51	38.1%	$119.29	52.7 (37.9)	70.0 (0.0)	70.3 (0.0)	0.6 (0.0)	0.0 (0.0)	38.7 (7.6)
Malaria				0.0 (0.0)	0.0 (0.0)	0.0 (0.0)	0.0 (0.0)	0.0 (0.0)	0.0 (0.0)
C7 Intermittent preventive treatment (pregnancy)	$1.02	36.1%	$0.37	0.0 (0.0)	0.0 (0.0)	0.0 (0.0)	0.0 (0.0)	0.0 (0.0)	0.0 (0.0)
Tuberculosis				51.2 (25.4)	68.1 (0.0)	25.7 (0.0)	0.0 (0.0)	0.0 (0.0)	29.0 (5.1)
HC27 Diagnosis & treatment of TB	$175.65	56.2%	$98.80	51.2 (25.4)	68.1 (0.0)	25.7 (0.0)	0.0 (0.0)	0.0 (0.0)	29.0 (5.1)
Other infectious & parasitic diseases				0.1 (0.0)	0.0 (0.0)	0.0 (0.0)	0.0 (0.0)	0.0 (0.0)	0.0 (0.0)
HC30 Management & referrals for fever (IMAI)	$6.83	46.3%	$3.16	0.1 (0.0)	0.0 (0.0)	0.0 (0.0)	0.0 (0.0)	0.0 (0.0)	0.0 (0.0)
Noncommunicable diseases (NCDs)				17.4 (12.7)	16.5 (14.6)	18.0 (4.9)	17.3 (0.0)	11.3 (0.0)	16.1 (6.4)
Cardiovascular diseases				9.8 (8.6)	13.0 (6.4)	14.0 (0.3)	8.0 (0.0)	4.0 (0.0)	9.8 (3.1)
HC38 Aspirin for acute myocardial infarction	$0.05	64.7%	$0.03	0.0 (0.0)	0.0 (0.0)	0.0 (0.0)	0.0 (0.0)	0.0 (0.0)	0.0 (0.0)
HC43 Management of ischemic heart disease	$190.17	64.7%	$122.99	14.6 (11.1)	19.3 (0.0)	20.7 (0.0)	1.0 (0.0)	0.0 (0.0)	11.1 (2.2)
HC44 Management of heart failure	$342.48	64.7%	$221.49	14.8 (14.8)	19.6 (19.2)	21.4 (1.0)	22.9 (0.0)	12.1 (0.0)	18.1 (7.0)
Endocrine & metabolic disorders				2.4 (0.4)	0.0 (0.0)	0.0 (0.0)	0.0 (0.0)	0.0 (0.0)	0.5 (0.1)
HC40 Screening & management of diabetes	$92.52	60.5%	$55.97	2.4 (0.4)	0.0 (0.0)	0.0 (0.0)	0.0 (0.0)	0.0 (0.0)	0.5 (0.1)
Mental/behavioral disorders & neurological conditions				28.4 (20.2)	25.3 (25.3)	27.6 (9.7)	29.8 (0.0)	20.2 (0.0)	26.3 (11.0)
HC49 Management of bipolar disorder	$365.39	71.3%	$260.67	47.5 (47.5)	63.2 (63.2)	69.0 (35.0)	74.6 (0.0)	55.2 (0.0)	61.9 (29.1)
HC50 Management of depression	$48.02	71.3%	$34.26	17.6 (2.2)	0.0 (0.0)	0.0 (0.0)	0.0 (0.0)	0.0 (0.0)	3.5 (0.4)
HC51 Management of epilepsy	$53.73	71.3%	$38.33	26.3 (3.4)	0.0 (0.0)	0.0 (0.0)	0.0 (0.0)	0.0 (0.0)	5.3 (0.7)
HC52 Management of schizophrenia	$329.34	71.3%	$234.95	47.6 (47.6)	63.3 (63.2)	69.0 (13.5)	74.7 (0.0)	45.8 (0.0)	60.1 (24.9)
HC66 Psychosocial support & counseling	$21.32	71.3%	$15.21	2.8 (0.3)	0.0 (0.0)	0.0 (0.0)	0.0 (0.0)	0.0 (0.0)	0.6 (0.1)
Other NCDs				0.0 (0.0)	0.0 (0.0)	0.0 (0.0)	0.0 (0.0)	0.0 (0.0)	0.0 (0.0)
HC47 Palliative care	$21.32	47.1%	$10.04	0.0 (0.0)	0.0 (0.0)	0.0 (0.0)	0.0 (0.0)	0.0 (0.0)	0.0 (0.0)
Reproductive health				5.0 (2.4)	6.8 (0.0)	2.8 (0.0)	0.0 (0.0)	0.0 (0.0)	2.9 (0.5)
Family planning				0.0 (0.0)	0.0 (0.0)	0.0 (0.0)	0.0 (0.0)	0.0 (0.0)	0.0 (0.0)
HC4 Contraceptives	$10.32	9.7%	$1.00	0.0 (0.0)	0.0 (0.0)	0.0 (0.0)	0.0 (0.0)	0.0 (0.0)	0.0 (0.0)
Maternal conditions				8.3 (4.0)	11.3 (0.0)	4.7 (0.0)	0.0 (0.0)	0.0 (0.0)	4.9 (0.8)
C5 Antenatal tetanus immunization	$0.44	67.5%	$0.29	0.0 (0.0)	0.0 (0.0)	0.0 (0.0)	0.0 (0.0)	0.0 (0.0)	0.0 (0.0)
HC11 Basic emergency newborn & obstetric care	$145.10	67.5%	$97.98	49.5 (24.1)	68.0 (0.0)	28.3 (0.0)	0.0 (0.0)	0.0 (0.0)	29.2 (4.8)
HC2 Post-abortion care	$8.23	67.5%	$5.56	0.2 (0.0)	0.0 (0.0)	0.0 (0.0)	0.0 (0.0)	0.0 (0.0)	0.0 (0.0)
HC3 Treatment of premature membrane rupture	$4.66	67.5%	$3.15	0.1 (0.0)	0.0 (0.0)	0.0 (0.0)	0.0 (0.0)	0.0 (0.0)	0.0 (0.0)
HC5 Kangaroo mother care counseling	$4.61	67.5%	$3.11	0.1 (0.0)	0.0 (0.0)	0.0 (0.0)	0.0 (0.0)	0.0 (0.0)	0.0 (0.0)
HC7 Medical abortion	$5.52	67.5%	$3.73	0.2 (0.0)	0.0 (0.0)	0.0 (0.0)	0.0 (0.0)	0.0 (0.0)	0.0 (0.0)

**Senegal**				**CHE risk**<br>10% threshold (25% threshold)					
Disease category, disease, intervention	Cost ($)	OOP (%)	OOP ($)	Q1	Q2	Q3	Q4	Q5	Total
Perinatal conditions				0.1 (0.0)	0.0 (0.0)	0.0 (0.0)	0.0 (0.0)	0.0 (0.0)	0.0 (0.0)
C13 Cotrimoxazole for HIV-exposed children	$11.95	74.2%	$8.86	0.3 (0.0)	0.0 (0.0)	0.0 (0.0)	0.0 (0.0)	0.0 (0.0)	0.1 (0.0)
HC1 Antibiotics for neonatal pneumonia	$6.61	74.2%	$4.90	0.1 (0.0)	0.0 (0.0)	0.0 (0.0)	0.0 (0.0)	0.0 (0.0)	0.0 (0.0)
HC6 Neonatal sepsis, pneumonia, meningitis	$3.51	74.2%	$2.60	0.0 (0.0)	0.0 (0.0)	0.0 (0.0)	0.0 (0.0)	0.0 (0.0)	0.0 (0.0)

Table B.29. Full model results: Sierra Leone.

**Sierra Leone**				**CHE risk**<br>10% threshold (25% threshold)					
Disease category, disease, intervention	Cost ($)	OOP (%)	OOP ($)	Q1	Q2	Q3	Q4	Q5	Total
Childhood health				0.0 (0.0)	0.0 (0.0)	0.0 (0.0)	0.0 (0.0)	0.0 (0.0)	0.0 (0.0)
Childhood health				0.0 (0.0)	0.0 (0.0)	0.0 (0.0)	0.0 (0.0)	0.0 (0.0)	0.0 (0.0)
HC42 Acute pharyngitis treatment	$0.17	42.3%	$0.07	0.0 (0.0)	0.0 (0.0)	0.0 (0.0)	0.0 (0.0)	0.0 (0.0)	0.0 (0.0)
Infectious & parasitic diseases				18.6 (15.8)	17.3 (9.3)	16.2 (0.0)	18.4 (0.0)	5.5 (0.0)	15.2 (5.0)
Diarrheal diseases				0.4 (0.0)	0.0 (0.0)	0.0 (0.0)	0.0 (0.0)	0.0 (0.0)	0.1 (0.0)
HC12 Diagnosis & treatment of infections (IMCI)	$4.79	65.9%	$3.16	0.4 (0.0)	0.0 (0.0)	0.0 (0.0)	0.0 (0.0)	0.0 (0.0)	0.1 (0.0)
HIV/AIDS & other sexually transmitted diseases				20.0 (14.5)	16.9 (6.0)	13.9 (0.0)	15.8 (0.0)	2.6 (0.0)	13.8 (4.1)
HC13 ART & viral load monitoring	$71.45	50.7%	$36.24	28.6 (6.6)	14.3 (0.0)	0.0 (0.0)	0.0 (0.0)	0.0 (0.0)	8.6 (1.3)
HC17 Syndromic management of STI	$5.67	50.7%	$2.88	0.0 (0.0)	0.0 (0.0)	0.0 (0.0)	0.0 (0.0)	0.0 (0.0)	0.0 (0.0)
HC23 HIV, STIs, hepatitis testing & counseling	$4.31	50.7%	$2.19	0.1 (0.0)	0.0 (0.0)	0.0 (0.0)	0.0 (0.0)	0.0 (0.0)	0.0 (0.0)
HC8 HIV & syphilis PMTCT	$176.35	50.7%	$89.44	51.4 (51.4)	53.2 (24.1)	55.8 (0.0)	63.3 (0.0)	10.4 (0.0)	46.8 (15.1)
Malaria				0.0 (0.0)	0.0 (0.0)	0.0 (0.0)	0.0 (0.0)	0.0 (0.0)	0.0 (0.0)
C7 Intermittent preventive treatment (pregnancy)	$0.45	66.0%	$0.30	0.0 (0.0)	0.0 (0.0)	0.0 (0.0)	0.0 (0.0)	0.0 (0.0)	0.0 (0.0)
Tuberculosis				68.4 (68.4)	70.9 (50.2)	74.2 (0.0)	84.2 (0.0)	33.2 (0.0)	66.2 (23.7)
HC27 Diagnosis & treatment of TB	$135.09	73.1%	$98.78	68.4 (68.4)	70.9 (50.2)	74.2 (0.0)	84.2 (0.0)	33.2 (0.0)	66.2 (23.7)
Other infectious & parasitic diseases				0.1 (0.0)	0.0 (0.0)	0.0 (0.0)	0.0 (0.0)	0.0 (0.0)	0.0 (0.0)
HC30 Management & referrals for fever (IMAI)	$3.11	63.6%	$1.98	0.1 (0.0)	0.0 (0.0)	0.0 (0.0)	0.0 (0.0)	0.0 (0.0)	0.0 (0.0)
Noncommunicable diseases (NCDs)				25.4 (14.8)	21.1 (7.1)	14.3 (3.2)	10.9 (0.1)	6.0 (0.0)	15.5 (5.0)
Cardiovascular diseases				20.6 (15.2)	21.4 (10.7)	15.8 (10.7)	12.8 (0.3)	12.0 (0.0)	16.5 (7.4)
HC38 Aspirin for acute myocardial infarction	$0.03	59.8%	$0.02	0.0 (0.0)	0.0 (0.0)	0.0 (0.0)	0.0 (0.0)	0.0 (0.0)	0.0 (0.0)
HC43 Management of ischemic heart disease	$83.97	59.8%	$50.24	30.9 (14.9)	32.1 (0.0)	13.9 (0.0)	0.0 (0.0)	0.0 (0.0)	15.4 (3.0)
HC44 Management of heart failure	$249.96	59.8%	$149.56	30.8 (30.8)	32.1 (32.1)	33.6 (32.0)	38.3 (0.8)	35.9 (0.0)	34.1 (19.1)
Endocrine & metabolic disorders				9.5 (2.9)	8.0 (0.0)	0.0 (0.0)	0.0 (0.0)	0.0 (0.0)	3.5 (0.6)
HC40 Screening & management of diabetes	$64.16	63.7%	$40.85	9.5 (2.9)	8.0 (0.0)	0.0 (0.0)	0.0 (0.0)	0.0 (0.0)	3.5 (0.6)
Mental/behavioral disorders & neurological conditions				36.5 (19.9)	27.7 (7.7)	19.0 (0.0)	14.2 (0.0)	4.8 (0.0)	20.5 (5.5)
HC49 Management of bipolar disorder	$184.57	52.2%	$96.41	57.7 (57.7)	60.1 (38.6)	62.7 (0.0)	71.0 (0.0)	24.2 (0.0)	55.1 (19.3)
HC50 Management of depression	$16.11	52.2%	$8.42	3.6 (0.3)	0.0 (0.0)	0.0 (0.0)	0.0 (0.0)	0.0 (0.0)	0.7 (0.1)
HC51 Management of epilepsy	$27.53	52.2%	$14.38	5.6 (0.6)	0.0 (0.0)	0.0 (0.0)	0.0 (0.0)	0.0 (0.0)	1.1 (0.1)
HC52 Management of schizophrenia	$99.43	52.2%	$51.94	57.6 (29.5)	59.7 (0.0)	32.5 (0.0)	0.0 (0.0)	0.0 (0.0)	30.0 (5.9)
HC66 Psychosocial support & counseling	$64.63	52.2%	$33.76	57.8 (11.3)	18.8 (0.0)	0.0 (0.0)	0.0 (0.0)	0.0 (0.0)	15.3 (2.3)

Sierra Leone				CHE risk 10% threshold (25% threshold)					
Disease category, disease, intervention	Cost ($)	OOP (%)	OOP ($)	Q1	Q2	Q3	Q4	Q5	Total
Other NCDs				0.1 (0.0)	0.0 (0.0)	0.0 (0.0)	0.0 (0.0)	0.0 (0.0)	0.0 (0.0)
HC47 Palliative care	$64.63	37.2%	$24.06	0.1 (0.0)	0.0 (0.0)	0.0 (0.0)	0.0 (0.0)	0.0 (0.0)	0.0 (0.0)
Reproductive health				8.6 (2.9)	7.9 (0.0)	0.1 (0.0)	0.0 (0.0)	0.0 (0.0)	3.3 (0.6)
Family planning				0.1 (0.0)	0.0 (0.0)	0.0 (0.0)	0.0 (0.0)	0.0 (0.0)	0.0 (0.0)
HC4 Contraceptives	$4.97	70.9%	$3.52	0.1 (0.0)	0.0 (0.0)	0.0 (0.0)	0.0 (0.0)	0.0 (0.0)	0.0 (0.0)
Maternal conditions				14.2 (4.9)	13.1 (0.0)	0.2 (0.0)	0.0 (0.0)	0.0 (0.0)	5.5 (1.0)
C5 Antenatal tetanus immunization	$0.39	62.6%	$0.24	0.0 (0.0)	0.0 (0.0)	0.0 (0.0)	0.0 (0.0)	0.0 (0.0)	0.0 (0.0)
HC11Basic emergency newborn & obstetric care	$69.14	62.6%	$43.26	84.2 (29.2)	78.6 (0.0)	1.4 (0.0)	0.0 (0.0)	0.0 (0.0)	32.9 (5.8)
HC2 Post-abortion care	$4.54	62.6%	$2.84	0.3 (0.0)	0.0 (0.0)	0.0 (0.0)	0.0 (0.0)	0.0 (0.0)	0.1 (0.0)
HC3 Treatment of premature membrane rupture	$4.04	62.6%	$2.53	0.2 (0.0)	0.0 (0.0)	0.0 (0.0)	0.0 (0.0)	0.0 (0.0)	0.0 (0.0)
HC5 Kangaroo mother care counseling	$2.33	62.6%	$1.46	0.0 (0.0)	0.0 (0.0)	0.0 (0.0)	0.0 (0.0)	0.0 (0.0)	0.0 (0.0)
HC7 Medical abortion	$4.81	62.6%	$3.01	0.4 (0.0)	0.0 (0.0)	0.0 (0.0)	0.0 (0.0)	0.0 (0.0)	0.1 (0.0)
Perinatal conditions				0.1 (0.0)	0.0 (0.0)	0.0 (0.0)	0.0 (0.0)	0.0 (0.0)	0.0 (0.0)
C13 Cotrimoxazole for HIV-exposed children	$6.55	53.1%	$3.48	0.2 (0.0)	0.0 (0.0)	0.0 (0.0)	0.0 (0.0)	0.0 (0.0)	0.0 (0.0)
HC1 Antibiotics for neonatal pneumonia	$6.22	53.1%	$3.30	0.2 (0.0)	0.0 (0.0)	0.0 (0.0)	0.0 (0.0)	0.0 (0.0)	0.0 (0.0)
HC6 Neonatal sepsis, pneumonia, meningitis	$2.66	53.1%	$1.41	0.0 (0.0)	0.0 (0.0)	0.0 (0.0)	0.0 (0.0)	0.0 (0.0)	0.0 (0.0)

Table B.30. Full model results: Tajikistan.

Tajikistan				CHE risk 10% threshold (25% threshold)					
Disease category, disease, intervention	Cost ($)	OOP (%)	OOP ($)	Q1	Q2	Q3	Q4	Q5	Total
Childhood health				0.0 (0.0)	0.0 (0.0)	0.0 (0.0)	0.0 (0.0)	0.0 (0.0)	0.0 (0.0)
Childhood health				0.0 (0.0)	0.0 (0.0)	0.0 (0.0)	0.0 (0.0)	0.0 (0.0)	0.0 (0.0)
HC42 Acute pharyngitis treatment	$0.24	45.3%	$0.11	0.0 (0.0)	0.0 (0.0)	0.0 (0.0)	0.0 (0.0)	0.0 (0.0)	0.0 (0.0)
Infectious & parasitic diseases				7.2 (0.7)	0.0 (0.0)	0.0 (0.0)	0.0 (0.0)	0.0 (0.0)	1.4 (0.1)
Diarrheal diseases				0.2 (0.0)	0.0 (0.0)	0.0 (0.0)	0.0 (0.0)	0.0 (0.0)	0.0 (0.0)
HC12 Diagnosis & treatment of infections (IMCI)	$10.29	69.9%	$7.19	0.2 (0.0)	0.0 (0.0)	0.0 (0.0)	0.0 (0.0)	0.0 (0.0)	0.0 (0.0)
HIV/AIDS & other sexually transmitted diseases				10.1 (1.1)	0.0 (0.0)	0.0 (0.0)	0.0 (0.0)	0.0 (0.0)	2.0 (0.2)
HC13 ART & viral load monitoring	$121.57	13.9%	$16.84	0.6 (0.0)	0.0 (0.0)	0.0 (0.0)	0.0 (0.0)	0.0 (0.0)	0.1 (0.0)
HC17 Syndromic management of STI	$10.69	13.9%	$1.48	0.0 (0.0)	0.0 (0.0)	0.0 (0.0)	0.0 (0.0)	0.0 (0.0)	0.0 (0.0)
HC23 HIV, STIs, hepatitis testing & counseling	$6.08	13.9%	$0.84	0.0 (0.0)	0.0 (0.0)	0.0 (0.0)	0.0 (0.0)	0.0 (0.0)	0.0 (0.0)
HC8 HIV & syphilis PMTCT	$313.51	13.9%	$43.42	39.9 (4.3)	0.0 (0.0)	0.0 (0.0)	0.0 (0.0)	0.0 (0.0)	8.0 (0.9)
Malaria				0.0 (0.0)	0.0 (0.0)	0.0 (0.0)	0.0 (0.0)	0.0 (0.0)	0.0 (0.0)
C7 Intermittent preventive treatment (pregnancy)	$1.02	15.0%	$0.15	0.0 (0.0)	0.0 (0.0)	0.0 (0.0)	0.0 (0.0)	0.0 (0.0)	0.0 (0.0)
Tuberculosis				16.7 (1.6)	0.0 (0.0)	0.0 (0.0)	0.0 (0.0)	0.0 (0.0)	3.3 (0.3)
HC27 Diagnosis & treatment of TB	$175.65	18.2%	$32.02	16.7 (1.6)	0.0 (0.0)	0.0 (0.0)	0.0 (0.0)	0.0 (0.0)	3.3 (0.3)
Other infectious & parasitic diseases				0.0 (0.0)	0.0 (0.0)	0.0 (0.0)	0.0 (0.0)	0.0 (0.0)	0.0 (0.0)
HC30 Management & referrals for fever (IMAI)	$6.83	28.1%	$1.92	0.0 (0.0)	0.0 (0.0)	0.0 (0.0)	0.0 (0.0)	0.0 (0.0)	0.0 (0.0)
Noncommunicable diseases (NCDs)				17.8 (15.0)	15.1 (12.4)	16.2 (2.8)	16.6 (0.0)	11.7 (0.0)	15.5 (6.0)

Tajikistan				CHE risk 10% threshold (25% threshold)					
Disease category, disease, intervention	Cost ($)	OOP (%)	OOP ($)	Q1	Q2	Q3	Q4	Q5	Total
Cardiovascular diseases				14.0 (13.8)	14.3 (7.3)	15.5 (4.6)	13.8 (0.0)	8.1 (0.0)	13.1 (5.1)
HC38 Aspirin for acute myocardial infarction	$0.05	78.4%	$0.04	0.0 (0.0)	0.0 (0.0)	0.0 (0.0)	0.0 (0.0)	0.0 (0.0)	0.0 (0.0)
HC43 Management of ischemic heart disease	$190.17	78.4%	$149.19	21.1 (20.5)	21.4 (0.5)	23.2 (0.0)	16.3 (0.0)	0.0 (0.0)	16.4 (4.2)
HC44 Management of heart failure	$342.48	78.4%	$268.67	20.9 (20.9)	21.4 (21.4)	23.2 (13.7)	25.0 (0.0)	24.3 (0.0)	23.0 (11.2)
Endocrine & metabolic disorders				7.0 (1.2)	1.6 (0.0)	0.0 (0.0)	0.0 (0.0)	0.0 (0.0)	1.7 (0.2)
HC40 Screening & management of diabetes	$92.52	71.9%	$66.50	7.0 (1.2)	1.6 (0.0)	0.0 (0.0)	0.0 (0.0)	0.0 (0.0)	1.7 (0.2)
Mental/behavioral disorders & neurological conditions				25.7 (21.4)	21.4 (20.5)	23.2 (2.9)	24.9 (0.0)	18.6 (0.0)	22.8 (9.0)
HC49 Management of bipolar disorder	$365.39	66.0%	$241.31	52.4 (52.4)	53.6 (53.6)	57.9 (13.9)	62.3 (0.0)	52.0 (0.0)	55.7 (24.0)
HC50 Management of depression	$48.02	66.0%	$31.72	13.2 (1.2)	0.0 (0.0)	0.0 (0.0)	0.0 (0.0)	0.0 (0.0)	2.6 (0.2)
HC51 Management of epilepsy	$53.73	66.0%	$35.48	8.7 (0.9)	0.0 (0.0)	0.0 (0.0)	0.0 (0.0)	0.0 (0.0)	1.7 (0.2)
HC52 Management of schizophrenia	$329.34	66.0%	$217.49	52.5 (52.5)	53.5 (48.8)	57.9 (0.3)	62.3 (0.0)	40.9 (0.0)	53.4 (20.3)
HC66 Psychosocial support & counseling	$21.32	66.0%	$14.08	1.7 (0.1)	0.0 (0.0)	0.0 (0.0)	0.0 (0.0)	0.0 (0.0)	0.3 (0.0)
Other NCDs				0.4 (0.0)	0.0 (0.0)	0.0 (0.0)	0.0 (0.0)	0.0 (0.0)	0.1 (0.0)
HC47 Palliative care	$21.32	83.4%	$17.78	0.4 (0.0)	0.0 (0.0)	0.0 (0.0)	0.0 (0.0)	0.0 (0.0)	0.1 (0.0)
Reproductive health				9.2 (3.6)	9.3 (0.0)	2.0 (0.0)	0.0 (0.0)	0.0 (0.0)	4.1 (0.7)
Family planning				0.1 (0.0)	0.0 (0.0)	0.0 (0.0)	0.0 (0.0)	0.0 (0.0)	0.0 (0.0)
HC4 Contraceptives	$10.32	67.6%	$6.98	0.1 (0.0)	0.0 (0.0)	0.0 (0.0)	0.0 (0.0)	0.0 (0.0)	0.0 (0.0)
Maternal conditions				15.2 (5.9)	15.6 (0.0)	3.3 (0.0)	0.0 (0.0)	0.0 (0.0)	6.8 (1.2)
C5 Antenatal tetanus immunization	$0.44	65.8%	$0.29	0.0 (0.0)	0.0 (0.0)	0.0 (0.0)	0.0 (0.0)	0.0 (0.0)	0.0 (0.0)
HC11 Basic emergency newborn & obstetric care	$145.10	65.8%	$95.54	91.0 (35.6)	93.3 (0.0)	19.9 (0.0)	0.0 (0.0)	0.0 (0.0)	40.9 (7.1)
HC2 Post-abortion care	$8.23	65.8%	$5.42	0.2 (0.0)	0.0 (0.0)	0.0 (0.0)	0.0 (0.0)	0.0 (0.0)	0.0 (0.0)
HC3 Treatment of premature membrane rupture	$4.66	65.8%	$3.07	0.0 (0.0)	0.0 (0.0)	0.0 (0.0)	0.0 (0.0)	0.0 (0.0)	0.0 (0.0)
HC5 Kangaroo mother care counseling	$4.61	65.8%	$3.04	0.0 (0.0)	0.0 (0.0)	0.0 (0.0)	0.0 (0.0)	0.0 (0.0)	0.0 (0.0)
HC7 Medical abortion	$5.52	65.8%	$3.63	0.1 (0.0)	0.0 (0.0)	0.0 (0.0)	0.0 (0.0)	0.0 (0.0)	0.0 (0.0)
Perinatal conditions				0.1 (0.0)	0.0 (0.0)	0.0 (0.0)	0.0 (0.0)	0.0 (0.0)	0.0 (0.0)
C13 Cotrimoxazole for HIV-exposed children	$11.95	48.6%	$5.81	0.2 (0.0)	0.0 (0.0)	0.0 (0.0)	0.0 (0.0)	0.0 (0.0)	0.0 (0.0)
HC1 Antibiotics for neonatal pneumonia	$6.61	48.6%	$3.22	0.0 (0.0)	0.0 (0.0)	0.0 (0.0)	0.0 (0.0)	0.0 (0.0)	0.0 (0.0)
HC6 Neonatal sepsis, pneumonia, meningitis	$3.51	48.6%	$1.71	0.0 (0.0)	0.0 (0.0)	0.0 (0.0)	0.0 (0.0)	0.0 (0.0)	0.0 (0.0)

Table B.31. Full model results: Tanzania.

Tanzania				CHE risk 10% threshold (25% threshold)					
Disease category, disease, intervention	Cost ($)	OOP (%)	OOP ($)	Q1	Q2	Q3	Q4	Q5	Total
Childhood health				0.0 (0.0)	0.0 (0.0)	0.0 (0.0)	0.0 (0.0)	0.0 (0.0)	0.0 (0.0)
Childhood health				0.0 (0.0)	0.0 (0.0)	0.0 (0.0)	0.0 (0.0)	0.0 (0.0)	0.0 (0.0)
HC42 Acute pharyngitis treatment	$0.17	27.1%	$0.05	0.0 (0.0)	0.0 (0.0)	0.0 (0.0)	0.0 (0.0)	0.0 (0.0)	0.0 (0.0)
Infectious & parasitic diseases				4.3 (0.6)	0.0 (0.0)	0.0 (0.0)	0.0 (0.0)	0.0 (0.0)	0.9 (0.1)
Diarrheal diseases				0.1 (0.0)	0.0 (0.0)	0.0 (0.0)	0.0 (0.0)	0.0 (0.0)	0.0 (0.0)
HC12 Diagnosis & treatment of infections (IMCI)	$4.79	42.4%	$2.03	0.1 (0.0)	0.0 (0.0)	0.0 (0.0)	0.0 (0.0)	0.0 (0.0)	0.0 (0.0)

Tanzania				CHE risk 10% threshold (25% threshold)					
Disease category, disease, intervention	Cost ($)	OOP (%)	OOP ($)	Q1	Q2	Q3	Q4	Q5	Total
HIV/AIDS & other sexually transmitted diseases				6.3 (0.8)	0.0 (0.0)	0.0 (0.0)	0.0 (0.0)	0.0 (0.0)	1.3 (0.2)
HC13 ART & viral load monitoring	$71.45	12.4%	$8.84	2.1 (0.2)	0.0 (0.0)	0.0 (0.0)	0.0 (0.0)	0.0 (0.0)	0.4 (0.0)
HC17 Syndromic management of STI	$5.67	12.4%	$0.70	0.0 (0.0)	0.0 (0.0)	0.0 (0.0)	0.0 (0.0)	0.0 (0.0)	0.0 (0.0)
HC23 HIV, STIs, hepatitis testing & counseling	$4.31	12.4%	$0.53	0.0 (0.0)	0.0 (0.0)	0.0 (0.0)	0.0 (0.0)	0.0 (0.0)	0.0 (0.0)
HC8 HIV & syphilis PMTCT	$176.35	12.4%	$21.81	23.1 (3.1)	0.0 (0.0)	0.0 (0.0)	0.0 (0.0)	0.0 (0.0)	4.6 (0.6)
Malaria				0.0 (0.0)	0.0 (0.0)	0.0 (0.0)	0.0 (0.0)	0.0 (0.0)	0.0 (0.0)
C7 Intermittent preventive treatment (pregnancy)	$0.45	20.3%	$0.09	0.0 (0.0)	0.0 (0.0)	0.0 (0.0)	0.0 (0.0)	0.0 (0.0)	0.0 (0.0)
Tuberculosis				8.9 (1.1)	0.0 (0.0)	0.0 (0.0)	0.0 (0.0)	0.0 (0.0)	1.8 (0.2)
HC27 Diagnosis & treatment of TB	$135.09	12.7%	$17.10	8.9 (1.1)	0.0 (0.0)	0.0 (0.0)	0.0 (0.0)	0.0 (0.0)	1.8 (0.2)
Other infectious & parasitic diseases				0.0 (0.0)	0.0 (0.0)	0.0 (0.0)	0.0 (0.0)	0.0 (0.0)	0.0 (0.0)
HC30 Management & referrals for fever (IMAI)	$3.11	19.8%	$0.62	0.0 (0.0)	0.0 (0.0)	0.0 (0.0)	0.0 (0.0)	0.0 (0.0)	0.0 (0.0)
Noncommunicable diseases (NCDs)				8.0 (3.9)	4.6 (1.7)	3.7 (0.0)	3.1 (0.0)	0.8 (0.0)	4.0 (1.1)
Cardiovascular diseases				14.0 (8.6)	10.4 (5.6)	8.5 (0.0)	9.7 (0.0)	2.6 (0.0)	9.0 (2.8)
HC38 Aspirin for acute myocardial infarction	$0.03	56.5%	$0.02	0.0 (0.0)	0.0 (0.0)	0.0 (0.0)	0.0 (0.0)	0.0 (0.0)	0.0 (0.0)
HC43 Management of ischemic heart disease	$83.97	56.5%	$47.48	21.1 (4.9)	7.4 (0.0)	0.0 (0.0)	0.0 (0.0)	0.0 (0.0)	5.7 (1.0)
HC44 Management of heart failure	$249.96	56.5%	$141.32	21.0 (21.0)	23.9 (16.8)	25.5 (0.0)	29.0 (0.0)	7.8 (0.0)	21.5 (7.6)
Endocrine & metabolic disorders				5.6 (0.9)	0.0 (0.0)	0.0 (0.0)	0.0 (0.0)	0.0 (0.0)	1.1 (0.2)
HC40 Screening & management of diabetes	$64.16	53.9%	$34.58	5.6 (0.9)	0.0 (0.0)	0.0 (0.0)	0.0 (0.0)	0.0 (0.0)	1.1 (0.2)
Mental/behavioral disorders & neurological conditions				5.0 (2.3)	3.0 (0.0)	2.2 (0.0)	0.4 (0.0)	0.0 (0.0)	2.1 (0.5)
HC49 Management of bipolar disorder	$184.57	50.5%	$93.19	9.2 (8.1)	10.4 (0.0)	11.0 (0.0)	1.8 (0.0)	0.0 (0.0)	6.5 (1.6)
HC50 Management of depression	$16.11	50.5%	$8.14	0.3 (0.0)	0.0 (0.0)	0.0 (0.0)	0.0 (0.0)	0.0 (0.0)	0.1 (0.0)
HC51 Management of epilepsy	$27.53	50.5%	$13.90	0.0 (0.0)	0.0 (0.0)	0.0 (0.0)	0.0 (0.0)	0.0 (0.0)	0.0 (0.0)
HC52 Management of schizophrenia	$99.43	50.5%	$50.20	9.2 (2.4)	4.5 (0.0)	0.0 (0.0)	0.0 (0.0)	0.0 (0.0)	2.7 (0.5)
HC66 Psychosocial support & counseling	$64.63	50.5%	$32.63	6.1 (0.9)	0.0 (0.0)	0.0 (0.0)	0.0 (0.0)	0.0 (0.0)	1.2 (0.2)
Other NCDs				7.6 (1.2)	0.0 (0.0)	0.0 (0.0)	0.0 (0.0)	0.0 (0.0)	1.5 (0.2)
HC47 Palliative care	$64.63	57.7%	$37.32	7.6 (1.2)	0.0 (0.0)	0.0 (0.0)	0.0 (0.0)	0.0 (0.0)	1.5 (0.2)
Reproductive health				1.3 (0.2)	0.0 (0.0)	0.0 (0.0)	0.0 (0.0)	0.0 (0.0)	0.3 (0.0)
Family planning				0.0 (0.0)	0.0 (0.0)	0.0 (0.0)	0.0 (0.0)	0.0 (0.0)	0.0 (0.0)
HC4 Contraceptives	$4.97	0.3%	$0.01	0.0 (0.0)	0.0 (0.0)	0.0 (0.0)	0.0 (0.0)	0.0 (0.0)	0.0 (0.0)
Maternal conditions				2.1 (0.3)	0.0 (0.0)	0.0 (0.0)	0.0 (0.0)	0.0 (0.0)	0.4 (0.1)
C5 Antenatal tetanus immunization	$0.39	29.9%	$0.12	0.0 (0.0)	0.0 (0.0)	0.0 (0.0)	0.0 (0.0)	0.0 (0.0)	0.0 (0.0)
HC11 Basic emergency newborn & obstetric care	$69.14	29.9%	$20.66	12.2 (1.6)	0.0 (0.0)	0.0 (0.0)	0.0 (0.0)	0.0 (0.0)	2.4 (0.3)
HC2 Post-abortion care	$4.54	29.9%	$1.36	0.0 (0.0)	0.0 (0.0)	0.0 (0.0)	0.0 (0.0)	0.0 (0.0)	0.0 (0.0)
HC3 Treatment of premature membrane rupture	$4.04	29.9%	$1.21	0.0 (0.0)	0.0 (0.0)	0.0 (0.0)	0.0 (0.0)	0.0 (0.0)	0.0 (0.0)
HC5 Kangaroo mother care counseling	$2.33	29.9%	$0.70	0.0 (0.0)	0.0 (0.0)	0.0 (0.0)	0.0 (0.0)	0.0 (0.0)	0.0 (0.0)
HC7 Medical abortion	$4.81	29.9%	$1.44	0.0 (0.0)	0.0 (0.0)	0.0 (0.0)	0.0 (0.0)	0.0 (0.0)	0.0 (0.0)
Perinatal conditions				0.1 (0.0)	0.0 (0.0)	0.0 (0.0)	0.0 (0.0)	0.0 (0.0)	0.0 (0.0)
C13 Cotrimoxazole for HIV-exposed children	$6.55	38.6%	$2.53	0.1 (0.0)	0.0 (0.0)	0.0 (0.0)	0.0 (0.0)	0.0 (0.0)	0.0 (0.0)
HC1 Antibiotics for neonatal pneumonia	$6.22	38.6%	$2.40	0.1 (0.0)	0.0 (0.0)	0.0 (0.0)	0.0 (0.0)	0.0 (0.0)	0.0 (0.0)
HC6 Neonatal sepsis, pneumonia, meningitis	$2.66	38.6%	$1.03	0.0 (0.0)	0.0 (0.0)	0.0 (0.0)	0.0 (0.0)	0.0 (0.0)	0.0 (0.0)

Table B.32. Full model results: Uganda.

Uganda				CHE risk 10% threshold (25% threshold)					
Disease category, disease, intervention	Cost ($)	OOP (%)	OOP ($)	Q1	Q2	Q3	Q4	Q5	Total
Childhood health				0.0 (0.0)	0.0 (0.0)	0.0 (0.0)	0.0 (0.0)	0.0 (0.0)	0.0 (0.0)
Childhood health				0.0 (0.0)	0.0 (0.0)	0.0 (0.0)	0.0 (0.0)	0.0 (0.0)	0.0 (0.0)
HC42 Acute pharyngitis treatment	$0.17	39.3%	$0.07	0.0 (0.0)	0.0 (0.0)	0.0 (0.0)	0.0 (0.0)	0.0 (0.0)	0.0 (0.0)
Infectious & parasitic diseases				2.9 (0.4)	0.0 (0.0)	0.0 (0.0)	0.0 (0.0)	0.0 (0.0)	0.6 (0.1)
Diarrheal diseases				0.4 (0.1)	0.0 (0.0)	0.0 (0.0)	0.0 (0.0)	0.0 (0.0)	0.1 (0.0)
HC12 Diagnosis & treatment of infections (IMCI)	$4.79	54.3%	$2.60	0.4 (0.1)	0.0 (0.0)	0.0 (0.0)	0.0 (0.0)	0.0 (0.0)	0.1 (0.0)
HIV/AIDS & other sexually transmitted diseases				5.6 (0.8)	0.0 (0.0)	0.0 (0.0)	0.0 (0.0)	0.0 (0.0)	1.1 (0.2)
HC13 ART & viral load monitoring	$71.45	8.1%	$5.77	2.5 (0.3)	0.0 (0.0)	0.0 (0.0)	0.0 (0.0)	0.0 (0.0)	0.5 (0.1)
HC17 Syndromic management of STI	$5.67	8.1%	$0.46	0.0 (0.0)	0.0 (0.0)	0.0 (0.0)	0.0 (0.0)	0.0 (0.0)	0.0 (0.0)
HC23 HIV, STIs, hepatitis testing & counseling	$4.31	8.1%	$0.35	0.0 (0.0)	0.0 (0.0)	0.0 (0.0)	0.0 (0.0)	0.0 (0.0)	0.0 (0.0)
HC8 HIV & syphilis PMTCT	$176.35	8.1%	$14.23	20.0 (2.8)	0.0 (0.0)	0.0 (0.0)	0.0 (0.0)	0.0 (0.0)	4.0 (0.6)
Malaria				0.0 (0.0)	0.0 (0.0)	0.0 (0.0)	0.0 (0.0)	0.0 (0.0)	0.0 (0.0)
C7 Intermittent preventive treatment (pregnancy)	$0.45	70.6%	$0.32	0.0 (0.0)	0.0 (0.0)	0.0 (0.0)	0.0 (0.0)	0.0 (0.0)	0.0 (0.0)
Tuberculosis				0.0 (0.0)	0.0 (0.0)	0.0 (0.0)	0.0 (0.0)	0.0 (0.0)	0.0 (0.0)
HC27 Diagnosis & treatment of TB	$135.09	0.0%	$0.04	0.0 (0.0)	0.0 (0.0)	0.0 (0.0)	0.0 (0.0)	0.0 (0.0)	0.0 (0.0)
Other infectious & parasitic diseases				0.1 (0.0)	0.0 (0.0)	0.0 (0.0)	0.0 (0.0)	0.0 (0.0)	0.0 (0.0)
HC30 Management & referrals for fever (IMAI)	$3.11	33.5%	$1.04	0.1 (0.0)	0.0 (0.0)	0.0 (0.0)	0.0 (0.0)	0.0 (0.0)	0.0 (0.0)
Noncommunicable diseases (NCDs)				3.9 (2.1)	2.9 (0.1)	1.6 (0.0)	0.5 (0.0)	0.0 (0.0)	1.8 (0.4)
Cardiovascular diseases				0.0 (0.0)	0.0 (0.0)	0.0 (0.0)	0.0 (0.0)	0.0 (0.0)	0.0 (0.0)
HC38 Aspirin for acute myocardial infarction	$0.03	0.1%	$0.00	0.0 (0.0)	0.0 (0.0)	0.0 (0.0)	0.0 (0.0)	0.0 (0.0)	0.0 (0.0)
HC43 Management of ischemic heart disease	$83.97	0.1%	$0.06	0.0 (0.0)	0.0 (0.0)	0.0 (0.0)	0.0 (0.0)	0.0 (0.0)	0.0 (0.0)
HC44 Management of heart failure	$249.96	0.1%	$0.17	0.0 (0.0)	0.0 (0.0)	0.0 (0.0)	0.0 (0.0)	0.0 (0.0)	0.0 (0.0)
Endocrine & metabolic disorders				0.0 (0.0)	0.0 (0.0)	0.0 (0.0)	0.0 (0.0)	0.0 (0.0)	0.0 (0.0)
HC40 Screening & management of diabetes	$64.16	0.1%	$0.06	0.0 (0.0)	0.0 (0.0)	0.0 (0.0)	0.0 (0.0)	0.0 (0.0)	0.0 (0.0)
Mental/behavioral disorders & neurological conditions				5.9 (3.0)	3.6 (0.2)	2.2 (0.0)	0.9 (0.0)	0.0 (0.0)	2.5 (0.6)
HC49 Management of bipolar disorder	$184.57	45.1%	$83.22	10.1 (10.1)	10.8 (0.8)	11.2 (0.0)	4.6 (0.0)	0.0 (0.0)	7.3 (2.2)
HC50 Management of depression	$16.11	45.1%	$7.26	0.6 (0.1)	0.0 (0.0)	0.0 (0.0)	0.0 (0.0)	0.0 (0.0)	0.1 (0.0)
HC51 Management of epilepsy	$27.53	45.1%	$12.41	0.0 (0.0)	0.0 (0.0)	0.0 (0.0)	0.0 (0.0)	0.0 (0.0)	0.0 (0.0)
HC52 Management of schizophrenia	$99.43	45.1%	$44.83	10.2 (3.6)	7.5 (0.0)	0.0 (0.0)	0.0 (0.0)	0.0 (0.0)	3.5 (0.7)
HC66 Psychosocial support & counseling	$64.63	45.1%	$29.14	8.5 (1.5)	0.0 (0.0)	0.0 (0.0)	0.0 (0.0)	0.0 (0.0)	1.7 (0.3)
Other NCDs				9.8 (6.1)	10.5 (0.0)	5.2 (0.0)	0.0 (0.0)	0.0 (0.0)	5.1 (1.2)
HC47 Palliative care	$64.63	93.6%	$60.50	9.8 (6.1)	10.5 (0.0)	5.2 (0.0)	0.0 (0.0)	0.0 (0.0)	5.1 (1.2)
Reproductive health				6.9 (2.0)	3.4 (0.0)	0.0 (0.0)	0.0 (0.0)	0.0 (0.0)	2.1 (0.4)
Family planning				0.0 (0.0)	0.0 (0.0)	0.0 (0.0)	0.0 (0.0)	0.0 (0.0)	0.0 (0.0)
HC4 Contraceptives	$4.97	0.0%	$0.00	0.0 (0.0)	0.0 (0.0)	0.0 (0.0)	0.0 (0.0)	0.0 (0.0)	0.0 (0.0)
Maternal conditions				11.3 (3.4)	5.7 (0.0)	0.0 (0.0)	0.0 (0.0)	0.0 (0.0)	3.4 (0.7)
C5 Antenatal tetanus immunization	$0.39	60.0%	$0.23	0.0 (0.0)	0.0 (0.0)	0.0 (0.0)	0.0 (0.0)	0.0 (0.0)	0.0 (0.0)
HC11 Basic emergency newborn & obstetric care	$69.14	60.0%	$41.49	66.1 (20.0)	34.0 (0.0)	0.0 (0.0)	0.0 (0.0)	0.0 (0.0)	20.0 (4.0)

Uganda				CHE risk 10% threshold (25% threshold)					
Disease category, disease, intervention	Cost ($)	OOP (%)	OOP ($)	Q1	Q2	Q3	Q4	Q5	Total
HC2 Post-abortion care	$4.54	60.0%	$2.73	0.4 (0.1)	0.0 (0.0)	0.0 (0.0)	0.0 (0.0)	0.0 (0.0)	0.1 (0.0)
HC3 Treatment of premature membrane rupture	$4.04	60.0%	$2.42	0.3 (0.0)	0.0 (0.0)	0.0 (0.0)	0.0 (0.0)	0.0 (0.0)	0.1 (0.0)
HC5 Kangaroo mother care counseling	$2.33	60.0%	$1.40	0.1 (0.0)	0.0 (0.0)	0.0 (0.0)	0.0 (0.0)	0.0 (0.0)	0.0 (0.0)
HC7 Medical abortion	$4.81	60.0%	$2.88	0.7 (0.1)	0.0 (0.0)	0.0 (0.0)	0.0 (0.0)	0.0 (0.0)	0.1 (0.0)
Perinatal conditions				0.5 (0.1)	0.0 (0.0)	0.0 (0.0)	0.0 (0.0)	0.0 (0.0)	0.1 (0.0)
C13 Cotrimoxazole for HIV-exposed children	$6.55	67.6%	$4.43	0.7 (0.1)	0.0 (0.0)	0.0 (0.0)	0.0 (0.0)	0.0 (0.0)	0.1 (0.0)
HC1 Antibiotics for neonatal pneumonia	$6.22	67.6%	$4.21	0.6 (0.1)	0.0 (0.0)	0.0 (0.0)	0.0 (0.0)	0.0 (0.0)	0.1 (0.0)
HC6 Neonatal sepsis, pneumonia, meningitis	$2.66	67.6%	$1.80	0.1 (0.0)	0.0 (0.0)	0.0 (0.0)	0.0 (0.0)	0.0 (0.0)	0.0 (0.0)

Table B.33. Full model results: Viet Nam.

Viet Nam				CHE risk 10% threshold (25% threshold)					
Disease category, disease, intervention	Cost ($)	OOP (%)	OOP ($)	Q1	Q2	Q3	Q4	Q5	Total
Childhood health				0.0 (0.0)	0.0 (0.0)	0.0 (0.0)	0.0 (0.0)	0.0 (0.0)	0.0 (0.0)
Childhood health				0.0 (0.0)	0.0 (0.0)	0.0 (0.0)	0.0 (0.0)	0.0 (0.0)	0.0 (0.0)
HC42 Acute pharyngitis treatment	$0.24	45.3%	$0.11	0.0 (0.0)	0.0 (0.0)	0.0 (0.0)	0.0 (0.0)	0.0 (0.0)	0.0 (0.0)
Infectious & parasitic diseases				1.4 (0.1)	0.0 (0.0)	0.0 (0.0)	0.0 (0.0)	0.0 (0.0)	0.3 (0.0)
Diarrheal diseases				0.1 (0.0)	0.0 (0.0)	0.0 (0.0)	0.0 (0.0)	0.0 (0.0)	0.0 (0.0)
HC12 Diagnosis & treatment of infections (IMCI)	$10.29	72.7%	$7.48	0.1 (0.0)	0.0 (0.0)	0.0 (0.0)	0.0 (0.0)	0.0 (0.0)	0.0 (0.0)
HIV/AIDS & other sexually transmitted diseases				0.2 (0.0)	0.0 (0.0)	0.0 (0.0)	0.0 (0.0)	0.0 (0.0)	0.0 (0.0)
HC13 ART & viral load monitoring	$121.57	4.7%	$5.75	0.0 (0.0)	0.0 (0.0)	0.0 (0.0)	0.0 (0.0)	0.0 (0.0)	0.0 (0.0)
HC17 Syndromic management of STI	$10.69	4.7%	$0.51	0.0 (0.0)	0.0 (0.0)	0.0 (0.0)	0.0 (0.0)	0.0 (0.0)	0.0 (0.0)
HC23 HIV, STIs, hepatitis testing & counseling	$6.08	4.7%	$0.29	0.0 (0.0)	0.0 (0.0)	0.0 (0.0)	0.0 (0.0)	0.0 (0.0)	0.0 (0.0)
HC8 HIV & syphilis PMTCT	$313.51	4.7%	$14.83	0.6 (0.1)	0.0 (0.0)	0.0 (0.0)	0.0 (0.0)	0.0 (0.0)	0.1 (0.0)
Malaria				0.0 (0.0)	0.0 (0.0)	0.0 (0.0)	0.0 (0.0)	0.0 (0.0)	0.0 (0.0)
C7 Intermittent preventive treatment (pregnancy)	$1.02	5.9%	$0.06	0.0 (0.0)	0.0 (0.0)	0.0 (0.0)	0.0 (0.0)	0.0 (0.0)	0.0 (0.0)
Tuberculosis				10.6 (1.1)	0.0 (0.0)	0.0 (0.0)	0.0 (0.0)	0.0 (0.0)	2.1 (0.2)
HC27 Diagnosis & treatment of TB	$175.65	30.1%	$52.85	10.6 (1.1)	0.0 (0.0)	0.0 (0.0)	0.0 (0.0)	0.0 (0.0)	2.1 (0.2)
Other infectious & parasitic diseases				0.0 (0.0)	0.0 (0.0)	0.0 (0.0)	0.0 (0.0)	0.0 (0.0)	0.0 (0.0)
HC30 Management & referrals for fever (IMAI)	$6.83	46.4%	$3.17	0.0 (0.0)	0.0 (0.0)	0.0 (0.0)	0.0 (0.0)	0.0 (0.0)	0.0 (0.0)
Noncommunicable diseases (NCDs)				12.3 (9.5)	13.1 (0.2)	11.3 (0.0)	6.0 (0.0)	0.0 (0.0)	8.5 (1.9)
Cardiovascular diseases				16.3 (10.6)	17.0 (0.5)	9.8 (0.0)	7.0 (0.0)	0.0 (0.0)	10.0 (2.2)
HC38 Aspirin for acute myocardial infarction	$0.05	80.5%	$0.04	0.0 (0.0)	0.0 (0.0)	0.0 (0.0)	0.0 (0.0)	0.0 (0.0)	0.0 (0.0)
HC43 Management of ischemic heart disease	$190.17	80.5%	$153.03	24.4 (7.5)	22.8 (0.0)	0.0 (0.0)	0.0 (0.0)	0.0 (0.0)	9.4 (1.5)
HC44 Management of heart failure	$342.48	80.5%	$275.59	24.4 (24.2)	28.3 (1.4)	29.5 (0.0)	20.9 (0.0)	0.0 (0.0)	20.6 (5.1)
Endocrine & metabolic disorders				1.5 (0.2)	0.0 (0.0)	0.0 (0.0)	0.0 (0.0)	0.0 (0.0)	0.3 (0.0)
HC40 Screening & management of diabetes	$92.52	78.4%	$72.53	1.5 (0.2)	0.0 (0.0)	0.0 (0.0)	0.0 (0.0)	0.0 (0.0)	0.3 (0.0)
Mental/behavioral disorders & neurological conditions				14.6 (12.6)	16.0 (0.2)	16.7 (0.0)	7.8 (0.0)	0.0 (0.0)	11.0 (2.6)
HC49 Management of bipolar disorder	$365.39	73.9%	$270.01	34.8 (34.0)	39.9 (1.1)	41.7 (0.0)	27.0 (0.0)	0.0 (0.0)	28.7 (7.0)

Viet Nam				CHE risk 10% threshold (25% threshold)					
Disease category, disease, intervention	Cost ($)	OOP (%)	OOP ($)	Q1	Q2	Q3	Q4	Q5	Total
HC50 Management of depression	$48.02	73.9%	$35.49	2.9 (0.3)	0.0 (0.0)	0.0 (0.0)	0.0 (0.0)	0.0 (0.0)	0.6 (0.1)
HC51 Management of epilepsy	$53.73	73.9%	$39.70	0.3 (0.0)	0.0 (0.0)	0.0 (0.0)	0.0 (0.0)	0.0 (0.0)	0.1 (0.0)
HC52 Management of schizophrenia	$329.34	73.9%	$243.37	34.7 (28.5)	39.9 (0.0)	41.9 (0.0)	11.8 (0.0)	0.0 (0.0)	25.7 (5.7)
HC66 Psychosocial support & counseling	$21.32	73.9%	$15.75	0.4 (0.0)	0.0 (0.0)	0.0 (0.0)	0.0 (0.0)	0.0 (0.0)	0.1 (0.0)
Other NCDs				0.1 (0.0)	0.0 (0.0)	0.0 (0.0)	0.0 (0.0)	0.0 (0.0)	0.0 (0.0)
HC47 Palliative care	$21.32	81.2%	$17.31	0.1 (0.0)	0.0 (0.0)	0.0 (0.0)	0.0 (0.0)	0.0 (0.0)	0.0 (0.0)
Reproductive health				5.6 (0.8)	0.3 (0.0)	0.0 (0.0)	0.0 (0.0)	0.0 (0.0)	1.2 (0.2)
Family planning				0.1 (0.0)	0.0 (0.0)	0.0 (0.0)	0.0 (0.0)	0.0 (0.0)	0.0 (0.0)
HC4 Contraceptives	$10.32	59.9%	$6.18	0.1 (0.0)	0.0 (0.0)	0.0 (0.0)	0.0 (0.0)	0.0 (0.0)	0.0 (0.0)
Maternal conditions				9.2 (1.3)	0.5 (0.0)	0.0 (0.0)	0.0 (0.0)	0.0 (0.0)	1.9 (0.3)
C5 Antenatal tetanus immunization	$0.44	75.3%	$0.33	0.0 (0.0)	0.0 (0.0)	0.0 (0.0)	0.0 (0.0)	0.0 (0.0)	0.0 (0.0)
HC11 Basic emergency newborn & obstetric care	$145.10	75.3%	$109.31	55.3 (7.9)	2.8 (0.0)	0.0 (0.0)	0.0 (0.0)	0.0 (0.0)	11.6 (1.6)
HC2 Post-abortion care	$8.23	75.3%	$6.20	0.0 (0.0)	0.0 (0.0)	0.0 (0.0)	0.0 (0.0)	0.0 (0.0)	0.0 (0.0)
HC3 Treatment of premature membrane rupture	$4.66	75.3%	$3.51	0.0 (0.0)	0.0 (0.0)	0.0 (0.0)	0.0 (0.0)	0.0 (0.0)	0.0 (0.0)
HC5 Kangaroo mother care counseling	$4.61	75.3%	$3.47	0.0 (0.0)	0.0 (0.0)	0.0 (0.0)	0.0 (0.0)	0.0 (0.0)	0.0 (0.0)
HC7 Medical abortion	$5.52	75.3%	$4.16	0.0 (0.0)	0.0 (0.0)	0.0 (0.0)	0.0 (0.0)	0.0 (0.0)	0.0 (0.0)
Perinatal conditions				0.0 (0.0)	0.0 (0.0)	0.0 (0.0)	0.0 (0.0)	0.0 (0.0)	0.0 (0.0)
C13 Cotrimoxazole for HIV-exposed children	$11.95	55.2%	$6.60	0.1 (0.0)	0.0 (0.0)	0.0 (0.0)	0.0 (0.0)	0.0 (0.0)	0.0 (0.0)
HC1 Antibiotics for neonatal pneumonia	$6.61	55.2%	$3.65	0.0 (0.0)	0.0 (0.0)	0.0 (0.0)	0.0 (0.0)	0.0 (0.0)	0.0 (0.0)
HC6 Neonatal sepsis, pneumonia, meningitis	$3.51	55.2%	$1.94	0.0 (0.0)	0.0 (0.0)	0.0 (0.0)	0.0 (0.0)	0.0 (0.0)	0.0 (0.0)

Table B.34. Full model results: Zimbabwe.

Zimbabwe				CHE risk 10% threshold (25% threshold)					
Disease category, disease, intervention	Cost ($)	OOP (%)	OOP ($)	Q1	Q2	Q3	Q4	Q5	Total
Childhood health				0.0 (0.0)	0.0 (0.0)	0.0 (0.0)	0.0 (0.0)	0.0 (0.0)	0.0 (0.0)
Childhood health				0.0 (0.0)	0.0 (0.0)	0.0 (0.0)	0.0 (0.0)	0.0 (0.0)	0.0 (0.0)
HC42 Acute pharyngitis treatment	$0.17	39.3%	$0.07	0.0 (0.0)	0.0 (0.0)	0.0 (0.0)	0.0 (0.0)	0.0 (0.0)	0.0 (0.0)
Infectious & parasitic diseases				19.6 (7.0)	9.6 (0.0)	0.0 (0.0)	0.0 (0.0)	0.0 (0.0)	5.8 (1.4)
Diarrheal diseases				0.4 (0.1)	0.0 (0.0)	0.0 (0.0)	0.0 (0.0)	0.0 (0.0)	0.1 (0.0)
HC12 Diagnosis & treatment of infections (IMCI)	$4.79	42.4%	$2.03	0.4 (0.1)	0.0 (0.0)	0.0 (0.0)	0.0 (0.0)	0.0 (0.0)	0.1 (0.0)
HIV/AIDS & other sexually transmitted diseases				30.1 (12.1)	19.2 (0.0)	0.0 (0.0)	0.0 (0.0)	0.0 (0.0)	9.9 (2.4)
HC13 ART & viral load monitoring	$71.45	23.0%	$16.41	35.1 (6.9)	0.0 (0.0)	0.0 (0.0)	0.0 (0.0)	0.0 (0.0)	7.0 (1.4)
HC17 Syndromic management of STI	$5.67	23.0%	$1.30	0.0 (0.0)	0.0 (0.0)	0.0 (0.0)	0.0 (0.0)	0.0 (0.0)	0.0 (0.0)
HC23 HIV, STIs, hepatitis testing & counseling	$4.31	23.0%	$0.99	0.2 (0.0)	0.0 (0.0)	0.0 (0.0)	0.0 (0.0)	0.0 (0.0)	0.0 (0.0)
HC8 HIV & syphilis PMTCT	$176.35	23.0%	$40.50	85.0 (41.6)	76.9 (0.0)	0.1 (0.0)	0.0 (0.0)	0.0 (0.0)	32.4 (8.3)
Malaria				0.0 (0.0)	0.0 (0.0)	0.0 (0.0)	0.0 (0.0)	0.0 (0.0)	0.0 (0.0)
C7 Intermittent preventive treatment (pregnancy)	$0.45	25.6%	$0.12	0.0 (0.0)	0.0 (0.0)	0.0 (0.0)	0.0 (0.0)	0.0 (0.0)	0.0 (0.0)
Tuberculosis				35.9 (7.1)	0.0 (0.0)	0.0 (0.0)	0.0 (0.0)	0.0 (0.0)	7.2 (1.4)

**Zimbabwe**				**CHE risk** 10% threshold (25% threshold)					
Disease category, disease, intervention	Cost ($)	OOP (%)	OOP ($)	Q1	Q2	Q3	Q4	Q5	Total
HC27 Diagnosis & treatment of TB	$135.09	12.7%	$17.10	35.9 (7.1)	0.0 (0.0)	0.0 (0.0)	0.0 (0.0)	0.0 (0.0)	7.2 (1.4)
Other infectious & parasitic diseases				0.0 (0.0)	0.0 (0.0)	0.0 (0.0)	0.0 (0.0)	0.0 (0.0)	0.0 (0.0)
HC30 Management & referrals for fever (IMAI)	$3.11	19.8%	$0.62	0.0 (0.0)	0.0 (0.0)	0.0 (0.0)	0.0 (0.0)	0.0 (0.0)	0.0 (0.0)
Noncommunicable diseases (NCDs)				8.4 (5.2)	6.6 (2.2)	3.4 (1.1)	2.7 (0.0)	1.3 (0.0)	4.5 (1.7)
Cardiovascular diseases				9.5 (7.8)	9.8 (4.9)	6.2 (3.5)	5.2 (0.0)	4.2 (0.0)	7.0 (3.2)
HC38 Aspirin for acute myocardial infarction	$0.03	56.5%	$0.02	0.0 (0.0)	0.0 (0.0)	0.0 (0.0)	0.0 (0.0)	0.0 (0.0)	0.0 (0.0)
HC43 Management of ischemic heart disease	$83.97	56.5%	$47.48	14.2 (9.0)	14.8 (0.0)	3.9 (0.0)	0.0 (0.0)	0.0 (0.0)	6.6 (1.8)
HC44 Management of heart failure	$249.96	56.5%	$141.32	14.2 (14.2)	14.7 (14.7)	14.8 (10.6)	15.7 (0.0)	12.7 (0.0)	14.4 (7.9)
Endocrine & metabolic disorders				4.9 (1.8)	2.6 (0.0)	0.0 (0.0)	0.0 (0.0)	0.0 (0.0)	1.5 (0.4)
HC40 Screening & management of diabetes	$64.16	53.9%	$34.58	4.9 (1.8)	2.6 (0.0)	0.0 (0.0)	0.0 (0.0)	0.0 (0.0)	1.5 (0.4)
Mental/behavioral disorders & neurological conditions				8.2 (4.6)	5.2 (1.5)	3.0 (0.0)	2.2 (0.0)	0.1 (0.0)	3.7 (1.2)
HC49 Management of bipolar disorder	$184.57	50.5%	$93.19	10.6 (10.6)	10.8 (7.4)	10.9 (0.0)	11.2 (0.0)	0.4 (0.0)	8.8 (3.6)
HC50 Management of depression	$16.11	50.5%	$8.14	1.6 (0.3)	0.0 (0.0)	0.0 (0.0)	0.0 (0.0)	0.0 (0.0)	0.3 (0.1)
HC51 Management of epilepsy	$27.53	50.5%	$13.90	7.8 (1.5)	0.0 (0.0)	0.0 (0.0)	0.0 (0.0)	0.0 (0.0)	1.6 (0.3)
HC52 Management of schizophrenia	$99.43	50.5%	$50.20	10.4 (7.2)	10.8 (0.0)	4.3 (0.0)	0.0 (0.0)	0.0 (0.0)	5.1 (1.4)
HC66 Psychosocial support & counseling	$64.63	50.5%	$32.63	10.4 (3.5)	4.6 (0.0)	0.0 (0.0)	0.0 (0.0)	0.0 (0.0)	3.0 (0.7)
Other NCDs				10.2 (4.3)	7.3 (0.0)	0.0 (0.0)	0.0 (0.0)	0.0 (0.0)	3.5 (0.9)
HC47 Palliative care	$64.63	57.7%	$37.32	10.2 (4.3)	7.3 (0.0)	0.0 (0.0)	0.0 (0.0)	0.0 (0.0)	3.5 (0.9)
Reproductive health				5.0 (1.0)	0.0 (0.0)	0.0 (0.0)	0.0 (0.0)	0.0 (0.0)	1.0 (0.2)
Family planning				0.0 (0.0)	0.0 (0.0)	0.0 (0.0)	0.0 (0.0)	0.0 (0.0)	0.0 (0.0)
HC4 Contraceptives	$4.97	0.3%	$0.01	0.0 (0.0)	0.0 (0.0)	0.0 (0.0)	0.0 (0.0)	0.0 (0.0)	0.0 (0.0)
Maternal conditions				8.2 (1.7)	0.0 (0.0)	0.0 (0.0)	0.0 (0.0)	0.0 (0.0)	1.6 (0.3)
C5 Antenatal tetanus immunization	$0.39	29.9%	$0.12	0.0 (0.0)	0.0 (0.0)	0.0 (0.0)	0.0 (0.0)	0.0 (0.0)	0.0 (0.0)
HC11 Basic emergency newborn & obstetric care	$69.14	29.9%	$20.66	47.8 (9.9)	0.0 (0.0)	0.0 (0.0)	0.0 (0.0)	0.0 (0.0)	9.6 (2.0)
HC2 Post-abortion care	$4.54	29.9%	$1.36	0.3 (0.0)	0.0 (0.0)	0.0 (0.0)	0.0 (0.0)	0.0 (0.0)	0.1 (0.0)
HC3 Treatment of premature membrane rupture	$4.04	29.9%	$1.21	0.2 (0.0)	0.0 (0.0)	0.0 (0.0)	0.0 (0.0)	0.0 (0.0)	0.0 (0.0)
HC5 Kangaroo mother care counseling	$2.33	29.9%	$0.70	0.1 (0.0)	0.0 (0.0)	0.0 (0.0)	0.0 (0.0)	0.0 (0.0)	0.0 (0.0)
HC7 Medical abortion	$4.81	29.9%	$1.44	0.5 (0.1)	0.0 (0.0)	0.0 (0.0)	0.0 (0.0)	0.0 (0.0)	0.1 (0.0)
Perinatal conditions				0.4 (0.1)	0.0 (0.0)	0.0 (0.0)	0.0 (0.0)	0.0 (0.0)	0.1 (0.0)
C13 Cotrimoxazole for HIV-exposed children	$6.55	38.6%	$2.53	0.5 (0.1)	0.0 (0.0)	0.0 (0.0)	0.0 (0.0)	0.0 (0.0)	0.1 (0.0)
HC1 Antibiotics for neonatal pneumonia	$6.22	38.6%	$2.40	0.5 (0.1)	0.0 (0.0)	0.0 (0.0)	0.0 (0.0)	0.0 (0.0)	0.1 (0.0)
HC6 Neonatal sepsis, pneumonia, meningitis	$2.66	38.6%	$1.03	0.1 (0.0)	0.0 (0.0)	0.0 (0.0)	0.0 (0.0)	0.0 (0.0)	0.0 (0.0)
